# Construction of Vicinal Quaternary Centers via Ru-Catalyzed Enantiospecific Allylic Substitution with Lithium Ester Enolates

**DOI:** 10.1021/jacs.4c07690

**Published:** 2024-08-19

**Authors:** Sven M. Papidocha, Erick M. Carreira

**Affiliations:** Department of Chemistry and Applied Biosciences, Laboratory of Organic Chemistry, ETH Zürich, Zürich 8093, Switzerland

## Abstract

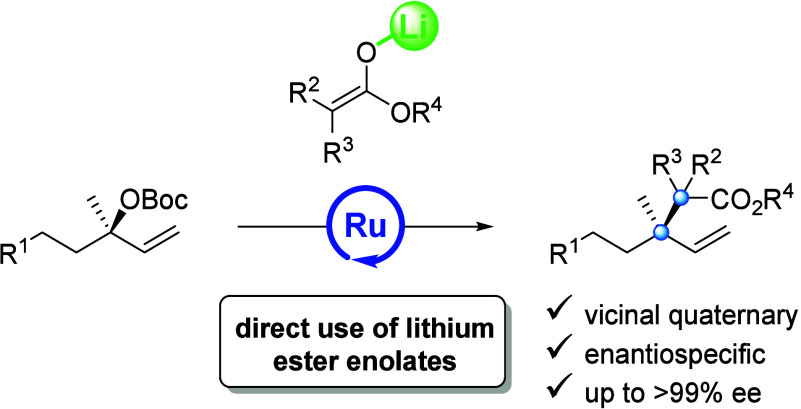

The installation of vicinal quaternary centers with absolute stereocontrol constitutes a considerable challenge in organic synthesis. Herein, we introduce a novel [Cp*Ru(MeCN)_3_]PF_6_/phenoxythiazoline catalyst system that achieves enantiospecific allylic substitution of tertiary carbonates with α,α-disubstituted lithium ester enolates to give products containing vicinal quaternary centers. Noteworthy features include the direct use of nonstabilized ester enolates, a class of nucleophiles which has rarely been used in transition metal-catalyzed allylic substitution reactions. The approach is demonstrated for a broad scope of tertiary electrophiles as well as ester enolates and accomplishes stereoretentive substitution with excellent conservation of ee (89–99%) and branched/linear regioselectivities (up to 40:1).

Transition metal-catalyzed allylic and related allenylic substitution reactions have found widespread application in enantioselective synthesis for a broad range of settings. These reactions have been deployed for the stereocontrolled construction of carbon–heteroatom (–N, –O, –S) as well as carbon–carbon bonds,^1^ with some methods providing access to quaternary centers.^2^ The stereocontrolled synthesis of fragments that incorporate vicinal quaternary centers remains a significant challenge both for the general field of asymmetric synthesis^3^ and also, specifically, in transition metal-mediated allylic substitution reactions.^4^ In general, the field has been dominated by catalysts derived from Pd,^5^ Ir,^6^ and Rh.^7^ Herein, we present the first enantiospecific Ru-catalyzed substitution of *tert*-butyl allyl carbonates with lithium ester enolates. The use of this underrepresented class of nucleophiles provides access to products that feature vicinal quaternary centers (Scheme 1). A novel feature of our approach is the direct use of ester enolates in combination with Ru catalysis in allylic substitution reactions. The catalyst is conveniently generated *in situ* from [Cp*Ru(MeCN)_3_]PF_6_ and phenoxythiazoline **L1**.^8^ The enantiospecific transformation delivers optically active adducts for a broad range of α,α-disubstituted ester enolates and tertiary allylic electrophiles.

**Scheme 1:**
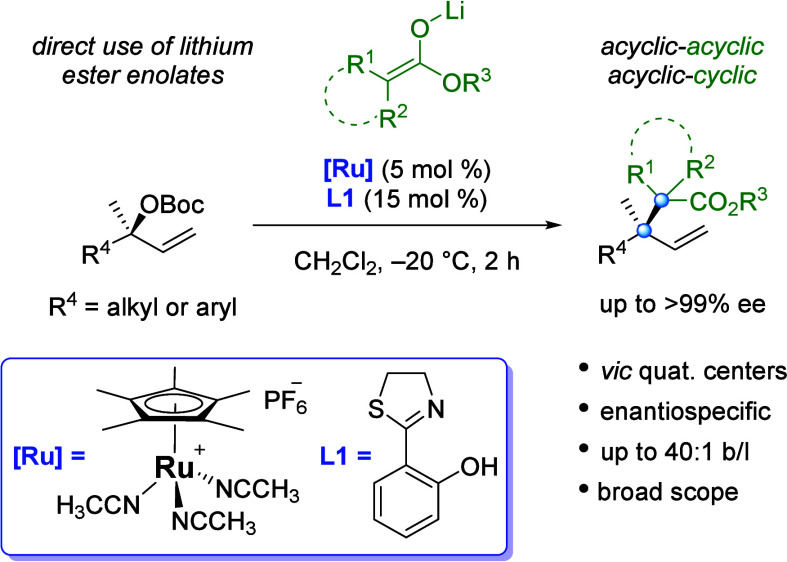
Enantiospecific Ru-cat. Allylic Substitution of Ester Enolates and Tertiary Carbonates

The stereocontrolled synthesis of quaternary centers continues to challenge organic chemists, especially for the construction of complex scaffolds as encountered in many natural products and bioactive molecules.^9^ This challenge is further compounded for targets featuring vicinal quaternary centers,^3^ as exemplified by lingzhiol,^10^ cuparenone,^11^ and trichodiene.^12^ Although asymmetric allylic substitutions have been broadly applied to the synthesis of methine and quaternary centers,^2^ access to vicinal quaternary centers is limited (Scheme 2). In the field, the synthesis of acyclic vicinal quaternary centers has been perceived as a particular challenge.^13^ Asymmetric allylic substitutions based on Pd and Ir catalysts have been utilized for the synthesis of acyclic-cyclic vicinal quaternary centers.^4a−4d,4g^ Two reports have demonstrated the synthesis of products incorporating acyclic-acyclic vicinal quaternary centers. In a study of the regiochemistry of Ru-catalyzed allylic substitutions of secondary allylic esters and carbonates, Kawatsura and Itoh included as a singular example a reaction that gave acyclic vicinal quaternary centers with a malonate as nucleophile.^4h^ In 2018, Stoltz reported the first enantioselective allylic substitution reaction leading to acyclic vicinal quaternary centers using an Ir catalyst with malononitriles and substituted cinnamyl carbonates.^4e^ Additionally, Ir-catalyzed asymmetric synthesis has been demonstrated for related allenylic systems from silyl ketene acetals^15^ and racemic allenylic alcohols.^16^

**Scheme 2:**
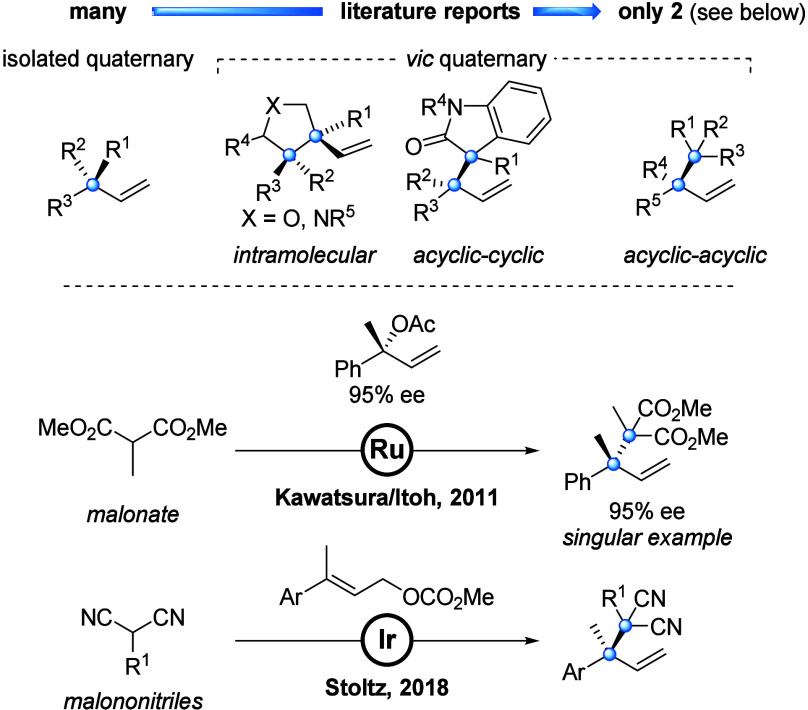
Asymmetric Allylic Substitutions: Vicinal Quaternary Centers

Transition metal-catalyzed allylic substitutions are effected predominantly with nucleophiles such as malonates, malononitriles, and β-ketoesters.^17^ By contrast, there are few reports on the direct use of less stabilized enolates, such as ester enolates.^18^ Our long-standing interest in Ir-catalyzed allylic substitutions has compelled us to explore alternative metals. We reasoned that the use of new catalyst systems could expand the scope of nucleophiles, electrophiles (nonbenzylic), and thereby products. We were particularly attracted to Ru because it is relatively underexplored in enantioselective allylic substitutions compared to Pd and Ir.^1c,19^ Furthermore, we wanted to investigate the application of nonstabilized ester enolates and tertiary allylic electrophiles as the latter are also underrepresented in asymmetric allylic substitutions.

Our investigation was launched with the examination of various Ru catalyst precursors (see the Supporting Information). We found that treatment of tertiary carbonate **1a** (>99% ee) with [Cp*Ru(MeCN)_3_]PF_6_^20^ (5 mol %) and lithium ester enolate **2a** (3 equiv in 1.5:1 hexane–THF) in CH_2_Cl_2_ at −20 °C resulted in formation of product **3a**, albeit in very low yield (9%), surprisingly favoring the linear product (b/l = 1:2), and with marked erosion of ee (Table 1, entry 1). Inspired by the work of Bruneau^21^ and You,^22^ we chose to investigate quinaldic acid (**L0**) as ligand. Employing 15 mol % quinaldic acid (entry 2) significantly improved yield (32%), b/l ratio (7:1), and ee (55%). Hence, we explored the use of various other putative N/O-chelating ligands (see the Supporting Information) and found that phenoxythiazoline **L1**(8) (15 mol %) leads to optimal reaction performance (entry 3). In presence of **L1**, product **3a** was formed in excellent yield (92%), b/l ratio (40:1), and ee (>99%). In the absence of [Cp*Ru(MeCN)_3_]PF_6_ no reaction was observed (entry 4). When related catalyst precursor [CpRu(MeCN)_3_]PF_6_ was used, yield (11%), b/l ratio (1:1) and ee (79%) were significantly diminished (entry 5). Running the reaction with ligands **L2**,^8,23^**L3**,^24^ or **L4**(25) decreased yield, b/l ratio, and ee (entries 6–8). Use of different solvents, such as PhMe and THF influenced the regioselectivity (entries 9–10). Conducting the reaction at 23 °C (entry 11) led to a drop in yield (21%) of product **3a** and the formation of elimination products (diene isomers, 60% yield) derived from **1a**, presumably resulting from β-elimination from the allyl complex.^26^

**Table 1:**
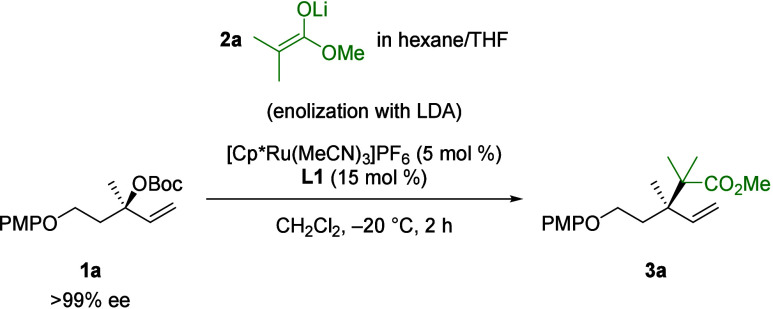
Optimization Study of the Ru-Catalyzed Allylation^a^

entry	deviation from standard conditions	yield^b^ (%)	b/l^c^	ee^d^(%)
1	no **L1**	9	1:2	29
2	**L0** instead of **L1**	32	7:1	55
3	none	92	40:1	>99
4	no [Ru]	0	-	-
5	[**X**Ru (MeCN)_3_]PF_6_**X** = Cp instead of Cp*	11	1:1	79
6	**L2** instead of **L1**	81	30:1	>99
7	**L3** instead of **L1**	68	30:1	98
8	**L4** instead of **L1**	30	7:1	34
9	PhMe instead of CH_2_Cl_2_	80	8:1	>99
10	THF instead of CH_2_Cl_2_	67	5:1	97
11	23 °C instead of –20 °C	21	14:1	95

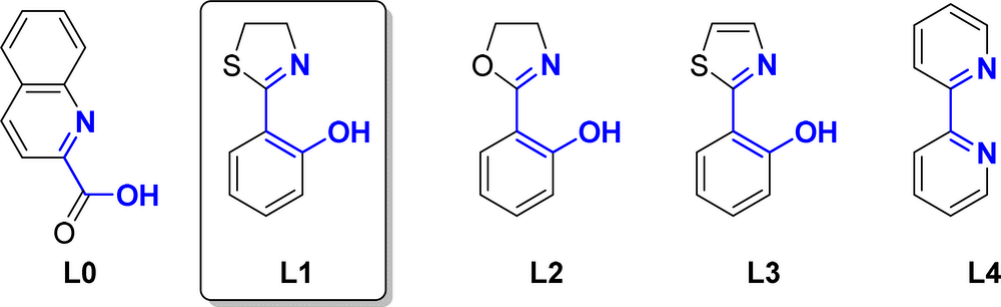

a Standard conditions: Allylic carbonate **1a** (0.2 mmol), [Cp*Ru(MeCN)_3_]PF_6_ (5 mol %), phenoxythiazoline **L1** (15 mol %), lithium ester enolate **2a** (3 equiv, 0.9 M in 1.5:1 hexane–THF), CH_2_Cl_2_ (0.2 M), –20 °C, 2 h. PMP = *para*-methoxyphenyl.

b Yield was determined by analysis of the ^1^H NMR spectra of the unpurified reaction mixture using phenanthrene as an internal standard.

c b/l refers to ratios of branched/linear isomers determined by analysis of the ^1^H NMR spectra of the unpurified reaction mixtures.

d Determined by SFC analysis using a chiral stationary phase.

With the optimized conditions in hand, we studied the scope of the reaction. A wide variety of α,α-disubstituted ester enolates were found to undergo Ru-catalyzed allylic substitution (Scheme 3). Ester enolates along the series of methyl (**2a**), ethyl (**2b**), *iso*-propyl (**2c**), and *tert*-butyl (**2d**) provided product in 81–94% yield and b/l ratios in the range of 25:1–40:1, while enantiomeric excess was completely conserved (>99% ee). The excellent performance of *tert*-butyl ester enolate **2d** is noteworthy since in earlier reports either no reaction^16^ or a lowered yield^15d^ was observed for reaction of the silyl ketene acetal of *tert*-butyl isobutyrate. We next examined ester enolates bearing other α-substituents as nucleophiles. α,α-Diethyl ester **3e** was obtained in slightly lowered b/l of 20:1 and 73% yield. The substitution reaction with cyclic substrates showed that 5-, 6- and 7-membered rings are well tolerated. The reaction of lithium ester enolate **2h** provided dihydropyran **3h** in 47% yield with 20:1 b/l. Cyclohexane-derived products **3i**-**3k** were all obtained in yields of ≥80% and b/l of ≥30:1. The absolute configurations of **3i** and **3j** were determined by X-ray crystallographic analysis and confirmed an overall stereoretentive process as reported for related Ru-based systems.^4h,27^ Notably, the symmetric 4-*tert-*butyl cyclohexane **3k** was obtained as a single diastereoisomer. Finally, cycloheptane **3l** was isolated in a yield of 75% and 30:1 b/l.

**Scheme 3:**
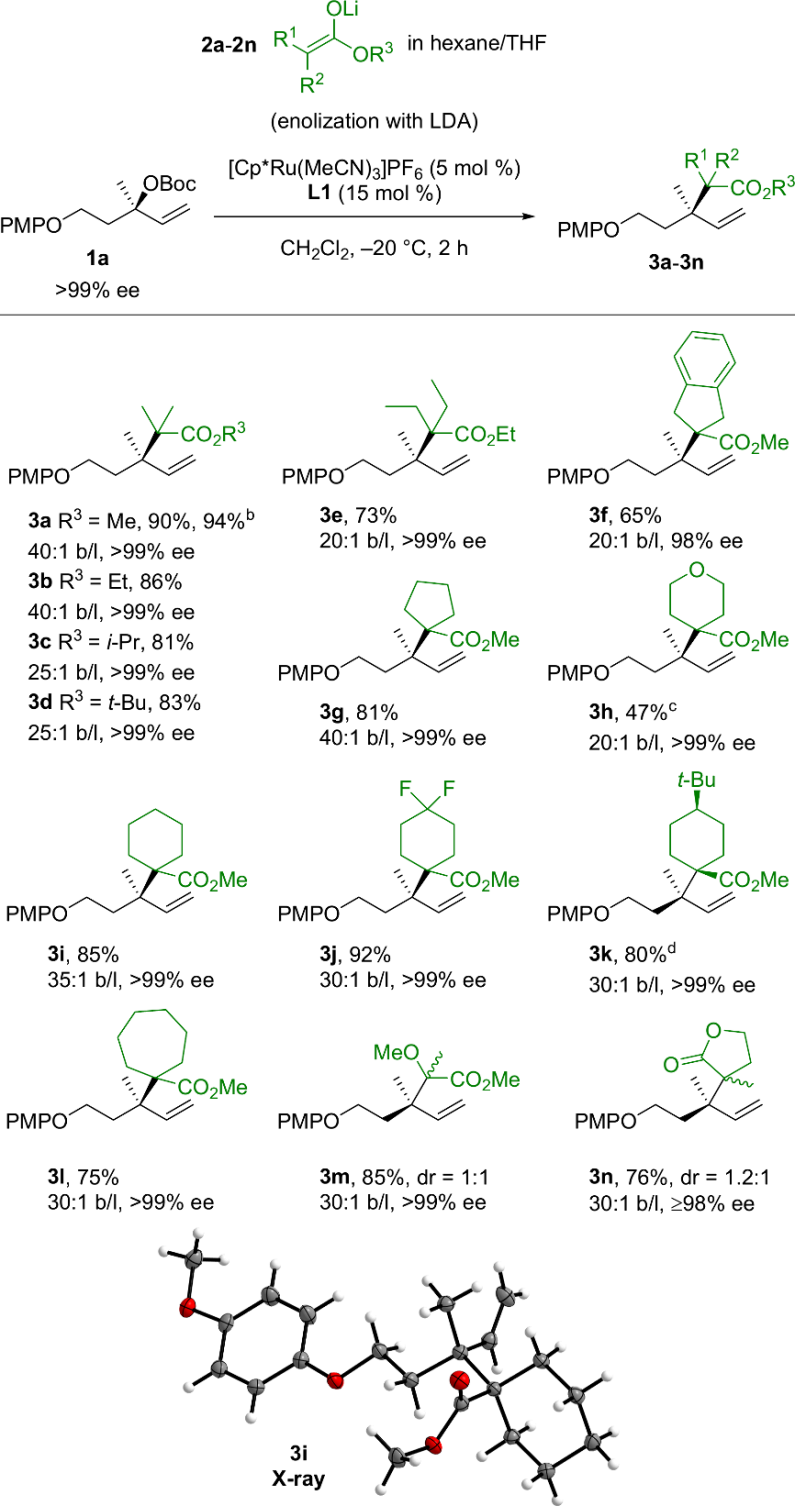
Scope of Lithium Ester Enolates for the Ru-Catalyzed Allylic Substitution Reaction Standard conditions were used. Isolated yields are provided. The reaction was run on gram scale (3.1 mmol). 0.45 M LDA was used instead. After 2 h at −20 °C, the reaction was warmed to 23 °C and stirred for another 10 h. **3k** was formed as a single diastereomer.

α-Methoxy propionate **3m** was obtained in a yield of 85% and 30:1 b/l (dr = 1:1).^28^ The method could be extended to 2-methyl butyrolactone to provide product **3n** in 76% yield and 30:1 b/l (dr = 1.2:1).^29^

We next examined the reaction scope with respect to the electrophile component (Scheme 4). Deprotection of the *para*-methoxyphenyl ether in **1a** and elaboration of the primary alcohol (see the Supporting Information) provided access to a broad set of substrates (**1b**-**1f** and **1k**, synthesized in >99% ee based on **1a**). Substrates incorporating potentially competing electrophiles, such as a tosylate, ester, and nitrile, could be utilized to give products (**4b**-**4d**) in high yields, ee and b/l.

**Scheme 4:**
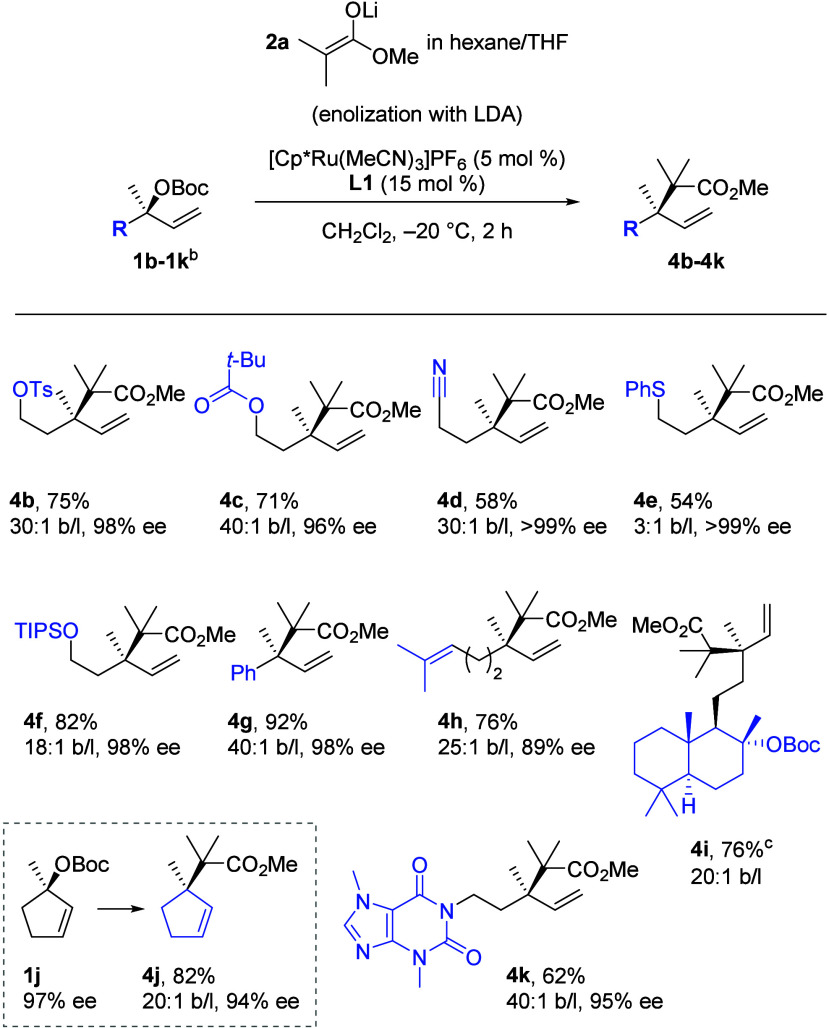
Scope of Enantioenriched Electrophiles for the Ru-Catalyzed Allylic Substitution Reaction Standard conditions were used. Isolated yields are provided. **1b**–**1f** and **1k** were accessed from **1a** in >99% ee. **1g** was accessed from (+)-1-phenylethanol in 98% ee. **1h** and **1j** were accessed from (−)-linalool in 97% ee. **4i** was formed as a single diastereomer.

Thioethers are known ligands for transition metal catalysts such as Ru which they often bind irreversibly.^30^ Accordingly, we were curious whether thioether **1e** would also be compatible with the catalyst. We subjected thioether **1e** to the reaction conditions. The corresponding coupling product **4e** was formed in 54% yield, 3:1 b/l, and excellent >99% ee. Frequently encountered bulky protecting groups such as the TIPS ether in **1f** were compatible with the method. Tertiary benzylic substrate **1g** was very well tolerated and provided **4g** in 92% yield, 40:1 b/l, and 98% ee. Commonly found terpenoid linalool-derived **1h** and diterpenoid sclareol-derived **1i** were successfully coupled with ester enolate **2a**. This afforded product **4h** in 76% yield and 89% ee as well as **4i** in 76% yield as a single diastereomer. We were able to extend the Ru-catalyzed allylic substitution to cyclic electrophiles. Tertiary carbonate **1j** provided cyclopentene **4j** featuring cyclic-acyclic vicinal quaternary centers. Heterocyclic and highly polar theobromine derivative **1k** was compatible and provided product in 62% yield and 40:1 b/l.

It is generally accepted^4h,27,31^ that Ru-mediated allylic substitution reactions proceed in analogy to the double inversion mechanism described for Pd^32^ and Ir.^33^ To the best of our knowledge, the stereochemical features of the oxidative addition of Ru^II^ to allylic electrophiles have not been studied. With that in mind, we set out to investigate the reaction of cyclic, diastereomeric carbonates **1l** and **1m**. Interestingly, the two diastereomers exhibited strikingly different reactivities (Scheme 5). Carbonate **1l** was unreactive under the standard conditions (−20 °C), however, conducting the reaction with **1l** at room temperature led to the formation of **4l** in 51% yield as a single diastereomer. Remarkably, **1m** did not react at all, even at 40 °C, and the starting material was fully recovered. This observation suggests that for **1m**, formation of an allyl complex is not possible under the reaction conditions. We hypothesize that stereoinvertive oxidative addition to the pseudoaxially oriented^34^ C–O bond proceeds for half-chair **1l** but is blocked by the adjacent isopropenyl group in half-chair **1m**. Hence, the differential reactivity of **1l** and **1m** further supports a double inversion mechanism to be operative, which accounts for the stereochemical net retention observed in Ru-catalyzed allylic substitution reactions.

**Scheme 5:**
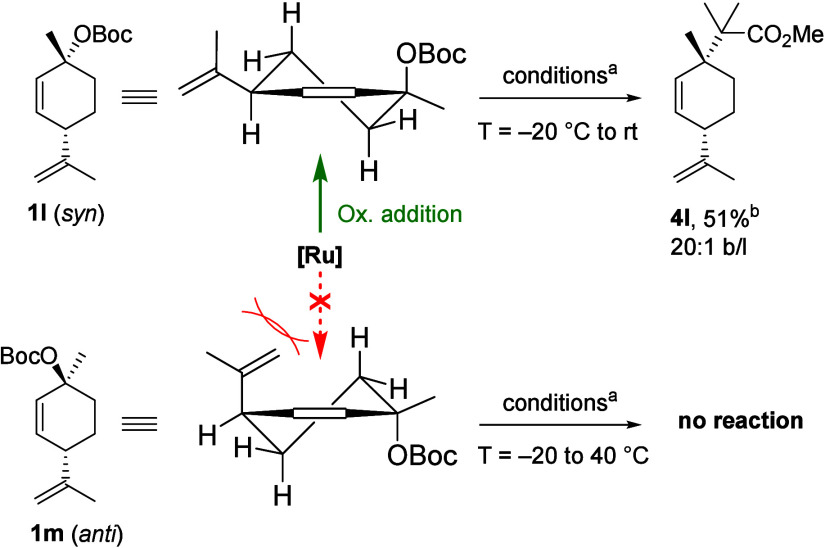
Allylic Substitution Reaction of Cyclic, Diastereomeric Electrophiles Allylic carbonate **1l**-**1m** (0.2 mmol), [Cp*Ru(MeCN)_3_]PF_6_ (5 mol %), phenoxythiazoline **L1** (15 mol %), lithium ester enolate **2a** (3 equiv, 0.9 M in 1.5:1 hexane–THF), CH_2_Cl_2_ (0.2 M), −20 °C. After 1 h at −20 °C, the reaction was warmed to 23 °C, additional 2 equiv of **2a** were added, and stirring was continued for 20 h. For **1m**, the reaction was further warmed to 40 °C and stirred for 3 h. Isolated yields are provided. **4l** was formed as a single diastereomer.

In conclusion, we have developed a novel catalyst based on the combination of [Cp*Ru(MeCN)_3_]PF_6_ and phenoxythiazoline ligand **L1** for the enantiospecific coupling of lithium ester enolates and tertiary allylic carbonates. The approach provides products featuring vicinal quaternary centers (acyclic-acyclic) in high yields, excellent ee (89–99%), and b/l ratios up to 40:1. Because the method is not limited to benzylic electrophiles as starting materials, a wide spectrum of highly congested products can be accessed. Finally, the results we describe provide fresh opportunities for the development of asymmetric allylation reactions that go beyond the traditional metals employed in the area to include ruthenium.

## Supporting Information Available

Experimental procedures and characterization data (PDF)
