# Supplementary material for: Construction of Vicinal Quaternary Centers via Ru-Catalyzed Enantiospecific Allylic Substitution with Lithium Ester Enolates
Source: J Am Chem Soc. 2024 Aug 19;146(34):23674–9. doi: 10.1021/jacs.4c07690 (PMC11363134; doi:10.1021/jacs.4c07690)
Supplement: Supplementary file 1 — ja4c07690_si_001.pdf [file ja4c07690_si_001.pdf]

Supporting Information

Construction of Vicinal Quaternary Centers via Ru-Catalyzed Enantiospecific Allylic Substitution with Lithium Ester Enolates

Sven M. Papidocha and Erick M. Carreira*

ETH Zürich, Vladimir-Prelog-Weg 3, HCI, 8093 Zürich, Switzerland

Table of Contents

1. General Remarks 2

2. Control Reactions 5

3. General Procedure and Characterization of Products 8

4. Preparation of Starting Materials 33

5. Preparation of Ligands 54

6. NMR Spectra 58

7. SFC Traces 111

8. HPLC Traces 135

9. X-Ray Crystallographic Data 137

10. References 141

1. General Remarks

Procedure

If not otherwise stated, all reactions have been conducted in flame-dried glassware and under a nitrogen atmosphere.

Chemicals

All chemicals and solvents used were supplied by the Department of Chemistry at ETH Zurich or bought directly from Sigma Aldrich, Acros Organics, TCI, Fluorochem and Apollo Scientific.

The precatalyst [Cp*Ru(MeCN)$_3$]PF$_6$ was bought from Sigma Aldrich (250 mg batch size) and used as received. It was stored in a freezer and a 250 mg batch was usually emptied within less than 1 month. The precatalyst was handled while being exposed to air and without the use of a glovebox.

The anhydrous THF and anhydrous CH_2Cl_2 used in the Ru-catalyzed allylic substitution were supplied by Acros Organics (extra dry over MS) and used as received. No further deoxygenation was applied.

Nuclear magnetic resonance spectroscopy

All NMR spectra were measured in deuterated solvents at room temperature with a Bruker Avance 400 (400 MHz, equipped with 9.4 T magnet and BBFO probe), Bruker Ascend 400 (400 MHz, equipped with 9.4 T magnet and BBFO probe), Bruker Ultrashield 400 (400 MHz, equipped with 9.4 T magnet and BBFO probe), an Oxford 400 (400 MHz, equipped with 9.4 T magnet and BBFO probe) or a Bruker Avance 500 (500 MHz, equipped with 11.7 T magnet and a BBFO probe). Chemical shifts in $CDCl_3$ are referenced to the solvent residual signal (^{1}H: δ = 7.26 ppm, ^{13}C: δ = 77.16 ppm). All chemical shifts are reported in parts per million (ppm). The following abbreviations are used in reporting NMR data: s = singlet, d = doublet, t = triplet, q = quartet, dd = doublet of doublets etc.

IR spectroscopy

Infrared spectra were recorded on a Perkin Elmer Two FT-IR spectrometer as thin films. Absorptions are given in wavenumbers (cm^{-1}).

High-resolution mass spectrometry

All mass spectra were measured by the ETH Zurich MS service on a Bruker Daltonics maXis ESI-QTOF or a Bruker Daltonics maXis II ESI-QTOF.

X-ray diffraction

The X-Ray diffraction was measured on a Rigaku Oxford Diffraction XtaLAB Synergy-S Dualflex kappa diffractometer equipped with a Dectris Pilatus 300 HPAD detector and using microfocus sealed tube Cu-Kα radiation with mirror optics (λ = 1.54178 Å). All measurements were carried out at 100K using an Oxford Cryosystems Cryostream 800 sample cryostat. Data collected on the Rigaku instrument were integrated using CrysAlisPro and corrected for absorption effects using a combination of empirical (ABSPACK) and numerical corrections. The structures were solved using SHELXT and refined by full-matrix least-squares analysis (SHELXL) using the program package OLEX2. All non-hydrogen atoms were refined anisotropically and hydrogen atoms were constrained to ideal geometries and refined with fixed isotropic displacement parameters (in terms of a riding model). The data was measured and analyzed by Dr. Nils Trapp and Michael Solar (all Small Molecule Crystallography Center, ETH Zurich).

Thin-layer chromatography

For reaction controls, TLC glass plates from Supelco® (TLC silica gel 60 F_{254}: 25 glass plates, 20 x 20 cm) were used. Spotted substances were made visible by exposure to ultraviolet light (254 nm or 365 nm) or TLC stain (CAM stain: Aqueous ceric ammonium molybdate solution).

Column chromatography

For column chromatography Sigma-Aldrich silica gel sorbent (high purity grade (9385), 230-400 mesh particle size, pore size 60) was used as a stationary phase.

Chiral separations

Supercritical fluid chromatography (SFC) was performed on a Jasco 2080 Plus system with a diode array detector (202 nm) under the conditions given for each measurement. Normal-phase (hexane/*i*-PrOH) high performance liquid chromatography (HPLC) was performed on a Dionex ultimate 3000 HPLC system (Thermo-Fischer) with a diode array detector under the conditions given for each measurement.

Specific rotation

Specific rotations (α) were measured on a Jasco P-2000 digital polarimeter at the sodium D line with a 10 cm cell length. Data are reported as follows: $[\alpha]^{T}$ in parentheses concentration (c = 1.00 corresponds to 10.0 $mg{\cdot}mL^{-1}$), and solvent. The temperature (T) at which the measurement was made is given as the superscript number (°C).

2. Control Reactions

The full set of control reactions is provided:

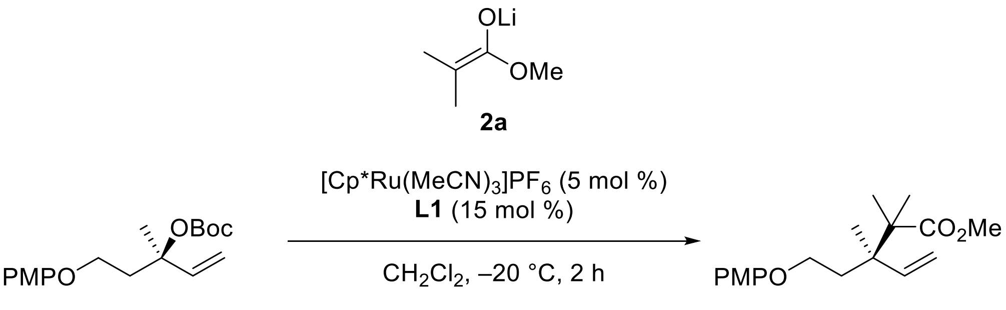

1a 3a

>99% ee PMP = *para*-methoxyphenyl

entry	deviation from standard conditions[a]	yield[b] (%)	b/l[c]	ee[d] (%)
1	no **L1**	9	1:2	29
2	**L0** instead of **L1**	32	7:1	55
3	none	92	40:1	>99
4	no [Ru]	0	-	-
5	[**X**Ru(MeCN)$_3$]PF$_6$ **X** = Cp instead of Cp*	11	1:1	79
6	**L2** instead of **L1**	81	30:1	>99
7	**L3** instead of **L1**	68	30:1	98
8	**L4** instead of **L1**	30	7:1	34
9	**L5** instead of **L1**	12	8:1	94
10	**L6** instead of **L1**	15	15:1	-
11	**L7** instead of **L1**	9	15:1	-
12	**L8** instead of **L1**	34	2:1	73
11	PhMe instead of CH_2Cl_2	80	8:1	>99
12	THF instead of CH_2Cl_2	67	5:1	97
13	rt instead of –20 °C	21	14:1	95
14	**1n** instead of **1a**	20	30:1	>99
15	ester enolization by LiHMDS instead of LDA	55	35:1	>99
16	ester enolization by NaHMDS instead of LDA	0	-	-
17	prestirring of [Cp*Ru(MeCN)$_3$]PF$_6$ and **L1** with 1 equiv K_2CO_3	89	40:1	>99
18	5 mol % [Cp*Ru(MeCN)$_3$]PF$_6$ and 5 mol % **L1** (1:1 ratio)	85	40:1	97
19	1 mol % [Cp*Ru(MeCN)$_3$]PF$_6$ and 3 mol % **L1**	62	40:1	>99
20	**2r** instead of **2a**	0	-	-
21	[(*p*-cymene)Ru(MeCN)$_3$](SbF$_6$)$_2$[1] instead of [Cp*Ru(MeCN)$_3$]PF$_6$, no **L1**	0	-	-
22	[Cp*RuCl$_2$]$_2$ instead of [Cp*Ru(MeCN)$_3$]PF$_6$, no **L1**	0	-	-
23	[C$_6$H$_6$RuCl$_2$]$_2$ instead of [Cp*Ru(MeCN)$_3$]PF$_6$, no **L1**	0	-	-

[a]Standard conditions: Allylic carbonate **1a** (0.2 mmol), $[Cp^*Ru(MeCN)_3]PF_6$ (5 mol %), phenoxythiazoline **L1** (15 mol %), lithium ester enolate **2a** (3 equiv, 0.9 M in 1.5:1 hexane–THF), CH_2Cl_2 (0.17 M), –20 °C, 2 h. [b]Yield was determined by analysis of the ^{1}H NMR spectra of the unpurified reaction mixture using phenanthrene as an internal standard. [c]b/l refers to ratios of branched/linear isomers determined by analysis of the ^{1}H NMR spectra of the unpurified reaction mixtures. [d]Determined by SFC analysis using a chiral stationary phase.

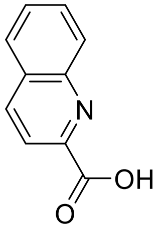

L0

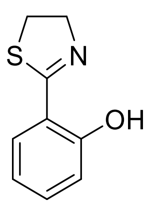

L1

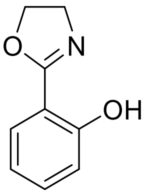

L2

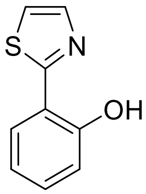

L3

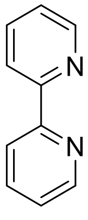

L4

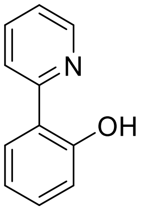

L5

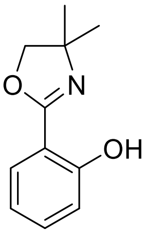

L6

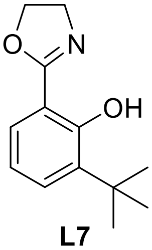

L7

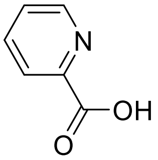

L8

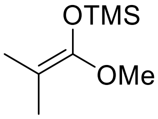

2r

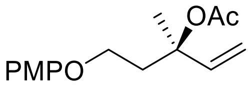

1n

Unsuccessful esters (enolate form):

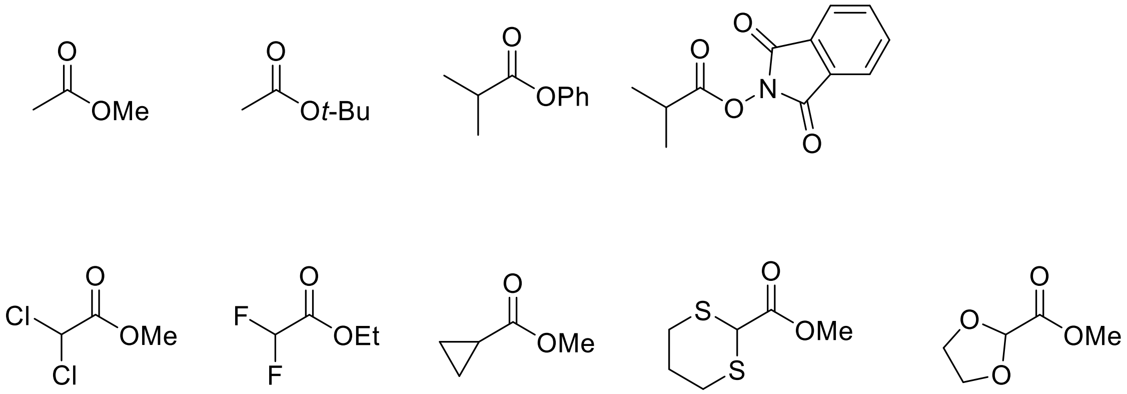

Other unsuccessful nucleophiles (deprotonated form):

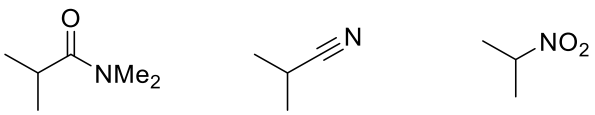

Investigation of the diastereoselectivity for the allylic substitution with enolate 2m:

Two geometrical isomers can be formed in the enolization process of **2m** and we wanted to clarify whether the lack of diastereocontrol derives from unselective enolization or unselective C-C coupling with electrophile **1a**. Hence, we generated enolate **2m** under the conditions described and trapped it at –78 °C with TMSCl, whereby we observe that the Z-isomer is selectively formed (Z/E = 15:1):

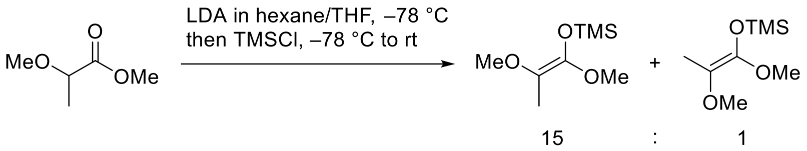

The lack of diastereoselectivity for the allylic substitution with **2m** suggests that the C-C coupling process does not display facial selectivity with respect to the enolate. In attempts to improve the diastereoselectivity of the allylic substitution with enolate **2m**, we investigated ligands **L0**, **L4**, **L6**, and **L7** instead of standard ligand **L1**:

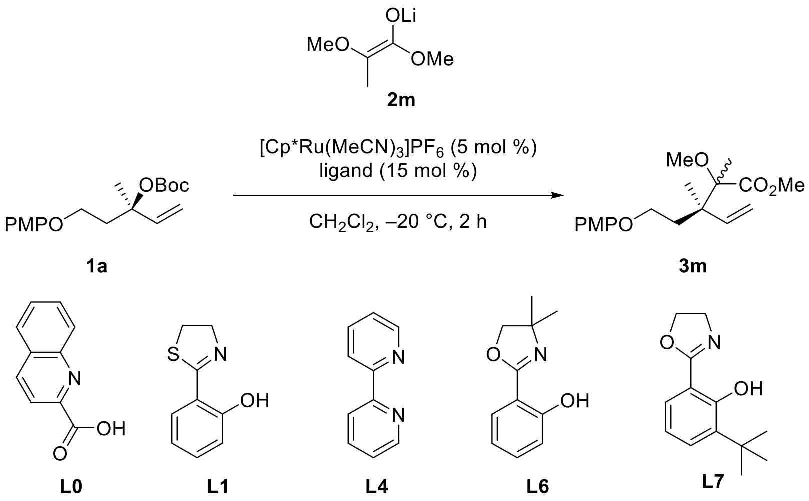

ligand	**L0**	**L1**	**L4**	**L6**	**L7**
yield[a] of **3m** (%)	46	85	46	11	10
dr[a]	1:1	1:1	1:1	1:1	1:1

[a]Yield and dr were determined by analysis of the ^{1}H NMR spectra of the unpurified reaction mixture using phenanthrene as an internal standard.

As observed for enolate **2a**, the choice of ligand greatly influenced the reaction yield. However, allylic substitution using the shown ligands above provided product **3m** in dr = 1:1. Hence, we did not observe a ligand effect on the diastereoselectivity of the reaction.

3. General Procedure and Characterization of Products

Preparation of 0.9 M LDA solution

Diisopropylamine (0.84 mL, 6.0 mmol, 1.1 equiv) was dissolved in THF (2.3 mL) inside a 20 mL Schlenk flask under N_2 atmosphere and the solution was cooled to 0 °C. 1.6 M *n*-BuLi in hexane (3.5 mL, 5.6 mmol, 1.0 equiv) was added dropwise and the solution was stirred for 30 min at 0 °C after which it was warmed to rt and used in the next step.

Preparation of lithium ester enolates

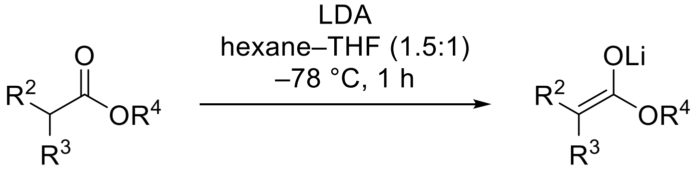

2a'-2n' **2a-2n**

Inside a 10 mL Schlenk flask under N_2 atmosphere, 0.9 M LDA solution (1.9 mL, 1.7 mmol, 1.0 equiv) was provided and cooled to –78 °C. Ester **2a'-2n'** (1.8 mmol, 1.1 equiv) was added drop-wise to the milky suspension and the thereby obtained, clear solution was stirred for 1 h at –78 °C after which it was used in the next step.

General procedure for the Ru-catalyzed allylic substitution (0.2 mmol scale)

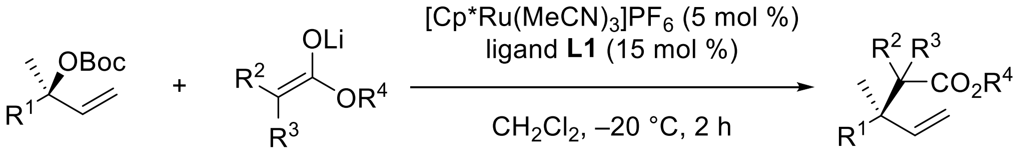

1a-1m **3a-3n**
4b-4l

$[Cp^*Ru(MeCN)_3]PF_6$ (5 mg, 0.01 mmol, 5 mol %) and **L1** (5.4 mg, 0.030 mmol, 15 mol %) were added to a 5 mL crimp vial equipped with a magnetic stir bar. The crimp vial was closed, evacu-ated, and backfilled with nitrogen three times. CH_2Cl_2 (0.4 mL) was added, and the dark greenish solution was stirred for one minute at rt, then cooled to –20 °C with a cryostat. Allylic carbonate

1a-1m (0.20 mmol, 1.0 equiv) was dissolved in CH_2Cl_2 (0.4 mL) inside a second 5 mL flask under nitrogen and this solution was added to the [Cp*Ru(MeCN)$_3$]PF_6 /**L1** mixture at –20 °C. Additional CH_2Cl_2 (0.4 mL) was used to wash the flask containing allylic carbonate **1a-1m**. The mixture was stirred 5 min at –20 °C, after which a solution (0.6 mL, 0.6 mmol, 3 equiv) of the respective ester enolate in hexane–THF (1.5:1) was quickly added by syringe (see Note 1 and 2). The orange/maroon mixture was stirred for 2 h at –20 °C. The reaction was poured onto a mixture of sat. aq. NH_4Cl (3 mL) and EtOAc (3 mL) containing phenanthrene as an internal standard. The phases were separated, and the aqueous phase was extracted once with EtOAc (2 mL). The combined organic extracts were dried over $MgSO_4$ and concentrated under reduced pressure. Purification by column chromatography afforded the pure products **3a**-**3n** and **4b**-**4l**.

Note 1: The ester enolate solution at –78 °C can alternatively be added by cannulation to minimize warming during transfer. However, we did not observe differences in isolated product yields between quick addition by syringe (<20 s) or cannulation.

Note 2: The addition of strong bases to CH_2Cl_2 can generate chlorocarbene[2] which poses an explosion hazard. However, in all our experiments we never observed violent or explosive reaction behavior employing the lithium ester enolates, which are generated in hexane/THF before addition to the reaction in CH_2Cl_2.

Procedure for the Ru-catalyzed allylic substitution on gram scale (3.1 mmol)

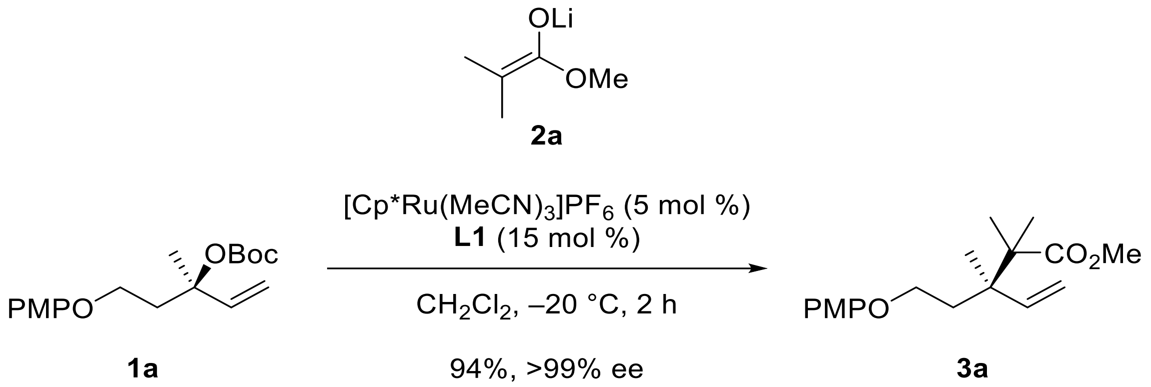

[Cp*Ru(MeCN)$_3$]PF$_6$ (78 mg, 0.16 mmol, 5.0 mol %) and **L1** (83 mg, 0.47 mmol, 15 mol %) were added to a 100 mL Schlenk flask equipped with a magnetic stir bar. The Schlenk flask was evacuated and backfilled with nitrogen three times. CH_2Cl_2 (6 mL) was added, and the dark greenish solution was stirred for one minute at rt, then cooled to –20 °C with a cryostat. Allylic carbonate **1a** (1.0 g, 3.1 mmol, 1.0 equiv) was dissolved in CH_2Cl_2 (6 mL) inside a second 25 mL flask under nitrogen and this solution was added to the [Cp*Ru(MeCN)$_3$]PF$_6$ /**L1** mixture at –20 °C. Additional CH_2Cl_2 (6 mL) was used to wash the flask containing allylic carbonate **1a**. The mixture was stirred 5 min at –20 °C, after which a solution (9.3 mL, 9.3 mmol, 3 equiv) of ester enolate **2a** in hexane–THF (1.5:1) was added by cannulation. The orange/maroon mixture was stirred for 2 h at –20 °C. The reaction was poured onto a mixture of sat. aq. NH_4Cl (30 mL) and EtOAc (30 mL) containing phenanthrene as an internal standard. The phases were separated, and the aqueous phase was extracted once with EtOAc (20 mL). The combined organic extracts were dried over $MgSO_4$ and concentrated under reduced pressure. Purification by column chromatography afforded the product **3a** as a colorless oil (890 mg, 2.9 mmol, 94%, >99% ee).

Characterization of products

Note: The absolute stereochemistry in products **3a-3h**, **3k-3n** and **4b-4k** was assigned in analogy to products **3i**, **3j** and **4l** (retention of configuration).

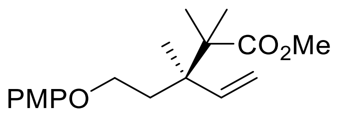

3a

Methyl (R)-3-(2-(4-methoxyphenoxy)ethyl)-2,2,3-trimethylpent-4-enoate (3a)

Yield: 57 mg, 0.18 mmol, 92% (40:1 b/l); **TLC:** R_f = 0.25 (hexane/EtOAc 20:1, CAM stain, UV); **^{1}H NMR** (400 MHz, $CDCl_3$): δ 6.83 – 6.75 (m, 4H), 5.86 (dd, *J* = 17.5, 10.9 Hz, 1H), 5.16 (dd, *J* = 10. 9, 1.3 Hz, 1H), 5.00 (dd, *J* = 17.5, 1.3 Hz, 1H), 3.81 (ddd, *J* = 8.6, 6.7, 2.2 Hz, 2H), 3.76 (s, 3H), 3.65 (s, 3H), 1.97 (ddd, *J* = 7.6, 6.6, 2.9 Hz, 2H), 1.17 (s, 3H), 1.17 (s, 3H), 1.09 (s, 3H); **^{13}C NMR** (101 MHz, $CDCl_3$): δ 177.1, 153.9, 153.2, 142.2, 115.5, 114.9, 114.7, 66.2, 55.9, 51.6, 48.9, 43.5, 34.6, 21.6, 21.5, 17.5; **IR:** 2950, 1724, 1508, 1466, 1230, 1136, 1109, 1040, 918, 825 cm^{-1}; **HRMS(ESI):** Exact mass calculated for $C_{18}H_{27}O_4$ [$(M+H)^+$], 307.1904; found 307.1905; **SFC:** Daicel Chiralcel OJ-H, 10% MeOH, 2.0 mL/min, 25 °C, >99% ee (TR (1, major enantiomer) = 5.6 min, TR (2, minor enantiomer) = 7.1 min); **$[\alpha]_D^{25}$** = +20.1 (c = 1.00, $CHCl_3$).

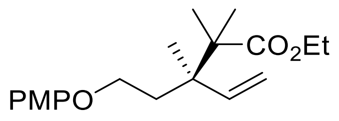

3b

Ethyl (R)-3-(2-(4-methoxyphenoxy)ethyl)-2,2,3-trimethylpent-4-enoate (3b)

Yield: 55 mg, 0.17 mmol, 86% (40:1 b/l); **TLC:** R_f = 0.25 (hexane/EtOAc 20:1, CAM stain, UV); **^{1}H NMR** (400 MHz, $CDCl_3$): δ 6.87 – 6.74 (m, 4H), 5.88 (dd, *J* = 17.5, 10.9 Hz, 1H), 5.16 (dd, *J* = 10.9, 1.3 Hz, 1H), 5.00 (dd, *J* = 17.5, 1.3 Hz, 1H), 4.12 (q, *J* = 7.1 Hz, 2H), 3.81 (ddd, *J* = 8.5, 6.6, 1.9 Hz, 2H), 3.76 (s, 3H), 1.98 (ddd, *J* = 7.6, 6.5, 3.9 Hz, 2H), 1.26 (t, *J* = 7.1 Hz, 3H), 1.16 (s, 3H), 1.16 (s, 3H), 1.10 (s, 3H); **^{13}C NMR** (101 MHz, $CDCl_3$): δ 176.6, 153.8, 153.2, 142.3,

115.6, 114.8, 114.7, 66.2, 60.4, 55.9, 48.6, 43.5, 34.5, 21.6, 21.5, 17.5, 14.4; **IR:** 2979, 1718, 1508, 1466, 1230, 1178, 1135, 1108, 1039, 825 cm^{-1}; **HRMS(ESI):** Exact mass calculated for $C_{19}H_{29}O_4$ [$(M+H)^+$], 321.2060; found 321.2057; **SFC:** Daicel Chiralcel OJ-H, 5% MeOH, 2.0 mL/min, 25 °C, >99% ee (T_R (1, major enantiomer) = 6.2 min, T_R (2, minor enantiomer) = 7.8 min); **$[\alpha]_D^{25}$** = +15.4 (c = 1.00, $CHCl_3$).

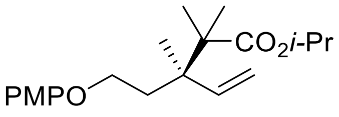

3c

Isopropyl (R)-3-(2-(4-methoxyphenoxy)ethyl)-2,2,3-trimethylpent-4-enoate (3c)

Yield: 54 mg, 0.16 mmol, 81% (25:1 b/l); **TLC:** R_f = 0.27 (hexane/EtOAc 20:1, CAM stain, UV); **^{1}H NMR** (400 MHz, $CDCl_3$): δ 6.85 – 6.74 (m, 4H), 5.88 (dd, *J* = 17.5, 10.9 Hz, 1H), 5.15 (dd, *J* = 10.9, 1.3 Hz, 1H), 5.05 – 4.95 (m, 2H), 3.82 (ddd, *J* = 8.3, 6.6, 1.6 Hz, 2H), 3.76 (s, 3H), 2.07 – 1.92 (m, 2H), 1.24 (d, *J* = 2.2 Hz, 3H), 1.22 (d, *J* = 2.2 Hz, 3H), 1.15 (s, 3H), 1.14 (s, 3H), 1.10 (s, 3H); **^{13}C NMR** (101 MHz, $CDCl_3$): δ 176.0, 153.8, 153.2, 142.3, 115.6, 114.8, 114.7, 67.9, 66.2, 55.9, 48.5, 43.4, 34.5, 22.0, 22.0, 21.6, 21.6, 17.5; **IR:** 2979, 1715, 1508, 1467, 1230, 1179, 1102, 1040, 916, 824 cm^{-1}; **HRMS(ESI):** Exact mass calculated for $C_{20}H_{31}O_4$ [$(M+H)^+$], 335.2217; found 335.2214; **SFC:** Daicel Chiralcel OJ-H, 5% MeOH, 2.0 mL/min, 25 °C, >99% ee (T_R (1, major enantiomer) = 4.3 min, T_R (2, minor enantiomer) = 4.8 min); **$[\alpha]_D^{25}$** = +17.4 (c = 1.00, $CHCl_3$).

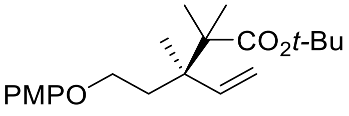

3d

tert-Butyl (R)-3-(2-(4-methoxyphenoxy)ethyl)-2,2,3-trimethylpent-4-enoate (3d)

Yield: 58 mg, 0.17 mmol, 83% (25:1 b/l); **TLC:** R_f = 0.30 (hexane/EtOAc 20:1, CAM stain, UV); **^{1}H NMR** (400 MHz, $CDCl_3$): δ 6.84 – 6.74 (m, 4H), 5.89 (dd, *J* = 17.5, 10.9 Hz, 1H), 5.15 (dd, *J*

= 10.9, 1.4 Hz, 1H), 4.99 (dd, J = 17.5, 1.4 Hz, 1H), 3.82 (ddd, J = 8.2, 6.6, 1.3 Hz, 2H), 3.76 (s, 3H), 2.08 – 1.92 (m, 2H), 1.44 (s, 9H), 1.12 (s, 3H), 1.11 (s, 3H), 1.10 (s, 3H); **^{13}C NMR** (101 MHz, $CDCl_3$): δ 175.8, 153.8, 153.2, 142.5, 115.6, 114.7, 114.6, 80.5, 66.2, 55.9, 48.9, 43.4, 34.4, 28.2, 21.8, 21.7, 17.6; **IR:** 2976, 1716, 1508, 1467, 1367, 12390, 1137, 1109, 1041, 825 cm^{-1}; **HRMS(ESI):** Exact mass calculated for $C_{21}H_{33}O_4$ [(M+H)$^+$], 349.2373; found 349.2375; **SFC:** Daicel Chiralcel OJ-H, 1% MeOH, 2.0 mL/min, 25 °C, >99% ee (T_R (1, major enantiomer) = 8.5 min, T_R (2, minor enantiomer) = 10.3 min); **[α]$_D$^{25}** = +16.1 (c = 1.00, $CHCl_3$).

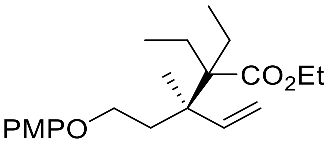

3e

Ethyl (R)-2,2-diethyl-3-(2-(4-methoxyphenoxy)ethyl)-3-methylpent-4-enoate (3e)

Yield: 51 mg, 0.15 mmol, 73% (20:1 b/l); **TLC:** R_f = 0.27 (hexane/EtOAc 20:1, CAM stain, UV); **^{1}H NMR** (400 MHz, $CDCl_3$): δ 6.83 – 6.74 (m, 4H), 5.93 (dd, J = 17.5, 10.9 Hz, 1H), 5.11 (dd, J = 10.9, 1.3 Hz, 1H), 4.97 (dd, J = 17.5, 1.4 Hz, 1H), 4.13 (qd, J = 7.1, 2.2 Hz, 2H), 3.82 – 3.77 (m, 2H), 3.75 (s, 3H), 2.06 (ddd, J = 13.4, 8.7, 6.9 Hz, 1H), 1.99 – 1.90 (m, 1H), 1.89 – 1.66 (m, 4H), 1.26 (t, J = 7.1 Hz, 3H), 1.12 – 1.10 (m, 3H), 0.90 (dt, J = 10.1, 7.4 Hz, 6H); **^{13}C NMR** (101 MHz, $CDCl_3$): δ 175.4, 153.8, 153.2, 143.5, 115.6, 114.7, 114.2, 66.1, 60.1, 55.9, 55.8, 44.7, 35.3, 23.6, 23.2, 18.6, 14.4, 10.6, 10.5; **IR:** 2978, 1717, 1508, 1465, 1227, 1135, 1106, 1039, 916, 824 cm^{-1}; **HRMS(ESI):** Exact mass calculated for $C_{21}H_{32}NaO_4$ [(M+Na)$^+$], 371.2193; found 371.2195; **SFC:** Daicel Chiralcel OJ-H, 5% MeOH, 2.0 mL/min, 25 °C, >99% ee (T_R (1, major enantiomer) = 6.1 min, T_R (2, minor enantiomer) = 7.4 min); **[α]$_D$^{25}** = +22.6 (c = 1.00, $CHCl_3$).

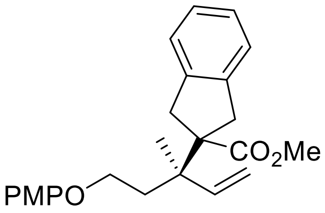

3f

Methyl (R)-2-(5-(4-methoxyphenoxy)-3-methylpent-1-en-3-yl)-2,3-dihydro-1H-indene-2-carboxylate (3f)

Yield: 50 mg, 0.13 mmol, 65% (20:1 b/l); **TLC:** R_f = 0.35 (hexane/EtOAc 15:1, CAM stain, UV); **^{1}H NMR** (400 MHz, $CDCl_3$): δ 7.17 – 7.08 (m, 4H), 6.85 – 6.76 (m, 4H), 5.92 (dd, *J* = 17.4, 10.9 Hz, 1H), 5.20 (dd, *J* = 10.9, 1.2 Hz, 1H), 5.08 (dd, *J* = 17.4, 1.2 Hz, 1H), 3.86 (dd, *J* = 8.1, 6.6 Hz, 2H), 3.77 (s, 3H), 3.64 (s, 3H), 3.52 (dd, *J* = 16.3, 14.4 Hz, 2H), 3.17 (dd, *J* = 16.3, 12.7 Hz, 2H), 2.21 – 1.94 (m, 2H), 1.15 (s, 3H); **^{13}C NMR** (101 MHz, $CDCl_3$): δ 176.3, 153.9, 153.2, 142.4, 141.7, 141.4, 126.6, 126.5, 124.2, 124.2, 115.6, 115.4, 114.8, 65.9, 63.4, 55.9, 52.1, 43.5, 38.2, 38.2, 36.0, 19.2; **IR:** 2950, 1721, 1507, 1461, 1229, 1173, 1039, 921, 824, 746 cm^{-1}; **HRMS(ESI):** Exact mass calculated for $C_{24}H_{29}O_4$ [$(M+H)^+$], 381.2060; found 381.2060; **SFC:** Daicel Chiralpak IB, 5% MeOH, 2.0 mL/min, 25 °C, 98% ee (T_R (1, minor enantiomer) = 13.2 min, T_R (2, major enantiomer) = 14.4 min) **$[\alpha]_D^{25}$** = –1.3 (c = 1.00, $CHCl_3$).

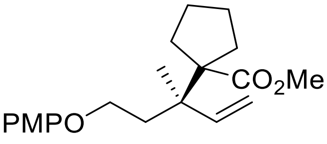

3g

Methyl (R)-1-(5-(4-methoxyphenoxy)-3-methylpent-1-en-3-yl)cyclopentane-1-carboxylate (3g)

Yield: 54 mg, 0.16 mmol, 81% (40:1 b/l); **TLC:** R_f = 0.25 (hexane/EtOAc 20:1, CAM stain, UV); **^{1}H NMR** (400 MHz, $CDCl_3$): δ 6.87 – 6.74 (m, 4H), 5.88 (dd, *J* = 17.5, 10.9 Hz, 1H), 5.15 (dd, *J* = 10.9, 1.3 Hz, 1H), 5.00 (dd, *J* = 17.5, 1.3 Hz, 1H), 3.81 (dd, *J* = 7.9, 7.0 Hz, 2H), 3.76 (s, 3H), 3.67 (s, 3H), 2.26 – 2.17 (m, 2H), 1.97 (td, *J* = 7.2, 1.5 Hz, 2H), 1.69 – 1.41 (m, 6H), 1.10 (s, 3H);

^{13}C NMR (101 MHz, $CDCl_3$): δ 177.0, 153.8, 153.2, 142.6, 115.5, 114.8, 114.7, 66.0, 63.1, 55.9, 51.8, 43.1, 36.0, 31.3, 31.2, 24.6, 19.4; **IR:** 2950, 1720, 1507, 1414, 1229, 1162, 1040, 918, 824, 757 cm^{-1}; **HRMS(ESI):** Exact mass calculated for $C_{20}H_{28}NaO_4$ $[(M+Na)^+]$, 355.1880; found 355.1878; **SFC:** Daicel Chiralcel OJ-H, 5% MeOH, 2.0 mL/min, 25 °C, >99% ee (T_R (1, major enantiomer) = 12.6 min, T_R (2, minor enantiomer) = 14.9 min); **$[\alpha]_D^{25}$** = +14.9 (c = 1.00, $CHCl_3$).

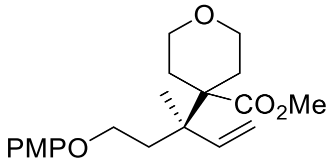

3h

Methyl (R)-4-(5-(4-methoxyphenoxy)-3-methylpent-1-en-3-yl)tetrahydro-2H-pyran-4-carboxylate (3h)

Note: Due to limited solubility of the ester enolate **2h** at –78 °C, a 0.45 M LDA solution was used instead for the enolization. After enolate addition and stirring for 2 h at –20 °C, the reaction was warmed to 23 °C and stirred for another 10 h. Work-up and purification occurred as described in the general procedure.

Yield: 33 mg, 0.095 mmol, 47% (20:1 b/l); **TLC:** R_f = 0.23 (hexane/EtOAc 4:1, CAM stain, UV); **^{1}H NMR** (500 MHz, $CDCl_3$): δ 6.83 – 6.75 (m, 4H), 5.81 (dd, *J* = 17.5, 10.9 Hz, 1H), 5.21 (dd, *J* = 10.9, 1.1 Hz, 1H), 5.01 (dd, *J* = 17.5, 1.2 Hz, 1H), 3.94 – 3.86 (m, 1H), 3.81 (ddd, *J* = 8.3, 6.7, 3.3 Hz, 1H), 3.76 (s, 3H), 3.74 (s, 3H), 3.29 (dtd, *J* = 26.4, 12.2, 1.9 Hz, 2H), 2.03 (td, *J* = 13.6, 2.5 Hz, 2H), 1.92 (td, *J* = 7.2, 1.4 Hz, 2H), 1.69 (dddd, *J* = 13.4, 12.3, 8.9, 4.7 Hz, 2H), 1.06 (s, 3H); **^{13}C NMR** (126 MHz, $CDCl_3$): δ 174.5, 153.9, 153.1, 141.6, 115.7, 115.6, 114.8, 66.4, 66.4, 65.9, 55.9, 52.7, 51.6, 43.7, 34.3, 29.5, 29.4, 17.5; **IR:** 2953, 2856, 1722, 1508, 1443, 1508, 1231, 1107, 1039, 826 cm^{-1}; **HRMS(ESI):** Exact mass calculated for $C_{20}H_{28}NaO_5$ $[(M+Na)^+]$, 371.1829; found 371.1822; **SFC:** Daicel Chiralcel OJ-H, 2% MeOH, 2.0 mL/min, 25 °C, >99% ee (T_R (1, major enantiomer) = 33.0 min, T_R (2, minor enantiomer) = 37.4 min); **$[\alpha]_D^{25}$** = +11.6 (c = 1.00, $CHCl_3$).

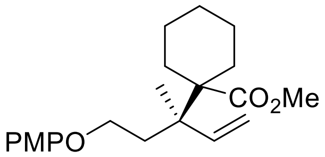

3i

Methyl (R)-1-(5-(4-methoxyphenoxy)-3-methylpent-1-en-3-yl)cyclohexane-1-carboxylate (3i)

Yield: 59 mg, 0.17 mmol, 85% (35:1 b/l); **TLC:** R_f = 0.25 (hexane/EtOAc 20:1, CAM stain, UV); **^{1}H NMR** (400 MHz, $CDCl_3$): δ 6.87 – 6.73 (m, 4H), 5.83 (dd, *J* = 17.5, 10.9 Hz, 1H), 5.15 (dd, *J* = 10.9, 1.3 Hz, 1H), 4.96 (dd, *J* = 17.5, 1.3 Hz, 1H), 3.79 (ddd, *J* = 8.5, 6.7, 3.1 Hz, 2H), 3.75 (s, 3H), 3.69 (s, 3H), 2.22 – 2.10 (m, 2H), 1.94 (td, *J* = 7.4, 1.9 Hz, 2H), 1.69 – 1.62 (m, 2H), 1.61 – 1.54 (m, 1H), 1.31 – 1.20 (m, 3H), 1.16 (dt, *J* = 12.8, 2.8 Hz, 1H), 1.12 – 1.00 (m, 4H); **^{13}C NMR** (101 MHz, $CDCl_3$): δ 175.3, 153.8, 153.2, 142.5, 115.5, 114.8, 114.7, 66.1, 55.9, 54.6, 51.2, 44.0, 34.5, 29.3, 29.2, 25.8, 24.4, 24.2, 17.7; **IR:** 2937, 1720, 1508, 1229, 1208, 1119, 1040, 915, 824, 757 cm^{-1}; **HRMS(ESI):** Exact mass calculated for $C_{21}H_{30}NaO_4$ $[(M+Na)^+]$, 369.2036; found 369.2042; **SFC:** Daicel Chiralcel OJ-H, 5% MeOH, 2.0 mL/min, 25 °C, >99% ee (T_R (1, major enantiomer) = 12.8 min, T_R (2, minor enantiomer) = 15.5 min); **$[\alpha]_D^{25}$** = +13.1 (c = 1.00, $CHCl_3$).

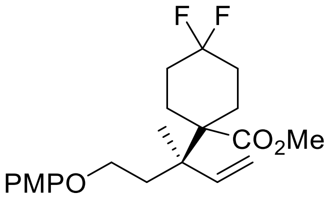

3j

Methyl (R)-4,4-difluoro-1-(5-(4-methoxyphenoxy)-3-methylpent-1-en-3-yl)cyclohexane-1-carboxylate (3j)

Yield: 70 mg, 0.18 mmol, 92% (30:1 b/l); **TLC:** R_f = 0.31 (hexane/EtOAc 15:1, CAM stain, UV); **^{1}H NMR** (400 MHz, $CDCl_3$): δ 6.85 – 6.73 (m, 4H), 5.81 (dd, *J* = 17.5, 10.9 Hz, 1H), 5.21 (dd, *J* = 10.9, 1.1 Hz, 1H), 5.02 (dd, *J* = 17.5, 1.1 Hz, 1H), 3.83 – 3.77 (m, 2H), 3.75 (s, 3H), 3.73 (s, 3H), 2.30 – 2.19 (m, 2H), 2.03 (dtt, *J* = 10.8, 6.2, 1.7 Hz, 1H), 1.95 (td, *J* = 7.8, 7.3, 6.3 Hz, 1H),

1.81 – 1.57 (m, 4H), 1.09 (s, 3H); **^{13}C NMR** (101 MHz, $CDCl_3$): δ 174.1, 153.9, 153.1, 141.5, 123.1 (dd, *J* = 238.7, 238.6 Hz), 65.8, 55.8, 53.4, 53.3, 51.8, 43.7, 43.7, 34.8, 31.9 (ddd, *J* = 25.3, 22.9, 8.5 Hz), 25.7 (dd, *J* = 11.2, 9.9 Hz), 17.9; **^{19}F NMR** (376 MHz, $CDCl_3$) δ -91.96 (d, *J* = 234.5 Hz), -103.67 (d, *J* = 234.4 Hz); **IR:** 2951, 1721, 1508, 1444, 1378, 1229, 1210, 1107, 1002, 825 cm^{-1}; **HRMS(ESI):** Exact mass calculated for $C_{21}H_{29}F_2O_4$ [(M+H)$^+$], 383.2028; found 383.2027; **SFC:** Daicel Chiralpak AS-H, 1% MeOH, 1.5 mL/min, 25 °C, >99% ee (T_R (1, major enantiomer) = 10.2 min, T_R (2, minor enantiomer) = 12.1 min); **$[\alpha]_D^{25}$** = +11.4 (c = 1.00, $CHCl_3$).

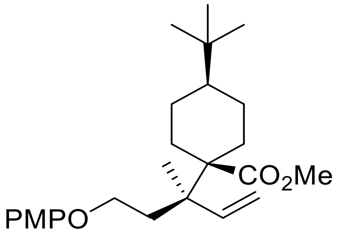

3k

Methyl (1s,4S)-4-(tert-butyl)-1-((R)-5-(4-methoxyphenoxy)-3-methylpent-1-en-3-yl)cyclohexane-1-carboxylate (3k)

The relative stereochemistry of **3k** could not be determined based on NOE measurements. However, it has been shown that alkylation of the exocyclic double bond of conformationally fixed cyclohexanone enolates selectively affords the equatorial products.[3] Therefore, we propose the shown diastereomer **3k** is formed.

Yield: 65 mg, 0.16 mmol, 80% (30:1 b/l); **TLC:** R_f = 0.32 (hexane/EtOAc 40:1, CAM stain, UV); **^{1}H NMR** (400 MHz, $CDCl_3$): δ 6.86 – 6.72 (m, 4H), 5.83 (dd, *J* = 17.5, 10.9 Hz, 1H), 5.15 (dd, *J* = 10.9, 1.3 Hz, 1H), 4.96 (dd, *J* = 17.5, 1.3 Hz, 1H), 3.79 (ddd, *J* = 8.5, 6.9, 2.9 Hz, 1H), 3.75 (s, 3H), 3.68 (s, 3H), 2.23 (ddd, *J* = 16.7, 13.2, 3.1 Hz, 2H), 1.94 (dd, *J* = 8.4, 6.8 Hz, 2H), 1.73 – 1.64 (m, 2H), 1.28 (tt, *J* = 12.5, 3.0 Hz, 2H), 1.05 (s, 3H), 1.03 – 0.83 (m, 3H), 0.79 (s, 9H); **^{13}C NMR** (101 MHz, $CDCl_3$): δ 175.3, 153.8, 153.2, 142.5, 115.5, 114.7, 114.7, 66.1, 55.8, 54.3, 51.2, 47.5, 43.7, 34.6, 32.4, 29.5, 29.5, 27.6, 25.2, 25.1, 17.8; **IR:** 2947, 1723, 1508, 1450, 1230, 1145, 1109, 1041, 916, 824 cm^{-1}; **HRMS(ESI):** Exact mass calculated for $C_{25}H_{39}HO_4$ [(M+Na)$^+$], 403.2843; found 403.2838; **SFC:** Daicel Chiralpak IA, 5% MeOH, 2.0 mL/min, 25 °C, >99% ee

(T_R (1, major enantiomer) = 9.5 min, T_R (2, minor enantiomer) = 11.2 min); **[α]$_D^{25}$** = +6.7 (c = 1.00, $CHCl_3$).

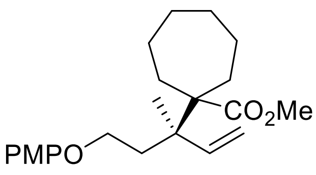

3l

Methyl (R)-1-(5-(4-methoxyphenoxy)-3-methylpent-1-en-3-yl)cycloheptane-1-carboxylate (3l)

Yield: 54 mg, 0.15 mmol, 75% (30:1 b/l); **TLC:** R_f = 0.25 (hexane/EtOAc 20:1, CAM stain, UV); **^{1}H NMR** (400 MHz, $CDCl_3$): δ 6.85 – 6.73 (m, 4H), 5.83 (dd, *J* = 17.5, 10.9 Hz, 1H), 5.14 (dd, *J* = 10.9, 1.3 Hz, 1H), 4.97 (dd, *J* = 17.5, 1.3 Hz, 1H), 3.80 – 3.76 (m, 2H), 3.75 (s, 3H), 3.67 (s, 3H), 2.23 (dddd, *J* = 14.3, 12.7, 8.9, 1.7 Hz, 2H), 2.09 – 1.85 (m, 2H), 1.73 – 1.22 (m, 10H), 1.06 (s, 3H); **^{13}C NMR** (101 MHz, $CDCl_3$): δ 176.7, 153.8, 153.2, 142.8, 115.5, 114.7, 114.6, 66.2, 57.3, 55.8, 51.4, 45.4, 34.8, 31.9, 31.7, 29.9, 29.8, 24.8, 24.8, 17.9; **IR:** 2922, 1719, 1508, 1464, 1229, 1193, 1040, 916, 824, 756 cm^{-1}; **HRMS(ESI):** Exact mass calculated for $C_{22}H_{33}O_4$ [$(M+H)^+$], 361.2372; found 361.2366; **SFC:** Daicel Chiralcel OJ-H, 5% MeOH, 2.0 mL/min, 25 °C, >99% ee (T_R (1, major enantiomer) = 12.6 min, T_R (2, minor enantiomer) = 14.8 min); **[α]$_D^{25}$** = +14.5 (c = 1.00, $CHCl_3$).

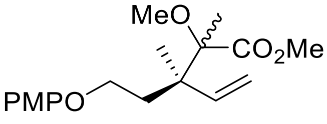

3m

Methyl (3R)-2-methoxy-3-(2-(4-methoxyphenoxy)ethyl)-2,3-dimethylpent-4-enoate (3m)

Note: The product was isolated as an inseparable 1:1 mixture of diastereomers.

Yield: 55 mg, 0.17 mmol, 85% (30:1 b/l); **TLC:** R_f = 0.21 (hexane/EtOAc 10:1, CAM stain, UV); **^{1}H NMR** (400 MHz, $CDCl_3$): δ 6.85 – 6.72 (m, 8H), 6.00 (m, 1H), 5.93 (m, 1H), 5.15 (ddd, *J* =

15.2, 10.9, 1.3 Hz, 2H), 5.02 (ddd, *J* = 17.6, 14.3, 1.3 Hz, 2H), 3.93 – 3.81 (m, 4H), 3.75 (s, 6H), 3.73 (s, 3H), 3.71 (s, 3H), 3.24 (s, 3H), 3.22 (s, 3H), 2.16 (ddd, *J* = 14.0, 8.3, 5.9 Hz, 1H), 2.01 (ddd, *J* = 8.0, 6.4, 3.7 Hz, 2H), 1.91 (ddd, *J* = 13.7, 8.8, 6.8 Hz, 1H), 1.38 (s, 3H), 1.35 (s, 3H), 1.13 (s, 3H), 1.11 (s, 3H); **^{13}C NMR** (101 MHz, $CDCl_3$): δ 173.6, 173.5, 153.8, 153.7, 153.3, 153.3, 142.0, 141.6, 115.5, 115.5, 114.9, 114.9, 114.7, 114.7, 114.3, 114.3, 85.9, 85.6, 66.0, 66.0, 55.9, 55.9, 52.6, 52.5, 51.7, 51.7, 46.3, 46.2, 34.3, 34.01, 17.9, 17.5, 16.4, 16.1; **IR:** 2950, 1728, 1507, 1464, 1228, 1130, 1102, 1038, 919, 823 cm^{-1}; **HRMS(ESI):** Exact mass calculated for $C_{18}H_{26}NaO_5$ [$(M+Na)^+$], 345.1672; found 345.1669; **SFC:** Daicel Chiralcel AS-H, 3% MeOH, 0.8 mL/min, 25 °C, >99% ee (T_R (1, major enantiomer, diastereoisomer 1) = 11.0 min, T_R (1, major enantiomer, diastereoisomer 2) = 11.0 min, T_R (2, minor enantiomer, diastereoisomer 1) = 11.9 min, T_R (3, minor enantiomer, diastereoisomer 2) = 12.9 min); **$[\alpha]_D^{28}$** = +12.2 (c = 1.00, $CHCl_3$).

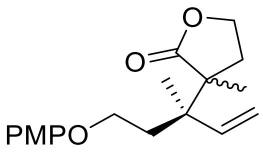

3n

3-((R)-5-(4-Methoxyphenoxy)-3-methylpent-1-en-3-yl)-3-methyldihydrofuran-2(3H)-one (3n)

Note: The product was isolated as an inseparable 1.2:1 mixture of diastereomers.

Yield: 46 mg, 0.15 mmol, 76% (30:1 b/l); **TLC:** R_f = 0.23 (hexane/EtOAc 5:1, CAM stain, UV); **^{1}H NMR** (400 MHz, $CDCl_3$): δ 6.84 – 6.77 (m, 8H), 6.12 (dd, *J* = 17.6, 10.9 Hz, 1H), 5.82 (dd, *J* = 17.4, 10.8 Hz, 1H), 5.28 – 5.25 (m, 1H), 5.25 – 5.21 (m, 1H), 5.12 (dd, *J* = 14.5, 1.2 Hz, 1H), 5.08 (dd, *J* = 14.6, 1.2 Hz, 1H), 4.31 – 4.12 (m, 4H), 3.92 – 3.81 (m, 4H), 3.76 (s, 6H), 2.64 – 2.37 (m, 3H), 2.30 – 2.15 (m, 1H), 2.01 – 1.87 (m, 2H), 1.82 (dddd, *J* = 12.9, 7.4, 6.1, 3.6 Hz, 2H), 1.30 (s, 3H), 1.28 (s, 3H), 1.18 (s, 3H), 1.18 (s, 3H); **^{13}C NMR** (101 MHz, $CDCl_3$): δ 180.4, 180.3, 153.9, 153.8, 153.1, 153.1, 141.5, 141.1, 116.7, 115.9, 115.5, 114.8, 114.7, 65.9, 65.6, 64.8, 64.8, 55.9, 48.6, 48.3, 43.6, 43.0, 33.7, 33.4, 32.6, 32.6, 19.6, 19.0, 17.3, 17.1; **IR:** 2978, 2834, 1761, 1508, 1466, 1368, 1230, 1078, 1037, 827 cm^{-1}; **HRMS(ESI):** Exact mass calculated for $C_{18}H_{24}NaO_4$ [$(M+Na)^+$], 327.1567; found 327.1566; **$[\alpha]_D^{28}$** = +19.1 (c = 1.00, $CHCl_3$).

Note: Direct determination of enantiomeric excess by chiral chromatography of **3n** was not possible because the racemic sample could not be separated. Hence, enantiomeric excess was measured by reduction with LAH and chiral chromatography of diol **SI-1**.

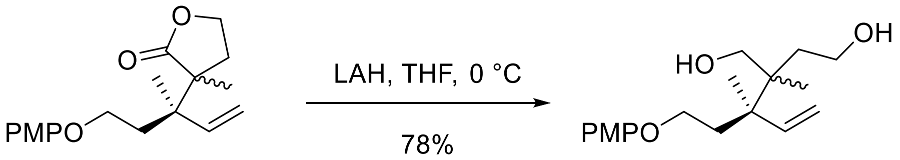

3n **SI-1**

2-((R)-5-(4-methoxyphenoxy)-3-methylpent-1-en-3-yl)-2-methylbutane-1,4-diol (SI-1). 3n (8 mg, 0.03 mmol, 1 equiv) was dissolved in THF (0.4 mL) and cooled to 0 °C. Lithium aluminum hydride (3 mg, 0.08 mmol, 3 equiv) was added and the mixture was stirred for 0.5 h at 0 °C. The reaction was quenched by dropwise addition of EtOAc (1 mL) followed by 1 M aq HCl (3 mL). The mixture was diluted with EtOAc (2 mL) and the phases were separated. The aqueous phase was extracted once with EtOAc (2 mL). The combined organic extracts were dried over $MgSO_4$ and concentrated under reduced pressure. Purification by column chromatography (hexane/EtOAc 1:1) afforded **SI-1** as a colorless oil (6 mg, 0.02 mmol, 80%).

Note: **SI-1** was isolated as an inseparable 1.2:1 mixture of diastereomers.

TLC: R_f = 0.17 (hexane/EtOAc 1:1, CAM stain); **^{1}H NMR** (500 MHz, $CDCl_3$): δ 6.86 – 6.76 (m, 8H), 5.97 (ddd, *J* = 17.5, 10.9, 8.9 Hz, 2H), 5.18 (td, *J* = 10.9, 1.3 Hz, 2H), 5.04 (dd, *J* = 15.0, 1.4 Hz, 1H), 5.00 (dd, *J* = 15.0, 1.4 Hz, 1H), 3.85 – 3.77 (m, 8H), 3.76 (s, 6H), 3.69 (dd, *J* = 11.6, 9.3 Hz, 2H), 3.50 (ddd, *J* = 23.2, 11.6, 0.6 Hz, 2H), 2.95 (s, 4H), 2.09 – 1.84 (m, 6H), 1.64 – 1.54 (m, 2H), 1.07 (d, *J* = 0.6 Hz, 6H), 0.90 (d, *J* = 0.6 Hz, 3H), 0.89 (d, *J* = 0.6 Hz, 3H); **^{13}C NMR** (126 MHz, $CDCl_3$): δ 153.9, 153.8, 153.2, 153.2, 143.7, 143.5, 115.5, 115.0, 114.8, 114.7, 66.9, 66.7, 66.2, 66.1, 59.5, 55.9, 44.4, 44.3, 43.2, 43.0, 36.1, 36.0, 34.0, 33.9, 17.3, 17.2, 16.7, 16.6; **IR:** 3308, 2955, 1508, 1466, 1230, 1106, 1039, 917, 825, 758 cm^{-1}; **HRMS(ESI):** Exact mass calculated for $C_{18}H_{28}NaO_4$ [(M+Na)$^+$], 331.1880; found 331.1874 ; **SFC:** Daicel Chiralcel AS-H, 10% MeOH, 2.0 mL/min, 25 °C, minor diastereomer: >99% ee /major diastereomer: 98% ee (T_R (1, major enantiomer, minor diastereoisomer) = 8.4 min, T_R (2, major enantiomer, major

diastereoisomer) = 10.2 min, T_R (3, minor enantiomer, major diastereoisomer) = 12.0 min, T_R (4, minor enantiomer, minor diastereoisomer) = 18.7 min); **$[\alpha]_D^{28}$** = +20.4 (c = 0.50, $CHCl_3$).

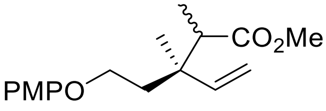

3s

Methyl (3R)-3-(2-(4-methoxyphenoxy)ethyl)-2,3-dimethylpent-4-enoate (3s)

Note 1: The product was isolated as an inseparable 1:1 mixture of diastereomers.

Note 2: Determination of enantiomeric excess by chiral chromatography of **3s** was not possible because the racemic sample could not be separated.

Yield: 9.6 mg, 0.032 mmol, 17% (20:1 b/l); **TLC:** R_f = 0.26 (hexane/EtOAc 16:1, CAM stain, UV); **^{1}H NMR** (500 MHz, $CDCl_3$): δ 6.88 – 6.74 (m, 8H), 5.92 (dd, *J* = 17.5, 10.9 Hz, 1H), 5.73 (dd, *J* = 17.5, 10.8 Hz, 1H), 5.13 (ddd, *J* = 17.9, 10.8, 1.1 Hz, 2H), 5.01 (ddd, *J* = 17.5, 13.3, 1.1 Hz, 2H), 3.93 – 3.85 (m, 4H), 3.76 (s, 6H), 3.67 (s, 3H), 3.63 (s, 3H), 2.55 – 2.45 (m, 2H), 2.09 – 1.78 (m, 4H), 1.14 – 1.12 (m, 9H), 1.09 (d, *J* = 7.1 Hz, 3H); **^{13}C NMR** (126 MHz, $CDCl_3$): δ 175.6, 175.6, 153.9, 153.2, 153.2, 143.8, 143.3, 115.6, 115.6, 114.8, 114.8, 114.4, 114.0, 65.5, 65.5, 55.9, 51.5, 51.3, 48.9, 48.2, 41.3, 41.0, 38.0, 37.6, 19.5, 18.5, 12.8, 12.6; **IR:** 2949, 1733, 1508, 1465, 1354, 1231, 1086, 1040, 918, 826 cm^{-1}; **HRMS(ESI):** Exact mass calculated for $C_{17}H_{24}NaO_4$ [$(M+Na)^+$], 315.1567; found 315.1562; **$[\alpha]_D^{29}$** = +16.0 (c = 0.21, $CHCl_3$).

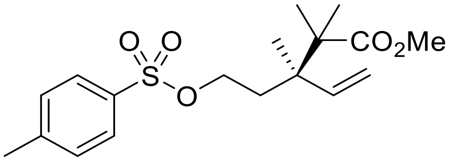

4b

Methyl (R)-2,2,3-trimethyl-3-(2-(tosyloxy)ethyl)pent-4-enoate (4b)

Yield: 53 mg, 0.15 mmol, 75% (30:1 b/l); **TLC:** R_f = 0.27 (hexane/EtOAc 8:1, CAM stain, UV); **^{1}H NMR** (400 MHz, $CDCl_3$): δ 7.75 (d, *J* = 8.3 Hz, 1H), 7.33 (d, *J* = 7.9 Hz, 1H), 5.67 (dd, *J* =

17.5, 10.9 Hz, 1H), 5.09 (dd, J = 10.9, 1.1 Hz, 1H), 4.86 (d, J = 1.1 Hz, 1H), 3.94 (ddd, J = 8.3, 6.8, 2.3 Hz, 2H), 3.60 (s, 3H), 2.44 (s, 3H), 1.84 (ddd, J = 7.6, 6.6, 3.8 Hz, 2H), 1.08 (s, 3H), 1.07 (s, 3H), 0.93 (s, 3H); **^{13}C NMR** (101 MHz, $CDCl_3$): δ 176.7, 144.8, 141.0, 133.4, 129.9, 128.0, 115.5, 68.5, 51.6, 48.7, 43.3, 34.0, 21.7, 21.4, 21.4, 17.3; **IR:** 2981, 1723, 1361, 1189, 1176, 1136, 958, 816, 663, 555 cm^{-1}; **HRMS(ESI):** Exact mass calculated for $C_{18}H_{26}NaO_5S$ [$(M+Na)^+$], 377.1393; found 377.1389; **SFC:** Daicel Chiralcel OJ-H, 1% MeOH, 2.0 mL/min, 25 °C, 98% ee (T_R (1, major enantiomer) = 11.5 min, T_R (2, minor enantiomer) = 13.3 min); **$[\alpha]_D^{25}$** = +11.9 (c = 1.00, $CHCl_3$).

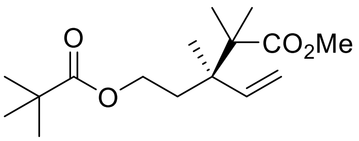

4c

Methyl (R)-2,2,3-trimethyl-3-(2-(pivaloyloxy)ethyl)pent-4-enoate (4c)

Yield: 41 mg, 0.14 mmol, 71% (40:1 b/l); **TLC:** R_f = 0.22 (hexane/EtOAc 20:1, CAM stain); **^{1}H NMR** (400 MHz, $CDCl_3$): δ 5.79 (dd, J = 17.5, 10.9 Hz, 1H), 5.14 (dd, J = 10.9, 1.2 Hz, 1H), 4.97 (dd, J = 17.5, 1.3 Hz, 1H), 3.95 (dd, J = 7.7, 6.9 Hz, 2H), 3.63 (s, 3H), 1.79 (t, J = 7.3 Hz, 2H), 1.16 (s, 9H), 1.13 (s, 3H), 1.12 (s, 3H), 1.03 (s, 3H); **^{13}C NMR** (101 MHz, $CDCl_3$): δ 178.7, 177.1, 141.8, 115.1, 62.1, 51.5, 48.8, 43.5, 38.7, 33.8, 27.3, 21.6, 21.5, 17.4; **IR:** 2976, 1725, 1480, 1283, 1259, 1154, 1109, 1033, 917, 773 cm^{-1}; **HRMS(ESI):** Exact mass calculated for $C_{16}H_{29}O_4$ [$(M+H)^+$], 285.2060; found 285.2062; **$[\alpha]_D^{25}$** = +19.0 (c = 1.00, $CHCl_3$).

Note: Direct determination of enantiomeric excess by chiral chromatography of **4c** was not possible because the racemic sample could not be separated. Hence, enantiomeric excess was measured by reduction with LAH and chiral chromatography of diol **SI-2**.

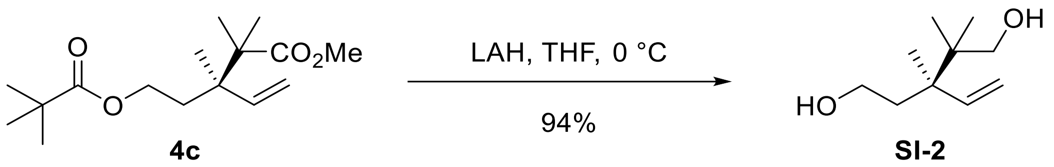

(R)-2,2,3-Trimethyl-3-vinylpentane-1,5-diol (SI-2). **4c** (24 mg, 0.084 mmol, 1.0 equiv) was dissolved in THF (0.5 mL) and cooled to 0 °C. Lithium aluminum hydride (7.7 mg, 0.20 mmol, 2.4 equiv) was added and the mixture was stirred for 1 h at 0 °C. The reaction was quenched by dropwise addition of EtOAc (0.2 mL) followed by 1 M aq HCl (3 mL). The mixture was diluted with EtOAc (3 mL) and the phases were separated. The aqueous phase was extracted once with EtOAc (2 mL). The combined organic extracts were dried over $MgSO_4$ and concentrated under reduced pressure. Purification by column chromatography (hexane/EtOAc 1:1) afforded **SI-2** as a colorless oil (14 mg, 0.082 mmol, 94%).

TLC: R_f = 0.24 (hexane/EtOAc 1:1, CAM stain); **^{1}H NMR** (400 MHz, $CDCl_3$): δ 6.00 (dd, *J* = 17.7, 10.9 Hz, 1H), 5.12 (dd, *J* = 10.9, 1.4 Hz, 1H), 4.99 (dd, *J* = 17.7, 1.4 Hz, 1H), 3.57 (dd, *J* = 7.8, 7.0 Hz, 2H), 3.51 (d, *J* = 11.1 Hz, 1H), 3.41 (d, *J* = 11.1 Hz, 1H), 1.86 – 1.74 (m, 1H), 1.70 (dt, *J* = 13.2, 7.1 Hz, 1H), 1.57 (s, 2H), 1.01 (s, 3H), 0.91 (s, 3H), 0.87 (s, 3H); **^{13}C NMR** (101 MHz, $CDCl_3$): δ 144.8, 114.0, 69.4, 60.3, 43.2, 40.8, 37.8, 20.4, 20.2, 17.0; **IR:** 3325, 2973, 2884, 1463, 1415, 1373, 1048, 1008, 912, 673 cm^{-1}; **HRMS(ESI):** Exact mass calculated for $C_{10}H_{20}NaO_2$ [$(M+Na)^+$], 195.1356; found 195.1353; **SFC:** Daicel Chiralcel AS-H, 3% MeOH, 2.0 mL/min, 25 °C, 96% ee (T_R (1, minor enantiomer) = 10.2 min, T_R (2, major enantiomer) = 11.7 min); **$[\alpha]_D^{28}$** = +21.1 (c = 1.00, $CHCl_3$).

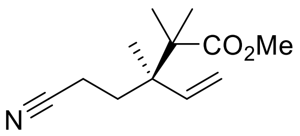

4d

Methyl (R)-3-(2-cyanoethyl)-2,2,3-trimethylpent-4-enoate (4d)

Yield: 24 mg, 0.12 mmol, 58% (30:1 b/l); **TLC:** R_f = 0.25 (hexane/EtOAc 10:1, CAM stain); **^{1}H NMR** (400 MHz, $CDCl_3$): δ 5.71 (dd, *J* = 17.5, 10.9 Hz, 1H), 5.24 (dd, *J* = 10.9, 1.1 Hz, 1H), 5.01 (dd, *J* = 17.5, 1.1 Hz, 1H), 3.66 (s, 3H), 2.14 (ddd, *J* = 9.6, 6.1, 2.1 Hz, 2H), 1.93 – 1.86 (m, 2H),

1.15 (s, 3H), 1.14 (s, 3H), 0.99 (s, 3H); **^{13}C NMR** (101 MHz, $CDCl_3$): δ 176.6, 140.4, 120.4, 116.5, 51.6, 48.6, 44.2, 31.2, 21.5, 21.5, 16.3, 12.9; **IR:** 2982, 2952, 2260, 1723, 1451, 1267, 1162, 1132, 1102, 923 cm^{-1}; **HRMS(ESI):** Exact mass calculated for $C_{12}H_{19}NNaO_2$ [$(M+Na)^+$], 232.1308; found 232.1303; **SFC:** Daicel Chiralcel OJ-H, 2% MeOH, 2.0 mL/min, 25 °C, >99% ee (T_R (1, major enantiomer) = 12.3 min, T_R (2, minor enantiomer) = 14.6 min); **$[\alpha]_D^{25}$** = +22.3 (c = 0.50, $CHCl_3$).

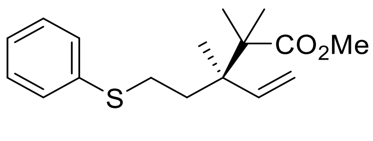

4e

Methyl (R)-2,2,3-trimethyl-3-(2-(phenylthio)ethyl)pent-4-enoate (4e)

Yield: 32 mg, 0.11 mmol, 54% (3:1 b/l); **TLC:** R_f = 0.28 (hexane/EtOAc 25:1, CAM stain, UV); **^{1}H NMR** (400 MHz, $CDCl_3$): δ 7.32 – 7.22 (m, 4H), 7.20 – 7.13 (m, 1H), 5.77 (dd, *J* = 17.5, 10.9 Hz, 1H), 5.18 (dd, *J* = 10.9, 1.3 Hz, 1H), 4.98 (dd, *J* = 17.5, 1.3 Hz, 1H), 3.59 (s, 3H), 2.73 (dd, *J* = 9.2, 7.8 Hz, 2H), 1.84 – 1.73 (m, 2H), 1.11 (s, 6H), 1.04 (s, 3H); **^{13}C NMR** (101 MHz, $CDCl_3$): δ 177.0, 141.8, 136.9, 129.1, 129.0, 125.9, 115.3, 51.5, 48.9, 44.7, 35.0, 29.5, 21.6, 21.6, 16.9 cm^{-1}; **HRMS(ESI):** Exact mass calculated for $C_{17}H_{24}NaO_2S$ [$(M+Na)^+$], 315.1389; found 315.1385; **SFC:** Daicel Chiralcel OJ-H, 100% CO_2, 1.0 mL/min, 25 °C, >99% ee (T_R (1, major enantiomer) = 38.5 min, T_R (2, minor enantiomer) = 41.4 min); **$[\alpha]_D^{25}$** = +38.2 (c = 1.00, $CHCl_3$).

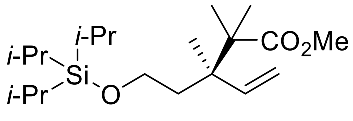

4f

Methyl (R)-2,2,3-trimethyl-3-(2-((triisopropylsilyl)oxy)ethyl)pent-4-enoate (4f)

Yield: 59 mg, 0.16 mmol, 82% (18:1 b/l); **TLC:** R_f = 0.24 (hexane/EtOAc 30:1, CAM stain); **^{1}H NMR** (400 MHz, $CDCl_3$): δ 5.81 (dd, *J* = 17.5, 10.9 Hz, 1H), 5.10 (dd, *J* = 10.9, 1.4 Hz, 1H), 4.93 (dd, *J* = 17.5, 1.4 Hz, 1H), 3.63 (s, 3H), 3.62 – 3.50 (m, 2H), 1.80 – 1.68 (m, 2H), 1.13 (s, 6H),

1.06 – 1.02 (m, 21H), 1.01 (s, 3H); **^{13}C NMR** (101 MHz, $CDCl_3$): δ 177.2, 142.6, 114.3, 60.8, 51.4, 48.9, 43.3, 38.3, 21.6, 21.5, 18.2, 17.4, 12.1; **IR:** 2944, 2866, 1728, 1463, 1253, 1092, 1011, 882, 808, 680 cm^{-1}; **HRMS(ESI):** Exact mass calculated for $C_{20}H_{41}O_3Si$ [$(M+H)^+$], 357.2819; found 357.2820; **$[\alpha]_D^{25}$** = +13.7 (c = 1.00, $CHCl_3$).

Note: Direct determination of enantiomeric excess by chiral chromatography of **4f** was not possible because the racemic sample could not be separated. Hence, enantiomeric excess was measured by TIPS deprotection/reduction with LAH and chiral chromatography of alcohol **SI-2**.

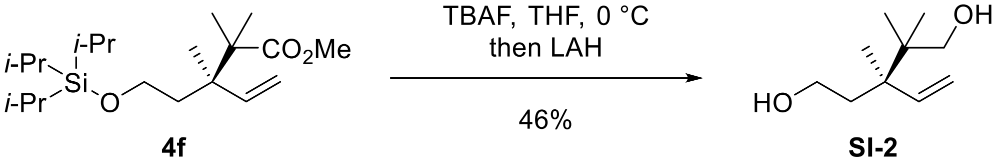

(R)-2,2,3-Trimethyl-3-vinylpentane-1,5-diol (SI-2). 4f (23 mg, 0.065 mmol, 1.0 equiv) was dissolved in THF (0.5 mL) and cooled to 0 °C. A 1 M solution of tetrabutylammonium fluoride in THF (0.13 mL, 0.13 mmol, 2.0 equiv) was added and the mixture was stirred for 1 h at 0 °C. Lithium aluminum hydride (10 mg, 0.26 mmol, 4.1 equiv) was added and the mixture was stirred for 1 h at 0 °C. The reaction was quenched by dropwise addition of EtOAc (0.2 mL) followed by 1 M aq HCl (3 mL). The mixture was diluted with EtOAc (3 mL) and the phases were separated. The aqueous phase was extracted once with EtOAc (2 mL). The combined organic extracts were dried over $MgSO_4$ and concentrated under reduced pressure. Purification by column chromatography (hexane/EtOAc 1:1) afforded **SI-2** as a colorless oil (5.1 mg, 0.030 mmol, 46%).

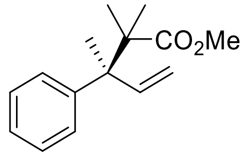

4g

Methyl (S)-2,2,3-trimethyl-3-phenylpent-4-enoate (4g)

Yield: 43 mg, 0.18 mmol, 92% (40:1 b/l); **TLC:** R_f = 0.23 (hexane/EtOAc 25:1, CAM stain, UV); **^{1}H NMR** (400 MHz, $CDCl_3$): δ 7.32 (m, 2H), 7.26 (m, 2H), 7.20 (m, 1H), 6.80 (dd, J = 17.4, 11.0

Hz, 1H), 5.19 (dd, J = 11.0, 1.3 Hz, 1H), 5.04 (dd, J = 17.4, 1.3 Hz, 1H), 3.51 (s, 3H), 1.55 (s, 3H), 1.15 (s, 3H), 1.13 (s, 3H); **^{13}C NMR** (101 MHz, $CDCl_3$): δ 177.1, 144.6, 143.4, 128.4, 127.4, 126.4, 114.3, 51.4, 49.5, 48.7, 22.4, 22.3, 21.2; **IR:** 2979, 1722, 1464, 1266, 1138, 1101, 1029, 918, 754, 701 cm^{-1}; **HRMS(ESI):** Exact mass calculated for $C_{15}H_{20}NaO_2$ [$(M+Na)^+$], 255.1356; found 255.1354; **SFC:** Daicel Chiralcel OJ-H, 2% MeOH, 2.0 mL/min, 25 °C, 98% ee (T_R (1, major enantiomer) = 11.1 min, T_R (2, minor enantiomer) = 16.7 min); **$[\alpha]_D^{23}$** = +46.0 (c = 1.00, $CHCl_3$).

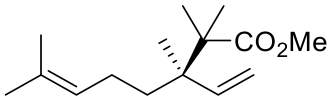

4h

Methyl (R)-2,2,3,7-tetramethyl-3-vinyloct-6-enoate (4h)

Yield: 36 mg, 0.15 mmol, 76% (25:1 b/l); **TLC:** R_f = 0.21 (hexane/EtOAc 40:1, CAM stain, UV); **^{1}H NMR** (400 MHz, $CDCl_3$): δ 5.78 (dd, J = 17.5, 10.9 Hz, 1H), 5.12 (dd, J = 10.9, 1.5 Hz, 1H), 5.06 (dddd, J = 7.1, 5.7, 2.8, 1.4 Hz, 1H), 4.94 (dd, J = 17.5, 1.5 Hz, 1H), 3.63 (s, 3H), 1.85 – 1.70 (m, 2H), 1.66 (d, J = 1.3 Hz, 3H), 1.56 (d, J = 1.3 Hz, 3H), 1.45 – 1.36 (m, 2H), 1.12 (s, 6H), 1.02 – 0.99 (m, 3H); **^{13}C NMR** (101 MHz, $CDCl_3$): δ 177.5, 142.8, 131.3, 125.1, 114.3, 51.4, 48.9, 44.5, 35.5, 25.8, 23.5, 21.8, 21.7, 17.7, 16.9; **IR:** 2977, 1727, 1462, 1433, 1376, 1269, 1134, 1009, 913, 836 cm^{-1}; **HRMS(ESI):** Exact mass calculated for $C_{15}H_{26}NaO_2$ [$(M+Na)^+$], 261.1825; found 261.1823; **$[\alpha]_D^{23}$** = +38.4 (c = 1.00, $CHCl_3$).

Note: Direct determination of enantiomeric excess by chiral chromatography of **4h** was not possible because the racemic sample could not be separated. Hence, enantiomeric excess was measured by reduction with LAH and chiral chromatography of alcohol **SI-3**.

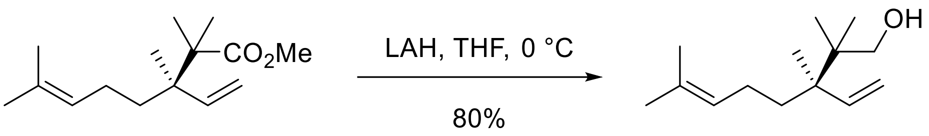

4h **SI-3**

(R)-2,2,3,7-Tetramethyl-3-vinyloct-6-en-1-ol (SI-3). 4h (100 mg, 0.42 mmol, 1.0 equiv) was dissolved in THF (4 mL) and cooled to 0 °C. Lithium aluminum hydride (19 mg, 0.50 mmol, 1.2 equiv) was added and the mixture was stirred for 0.5 h at 0 °C. The reaction was quenched by dropwise addition of EtOAc (1 mL) followed by 1 M aq HCl (10 mL). The mixture was diluted with EtOAc (10 mL) and the phases were separated. The aqueous phase was extracted once with EtOAc (8 mL). The combined organic extracts were dried over $MgSO_4$ and concentrated under reduced pressure. Purification by column chromatography (hexane/EtOAc 10:1) afforded **SI-3** as a colorless oil (71 mg, 0.34 mmol, 80%).

TLC: R_f = 0.31 (hexane/EtOAc 10:1, CAM stain); **^{1}H NMR** (400 MHz, $CDCl_3$): δ 5.90 (dd, *J* = 17.6, 10.9 Hz, 1H), 5.12 (dd, *J* = 10.9, 1.6 Hz, 1H), 5.08 (tdd, *J* = 5.7, 2.9, 1.4 Hz, 1H), 4.96 (dd, *J* = 17.6, 1.6 Hz, 1H), 3.53 (d, *J* = 11.1 Hz, 1H), 3.39 (d, *J* = 11.1 Hz, 1H), 1.82 – 1.71 (m, 2H), 1.67 (s, 3H), 1.57 (s, 3H), 1.45 (s, 1H), 1.40 (dt, *J* = 10.4, 6.9 Hz, 2H), 0.98 (s, 3H), 0.92 (s, 3H), 0.86 (s, 3H); **^{13}C NMR** (101 MHz, $CDCl_3$): δ 145.0, 131.3, 125.3, 113.8, 69.8, 44.1, 40.9, 34.9, 25.9, 23.2, 20.6, 20.3, 17.7, 16.6; **IR:** 3355, 2971, 2928, 2881, 1635, 1453, 1374, 1010, 911, 836 cm^{-1}; **HRMS(ESI):** Exact mass calculated for $C_{14}H_{26}NaO$ [$(M+Na)^+$], 233.1876; found 233.1872; **SFC:** Daicel Chiralpak IA, 2% MeOH, 2.0 mL/min, 25 °C, 89% ee (T_R (1, minor enantiomer) = 8.2 min, T_R (2, major enantiomer) = 9.4 min) **$[\alpha]_D^{25}$** = +46.4 (c = 1.00, $CHCl_3$).

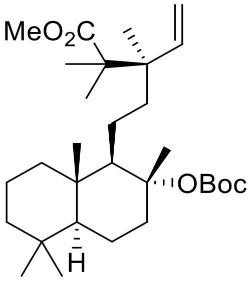

4i

Methyl (R)-3-(2-((1R,2R,4aS,8aS)-2-((tert-butoxycarbonyl)oxy)-2,5,5,8a-tetramethyldeca-hydronaphthalen-1-yl)ethyl)-2,2,3-trimethylpent-4-enoate (4i)

Yield: 66 mg, 0.13 mmol, 76% (20:1 b/l); **TLC:** R_f = 0.28 (hexane/EtOAc 25:1, CAM stain); **^{1}H NMR** (400 MHz, $CDCl_3$): δ 5.79 (dd, *J* = 17.5, 10.9 Hz, 1H), 5.10 (dd, *J* = 10.8, 1.5 Hz, 1H), 4.92 (dd, *J* = 17.5, 1.6 Hz, 1H), 3.61 (s, 3H), 2.28 (dt, *J* = 12.4, 3.3 Hz, 1H), 2.02 (td, *J* = 12.7, 4.2 Hz, 1H), 1.76 – 1.50 (m, 5H), 1.48 – 1.31 (m, 15H), 1.31 – 1.18 (m, 2H), 1.17 – 1.00 (m, 12H), 0.98 – 0.90 (m, 1H), 0.85 (s, 3H), 0.77 (s, 3H), 0.76 (s, 3H); **^{13}C NMR** (101 MHz, $CDCl_3$): δ 177.4, 152.1, 143.4, 114.0, 88.8, 80.5, 57.5, 55.5, 51.3, 49.0, 45.0, 42.0, 39.8, 39.7, 38.0, 37.6, 33.4, 33.3, 28.1, 21.9, 21.7, 21.6, 21.4, 20.6, 20.3, 18.6, 16.6, 15.9; **IR:** 2947, 1730, 1459, 1390, 1368, 1277, 1254, 1166, 1130, 912 cm^{-1}; **HRMS(ESI):** Exact mass calculated for $C_{30}H_{52}NaO_5$ [$(M+Na)^+$], 515.3707; found 515.3704; **Diastereomeric excess:** single diastereomer; **$[\alpha]_D^{23}$** = +6.0 (c = 1.00, $CHCl_3$).

Note: Diastereomeric excess was determined by ^{1}H NMR analysis of the unpurified reaction mixture.

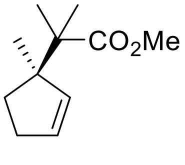

4j

Methyl (R)-2-methyl-2-(1-methylcyclopent-2-en-1-yl)propanoate (4j)

Yield: 30 mg, 0.16 mmol, 82% (20:1 b/l); **TLC:** R_f = 0.32 (hexane/EtOAc 40:1, CAM stain); **^{1}H NMR** (400 MHz, $CDCl_3$): δ 5.71 (dt, *J* = 5.7, 2.1 Hz, 1H), 5.64 (dt, *J* = 5.7, 2.3 Hz, 1H), 3.64 (s, 3H), 2.42 – 2.18 (m, 2H), 2.12 – 1.98 (m, 1H), 1.51 – 1.38 (m, 1H), 1.14 (s, 6H), 0.99 (s, 3H); **^{13}C**

NMR (101 MHz, $CDCl_3$): δ 178.0, 137.6, 129.3, 54.4, 51.5, 48.0, 33.3, 31.6, 23.0, 22.1, 21.8; **IR:** 2951, 1727, 1459, 1268, 1189, 1136, 1116, 1092, 990, 739 cm^{-1}; **HRMS(ESI):** Exact mass calculated for $C_{11}H_{18}NaO_2$ [$(M+Na)^+$], 205.1199; found 205.1197; $[\alpha]_D^{26}$ = –16.0 (c = 1.00, $CHCl_3$).

Note: Direct determination of enantiomeric excess by chiral chromatography of **4j** was not possible because the racemic sample could not be separated. Hence, enantiomeric excess was measured by oxidation with OsO_4 and chiral chromatography of diol **SI-4**.

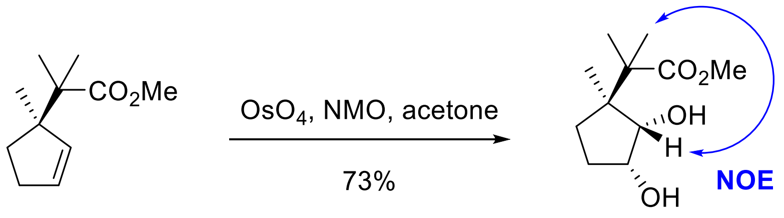

4j **SI-4**

single diastereomer

Methyl 2-((1S,2S,3R)-2,3-dihydroxy-1-methylcyclopentyl)-2-methylpropanoate (SI-4). 4j (15 mg, 0.082 mmol, 1.0 equiv) was dissolved in acetone (0.3 mL) and *N*-methylmorpholine *N*-oxide (19 mg, 0.16 mmol, 2.0 equiv) was added. A 4% solution of OsO_4 in water (0.05 mL, 0.02 mmol, 10 mol %) was added and the mixture was stirred for 3 h at rt. A mixture of sat. aq. $Na_2S_2O_3$ solution (2 mL) and sat. aq. $NaHCO_3$ (1 mL) solution was added and the mixture was stirred for 30 min. Water (1 mL) and EtOAc (2 mL) were added. The phases were separated and the aqueous phase extracted once with EtOAc (2 mL). The combined organic extracts were dried over $MgSO_4$ and concentrated under reduced pressure. Purification by column chromatography (hexane/EtOAc 10:1) afforded **SI-4** as a colorless oil (13 mg, 0.060 mmol, 73%).

TLC: R_f = 0.30 (hexane/EtOAc 1:1, $KMnO_4$ stain); **^{1}H NMR** (400 MHz, $CDCl_3$): δ 4.18 (tdd, *J* = 5.7, 3.2, 1.9 Hz, 1H), 4.10 (dd, *J* = 6.0, 3.5 Hz, 1H), 3.77 (d, *J* = 3.5 Hz, 1H), 3.69 (s, 3H), 2.67 (s, 1H), 1.86 – 1.58 (m, 3H), 1.56 – 1.47 (m, 1H), 1.21 (s, 3H), 1.19 (s, 3H), 1.03 (s, 3H); **^{13}C NMR** (101 MHz, $CDCl_3$): δ 180.0, 75.0, 73.5, 52.2, 48.6, 48.2, 31.8, 29.5, 22.3, 21.7, 18.1; **IR:** 3425, 2952, 1724, 1463, 1268, 1193, 1157, 1120, 1064, 983 cm^{-1}; **HRMS(ESI):** Exact mass calculated for $C_{11}H_{20}NaO_4$ [$(M+Na)^+$], 239.1254; found 239.1254; **HPLC:** Daicel Chiralpak AD-H, 5% *i*-

PrOH, 1.0 mL/min, 25 °C, 94% ee (T_R (1, minor enantiomer) =15.2 min, T_R (2, major enantiomer) = 18.2 min) **$[\alpha]_D^{27}$** = –4.3 (c = 0.50, $CHCl_3$).

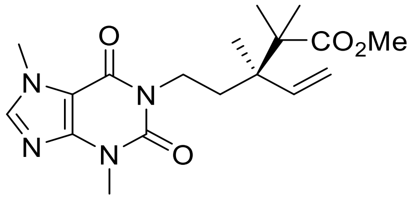

4k

Methyl (R)-3-(2-(3,7-dimethyl-2,6-dioxo-2,3,6,7-tetrahydro-1H-purin-1-yl)ethyl)-2,2,3-trimethylpent-4-enoate (4k)

Yield: 45 mg, 0.12 mmol, 62% (40:1 b/l); **TLC:** R_f = 0.31 (Et_2O/MeOH 30:1, CAM stain, UV); **^{1}H NMR** (400 MHz, $CDCl_3$): δ 7.48 (d, *J* = 0.7 Hz, 1H), 5.92 (dd, *J* = 17.5, 10.9 Hz, 1H), 5.22 (dd, *J* = 10.9, 1.3 Hz, 1H), 5.09 (dd, *J* = 17.6, 1.2 Hz, 1H), 3.96 (s, 3H), 3.94 – 3.76 (m, 2H), 3.61 (s, 3H), 3.54 (s, 3H), 1.84 – 1.64 (m, 2H), 1.16 (s, 3H), 1.12 (s, 3H), 1.12 (s, 3H); **^{13}C NMR** (101 MHz, $CDCl_3$): δ 177.1, 155.3, 151.5, 148.8, 141.8, 141.4, 115.2, 107.8, 51.5, 48.9, 43.7, 38.6, 33.7, 32.7, 29.8, 21.6, 21.5, 17.1; **IR:** 2980, 2950, 1702, 1656, 1550, 1455, 1358, 1140, 919, 764 cm^{-1}; **HRMS(ESI):** Exact mass calculated for $C_{18}H_{27}N_4O_4$ $[(M+H)^+]$, 363.2027; found 363.2025; **HPLC:** Daicel Chiralpak AD-H, 30% *i*-PrOH, 1.0 mL/min, 25 °C, 95% ee (T_R (1, minor enantiomer) = 7.3 min, T_R (2, major enantiomer) = 9.8 min); **$[\alpha]_D^{25}$** = +29.0 (c = 1.00, $CHCl_3$).

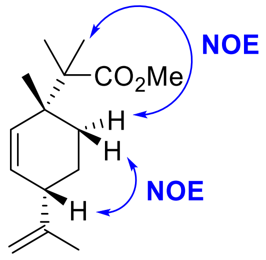

4l

Methyl 2-methyl-2-((1R,4S)-1-methyl-4-(prop-1-en-2-yl)cyclohex-2-en-1-yl)propanoate (4l)

Note: The reaction was set up according to the standard conditions. After stirring the reaction for 1 h at –20 °C, the reaction was warmed to rt. Additional 2 equiv of **2a** were added and stirring was continued for 20 h at rt. Work-up and purification occurred as described in the general procedure.

Yield: 24 mg, 0.10 mmol, 51% (20:1 b/l); **TLC:** R_f = 0.26 (hexane/EtOAc 40:1, CAM stain); **^{1}H NMR** (500 MHz, $CDCl_3$): δ 5.82 (dt, *J* = 10.4, 1.7 Hz, 1H), 5.58 (ddd, *J* = 10.4, 4.8, 1.4 Hz, 1H), 4.83 (t, *J* = 1.9 Hz, 1H), 4.68 – 4.63 (m, 1H), 3.64 (s, 3H), 2.60 – 2.52 (m, 1H), 1.84 – 1.76 (m, 1H), 1.75 (s, 3H), 1.74 – 1.67 (m, 1H), 1.66 – 1.61 (m, 1H), 1.16 (s, 3H), 1.13 (s, 3H), 1.13 – 1.09 (m, 1H), 1.00 (s, 3H); **^{13}C NMR** (126 MHz, $CDCl_3$): δ 177.6, 147.6, 134.0, 128.3, 112.1, 51.5, 48.6, 40.8, 39.7, 26.4, 22.9, 22.3, 22.3, 21.5, 21.4; **IR:** 2947, 1727, 1456, 1372, 1274, 1236, 1149, 1111, 896, 747 cm^{-1}; **HRMS(ESI):** Exact mass calculated for $C_{15}H_{24}NaO_2$ [$(M+Na)^+$], 259.1669; found 259.1667; **Diastereomeric excess:** single diastereomer; **$[\alpha]_D^{27}$** = –146.3 (c = 1.00, $CHCl_3$).

Note: Diastereomeric excess was determined by ^{1}H NMR analysis of the unpurified reaction mixture.

Characterization of the mixture of elimination products:

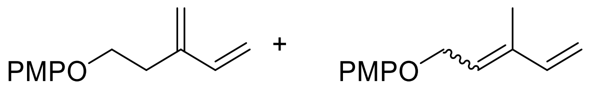

3o 1 **3p/3q** 2.8:1.3

1-Methoxy-4-((3-methylenepent-4-en-1-yl)oxy)benzene (3o) / 1-methoxy-4-((3-methylpent-3-en-1-yl)oxy)benzene (3p/3q).

Note: The elimination products **3o** and **3p**/**3q** were isolated as an inseparable 1:2.8:1.3 mixture.

TLC: R_f = 0.32 (hexane/EtOAc 20:1, CAM stain, UV); **^{1}H NMR** (400 MHz, $CDCl_3$): δ 6.90 – 6.81 (m, 12H), 6.81 – 6.71 (m, 1H), 6.52 – 6.34 (m, 2H), 5.78 – 5.72 (m, 1H), 5.70 – 5.62 (m, 1H), 5.39 – 5.18 (m, 5H), 5.16 – 5.04 (m, 3H), 4.66 – 4.61 (m, 4H), 4.06 (t, J = 7.2 Hz, 2H), 3.77 (s, 9H), 2.70 (td, J = 7.1, 1.1 Hz, 2H), 1.91 (d, J = 1.2 Hz, 3H), 1.84 (q, J = 0.9 Hz, 3H); **^{13}C NMR** (101 MHz, $CDCl_3$): δ 154.0, 154.0, 154.0, 153.1, 153.0, 142.7, 140.6, 138.8, 137.5, 136.7, 133.1, 127.2, 125.2, 117.7, 116.1, 115.8, 115.8, 115.7, 114.8, 114.8, 113.8, 113.4, 67.5, 65.5, 64.5, 55.9, 31.4, 19.9, 12.3; **IR:** 2934, 1609, 1505, 1225, 1180, 1037, 1007, 903, 822, 738cm^{-1}; **HRMS(ESI):** Exact mass calculated for $C_{13}H_{17}O_2$ [(M+H)$^+$], 205.1223; found 205.1223.

4. Preparation of Starting Materials

Overview: Synthesis of 1a in 4-5 steps

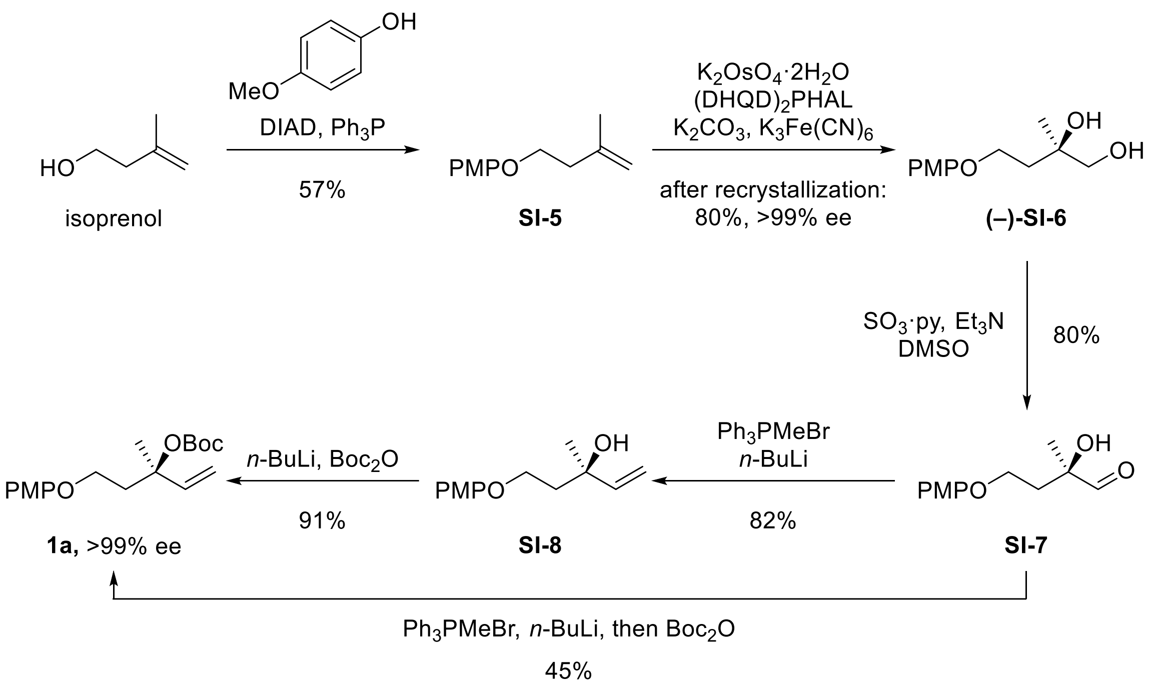

Overview: Preparation of allylic carbonates 1b-1f, 1k from 1a

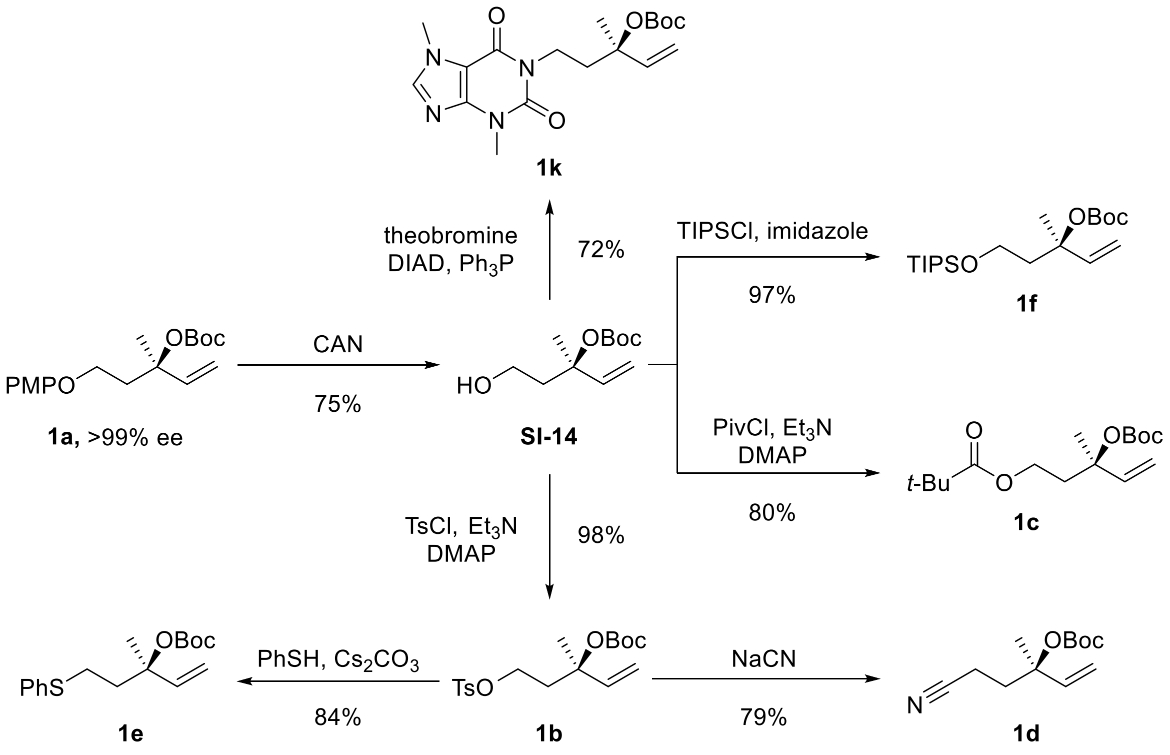

Preparation of enantioenriched tertiary allylic alcohols

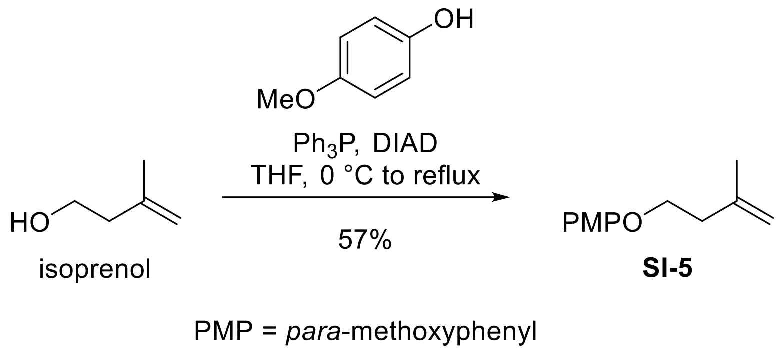

Note: The preparation of **SI-7** from isoprenol followed a literature procedure.[4-5]

1-Methoxy-4-((3-methylbut-3-en-1-yl)oxy)benzene (SI-5). Isoprenol (15.0 g, 174 mmol, 1.00 equiv), *para*-methoxyphenol (28.0 g, 225 mmol, 1.29 equiv) and triphenylphosphine (59.1 g, 225 mmol, 1.29 equiv) were dissolved in THF (500 mL) and the solution was cooled to 0 °C. Diisopropyl azodicarboxylate (44.3 mL, 225 mmol, 1.29 equiv) was added slowly. After stirring for 20 min at 0 °C, the reaction was heated to reflux and stirred for 3 h. The reaction was cooled tor rt and concentrated under reduced pressure. Triphenylphosphine oxide was precipitated by triturating the oily residue with hexane (1.5 L). The supernatant was filtered off and concentrated under reduced pressure. Purification by column chromatography (pentane/Et_2O 100:1) afforded **SI-5** as a colorless oil (19 g, 99 mmol, 57%).

TLC: R_f = 0.25 (pentane/Et_2O 100:1, CAM stain, UV); **^{1}H NMR** (400 MHz, $CDCl_3$): δ 6.84 (m, 4H), 4.82 (d, J = 17.4 Hz, 2H), 4.03 (t, J = 6.9 Hz, 2H), 3.77 (d, J = 0.7 Hz, 3H), 2.48 (t, J = 6.9 Hz, 1H), 1.81 (s, 1H); **^{13}C NMR** (101 MHz, $CDCl_3$): δ 154.0, 153.2, 142.5, 115.7, 114.8, 112.0, 67.3, 55.9, 37.5, 23.0; **IR:** 2936, 1712, 1506, 1467, 1226, 1180, 1107, 1039, 824, 736 cm^{-1}; **HRMS(ESI):** Exact mass calculated for $C_{12}H_{17}O_2$ [(M+H)$^+$], 193.1223; found 193.1224.

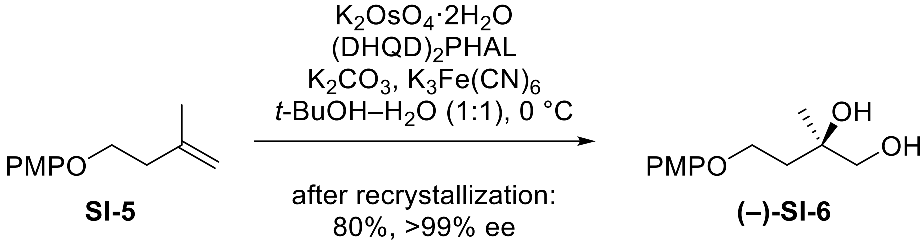

(R)-4-(4-Methoxyphenoxy)-2-methylbutane-1,2-diol ((–)-SI-6). K_2CO_3 (27.1 g, 196 mmol, 3.00 equiv), $(DHQD)_2PHAL$ (510 mg, 0.654 mmol, 1.00 mol %) and $K_3Fe(CN)_6$ (64.6 g, 196 mmol, 3.00 equiv) were mixed, grinded to a fine powder and dissolved in 1:1 *t*-BuOH–H_2O (650 mL). The orange biphasic mixture was cooled to 0 °C and $K_2OsO_4·2H_2O$ (48 mg, 0.13 mmol, 0.20 mol %) was added. After stirring 10 min, a solution of **SI-5** (12.6 g, 65.4 mmol, 1.00 equiv) in 1:1 *t*-BuOH–H_2O (150 mL) was added and the reaction was stirred for 3.5 h at 0 °C. Solid Na_2SO_3 (82.5 g, 654 mmol, 10.0 equiv) was added and the mixture was warmed to rt. After 30 min, the mixture was diluted with EtOAc (1 L) and water (1 L). The phases were separated and the aqueous phase extracted once with EtOAc (500 mL). The combined organic extracts were dried over $MgSO_4$ and concentrated under reduced pressure. Purification by column chromatography (CH_2Cl_2/MeOH 20:1) afforded a white solid of (–)-**SI-6** (14 g, 63 mmol, 96%, 97% ee). Additional recrystallization from refluxing 10:1 hexane–EtOAc (270 mL) afforded **(–)-SI-6** (12 g, 52 mmol, 80%, >99% ee) as colorless, needle crystals.

TLC: R_f = 0.23 (CH_2Cl_2/MeOH 20:1, CAM stain, UV); **^{1}H NMR** (400 MHz, $CDCl_3$): δ 6.91 – 6.77 (m, 4H), 4.29 – 4.04 (m, 2H), 3.77 (s, 3H), 3.61 – 3.40 (m, 2H), 2.87 (d, *J* = 1.6 Hz, 1H), 2.55 – 2.42 (m, 1H), 2.10 (ddd, *J* = 14.8, 7.9, 4.6 Hz, 1H), 1.91 (ddd, *J* = 14.9, 6.5, 4.3 Hz, 1H), 1.25 (s, 3H); **^{13}C NMR** (101 MHz, $CDCl_3$): δ 154.4, 152.5, 115.7, 114.9, 72.5, 70.2, 65.6, 55.9, 37.7, 24.3; **IR:** 3401, 2934, 1508, 1466, 1395, 1229, 1181, 1038, 826, 735 cm^{-1}; **HRMS(ESI):** Exact mass calculated for $C_{12}H_{18}NaO_4$ [$(M+Na)^+$], 249.1097; found 249.1095; **$[\alpha]_D^{25}$** = –8.8 (c = 1.00, $CHCl_3$).

For the racemic sample:

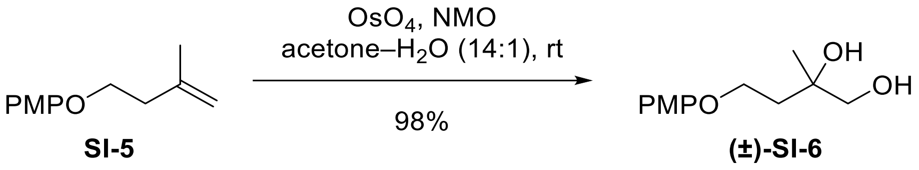

4-(4-Methoxyphenoxy)-2-methylbutane-1,2-diol ((±)-SI-6). SI-5 (6.02 g, 31.3 mmol, 1.00 equiv) was dissolved in acetone (100 mL) and *N*-methylmorpholine *N*-oxide (7.34 g, 62.6 mmol, 2.00 equiv) was added. A 4% solution of OsO_4 in water (7.16 mL, 1.57 mmol, 5 mol %) was added and the mixture was stirred for 1.5 h at rt. A mixture of sat. aq. $Na_2S_2O_3$ solution (50 mL) and sat. aq. $NaHCO_3$ (50 mL) solution was added and the mixture was stirred for 30 min. Water (300 mL) and EtOAc (500 mL) were added. The phases were separated and the aqueous phase extracted once with EtOAc (300 mL). The combined organic extracts were dried over $MgSO_4$ and concentrated under reduced pressure. Purification by column chromatography (CH_2Cl_2/MeOH 20:1) afforded a white solid of (±)-**SI-6** (7.0 g, 31 mmol, 98%).

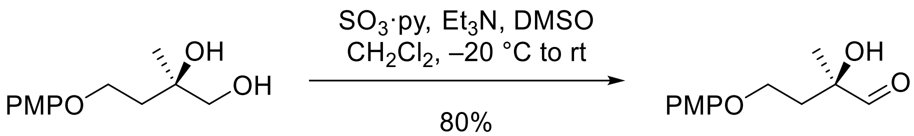

(–)-SI-6 → **SI-7**

(R)-2-Hydroxy-4-(4-methoxyphenoxy)-2-methylbutanal (SI-7). (–)-SI-6 (11.1 g, 48.8 mmol, 1.00 equiv), Et_3N (36.0 mL, 258 mmol, 5.28 equiv) and DMSO (37.0 mL, 521 mmol, 10.7 equiv) were dissolved in CH_2Cl_2 (350 mL) and the mixture was cooled to –20 °C. SO_3·py (23.3 g, 146 mmol, 3.00 equiv) was added at once and the mixture was stirred for 3 h at –20 °C. The mixture was warmed to rt and diluted with Et_2O (600 mL) and water (500 mL). The phases were separated and the aqueous phase extracted once with Et_2O (300 mL). The combined organic extracts were dried over $MgSO_4$ and concentrated under reduced pressure. Purification by column chromatography (hexane/EtOAc 4:1) afforded a white solid of **SI-7** (9.3 g, 39 mmol, 80%).

TLC: R_f = 0.33 (hexane/EtOAc 4:1, CAM stain, UV); **^{1}H NMR** (400 MHz, $CDCl_3$): δ 9.62 (d, *J* = 0.8 Hz, 1H), 6.84 – 6.72 (m, 4H), 4.00 (qd, *J* = 9.5, 4.0 Hz, 2H), 3.75 (s, 3H), 3.61 (s, 1H), 2.35 (ddd, *J* = 14.5, 9.5, 4.7 Hz, 1H), 2.06 (ddd, *J* = 14.8, 4.2, 3.3 Hz, 1H), 1.34 (s, 3H); **^{13}C NMR** (101 MHz, $CDCl_3$): δ 202.5, 154.3, 152.3, 115.6, 114.8, 76.6, 63.8, 55.9, 37.7, 23.8; **IR:** 3463, 2933, 1731, 1508, 1468, 1228, 1180, 1036, 826, 737 cm^{-1}; **HRMS(ESI):** Exact mass calculated for $C_{12}H_{16}NaO_4$ [$(M+Na)^+$], 247.0941; found 247.0940; **$[\alpha]_D^{25}$** = –41.1 (c = 1.00, $CHCl_3$).

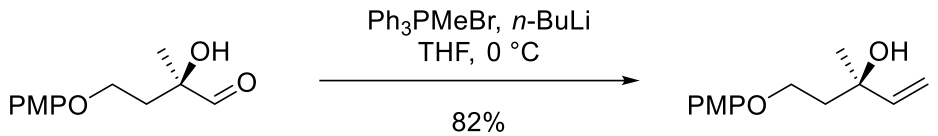

SI-7 **SI-8**

(R)-5-(4-Methoxyphenoxy)-3-methylpent-1-en-3-ol (SI-8). Methyltriphenylphosphonium bromide (30.6 g, 85.6 mmol, 2.53 equiv) was suspended in anhydrous THF (136 mL) and cooled to 0 °C. 1.6 M *n*-BuLi in hexane (51 mL, 82 mmol, 2.4 equiv) was added and the orange mixture was stirred for 1 h at 0 °C. A solution of **SI-7** (7.60 g, 33.9 mmol, 1.00 equiv) in THF (40 mL) was added at 0 °C and the mixture was stirred for 30 min. Sat. aq. NH_4Cl (200 mL) was added and the mixture was diluted with Et_2O (300 mL). The phases were separated and the aqueous phase extracted once with Et_2O (200 mL). The combined organic extracts were dried over $MgSO_4$ and concentrated under reduced pressure. Purification by column chromatography (hexane/EtOAc 6:1) afforded a colorless oil of **SI-8** (6.2 g, 160 mmol, 82%).

TLC: R_f = 0.21 (hexane/EtOAc 6:1, CAM stain, UV); **^{1}H NMR** (500 MHz, $CDCl_3$): δ 6.89 – 6.78 (m, 4H), 5.94 (dd, *J* = 17.2, 10.7 Hz, 1H), 5.31 (dd, *J* = 17.2, 1.4 Hz, 1H), 5.10 (dd, *J* = 10.7, 1.4 Hz, 1H), 4.14 – 4.00 (m, 2H), 3.76 (s, 3H), 2.77 (d, *J* = 7.6 Hz, 1H), 2.12 (ddd, *J* = 14.5, 8.3, 5.7 Hz, 1H), 1.93 (dt, *J* = 14.5, 5.2 Hz, 1H), 1.35 (s, 3H); **^{13}C NMR** (126 MHz, $CDCl_3$): δ 154.3, 152.7, 144.4, 115.8, 114.8, 112.6, 73.2, 66.3, 55.9, 40.5, 28.8; **IR:** 3468, 2933, 1507, 1466, 1225, 1179, 1036, 920, 824, 730 cm^{-1}; **HRMS(ESI):** Exact mass calculated for $C_{13}H_{18}NaO_3$ [$(M+Na)^+$], 245.1148; found 245.1142; **$[\alpha]_D^{25}$** = +19.4 (c = 1.00, $CHCl_3$).

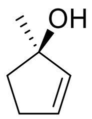

SI-9

(R)-1-Methylcyclopent-2-en-1-ol (SI-9)

SI-9 was prepared from (–)-linalool according to a literature procedure.[6] Crude **SI-9** was purified by Kugelrohr distillation.

TLC: R_f = 0.25 (hexane/EtOAc 10:1, CAM stain); **^{1}H NMR** (400 MHz, $CDCl_3$): δ 5.85 (dt, *J* = 5.6, 2.3 Hz, 1H), 5.73 (dt, *J* = 5.6, 2.2 Hz, 1H), 2.52 (dddt, *J* = 17.0, 8.4, 4.8, 2.3 Hz, 1H), 2.35 (dddt, *J* = 17.0, 8.3, 4.7, 2.3 Hz, 1H), 2.02 – 1.89 (m, 2H), 2.02 – 1.89 (m, 1H), 1.42 (s, 3H); **^{13}C NMR** (101 MHz, $CDCl_3$): δ 138.0, 132.8, 83.6, 39.8, 39.8, 31.2, 27.6; **IR:** 3349, 2965, 2930, 1368, 1176, 1141, 1085, 916, 738, 506 cm^{-1}; **HRMS(EI):** Exact mass calculated for C_6H_9 [$(M–OH)^+$], 81.0699; found 81.0698 ; **$[\alpha]_D^{26}$** = +27.2 (c = 1.00, CH_2Cl_2).

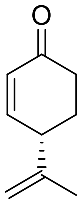

SI-10

(S)-4-(Prop-1-en-2-yl)cyclohex-2-en-1-one (SI-10)

SI-10 was prepared in three steps from (–)-perillaldehyde according to a literature procedure.[7] All spectral data matched the published values.

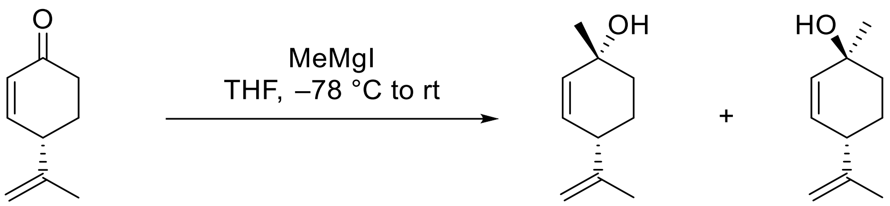

SI-10 **SI-11 minor** **SI-12 major**

14% 21%

(4S)-1-Methyl-4-(prop-1-en-2-yl)cyclohex-2-en-1-ol (SI-11/12). **SI-10** (0.56 g, 4.1 mmol, 1.0, equiv) was dissolved in THF (8 mL) and cooled to –78 °C. 3 M MeMgI in THF (1.8 mL, 5.4 mmol, 1.3 equiv) was added dropwise at –78 °C. The mixture was stirred for 30 min at –78 °C and warmed to rt. Water (10 mL) was added and the mixture was diluted with Et_2O (20 mL). The phases were separated and the aqueous phase extracted once with Et_2O (20 mL). The combined organic extracts were dried over $MgSO_4$ and concentrated under reduced pressure. Purification by column chromatography (hexane/EtOAc 10:1) afforded a colorless oil of **SI-11** (90 mg, 0.58 mmol, 14%) and **SI-12** (130 mg, 0.84 mmol, 21%). All spectral data matched the published values.[7]

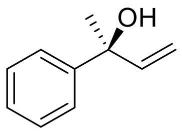

SI-13

(S)-2-Phenylbut-3-en-2-ol (SI-13)

SI-13 was prepared in two steps from (+)-1-phenylethanol according to a literature procedure.[9-10] All spectral data matched the published values.

Preparation of enantioenriched tertiary allylic *tert*-butyl carbonates

General procedure:

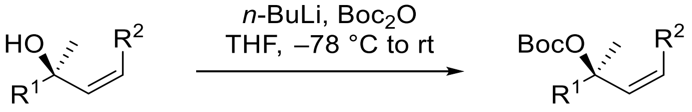

1a, 1g, 1h, 1j, 1l, 1m

Allylic alcohol (27 mmol, 1.0 equiv) was dissolved in anhydrous THF (130 mL) and cooled to –78 °C. 1.6 M *n*-BuLi in hexane (18.6 mL, 29.7 mmol, 1.10 equiv) was added and the solution was stirred for 20 min at –78 °C. Boc_2O (6.5 mL, 28 mmol, 1.1 equiv) was added at –78 °C and the mixture was warmed to rt over 2 h. Water (200 mL) was added and the mixture was diluted with Et_2O (200 mL). The phases were separated and the aqueous phase extracted once with Et_2O (100 mL). The combined organic extracts were dried over $MgSO_4$ and concentrated under reduced pressure. Purification by column chromatography afforded pure products **1a**, **1g**, **1h**, **1j**, **1l**, **1m**.

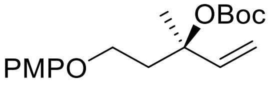

1a

(R)-tert-Butyl (5-(4-methoxyphenoxy)-3-methylpent-1-en-3-yl) carbonate (1a)

Yield: 8.0 g, 25 mmol, 91%; **TLC:** R_f = 0.24 (hexane/EtOAc 20:1, CAM stain, UV); **^{1}H NMR** (400 MHz, $CDCl_3$): δ 6.82 (s, 4H), 6.07 (dd, *J* = 17.6, 11.0 Hz, 1H), 5.29 – 5.13 (m, 2H), 4.08 – 3.95 (m, 2H), 3.76 (s, 3H), 2.32 (t, *J* = 6.9 Hz, 2H), 1.63 (s, 3H), 1.47 (s, 9H); **^{13}C NMR** (101 MHz, $CDCl_3$): δ 154.0, 153.1, 151.9, 141.4, 115.7, 114.8, 114.1, 82.5, 81.8, 64.5, 55.9, 39.0, 28.0, 24.1; **IR:** 2980, 1737, 1508, 1279, 1229, 1115, 1039, 924, 824, 712 cm^{-1}; **HRMS(ESI):** Exact mass calculated for $C_{18}H_{26}NaO_5$ [(M+Na)$^+$], 345.1672; found 345.1668; **SFC:** Daicel Chiralcel OJ-H, 5% MeOH, 2.0 mL/min, 25 °C, >99% ee (T_R (1, major enantiomer) = 4.3 min, T_R (2, minor enantiomer) = 5.1 min); **$[\alpha]_D^{25}$** = +1.7 (c = 1.00, $CHCl_3$).

One-pot procedure for the preparation of **1a** from **SI-7**:

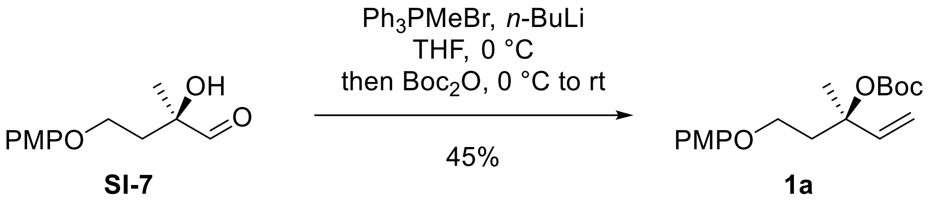

(R)-tert-Butyl (5-(4-methoxyphenoxy)-3-methylpent-1-en-3-yl) carbonate (1a). Methyltriphenylphosphonium bromide (0.57 g, 1.6 mmol, 2.5 equiv) was suspended in anhydrous THF (7 mL) and cooled to 0 °C. 1.6 M *n*-BuLi in hexane (1.0 mL, 1.6 mmol, 2.5 equiv) was added and the orange mixture was stirred for 1 h at 0 °C. A solution of **SI-7** (140 mg, 0.64 mmol, 1.0 equiv) in THF (1 mL) was added at 0 °C and the mixture was stirred for 1 h. Boc_2O (0.18 mL, 0.76 mmol, 1.2 equiv) was added and the mixture was warmed to rt and stirred for 3 h. Water (10 mL) was added and the mixture was diluted with Et_2O (20 mL). The phases were separated and the aqueous phase extracted once with Et_2O (20 mL). The combined organic extracts were dried over $MgSO_4$ and concentrated under reduced pressure. Purification by column chromatography (hexane/EtOAc 20:1) afforded a colorless oil of **1a** (93 mg, 0.28 mmol, 45%).

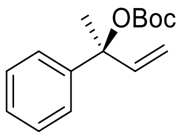

1g

(S)-tert-butyl (2-phenylbut-3-en-2-yl) carbonate (1g)

1g was prepared according to a literature procedure.[11] All spectral data matched the published values.

Note: To prevent decomposition of the product during chromatographic purification, the column was loaded with silica suspended in 5% Et_3N in hexane. Afterwards the column was washed with 2 column volumes of indicated eluent and run without Et_3N as additive.

Yield: 0.75 g, 3.0 mmol, 64%; **TLC:** R_f = 0.33 (hexane/EtOAc 10:1, CAM stain, UV); **^{1}H NMR** (400 MHz, $CDCl_3$): δ 7.41 – 7.37 (m, 2H), 7.36 – 7.30 (m, 2H), 7.28 – 7.23 (m, 1H), 6.35 (dd, *J* = 17.4, 10.9 Hz, 1H), 5.30 (dd, *J* = 7.3, 0.9 Hz, 1H), 5.26 (s, 1H), 1.87 (s, 3H), 1.41 (s, 9H); **IR:** 2981, 1744, 1369, 1277, 1255, 1155, 1099, 850, 762, 699 cm^{-1}; **SFC:** Daicel Chiralcel OJ-H, 1% MeOH, 2.0 mL/min, 25 °C, 98% ee (T_R (1, major enantiomer) = 3.3 min, T_R (2, minor enantiomer) = 3.8 min); **$[\alpha]_D^{28}$** = –14.3 (c = 1.00, $CHCl_3$).

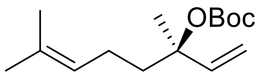

1h

(R)-tert-Butyl (3,7-dimethylocta-1,6-dien-3-yl) carbonate (1h)

Yield: 4.5 g, 18 mmol, 91%; **TLC:** R_f = 0.27 (hexane/EtOAc 40:1, CAM stain, UV); **^{1}H NMR** (400 MHz, $CDCl_3$): δ 6.02 (dd, *J* = 17.6, 11.0 Hz, 1H), 5.18 (dd, *J* = 12.5, 0.8 Hz, 1H), 5.14 (dd, *J* = 5.9, 0.8 Hz, 1H), 5.08 (tp, *J* = 7.1, 1.4 Hz, 1H), 1.99 (m, 2H), 1.83 (ddd, *J* = 10.7, 5.3, 2.6 Hz, 2H), 1.67 (s, 3H), 1.59 (s, 3H), 1.53 (s, 3H), 1.46 (s, 9H); **^{13}C NMR** (101 MHz, $CDCl_3$): δ 152.1, 141.8, 132.0, 123.9, 113.6, 83.7, 81.5, 39.7, 28.0, 25.8, 23.6, 22.5, 17.7; **IR:** 2980, 1738, 1369, 1281, 1265, 1150, 1094, 859, 733, 704 cm^{-1}; **HRMS(ESI):** Exact mass calculated for $C_{15}H_{26}NaO_3$ $[(M+Na)^+]$, 277.1774; found 277.1776; **$[\alpha]_D^{25}$** = +1.3 (c = 1.00, $CHCl_3$).

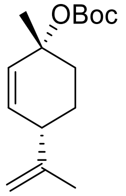

1l

tert-Butyl ((1R,4S)-1-methyl-4-(prop-1-en-2-yl)cyclohex-2-en-1-yl) carbonate (1l)

Yield: 50 mg, 0.20 mmol, 67%; **TLC:** R_f = 0.30 (hexane/EtOAc/Et_3N 100:1:1, CAM stain); **^{1}H NMR** (400 MHz, $CDCl_3$): δ 6.14 (ddd, *J* = 10.2, 2.6, 1.3 Hz, 1H), 5.73 (ddd, *J* = 10.2, 2.7, 0.6 Hz, 1H), 4.77 (s, 1H), 2.74 – 2.62 (m, 1H), 2.26 – 2.12 (m, 1H), 1.77 – 1.69 (m, 5H), 1.50 (m, 14H);

^{13}**C NMR** (101 MHz, $CDCl_3$): δ 152.5, 148.0, 134.0, 130.5, 111.0, 81.3, 78.4, 43.7, 35.2, 28.1, 25.8, 24.3, 20.6; **IR:** 2977, 2937, 1734, 1369, 1286, 1159, 1098, 896, 850, 795 cm^{-1}; **HRMS(ESI):** Exact mass calculated for $C_{15}H_{24}NaO_3$ [$(M+Na)^+$], 275.1618; found 275.1615; $[\alpha]_D^{29}$ = +17.3 (c = 1.00, $CHCl_3$).

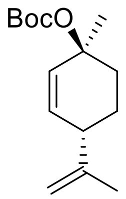

1m

tert-Butyl ((1S,4S)-1-methyl-4-(prop-1-en-2-yl)cyclohex-2-en-1-yl) carbonate (1m)

Yield: 66 mg, 0.26 mmol, 61%; **TLC:** R_f = 0.30 (hexane/EtOAc/Et_3N 100:1:1, CAM stain); ^{1}**H NMR** (400 MHz, $CDCl_3$): δ 6.06 – 5.98 (m, 1H), 5.70 (dd, *J* = 10.2, 3.2 Hz, 1H), 4.78 (t, *J* = 1.7 Hz, 1H), 4.68 – 4.63 (m, 1H), 2.79 (dp, *J* = 6.5, 3.1 Hz, 1H), 2.10 (ddd, *J* = 13.0, 9.6, 3.1 Hz, 1H), 2.01 – 1.91 (m, 1H), 1.83 (dddd, *J* = 12.5, 8.5, 3.0, 0.8 Hz, 1H), 1.73 (d, *J* = 0.7 Hz, 3H), 1.59 – 1.49 (m, 4H), 1.47 (s, 9H); ^{13}**C NMR** (101 MHz, $CDCl_3$): δ 152.3, 147.4, 132.5, 130.9, 111.1, 81.4, 80.1, 42.3, 33.0, 28.1, 26.0, 24.8, 21.4; **IR:** 2977, 2937, 1733, 1368, 1281, 1255, 1166, 1100, 895, 848 cm^{-1}; **HRMS(ESI):** Exact mass calculated for $C_{15}H_{24}NaO_3$ [$(M+Na)^+$], 275.1618; found 275.1617; $[\alpha]_D^{29}$ = –153.3 (c = 1.00, $CHCl_3$).

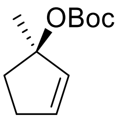

1j

(R)-tert-Butyl (1-methylcyclopent-2-en-1-yl) carbonate (1j)

1j decomposed during purification attempts by distillation or by column chromatography (even with Et_3N as basic additive). Hence, the crude sample was used in the next step without further purification. Crude **1j** had a purity of 76% based on quantitative NMR with phenanthrene as

internal standard. The yield of the subsequent Ru-catalyzed allylic substitution reaction was determined based on this purity.

Acetylation of tertiary allylic alcohol SI-8:

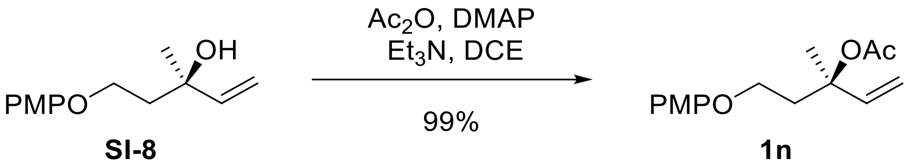

(R)-5-(4-Methoxyphenoxy)-3-methylpent-1-en-3-yl acetate (1n). Allylic alcohol **SI-8** (0.34 g, 1.5 mmol, 1.0 equiv) was dissolved in DCE (5 mL). Triethylamine (0.75 mL, 5.4 mmol, 3.6 equiv), followed by DMAP (49 mg, 40 mmol, 0.27 equiv) and acetic anhydride (0.38 mL, 4.0 mmol, 2.7 equiv) were added and the mixture was stirred for 2 h at rt. Water (10 mL) was added and the mixture was diluted with Et_2O (20 mL). The phases were separated and the aqueous phase extracted once with Et_2O (10 mL). The combined organic extracts were dried over $MgSO_4$ and concentrated under reduced pressure. Purification by column chromatography (hexane/EtOAc 20:1) afforded a colorless oil of **1n** (0.44 g, 1.7 mmol, 99%).

TLC: R_f = 0.21 (hexane/EtOAc 20:1, CAM stain, UV); **^{1}H NMR** (400 MHz, $CDCl_3$): δ 6.82 (d, *J* = 0.6 Hz, 4H), 6.03 (dd, *J* = 17.5, 11.0 Hz, 1H), 5.21 (dd, *J* = 17.5, 0.8 Hz, 1H), 5.16 (dd, *J* = 11.0, 0.8 Hz, 1H), 3.99 (*J* = 7.0, 2H), 3.77 (s, 3H), 2.38 (dt, *J* = 14.0, 6.7 Hz, 1H), 2.26 (dt, *J* = 14.0, 6.7 Hz, 1H), 2.02 (s, 3H), 1.63 (s, 3H); **^{13}C NMR** (101 MHz, $CDCl_3$): δ 170.1, 154.0, 153.0, 141.5, 115.6, 114.8, 113.7, 81.8, 64.5, 55.9, 38.9, 24.4, 22.3; **IR:** 2936, 1735, 1508, 1467, 1368, 1230, 1108, 1040, 928, 826 cm^{-1}; **HRMS(ESI):** Exact mass calculated for $C_{15}H_{20}NaO_4$ [$(M+Na)^+$], 287.1254; found 287.1250; **$[\alpha]_D^{25}$** = –2.8 (c = 1.00, $CHCl_3$).

Diversification of 1a: Preparation of allylic carbonates 1b-1f, 1k

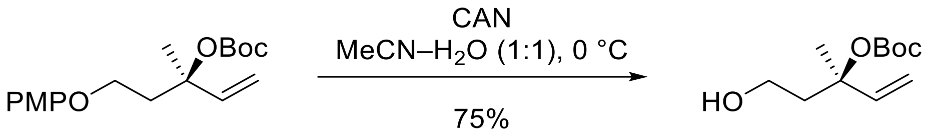

1a **SI-14**

(R)-tert-Butyl (5-hydroxy-3-methylpent-1-en-3-yl) carbonate (SI-14). **1a** (2.0 g, 6.2 mmol, 1.0 equiv) was dissolved in MeCN (24 mL) and water (24 mL) was added. The milky mixture was cooled to 0 °C and ceric ammonium nitrate (CAN, 7.8 g, 17 mmol, 2.7 equiv) was added. The reaction was stirred for 1 h at 0 °C and diluted with brine (200 mL) and EtOAc (200 mL). The phases were separated and the aqueous phase extracted once with EtOAc (100 mL). The combined organic extracts were dried over $MgSO_4$ and concentrated under reduced pressure. Purification by column chromatography (toluene/EtOAc 10:1) afforded a colorless oil of **SI-14** (1.0 g, 160 mmol, 75%).

TLC: R_f = 0.31 (toluene/EtOAc 10:1, CAM stain); **^{1}H NMR** (400 MHz, $CDCl_3$): δ 6.02 (dd, *J* = 17.6, 11.0 Hz, 1H), 5.18 (dd, *J* = 14.2, 0.7 Hz, 1H), 5.15 (dd, *J* = 7.6, 0.7 Hz, 1H), 3.74 (t, *J* = 6.5 Hz, 2H), 2.07 (td, *J* = 6.6, 3.0 Hz, 2H), 1.87 (s, 1H), 1.58 (s, 3H), 1.44 (s, 9H); **^{13}C NMR** (101 MHz, $CDCl_3$): δ 151.8, 141.5, 113.9, 83.1, 81.8, 58.8, 42.5, 27.9, 24.0; **IR:** 3378, 2981, 1740, 1370, 1281, 1158, 1107, 1045, 857, 794 cm^{-1}; **HRMS(ESI):** Exact mass calculated for $C_{11}H_{20}NaO_4$ [$(M+Na)^+$], 239.1254; found 239.1254; **$[\alpha]_D^{25}$** = –3.9 (c = 1.00, $CHCl_3$).

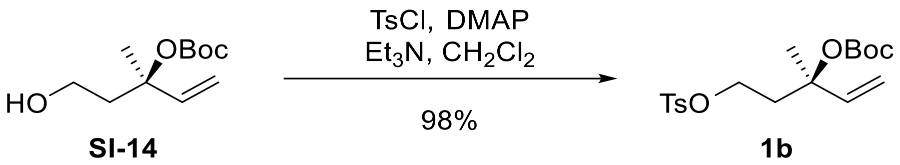

(R)-3-((tert-Butoxycarbonyl)oxy)-3-methylpent-4-en-1-yl 4-methylbenzenesulfonate (1b). Primary alcohol **SI-14** (95 mg, 0.44 mol, 1.0 equiv) was dissolved in CH_2Cl_2 (1.5 mL) and Et_3N (0.13 mL, 0.92 mmol, 2.1 equiv), tosyl chloride (130 mg, 0.69 mmol, 1.6 equiv) and DMAP (11 mg, 0.092 mmol, 0.21 equiv) were added. The reaction was stirred for 2 h at rt and diluted with water (10 mL) and Et_2O (10 mL). The phases were separated and the aqueous phase extracted once with Et_2O (10 mL). The combined organic extracts were dried over $MgSO_4$ and concentrated under reduced pressure. Purification by column chromatography (hexane/EtOAc 10:1) afforded a colorless oil of **1b** (160 mg, 0.43 mmol, 98%).

TLC: R_f = 0.28 (hexane/EtOAc 10:1, CAM stain, UV); **^{1}H NMR** (400 MHz, $CDCl_3$): δ 7.79 (d, *J* = 8.3 Hz, 2H), 7.34 (dd, *J* = 8.7, 0.7 Hz, 2H), 5.90 (dd, *J* = 17.5, 11.1 Hz, 1H), 5.14 (d, *J* = 2.1 Hz, 1H), 5.10 (dd, *J* = 9.0, 0.6 Hz, 1H), 4.11 (ddd, *J* = 7.2, 6.6, 1.3 Hz, 2H), 2.45 (s, 3H), 2.30 – 2.12 (m, 2H), 1.52 (s, 3H), 1.43 (s, 9H); **^{13}C NMR** (101 MHz, $CDCl_3$): δ 151.7, 144.9, 140.5, 133.2, 130.0, 128.1, 114.6, 82.1, 81.6, 66.5, 38.3, 27.9, 24.1, 21.8; **IR:** 2981, 1740, 1367, 1282, 1177, 1120, 967, 816, 664, 555 cm^{-1}; **HRMS(ESI):** Exact mass calculated for $C_{18}H_{26}NaO_6S$ [$(M+Na)^+$], 393.1342; found 393.1335; **$[\alpha]_D^{25}$** = +1.2 (c = 1.00, $CHCl_3$).

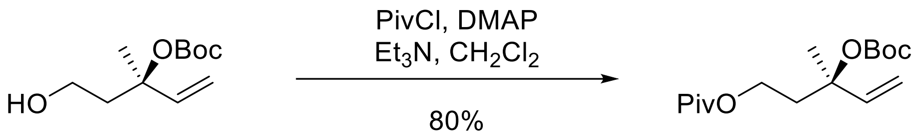

SI-14 **1c**

(R)-3-((tert-Butoxycarbonyl)oxy)-3-methylpent-4-en-1-yl pivalate (1c). Primary alcohol **SI-14** (79 mg, 0.36 mmol, 1.0 equiv) was dissolved in CH_2Cl_2 (2 mL). Triethylamine (0.10 mL, 0.74 mmol, 2.0 equiv), DMAP (9 mg, 0.07 mmol, 0.2 equiv) and pivaloyl chloride (0.07 mL, 0.6 mmol, 2 equiv) were added and the reaction was stirred for 2 h at rt. Sat. aq. $NaHCO_3$ (5 mL) and Et_2O (10 mL) were added. The phases were separated and the aqueous phase extracted once with Et_2O (10 mL). The combined organic extracts were dried over $MgSO_4$ and concentrated under reduced pressure. Purification by column chromatography (hexane/EtOAc 20:1) afforded a colorless oil of **1c** (87 mg, 0.29 mmol, 80%).

TLC: R_f = 0.37 (hexane/EtOAc 20:1, CAM stain); **^{1}H NMR** (400 MHz, $CDCl_3$): δ 6.02 (ddd, J = 17.5, 11.0, 1.0 Hz, 1H), 5.20 (d, J = 12.8 Hz, 1H), 5.17 (d, J = 6.2 Hz, 1H), 4.24 – 4.07 (m, 2H), 2.15 (t, J = 6.9 Hz, 2H), 1.59 (m, 3H), 1.45 (s, 9H), 1.17 (s, 9H); **^{13}C NMR** (101 MHz, $CDCl_3$): δ 178.6, 151.9, 141.1, 114.2, 82.2, 81.8, 60.5, 38.7, 28.0, 27.3, 23.7; **IR:** 2979, 1731, 1481, 1369, 1280, 1253, 1148, 1119, 855, 794 cm^{-1}; **HRMS(ESI):** Exact mass calculated for $C_{16}H_{28}NaO_5$ [$(M+Na)^+$], 323.1829; found 323.1834; **$[\alpha]_D^{25}$** = –3.9 (c = 1.00, $CHCl_3$).

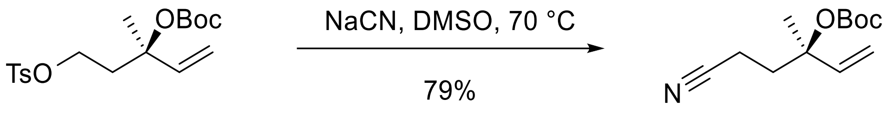

1b **1d**

(R)-tert-Butyl (5-cyano-3-methylpent-1-en-3-yl) carbonate (1d). Tosylate **1b** (110 mg, 0.30 mmol, 1.0 equiv) was dissolved in DMSO (0.8 mL). Sodium cyanide (29 mg, 0.60 mmol, 2.0 equiv) was added and the reaction was stirred for 0.75 h at 70 °C. Sat. aq. $NaHCO_3$ (5 mL) and Et_2O (10 mL) were added. The phases were separated and the aqueous phase extracted once with Et_2O (10 mL). The combined organic extracts were dried over $MgSO_4$ and concentrated under reduced pressure. Purification by column chromatography (hexane/EtOAc 10:1) afforded a colorless oil of **1d** (56 mg, 0.23 mmol, 79%).

TLC: R_f = 0.31 (hexane/EtOAc 10:1, CAM stain, UV); **^{1}H NMR** (400 MHz, $CDCl_3$): δ 5.92 (dd, *J* = 17.5, 11.0 Hz, 1H), 5.25 (d, *J* = 6.4 Hz, 1H), 5.21 (d, *J* = 12.8 Hz, 1H), 2.43 – 2.37 (m, 2H), 2.31 – 2.19 (m, 1H), 2.09 (ddd, *J* = 13.9, 8.8, 6.9 Hz, 1H), 1.59 (s, 3H), 1.46 (s, 9H); **^{13}C NMR** (101 MHz, $CDCl_3$): δ 151.6, 139.8, 119.6, 115.3, 82.3, 81.7, 35.7, 27.9, 23.5, 12.1; **IR:** 2982, 1739, 1370, 1283, 1256, 1159, 1104, 931, 854, 823 cm^{-1}; **HRMS(ESI):** Exact mass calculated for $C_{12}H_{23}N_2O_3$ [$(M+H)^+$], 243.1703; found 243.1700; **$[\alpha]_D^{25}$** = –0.5 (c = 1.00, $CHCl_3$).

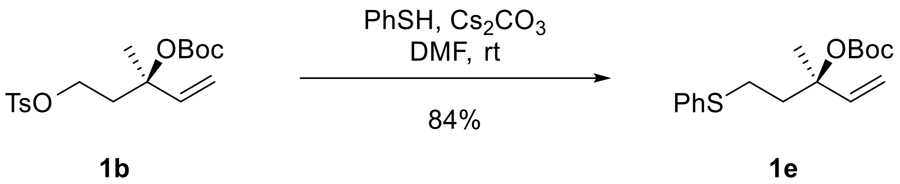

(R)-tert-Butyl (3-methyl-5-(phenylthio)pent-1-en-3-yl) carbonate (1e). Tosylate **1b** (110 mg, 0.30 mmol, 1.0 equiv) was dissolved in DMF (0.8 mL). Cesium carbonate (230 mg, 0.81 mmol, 2.4 equiv) and thiophenol (0.05 mL, 0.5 mmol, 2 equiv) were added and the reaction was stirred for 0.5 h at rt. Water (5 mL) and Et_2O (10 mL) were added. The phases were separated and the aqueous phase extracted once with Et_2O (10 mL). The combined organic extracts were dried over $MgSO_4$ and concentrated under reduced pressure. Purification by column chromatography (hexane/EtOAc 25:1) afforded a colorless oil of **1e** (77 mg, 0.25 mmol, 84%).

TLC: R_f = 0.34 (hexane/EtOAc 25:1, CAM stain, UV); **^{1}H NMR** (400 MHz, $CDCl_3$): δ 7.34 – 7.24 (m, 4H), 7.21 – 7.12 (m, 1H), 6.08 – 5.92 (m, 1H), 5.20 (s, 1H), 5.16 (dd, *J* = 6.3, 0.7 Hz, 1H), 2.97 – 2.88 (m, 2H), 2.26 – 2.03 (m, 2H), 1.57 (s, 3H), 1.47 (s, 9H); **^{13}C NMR** (101 MHz, $CDCl_3$): δ 151.9, 141.0, 136.4, 129.1, 129.0, 126.0, 114.3, 82.9, 81.8, 39.9, 27.9, 27.9, 23.6; **IR:** 2980, 1740, 1369, 1279, 1256, 1150, 1094, 855, 738, 691 cm^{-1}; **HRMS(ESI):** Exact mass calculated for $C_{17}H_{24}NaO_3S$ $[(M+Na)^+]$, 331.1338; found 331.1340; **$[\alpha]_D^{25}$** = +7.5 (c = 1.00, $CHCl_3$).

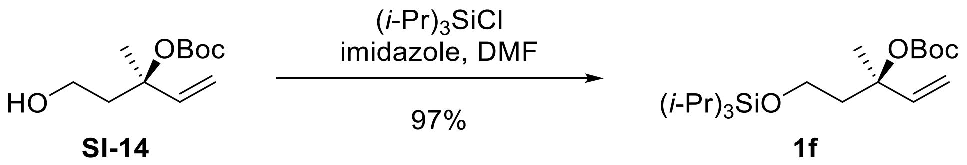

(R)-tert-Butyl (3-methyl-5-((triisopropylsilyl)oxy)pent-1-en-3-yl) carbonate (1f). Primary alcohol **SI-14** (79 mg, 0.36 mmol, 1.0 equiv) was dissolved in DMF (0.8 mL). Imidazole (50 mg, 0.74 mmol, 2.0 equiv) and triisopropylsilyl chloride (0.12 mL, 0.55 mmol, 1.5 equiv) were added and the reaction was stirred for 2 h at rt. Sat. aq. $NaHCO_3$ (5 mL) and Et_2O (10 mL) were added. The phases were separated and the aqueous phase extracted once with Et_2O (10 mL). The combined organic extracts were dried over $MgSO_4$ and concentrated under reduced pressure. Purification by column chromatography (hexane/EtOAc 40:1) afforded a colorless oil of **1f** (130 mg, 0.35 mmol, 97%).

TLC: R_f = 0.33 (hexane/EtOAc 40:1, CAM stain); **^{1}H NMR** (400 MHz, $CDCl_3$): δ 6.04 (dd, *J* = 17.5, 11.0 Hz, 1H), 5.18 (dd, *J* = 17.6, 0.8 Hz, 1H), 5.14 (dd, *J* = 11.0, 0.7 Hz, 1H), 3.82 – 3.71 (m, 2H), 2.24 – 2.04 (m, 2H), 1.58 (s, 3H), 1.46 (s, 9H), 1.08 – 1.02 (m, 21H); **^{13}C NMR** (101 MHz, $CDCl_3$): δ 151.9, 141.8, 113.6, 82.9, 81.6, 59.3, 42.5, 28.0, 24.1, 18.2, 12.1; **IR:** 2943, 2867, 1740, 1368, 1281, 1248, 1156, 1097, 882, 680 cm^{-1}; **HRMS(ESI):** Exact mass calculated for $C_{20}H_{40}NaO_4Si$ $[(M+Na)^+]$, 395.2588; found 395.2594; **$[\alpha]_D^{25}$** = –2.6 (c = 1.00, $CHCl_3$).

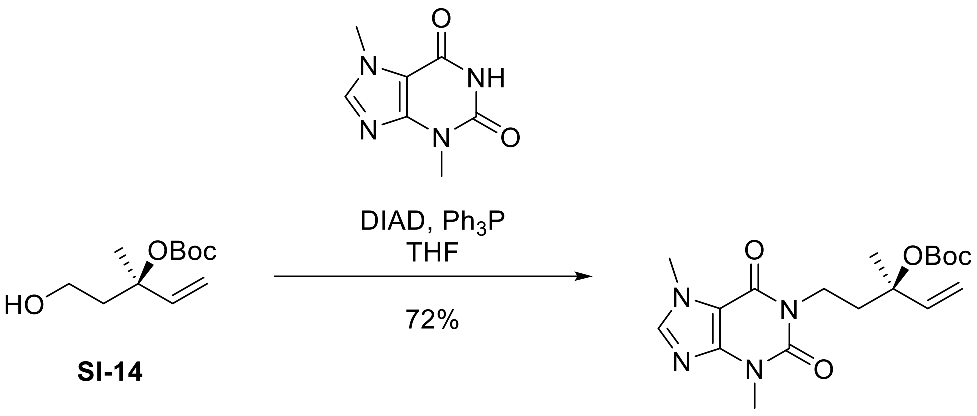

(R)-tert-Butyl (5-(3,7-dimethyl-2,6-dioxo-2,3,6,7-tetrahydro-1H-purin-1-yl)-3-methylpent-1-en-3-yl) carbonate (1k). Primary alcohol **SI-14** (80 mg, 0.37 mmol, 1.0 equiv) was dissolved in THF (3.5 mL). Triphenylphosphine (116 mg, 0.44 mmol, 1.2 equiv), theobromine (80 mg, 0.44 mmol, 1.2 equiv) and diisopropyl azodicarboxylate (0.09 mL, 0.44 mmol, 1.2 equiv) were added and the reaction was stirred for 24 h at rt. The mixture was diluted with water (5 mL) and EtOAc (5 mL). The phases were separated and the aqueous phase extracted once with EtOAc (5 mL). The combined organic extracts were dried over $MgSO_4$ and concentrated under reduced pressure. Purification by column chromatography (Et_2O/MeOH 50:1) afforded a colorless oil of **1k** (100 mg, 0.27 mmol, 72%).

TLC: R_f = 0.33 (Et_2O/MeOH 50:1, CAM stain); **^{1}H NMR** (400 MHz, $CDCl_3$): δ 7.47 (d, *J* = 0.7 Hz, 1H), 6.08 (dd, *J* = 17.6, 11.0 Hz, 1H), 5.24 (dd, *J* = 17.6, 0.7 Hz, 1H), 5.18 (dd, *J* = 11.0, 0.7 Hz, 1H), 4.09 – 4.01 (m, 2H), 3.95 (d, *J* = 0.7 Hz, 3H), 3.53 (s, 3H), 2.15 (ddd, *J* = 8.0, 7.0, 4.7 Hz, 2H), 1.61 (s, 3H), 1.43 (s, 9H); **^{13}C NMR** (101 MHz, $CDCl_3$): δ 155.1, 151.9, 151.4, 148.9, 141.4, 140.9, 114.2, 107.7, 82.4, 81.5, 37.1, 37.1, 33.6, 29.7, 27.9, 23.6; **IR:** 2980, 1740, 1705, 1659, 1550, 1368, 1394, 1254, 1144, 763 cm^{-1}; **HRMS(ESI):** Exact mass calculated for $C_{18}H_{26}KN_4O_5$ $[(M+K)^+]$, 417.1535; found 417.1534; **$[\alpha]_D^{25}$** = +1.9 (c = 1.00, $CHCl_3$).

Boc protection of (–)-sclareol

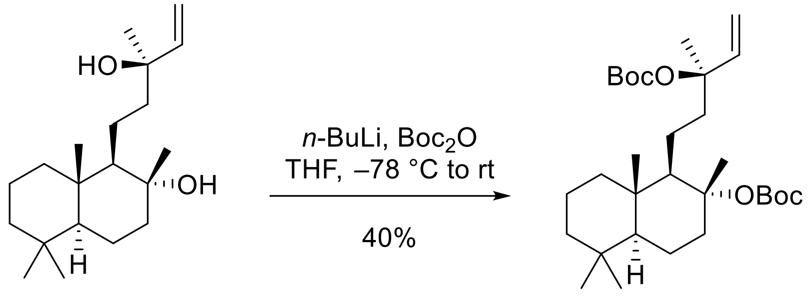

(–)-sclareol **1i**

(1R,2R,4aS,8aS)-1-((R)-3-((tert-Butoxycarbonyl)oxy)-3-methylpent-4-en-1-yl)-2,5,5,8a-tetramethyldecahydronaphthalen-2-yl tert-butyl carbonate (1i). (–)-Sclareol (0.50 g, 1.6 mmol, 1.0 equiv) was dissolved in THF (8 mL). The solution was cooled to –78 °C and a 1.6 M solution of *n*-BuLi in hexane (2.3 mL, 3.7 mmol, 2.3 equiv) was added and the solution was stirred for 20 min at –78 °C. Boc_2O (0.78 mL, 3.4 mmol, 2.1 equiv) was added at –78 °C. The mixture was warmed to rt and stirred for 2 h. Water (20 mL) was added and the mixture was diluted with Et_2O (20 mL). The phases were separated and the aqueous phase extracted once with Et_2O (10 mL). The combined organic extracts were dried over $MgSO_4$ and concentrated under reduced pressure. Purification by column chromatography (hexane/EtOAc 40:1) afforded a colorless oil of **1i** (0.33 g, 0.65 mmol, 40%).

TLC: R_f = 0.20 (hexane/EtOAc 40:1, CAM stain); **^{1}H NMR** (400 MHz, $CDCl_3$): δ 5.95 (dd, *J* = 17.6, 11.1 Hz, 1H), 5.21 (dd, *J* = 17.5, 1.0 Hz, 1H), 5.13 (dd, *J* = 11.1, 0.9 Hz, 1H), 2.22 (dt, *J* = 12.4, 3.5 Hz, 1H), 2.17 – 2.08 (m, 1H), 2.04 (dd, *J* = 13.2, 4.8 Hz, 1H), 1.89 – 1.77 (m, 2H), 1.65 (tt, *J* = 15.3, 2.3 Hz, 2H), 1.57 – 1.48 (m, 4H), 1.46 (s, 9H), 1.45 – 1.42 (m, 10H), 1.42 – 1.30 (m, 5H), 1.32 – 1.19 (m, 2H), 1.14 (td, *J* = 13.3, 4.1 Hz, 1H), 1.05 – 0.92 (m, 2H), 0.85 (s, 3H), 0.80 (s, 3H), 0.76 (s, 3H); **^{13}C NMR** (101 MHz, $CDCl_3$): δ 152.1, 152.0, 141.7, 113.7, 88.8, 84.3, 81.3, 80.7, 56.5, 55.5, 42.0, 41.0, 39.7, 39.7, 37.8, 33.4, 33.3, 28.1, 28.0, 24.3, 22.0, 21.6, 20.3, 19.6, 18.4, 15.8; **IR:** 2933, 1734, 1459, 1368, 1279, 1163, 1124, 1081, 838, 794 cm^{-1}; **HRMS(ESI):** Exact mass calculated for $C_{30}H_{52}NaO_6$ $[(M+Na)^+]$, 531.3656; found 531.3644; **$[\alpha]_D^{25}$** = –20.0 (c = 1.00, $CHCl_3$).

5. Preparation of Ligands

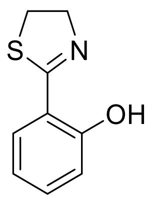

L1

2-(4,5-Dihydrothiazol-2-yl)phenol (L1)

L1 was prepared according to a literature procedure. All spectral data matched the published values.[12]

TLC: R_f = 0.30 (hexane/EtOAc 20:1, CAM stain, UV); **^{1}H NMR** (400 MHz, $CDCl_3$): δ 12.66 (s, 1H), 7.42 (dd, *J* = 7.8, 1.6 Hz, 1H), 7.34 (ddd, *J* = 8.7, 7.3, 1.7 Hz, 1H), 7.04 – 6.94 (m, 1H), 6.96 – 6.78 (m, 1H), 4.48 (t, *J* = 8.3 Hz, 2H), 3.36 (t, *J* = 8.3 Hz, 2H); **^{13}C NMR** (101 MHz, $CDCl_3$): δ 172.5, 159.2, 133.0, 130.8, 118.9, 117.1, 116.5, 63.5, 32.0; **IR:** 2853, 1590, 1487, 1310, 1220, 1012, 935, 816, 747, 666 cm^{-1}; **HRMS(ESI):** Exact mass calculated for $C_9H_{10}NOS$ [$(M+H)^+$], 180.0478; found 180.0477.

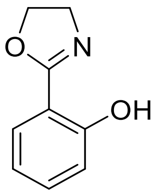

L2

2-(4,5-Dihydrooxazol-2-yl)phenol (L2)

L2 was prepared according to a literature procedure. All spectral data matched the published values.[12]

TLC: R_f = 0.26 (hexane/EtOAc 20:1, CAM stain, UV); **^{1}H NMR** (500 MHz, $CDCl_3$): δ 12.16 (s, 1H), 7.65 (dd, *J* = 7.8, 1.8 Hz, 1H), 7.37 (ddd, *J* = 8.3, 7.2, 1.7 Hz, 1H), 7.01 (ddd, *J* = 8.3, 1.1, 0.4 Hz, 1H), 6.87 (ddd, *J* = 7.7, 7.2, 1.1 Hz, 1H), 4.43 (td, *J* = 9.4, 0.6 Hz, 2H), 4.11 (t, *J* = 9.6 Hz,

2H); ^{13}**C NMR** (126 MHz, $CDCl_3$): δ 166.3, 159.9, 133.4, 128.1, 118.7, 116.8, 110.9, 66.9, 53.6; **IR:** 2883, 1639, 1490, 1367, 1259, 1232, 1155, 1064, 941, 752 cm^{-1}; **HRMS(ESI):** Exact mass calculated for $C_9H_{10}NO_2$ [$(M+H)^+$], 164.0706; found 164.0709.

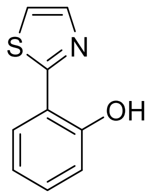

L3

2-(Thiazol-2-yl)phenol (L3)

L3 was prepared according to a literature procedure. All spectral data matched the published values.[13]

TLC: R_f = 0.37 (hexane/EtOAc 20:1, CAM stain, UV); ^{1}**H NMR** (400 MHz, $CDCl_3$): δ 12.30 (s, 1H), 7.80 (d, *J* = 3.4 Hz, 1H), 7.65 (dd, *J* = 7.8, 1.6 Hz, 1H), 7.36 – 7.26 (m, 2H), 7.07 (ddd, *J* = 8.4, 1.2, 0.5 Hz, 1H), 6.91 (ddd, *J* = 7.8, 7.3, 1.2 Hz, 1H); ^{13}**C NMR** (101 MHz, $CDCl_3$): δ 169.6, 157.1, 141.4, 131.9, 127.5, 119.5, 117.9, 117.2, 117.0; **IR:** 3120, 1620, 1581, 1488, 1397, 1302, 1224, 1144, 823, 747 cm^{-1}; **HRMS(ESI):** Exact mass calculated for C_9H_8NOS [$(M+H)^+$], 178.0321; found 178.0320.

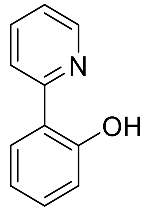

L5

2-(Pyridin-2-yl)phenol (L5)

L5 was prepared according to a literature procedure. All spectral data matched the published values.[14]

TLC: R_f = 0.25 (hexane/EtOAc 20:1, CAM stain, UV); **^{1}H NMR** (400 MHz, $CDCl_3$): δ 8.52 (d, *J* = 5.1 Hz, 0H), 7.93 (d, *J* = 8.3 Hz, 1H), 7.85 (ddd, *J* = 7.6, 1.9, 0.7 Hz, 1H), 7.81 (dd, *J* = 8.0, 1.6 Hz, 1H), 7.31 (ddd, *J* = 8.2, 7.2, 1.6 Hz, 1H), 7.24 (m, 1H), 7.03 (dd, *J* = 8.3, 1.3 Hz, 1H), 6.91 (ddd, *J* = 8.0, 7.2, 1.3 Hz, 1H); **^{13}C NMR** (101 MHz, $CDCl_3$): δ 160.2, 158.1, 146.0, 137.9, 131.6, 126.2, 121.6, 119.2, 118.9, 118.9, 118.8; **IR:** 3056, 2690, 1592, 1476, 1429, 1303, 1269, 833, 748, 725 cm^{-1}; **HRMS(ESI):** Exact mass calculated for $C_{11}H_{10}NO$ [$(M+H)^+$], 172.0757; found 172.0755.

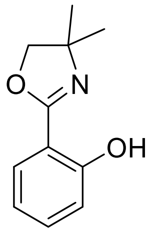

L6

2-(4,4-Dimethyl-4,5-dihydrooxazol-2-yl)phenol (L6)

L6 was prepared according to a literature procedure. All spectral data matched the published values.[15]

TLC: R_f = 0.42 (hexane/EtOAc 20:1, CAM stain, UV); **^{1}H NMR** (500 MHz, $CDCl_3$): δ 12.20 (s, 1H), 7.64 (ddd, *J* = 7.9, 1.8, 0.4 Hz, 1H), 7.36 (ddd, *J* = 8.3, 7.3, 1.8 Hz, 1H), 7.00 (ddd, *J* = 8.3, 1.2, 0.5 Hz, 1H), 6.87 (ddd, *J* = 7.8, 7.3, 1.1 Hz, 1H), 4.10 (s, 2H), 1.40 (s, 6H); **^{13}C NMR** (126 MHz, $CDCl_3$): δ 163.6, 160.0, 133.3, 128.0, 118.7, 116.8, 111.0, 78.5, 67.2, 28.6; **IR:** 2970, 1641, 1618, 1492, 1361, 1260, 1063, 963, 755, 691 cm^{-1}; **HRMS(ESI):** Exact mass calculated for $C_{11}H_{14}NO_2$ [$(M+H)^+$], 192.1019; found 192.1021.

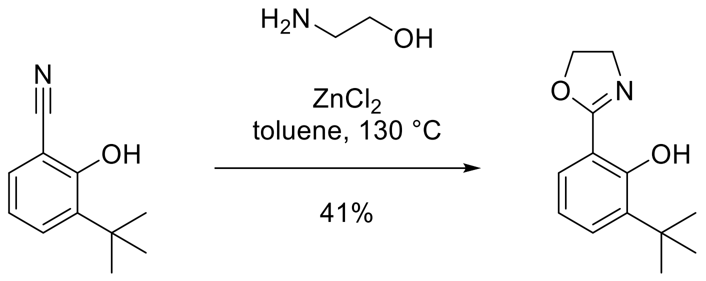

L7

2-(tert-Butyl)-6-(4,5-dihydrooxazol-2-yl)phenol (L7) 3-(tert-butyl)-2-hydroxybenzonitrile (0.29 g, 1.7 mmol, 1.0 equiv), ethanolamine (0.30 mL, 4.9 mmol, 3.0 equiv), and $ZnCl_2$ (21 mg, 0.16 mmol, 9 mol %) were added to toluene (3.3 mL) and the mixture was stirred for 20 h at 130 °C. The reaction was cooled to rt, water (10 mL) was added and the mixture was diluted with EtOAc (20 mL). The phases were separated and the aqueous phase extracted once with EtOAc (10 mL). The combined organic extracts were dried over $MgSO_4$ and concentrated under reduced pressure. Purification by column chromatography (hexane/EtOAc 40:1) afforded a white solid of **L7** (0.15 g, 0.68 mmol, 41%).

TLC: R_f = 0.44 (hexane/EtOAc 20:1, CAM stain, UV); **^{1}H NMR** (400 MHz, $CDCl_3$): δ 12.73 (s, 1H), 7.55 (dd, *J* = 7.8, 1.7 Hz, 1H), 7.38 (dd, *J* = 7.8, 1.7 Hz, 1H), 6.80 (t, *J* = 7.7 Hz, 1H), 4.41 (td, *J* = 9.4, 0.8 Hz, 2H), 4.11 (ddd, *J* = 9.9, 9.3, 0.7 Hz, 2H), 1.43 (s, 9H); **^{13}C NMR** (101 MHz, $CDCl_3$): δ 167.1, 159.2, 137.3, 130.5, 126.2, 117.9, 110.9, 66.7, 53.6, 35.1, 29.5; **IR:** 2958, 1636, 1431, 1371, 1252, 1211, 1155, 956, 829, 745 cm^{-1}; **HRMS(ESI):** Exact mass calculated for $C_{13}H_{18}NaO_2$ [$(M+H)^+$], 220.1332; found 220.1332.

6. NMR Spectra

^{1}H NMR (400 MHz, $CDCl_3$) of **3a**

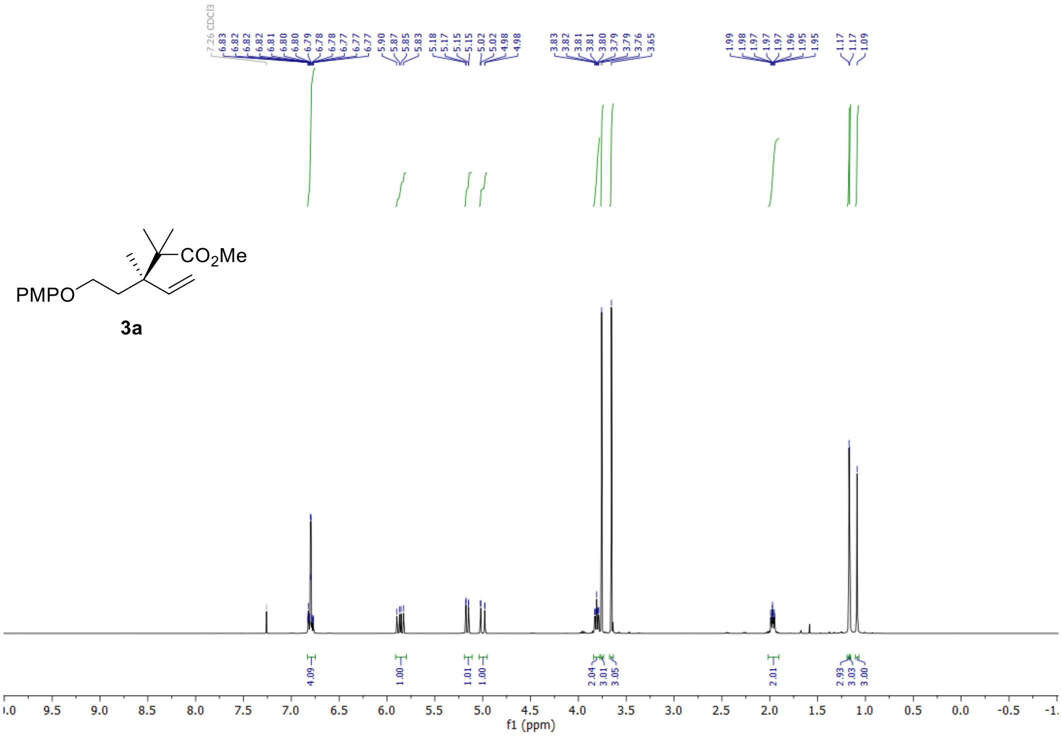

^{13}C NMR (101 MHz, $CDCl_3$) of **3a**

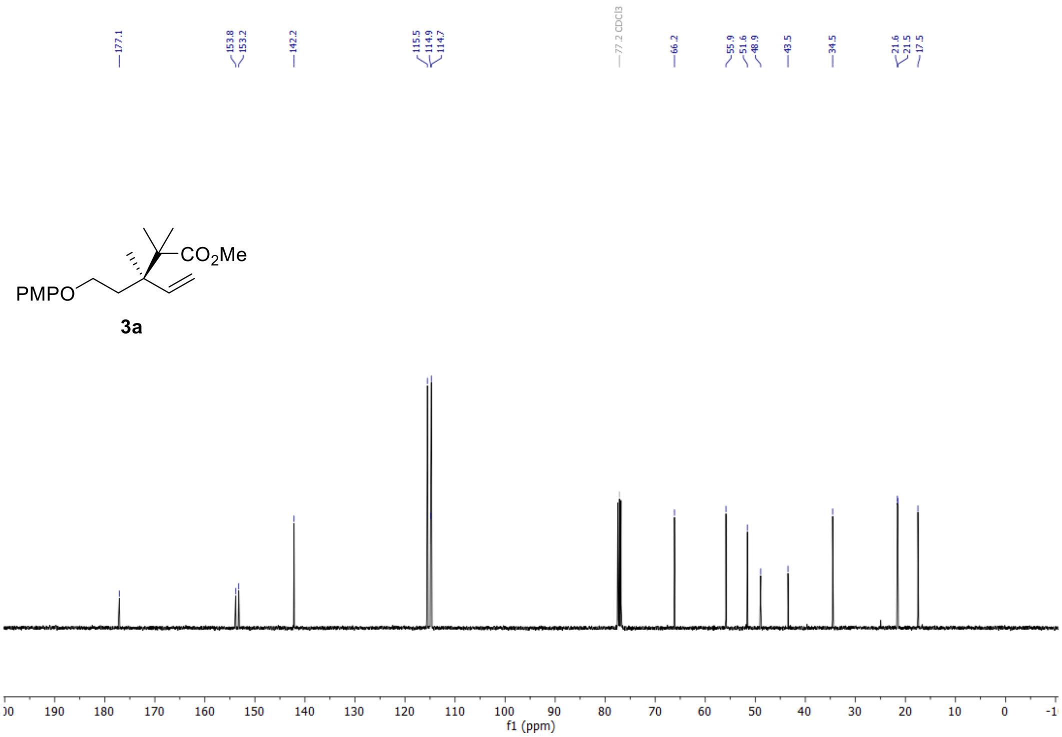

^{1}H NMR (400 MHz, $CDCl_3$) of **3b**

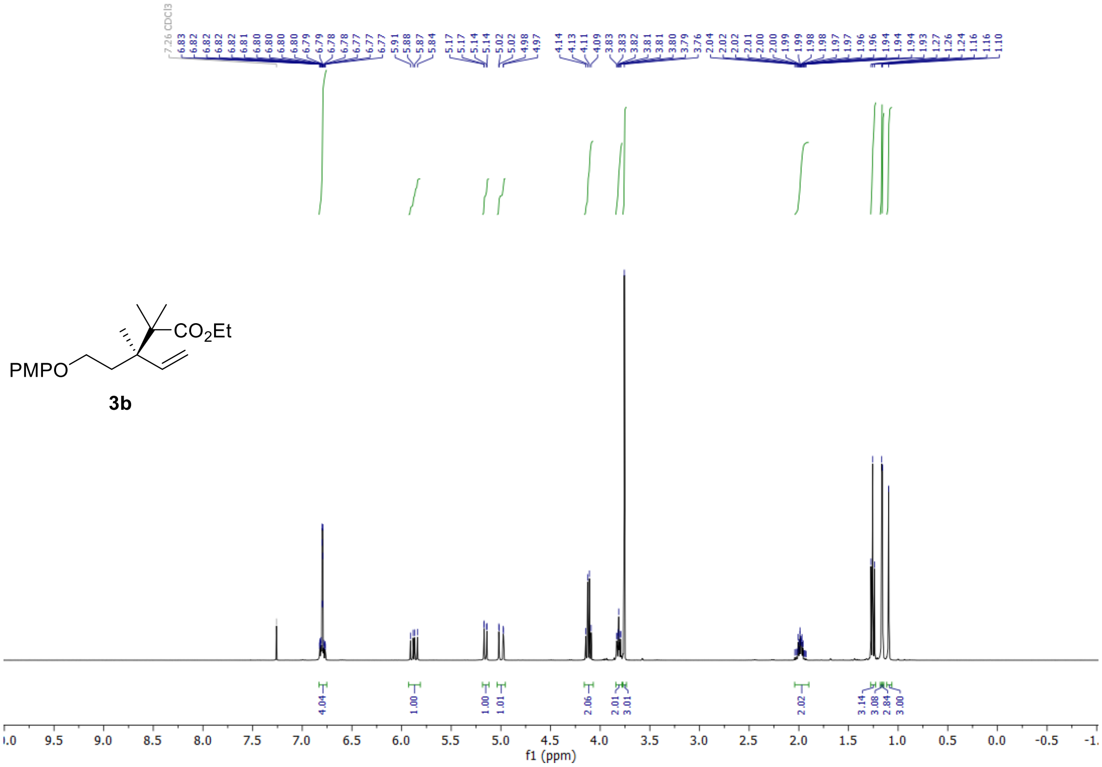

^{13}C NMR (101 MHz, $CDCl_3$) of **3b**

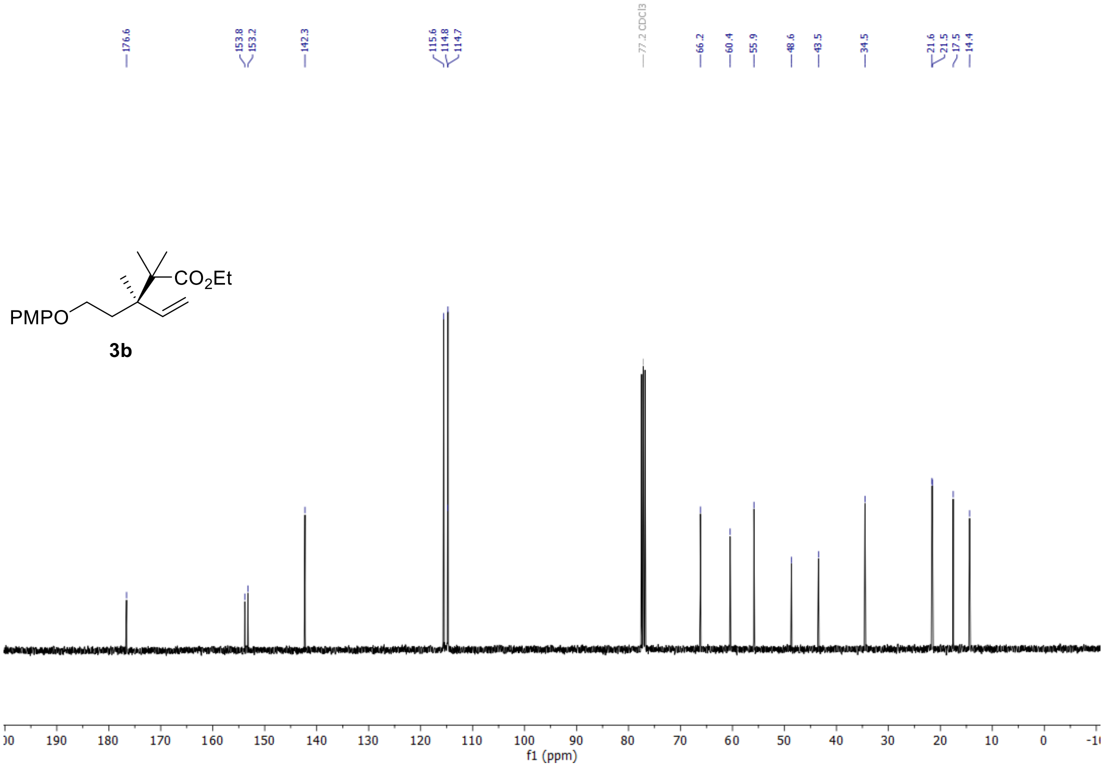

^{1}H NMR (400 MHz, $CDCl_3$) of **3c**

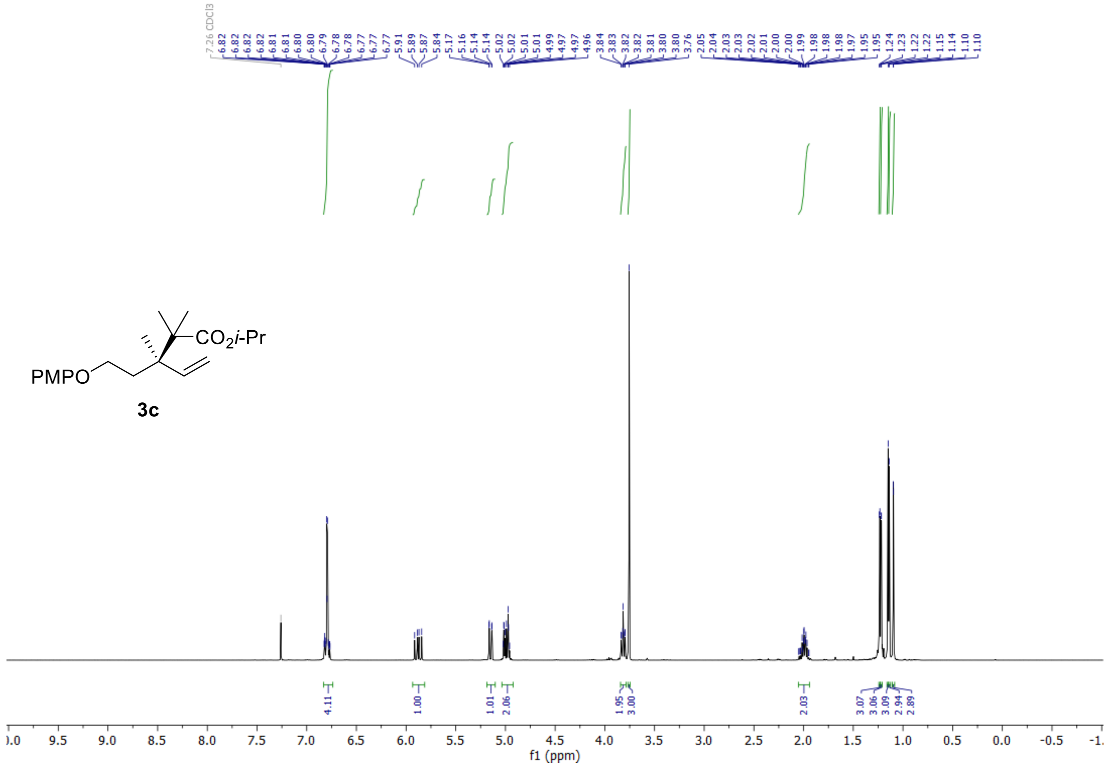

^{13}C NMR (101 MHz, $CDCl_3$) of **3c**

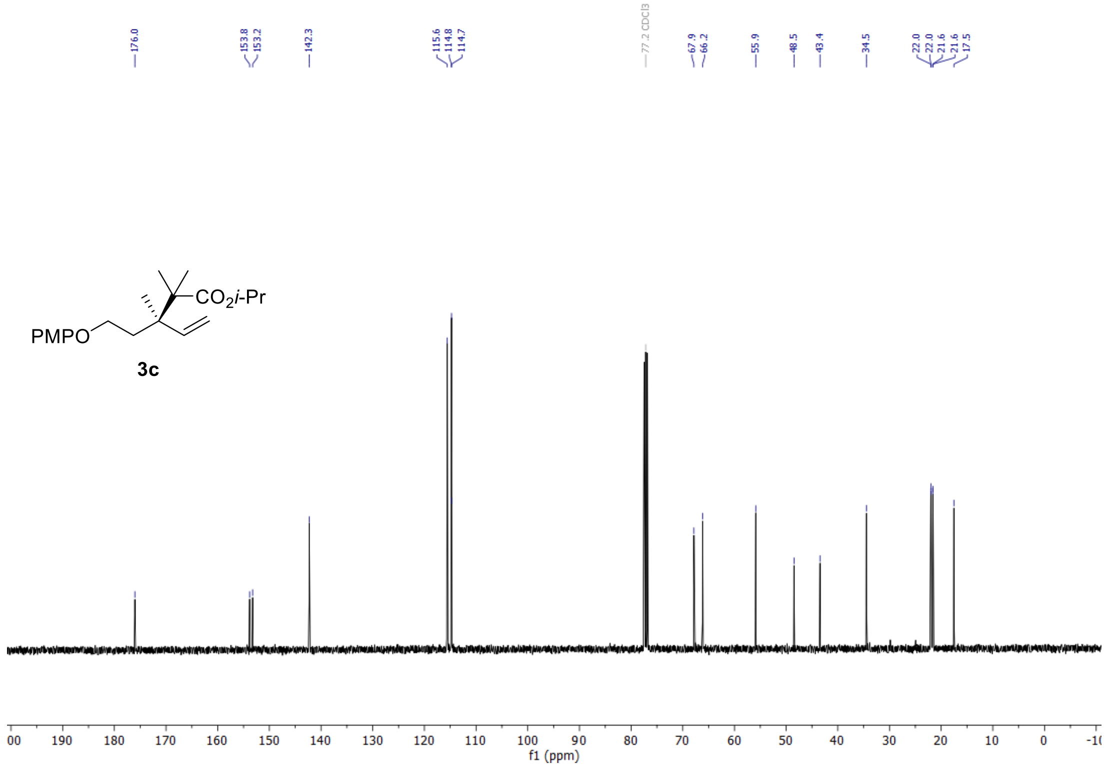

^{1}H NMR (400 MHz, $CDCl_3$) of **3d**

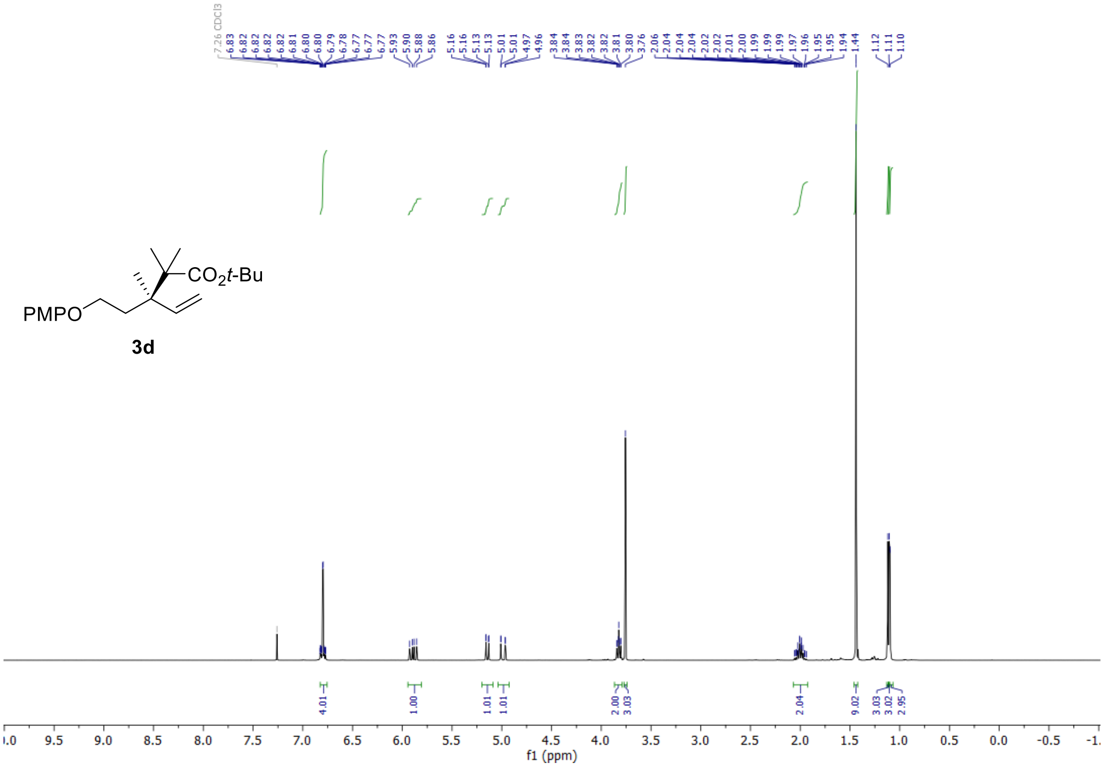

^{13}C NMR (101 MHz, $CDCl_3$) of **3d**

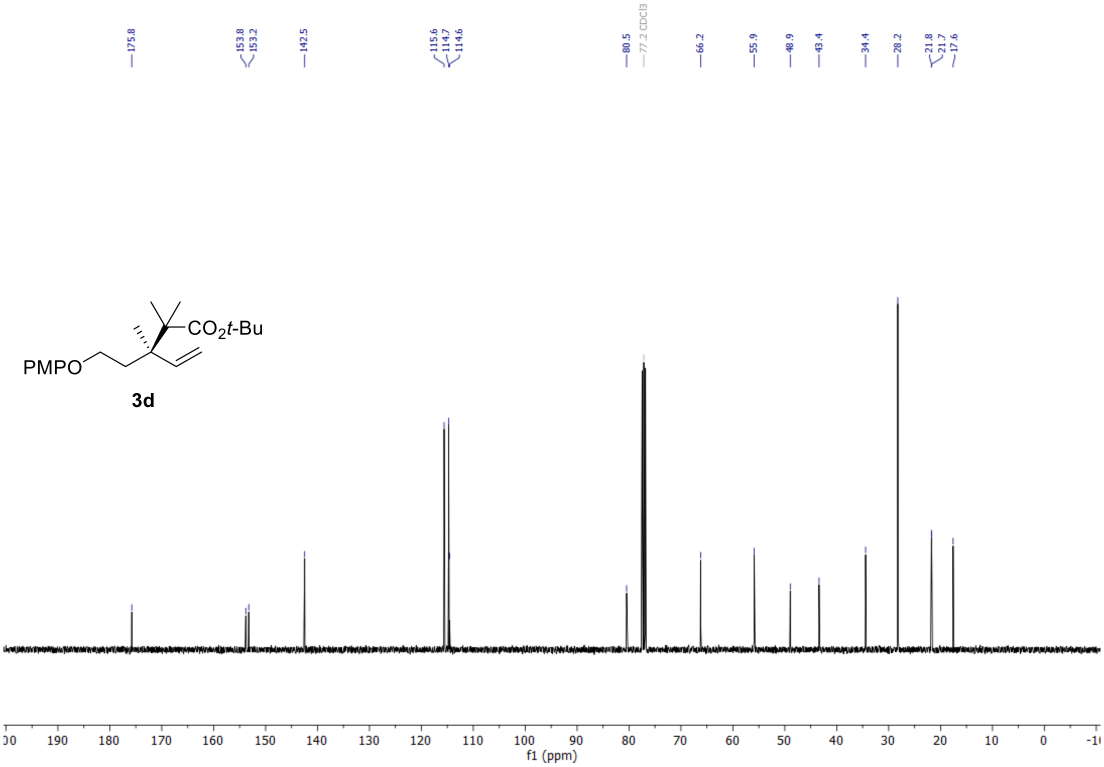

^{1}H NMR (400 MHz, $CDCl_3$) of **3e**

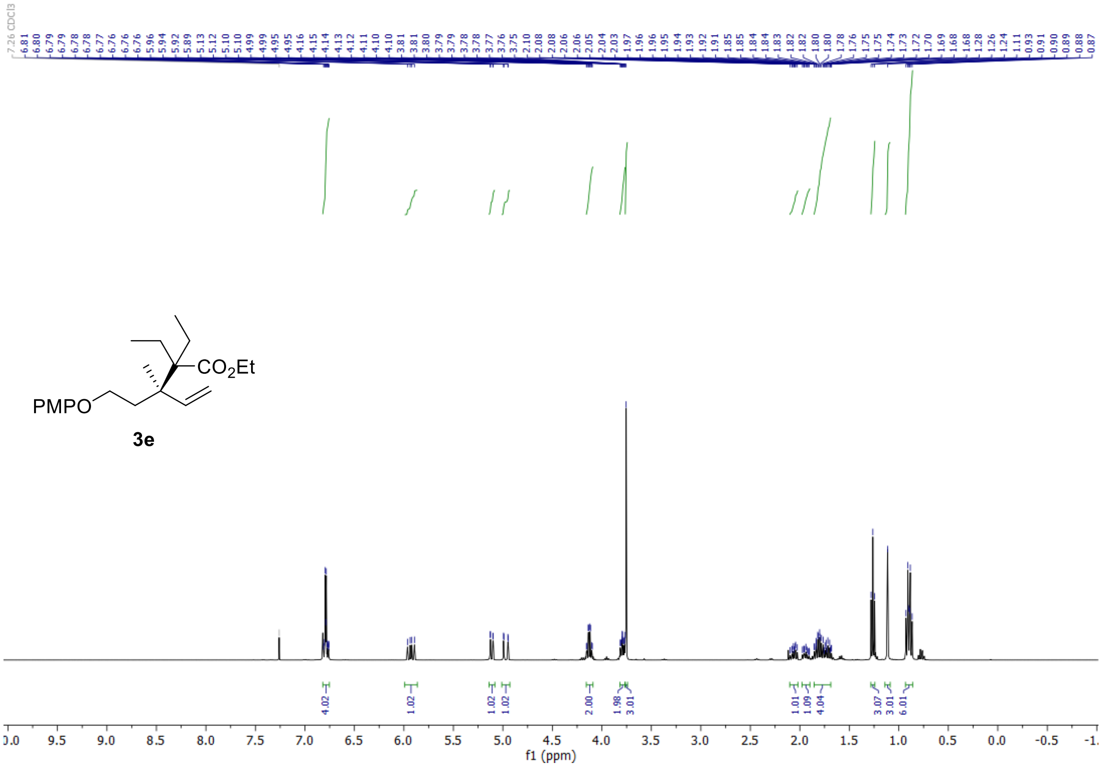

^{13}C NMR (101 MHz, $CDCl_3$) of **3e**

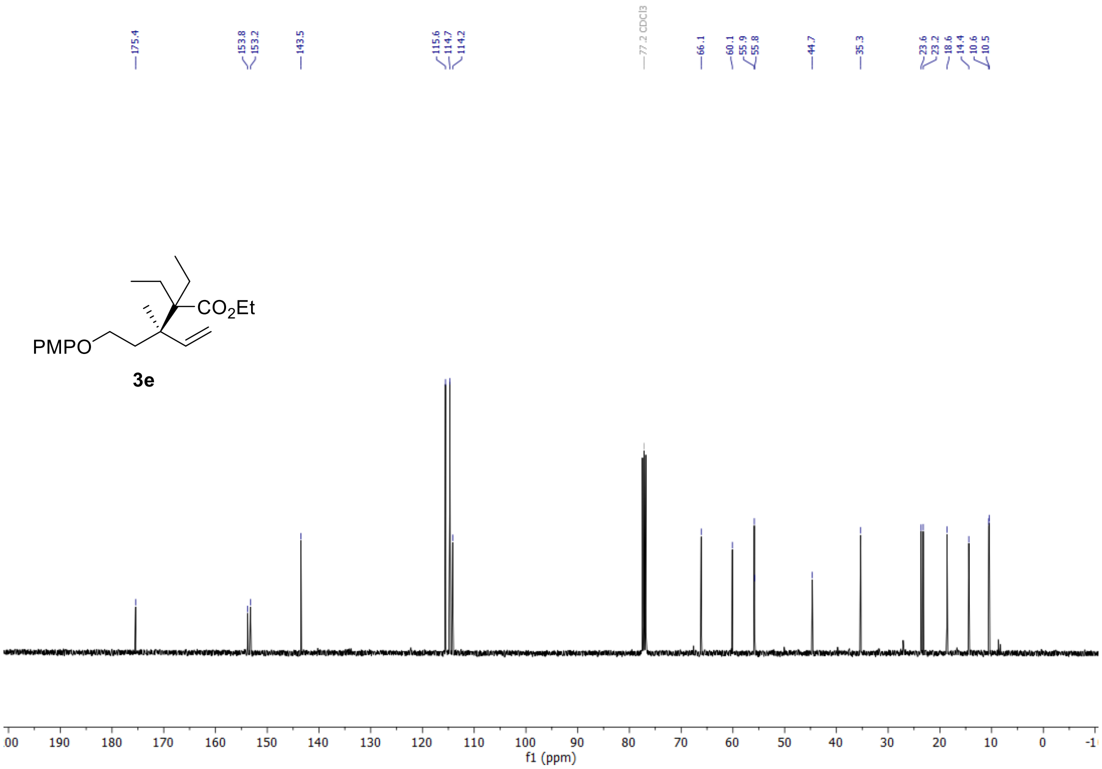

^{1}H NMR (400 MHz, $CDCl_3$) of **3f**

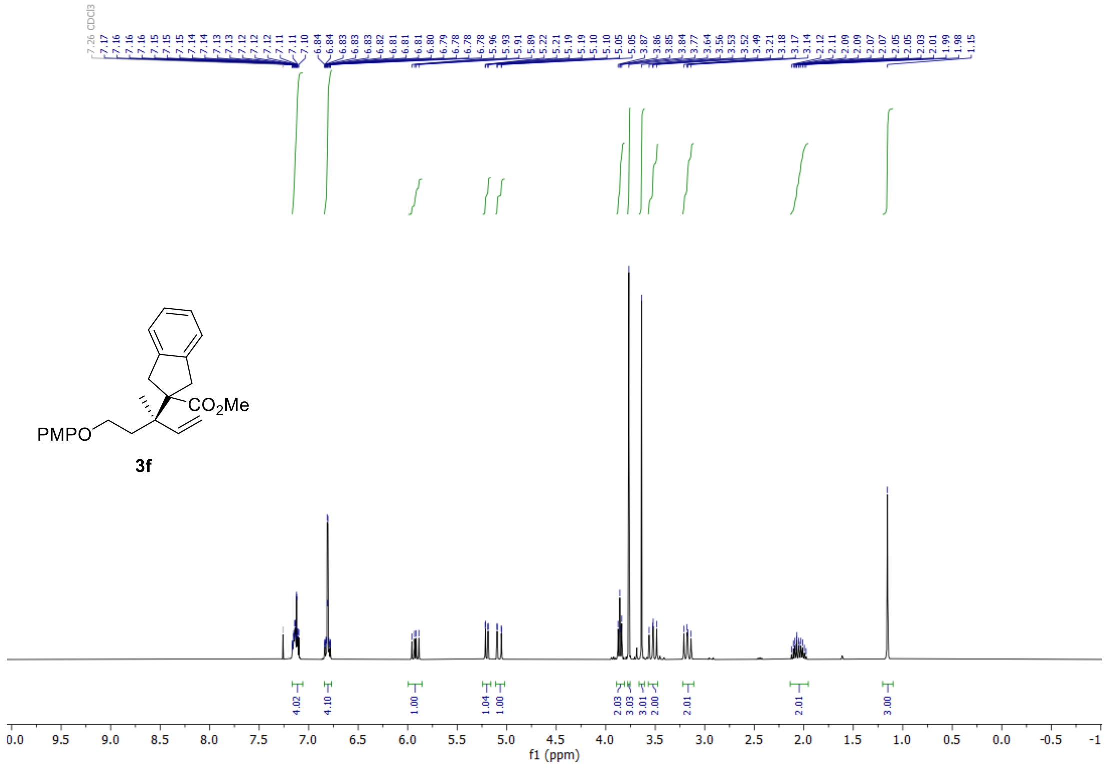

^{13}C NMR (101 MHz, $CDCl_3$) of **3f**

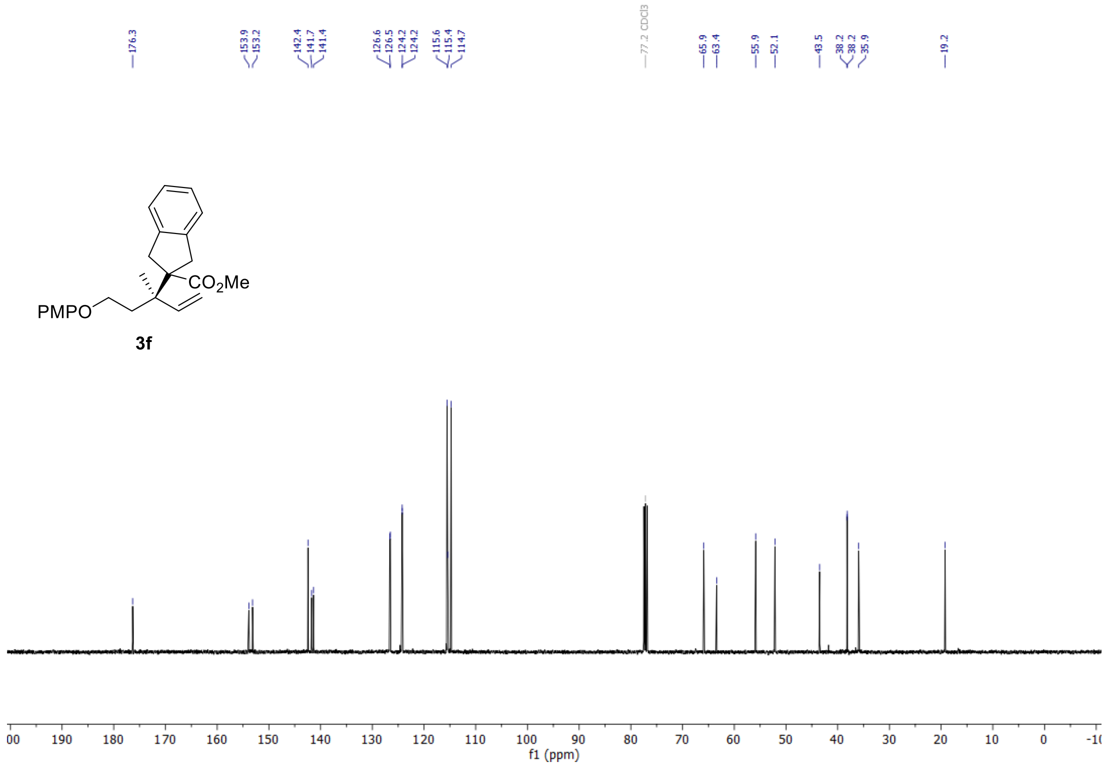

^{1}H NMR (400 MHz, $CDCl_3$) of **3g**

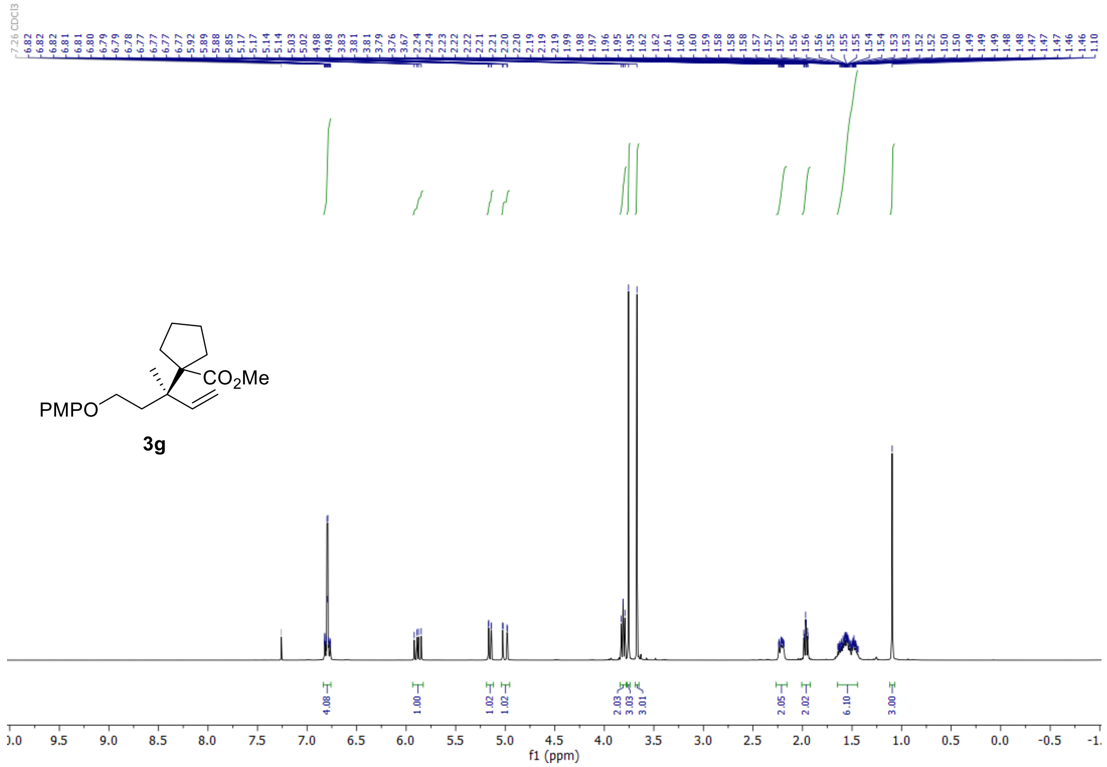

^{13}C NMR (101 MHz, $CDCl_3$) of **3g**

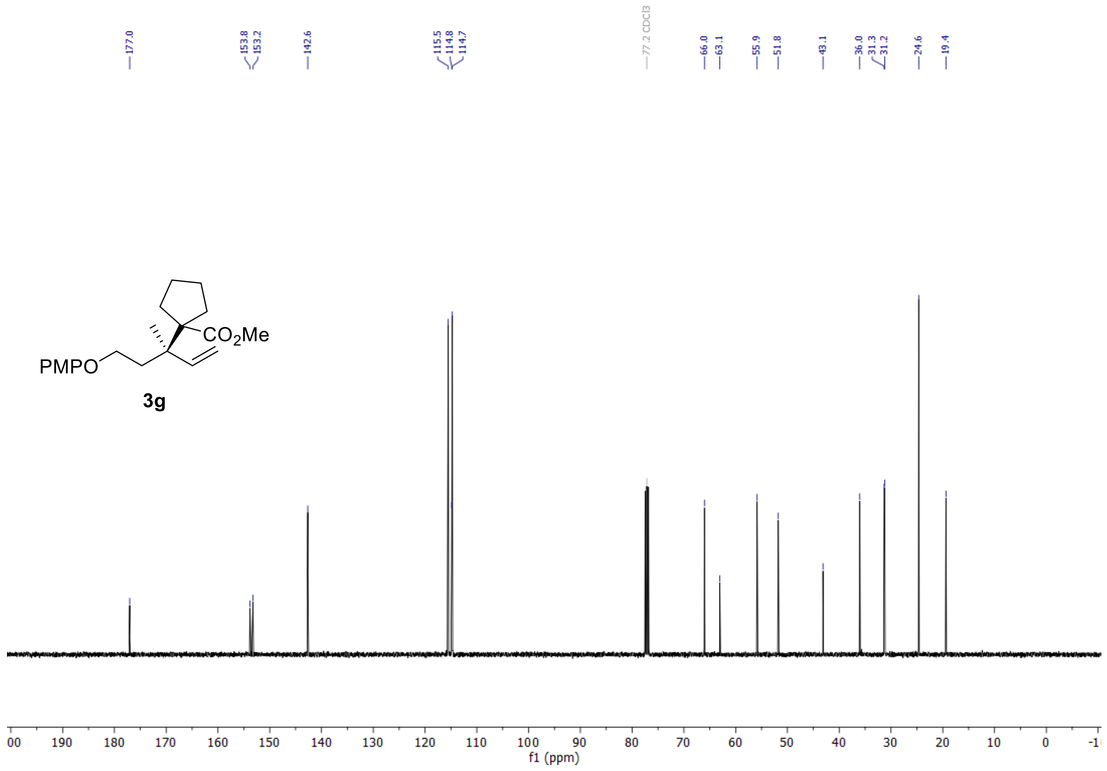

^{1}H NMR (400 MHz, $CDCl_3$) of **3h**

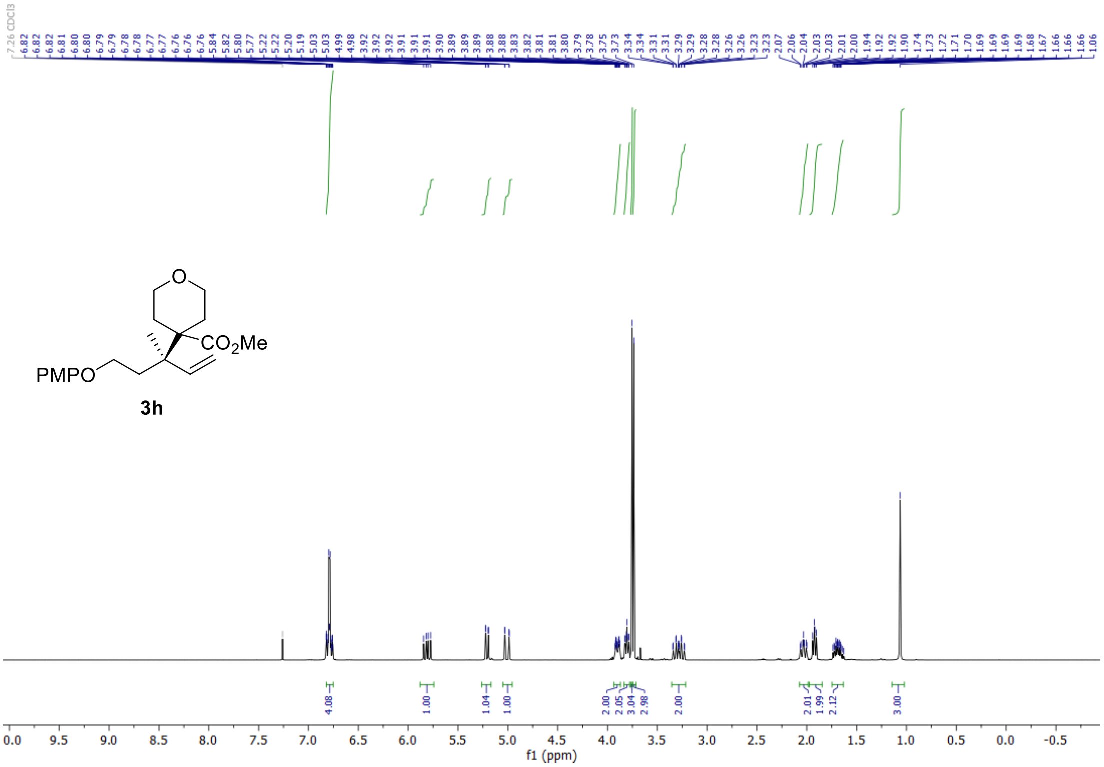

^{13}C NMR (101 MHz, $CDCl_3$) of **3h**

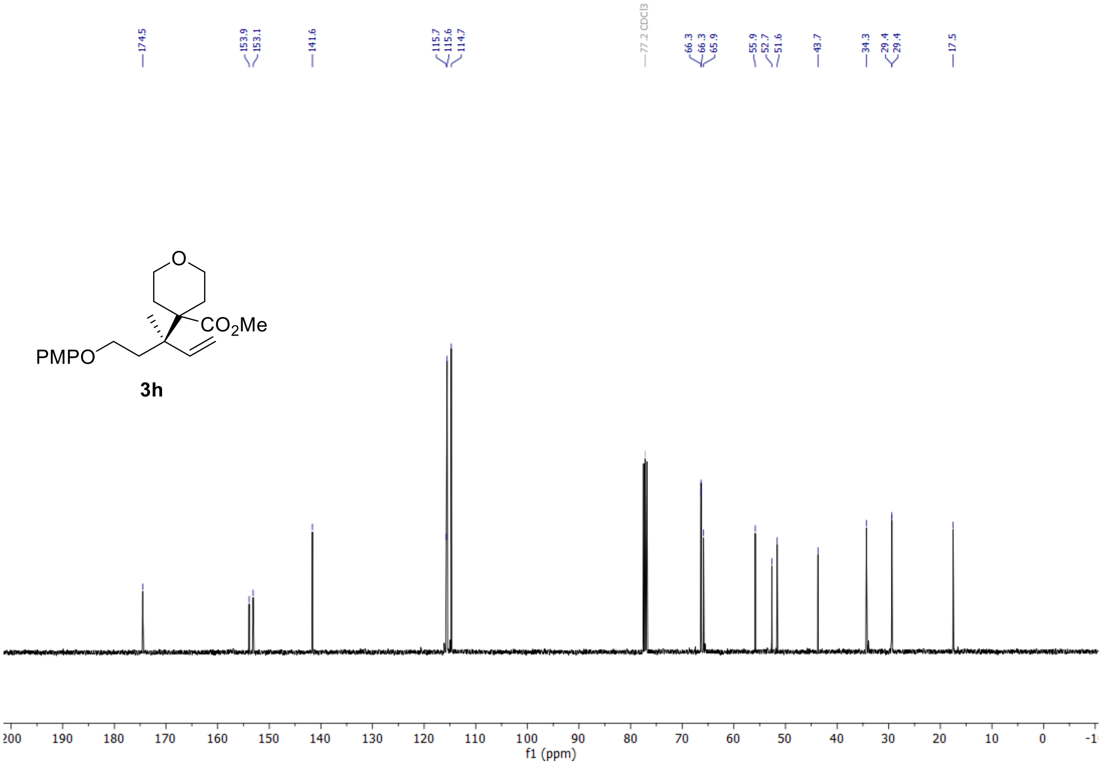

^{1}H NMR (400 MHz, $CDCl_3$) of **3i**

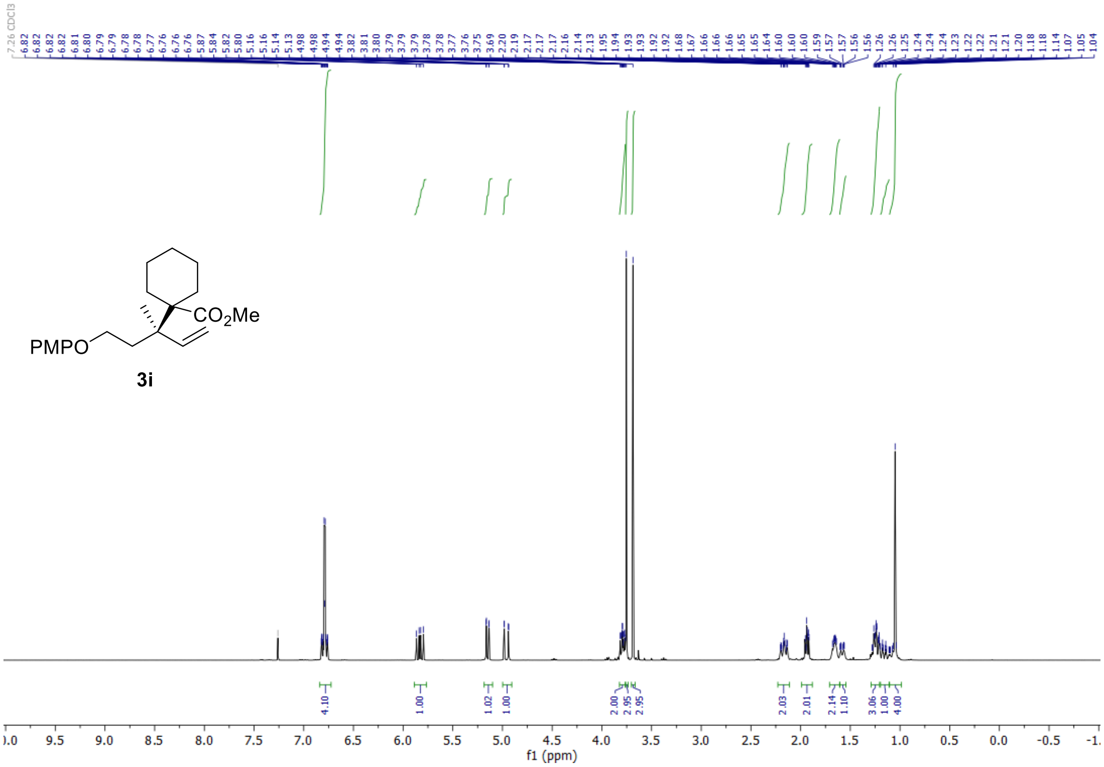

^{13}C NMR (101 MHz, $CDCl_3$) of **3i**

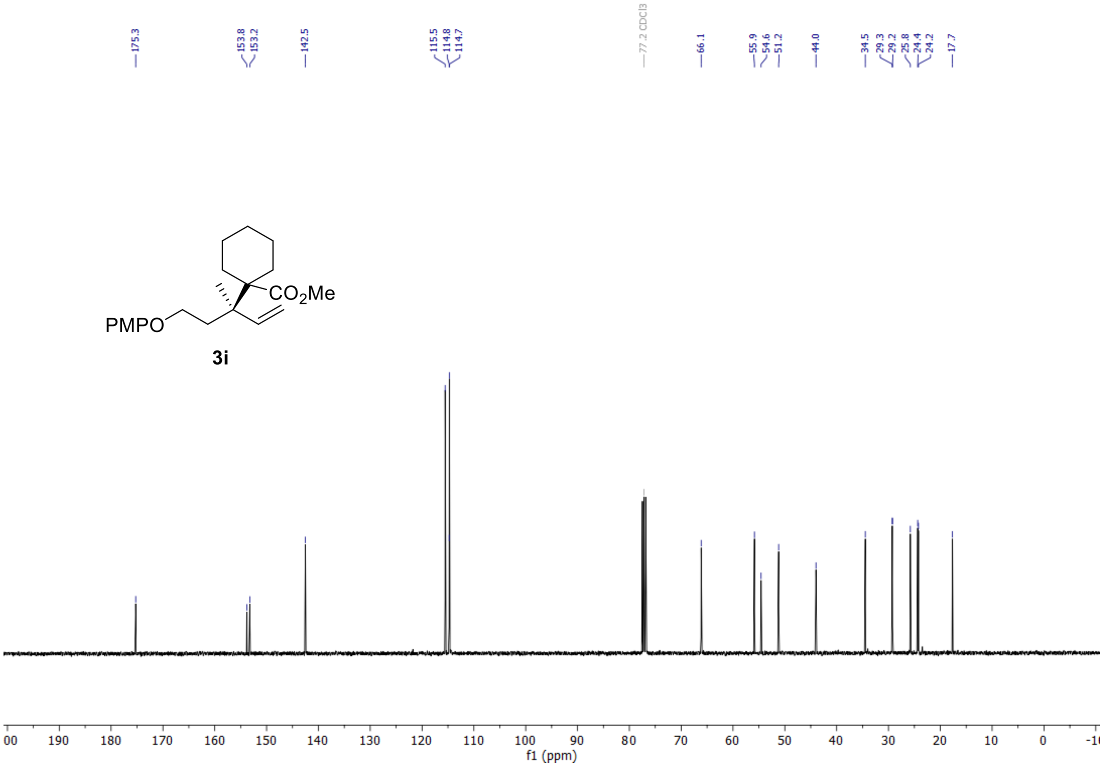

^{1}H NMR (400 MHz, $CDCl_3$) of **3j**

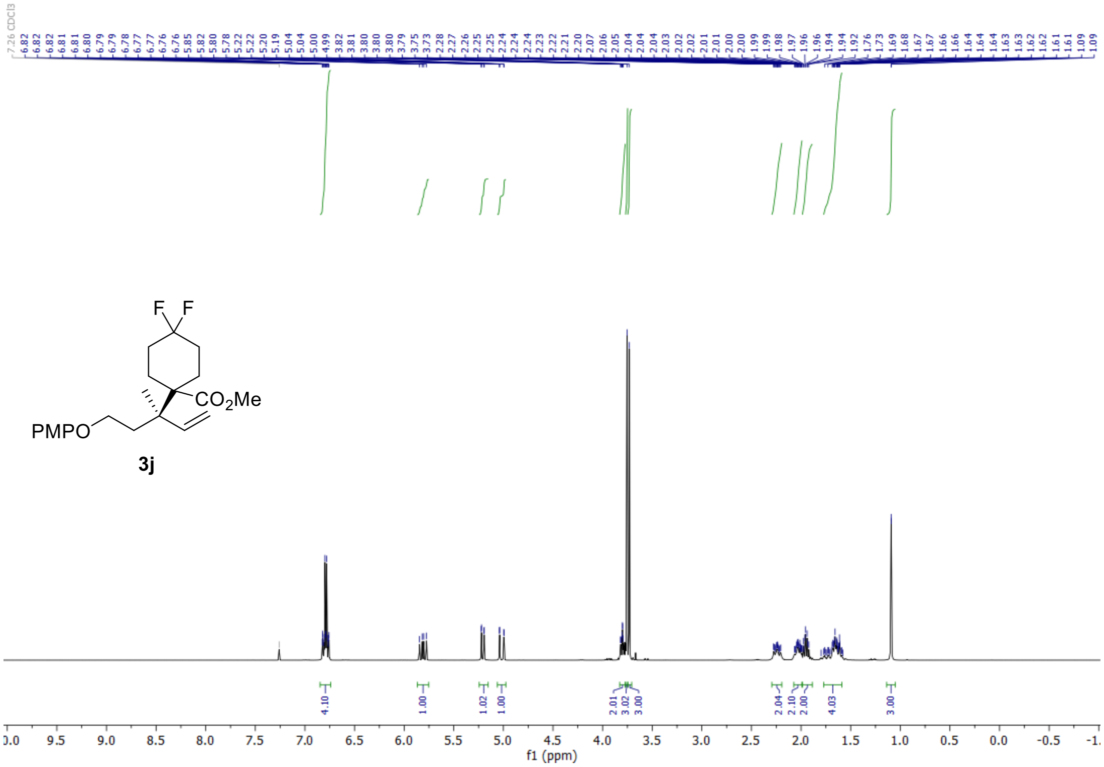

^{13}C NMR (101 MHz, $CDCl_3$) of **3j**

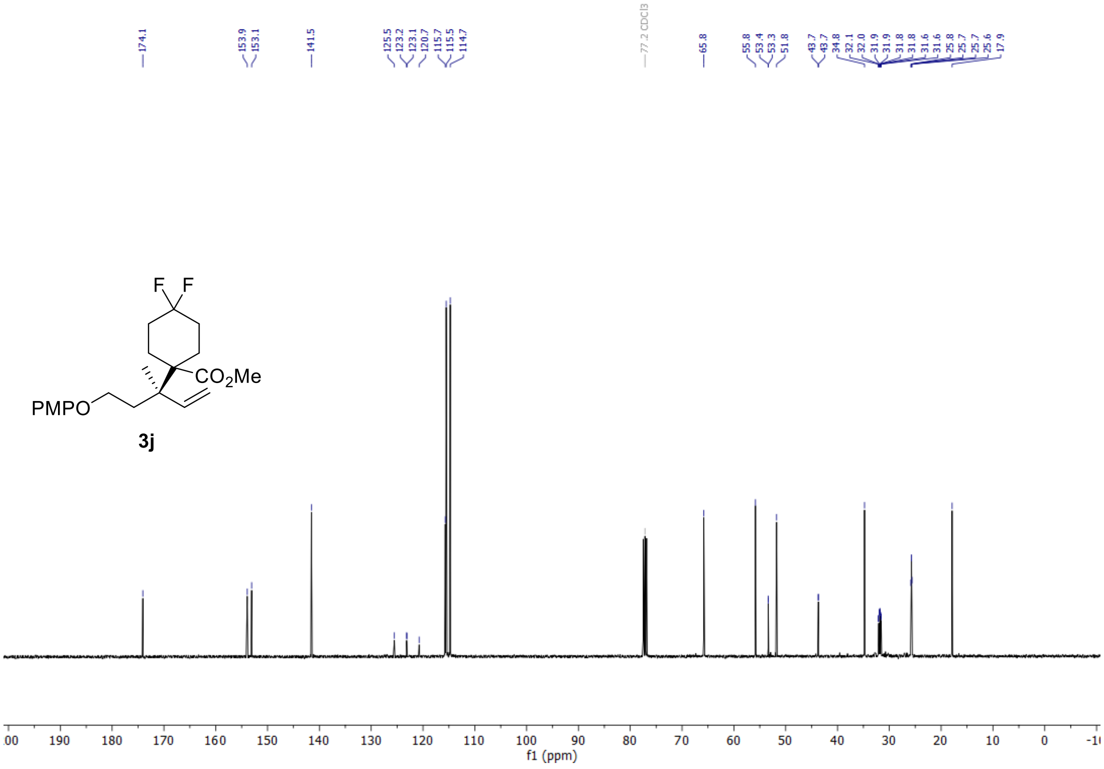

^{19}F NMR (376 MHz, $CDCl_3$) of **3j**

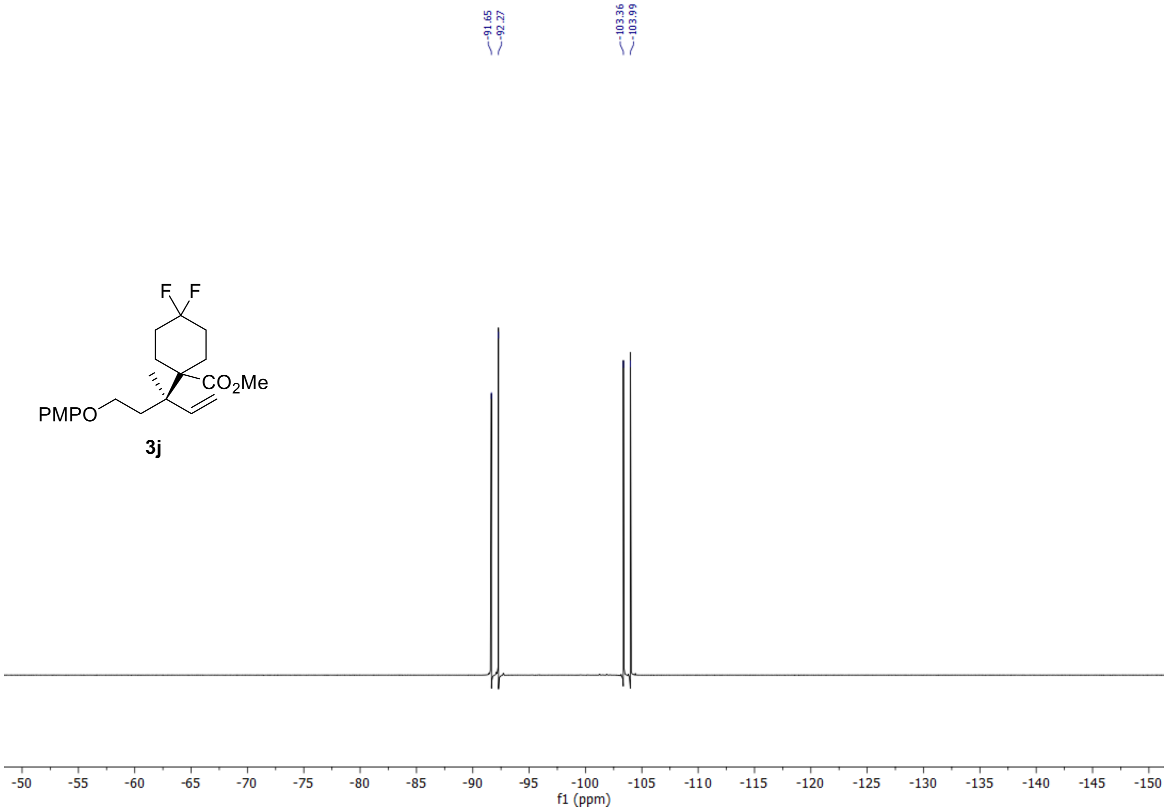

^{1}H NMR (400 MHz, $CDCl_3$) of **3k**

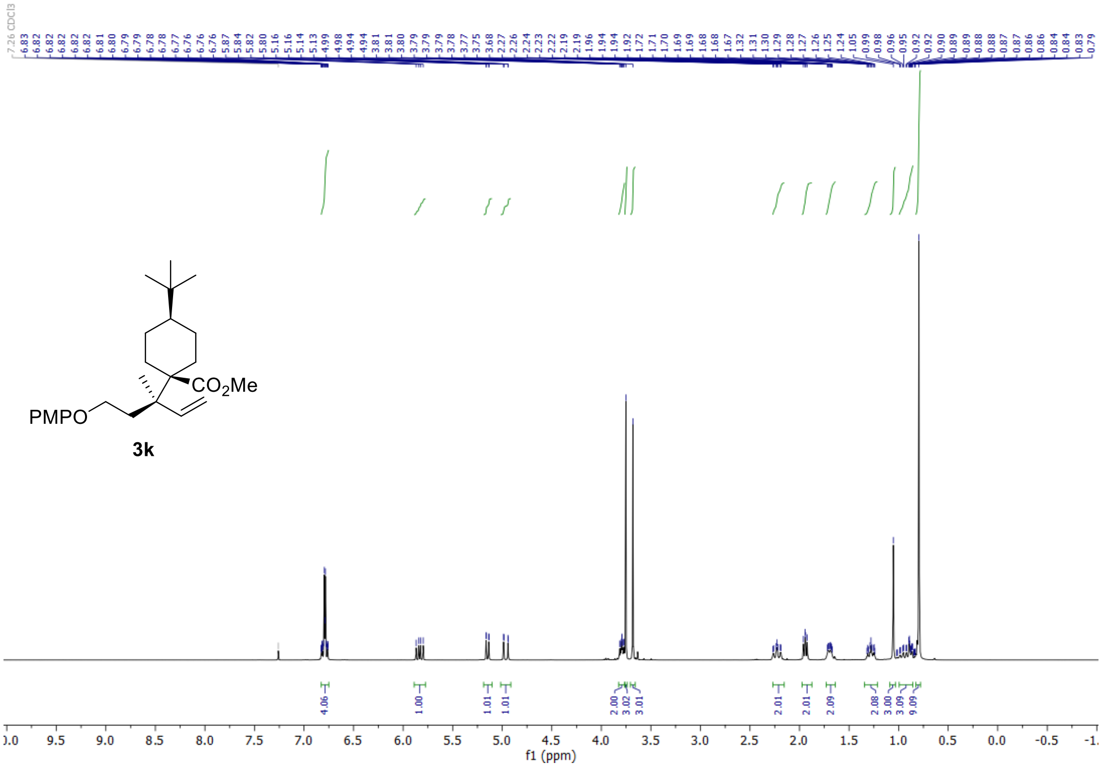

^{13}C NMR (101 MHz, $CDCl_3$) of **3k**

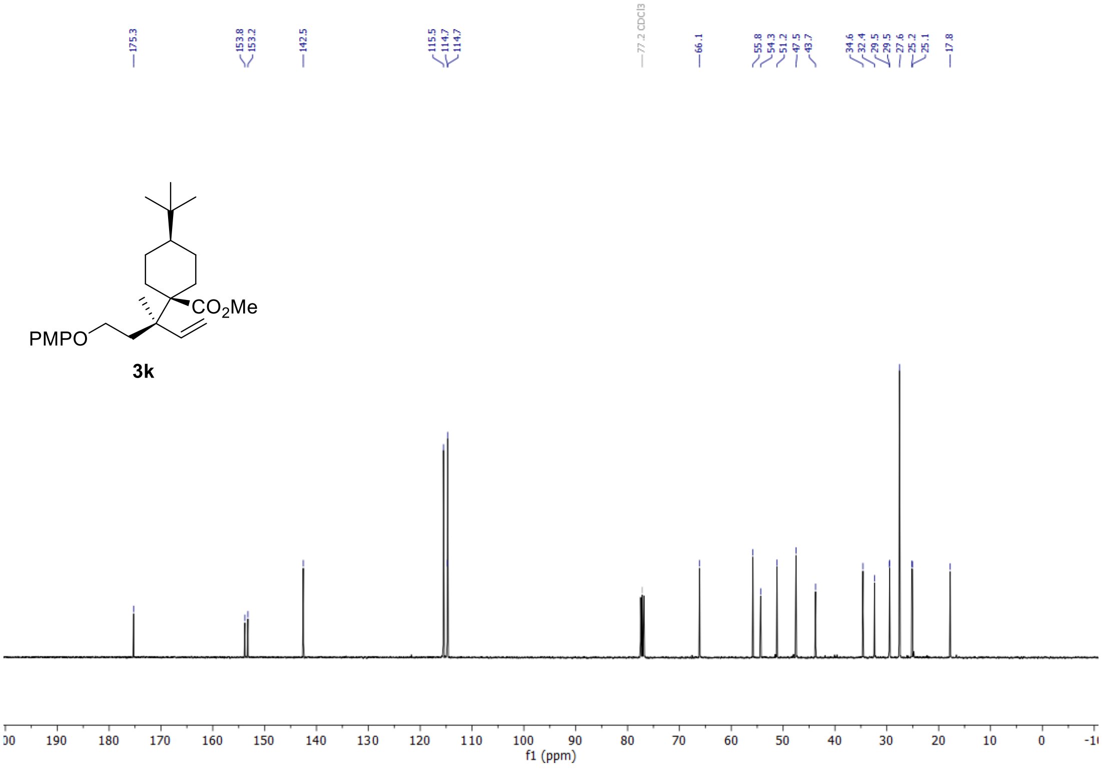

^{1}H NMR (400 MHz, $CDCl_3$) of **3l**

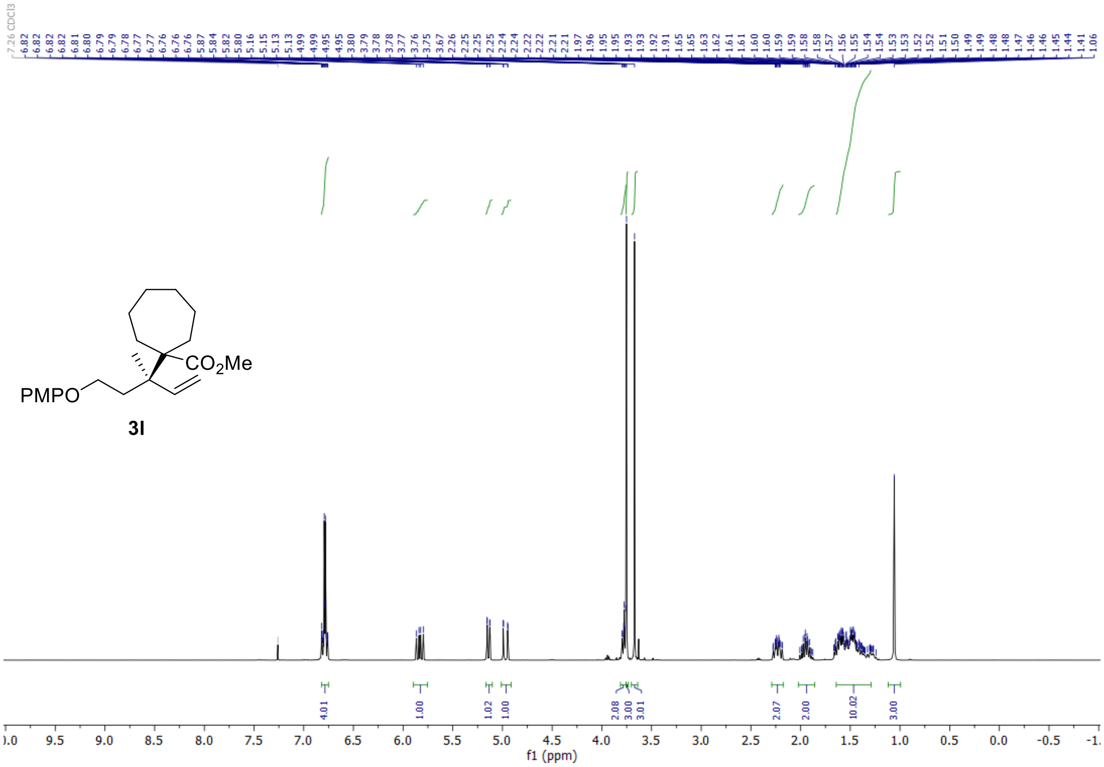

^{13}C NMR (101 MHz, $CDCl_3$) of **3l**

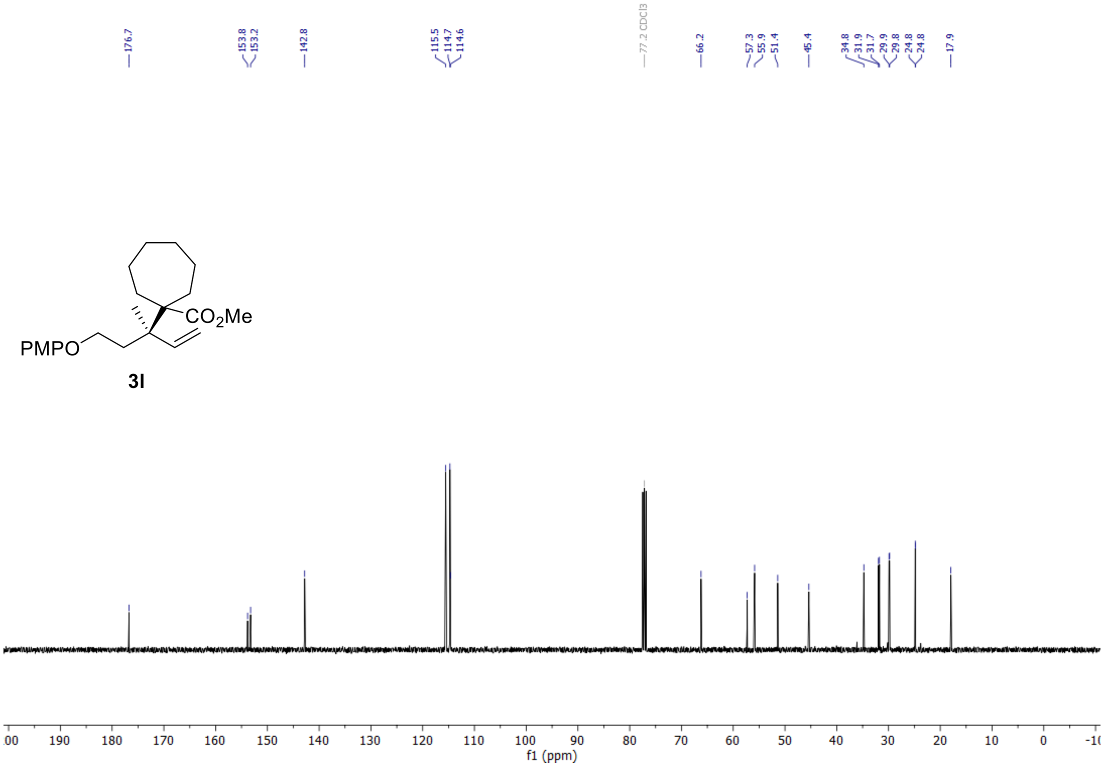

^{1}H NMR (400 MHz, $CDCl_3$) of **3m**

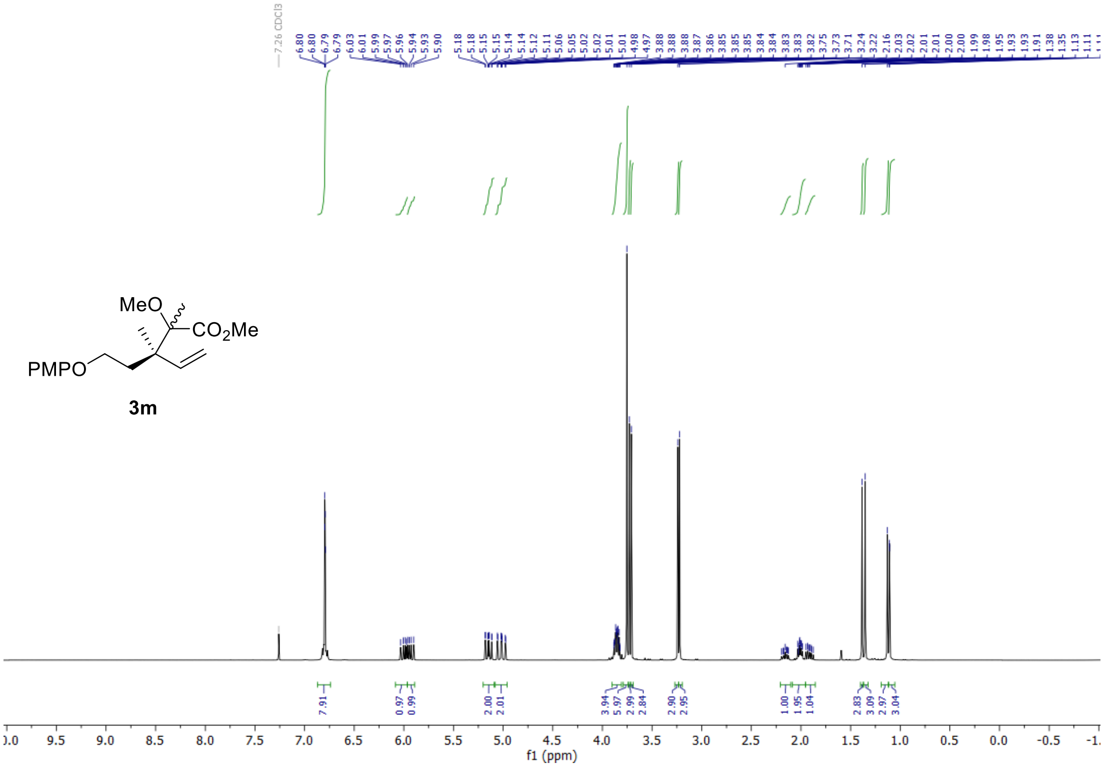

^{13}C NMR (101 MHz, $CDCl_3$) of **3m**

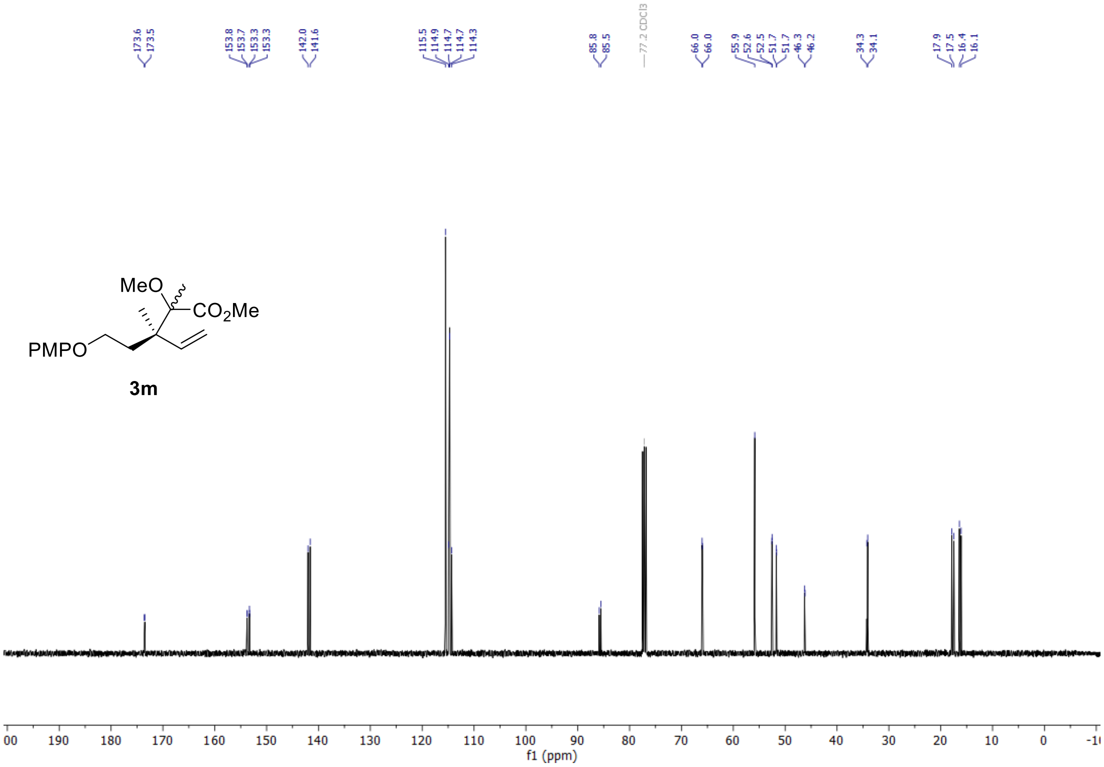

^{1}H NMR (400 MHz, $CDCl_3$) of **3n**

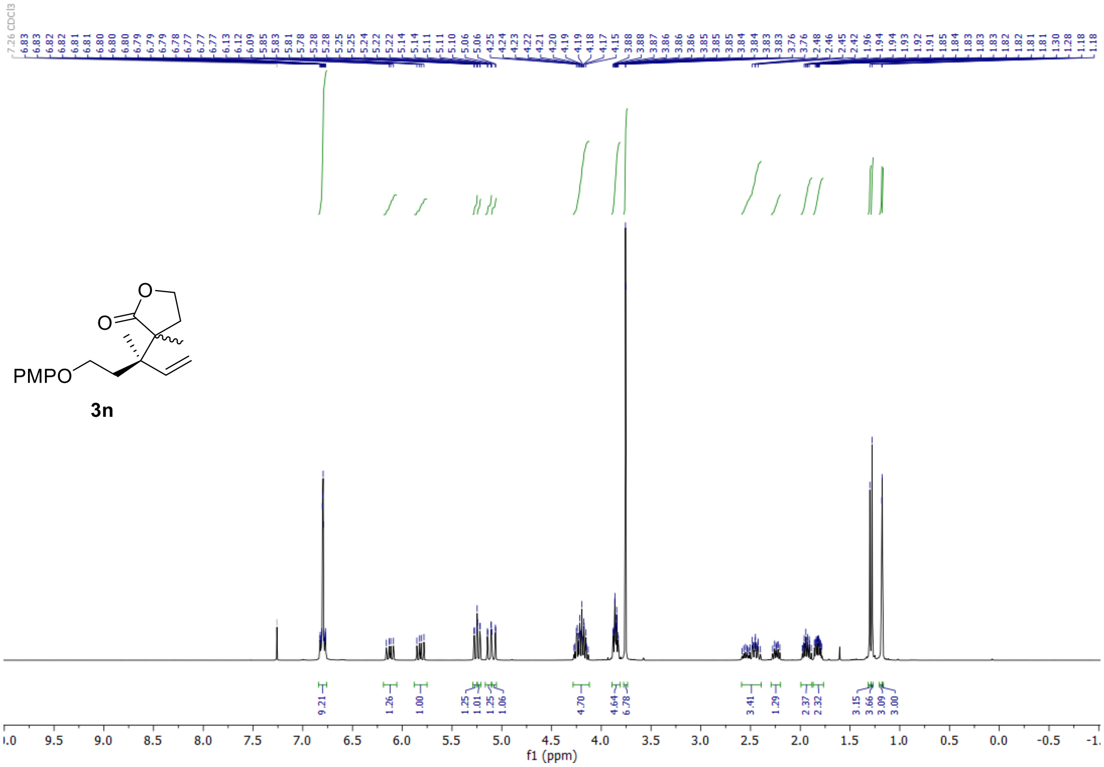

^{13}C NMR (101 MHz, $CDCl_3$) of **3n**

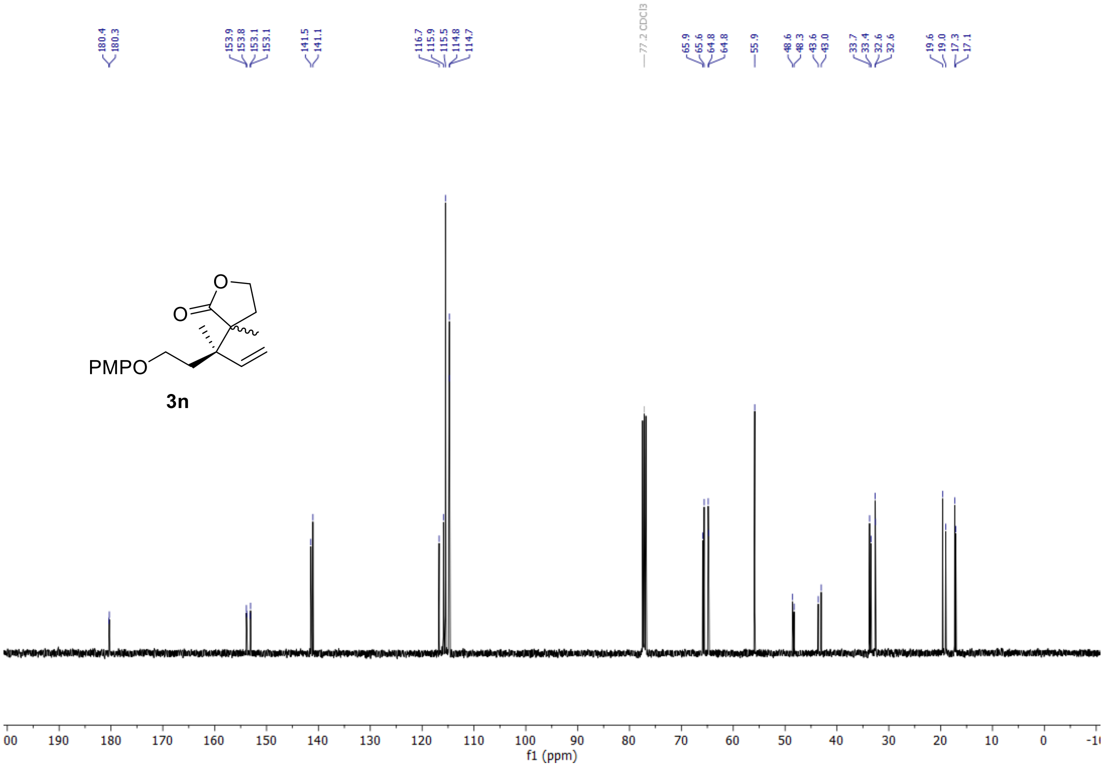

^{1}H NMR (500 MHz, $CDCl_3$) of **SI-1**

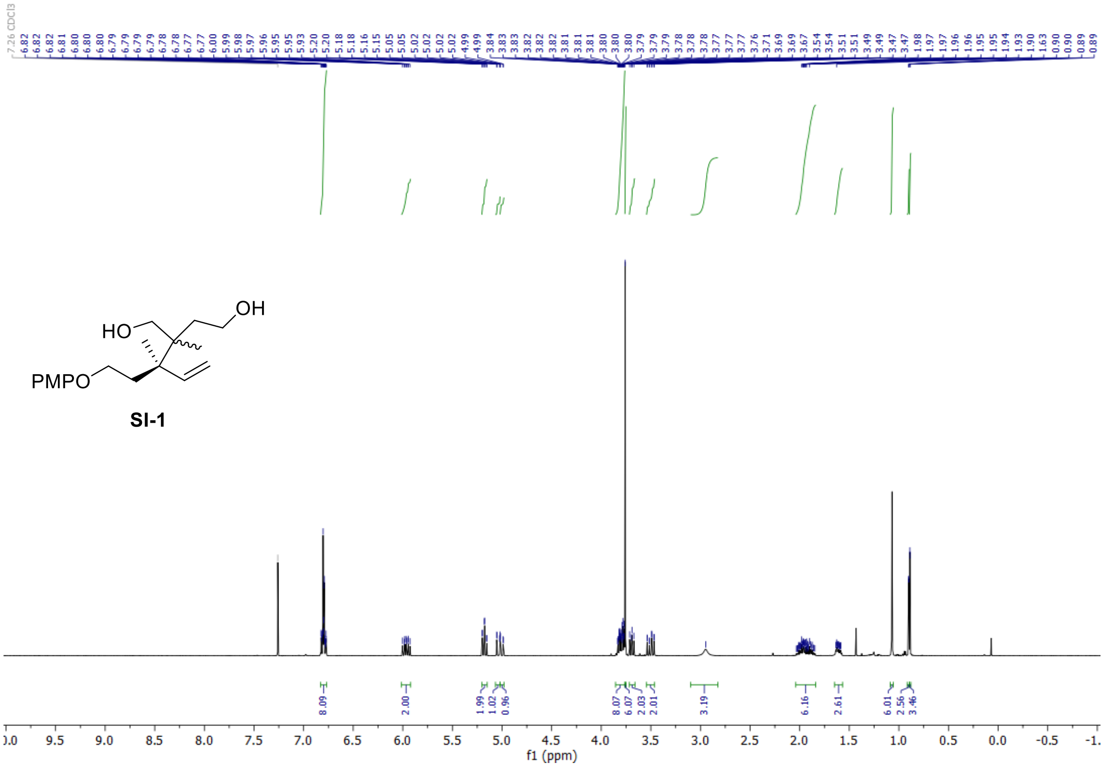

^{13}C NMR (126 MHz, $CDCl_3$) of **SI-1**

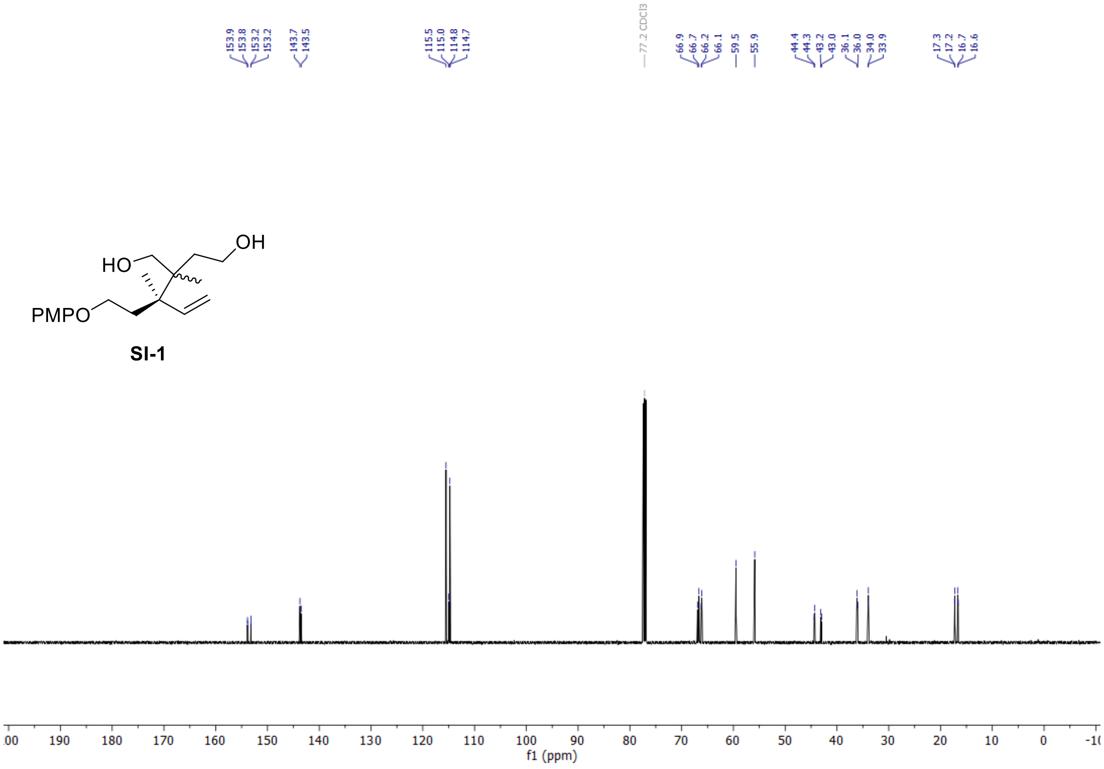

^{1}H NMR (500 MHz, $CDCl_3$) of **3s**

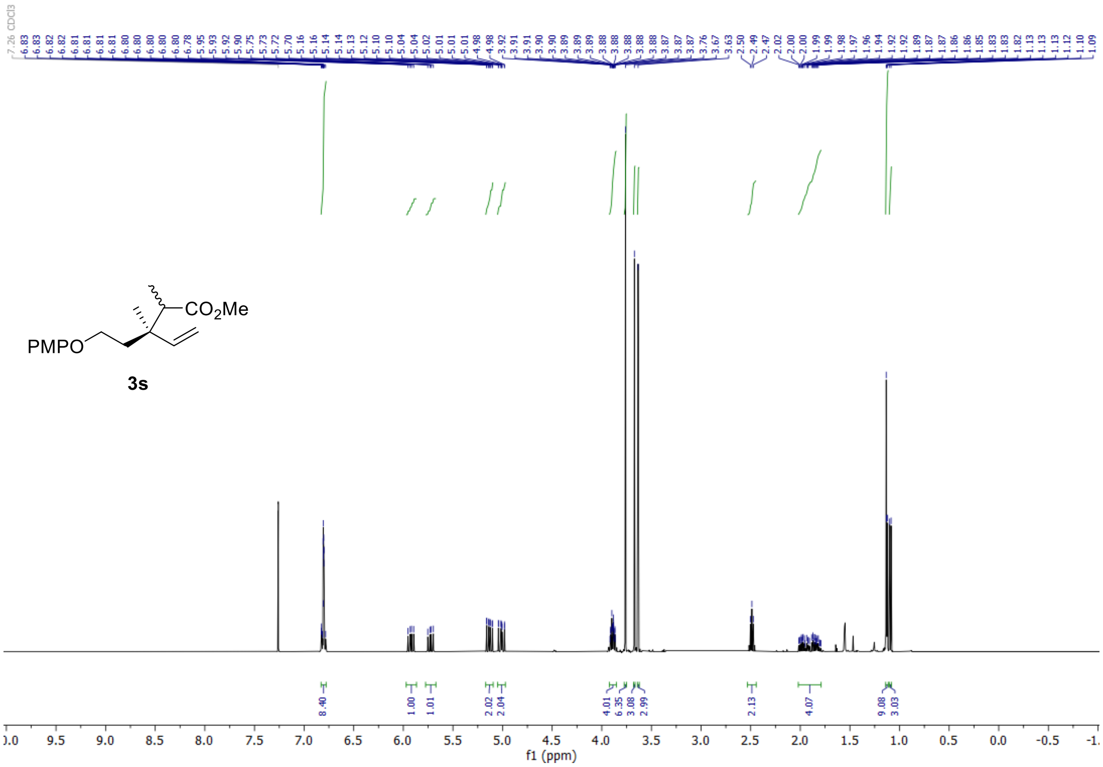

^{13}C NMR (126 MHz, $CDCl_3$) of **3s**

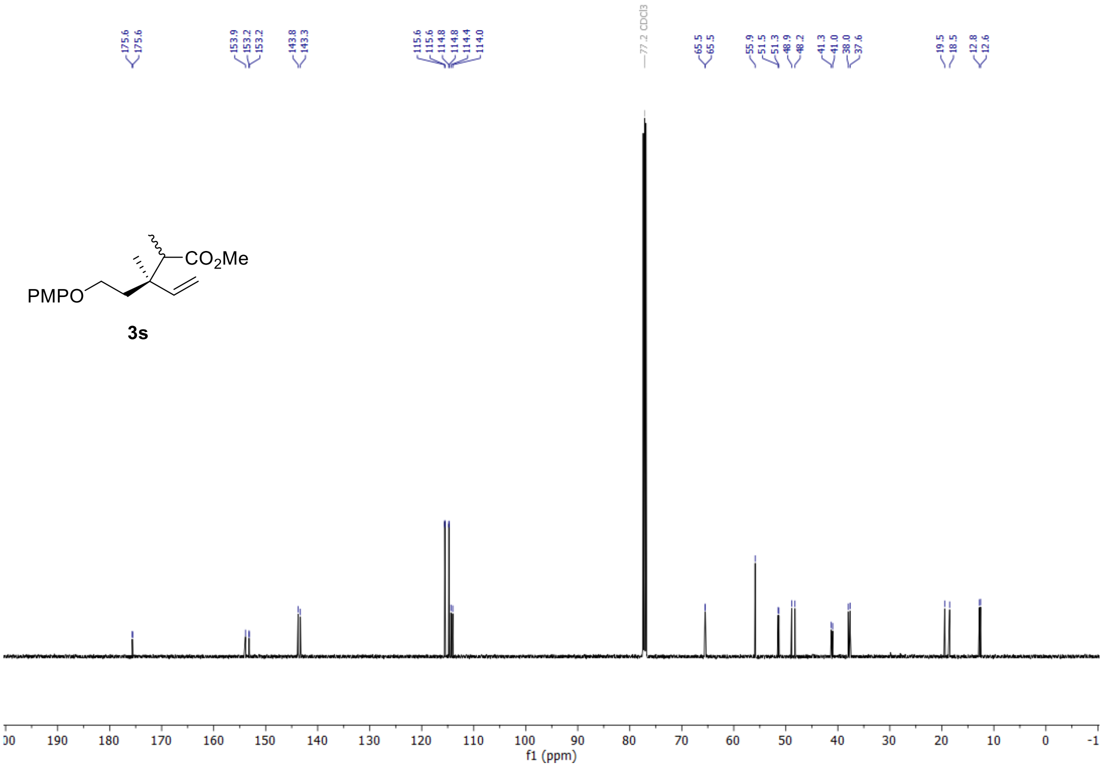

^{1}H NMR (400 MHz, $CDCl_3$) of **4b**

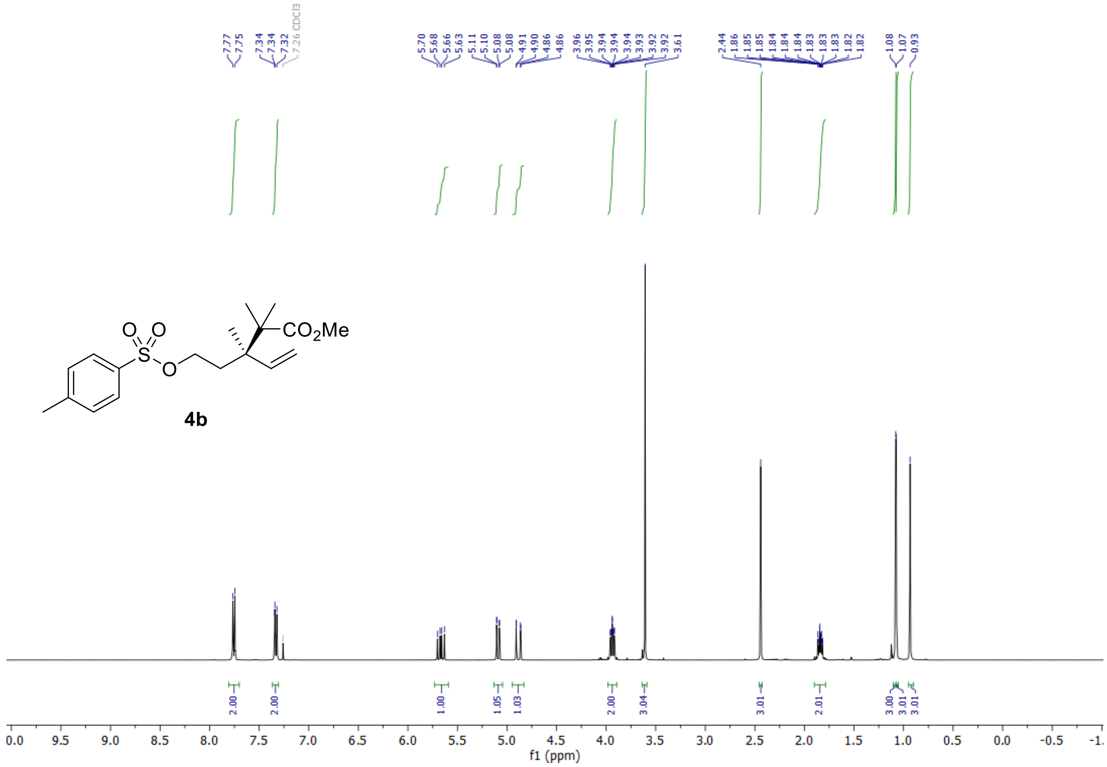

^{13}C NMR (101 MHz, $CDCl_3$) of **4b**

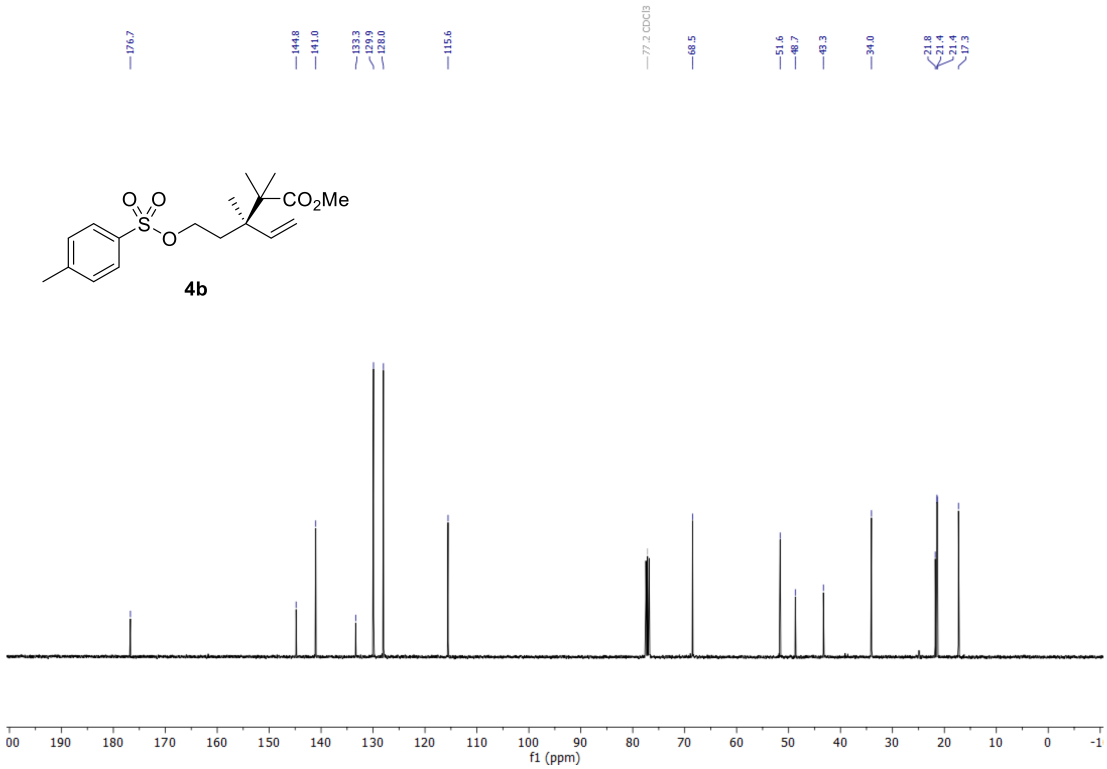

^{1}H NMR (400 MHz, $CDCl_3$) of **4c**

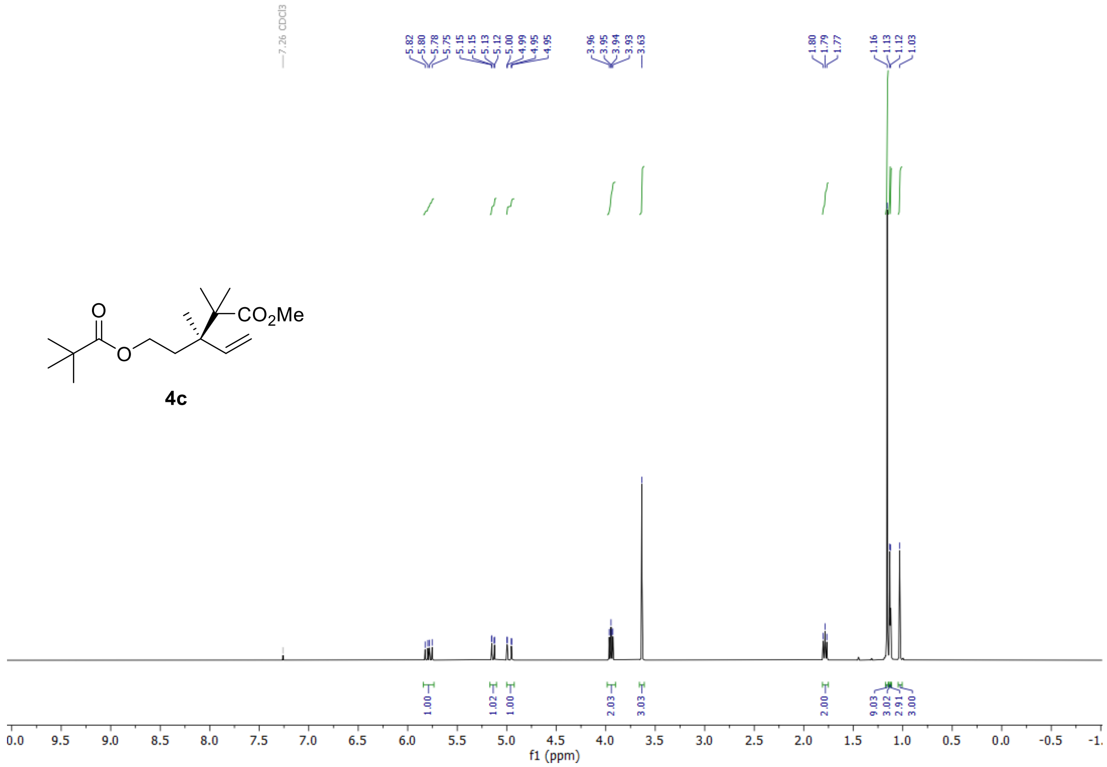

^{13}C NMR (101 MHz, $CDCl_3$) of **4c**

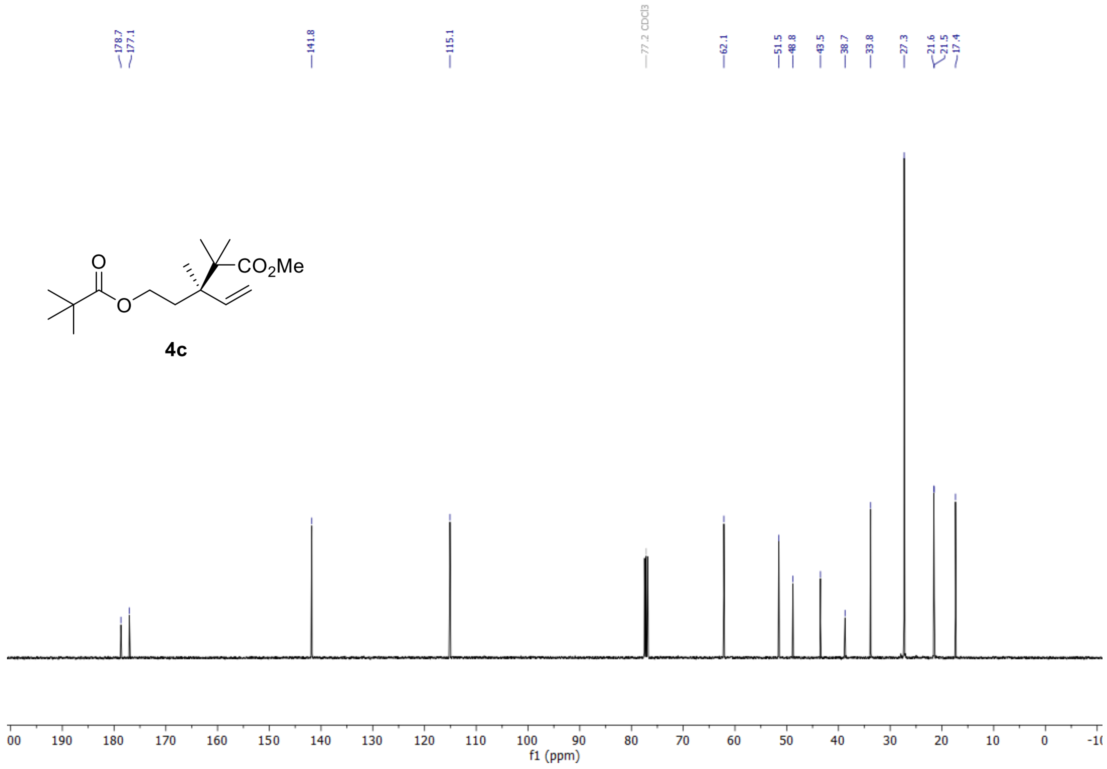

^{1}H NMR (400 MHz, $CDCl_3$) of **SI-2**

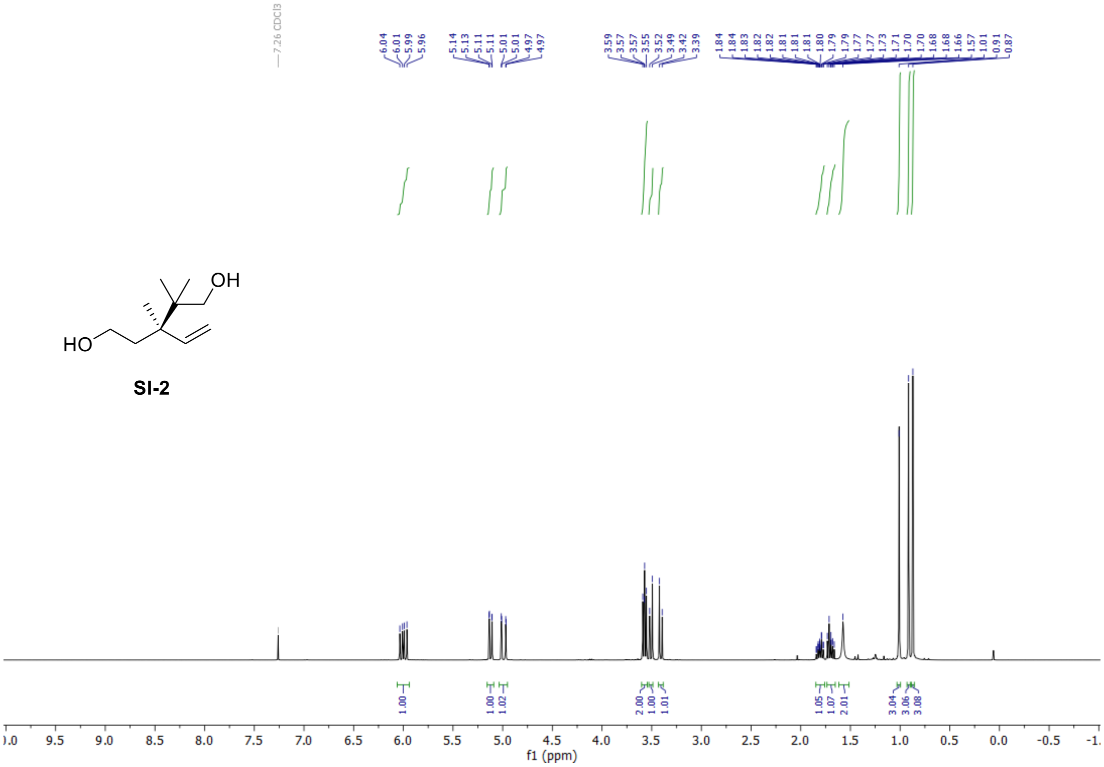

^{13}C NMR (101 MHz, $CDCl_3$) of **SI-2**

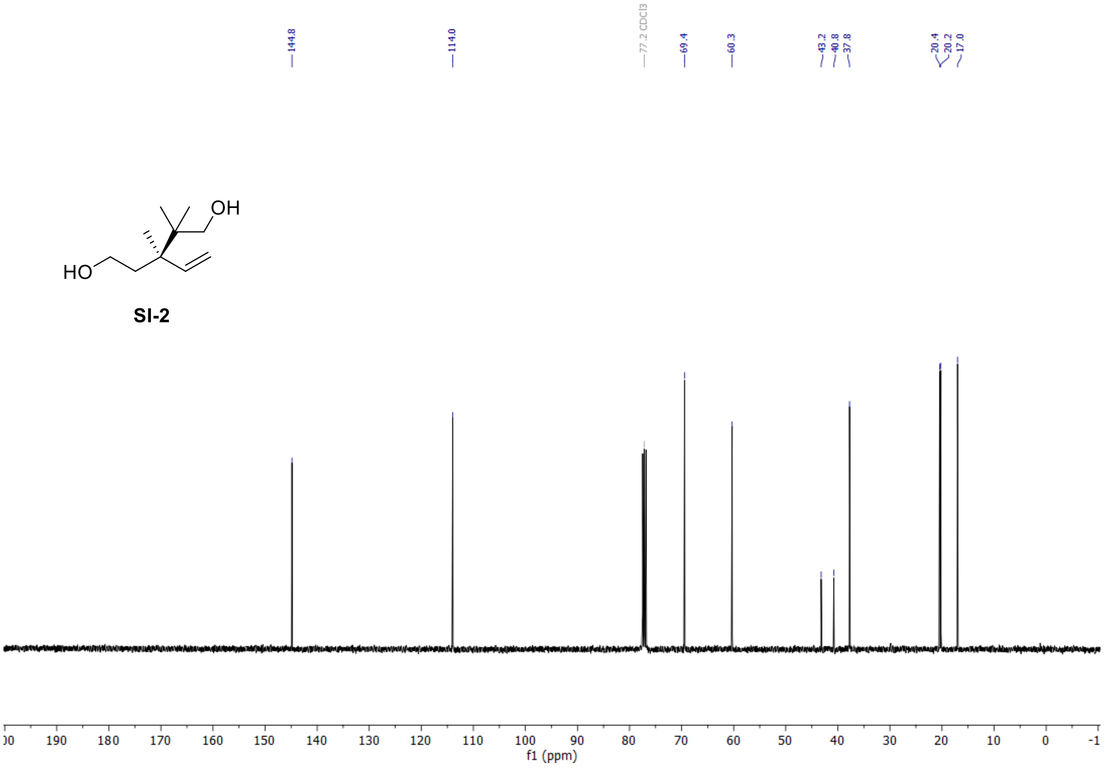

^{1}H NMR (400 MHz, $CDCl_3$) of **4d**

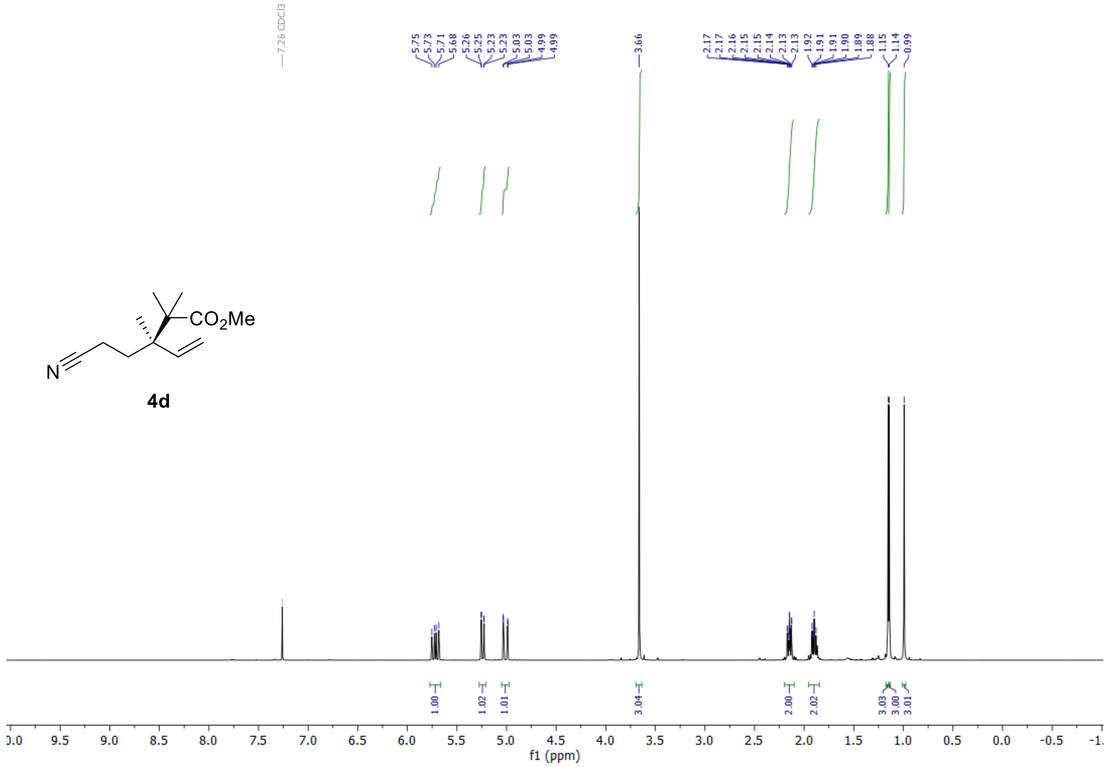

^{13}C NMR (101 MHz, $CDCl_3$) of **4d**

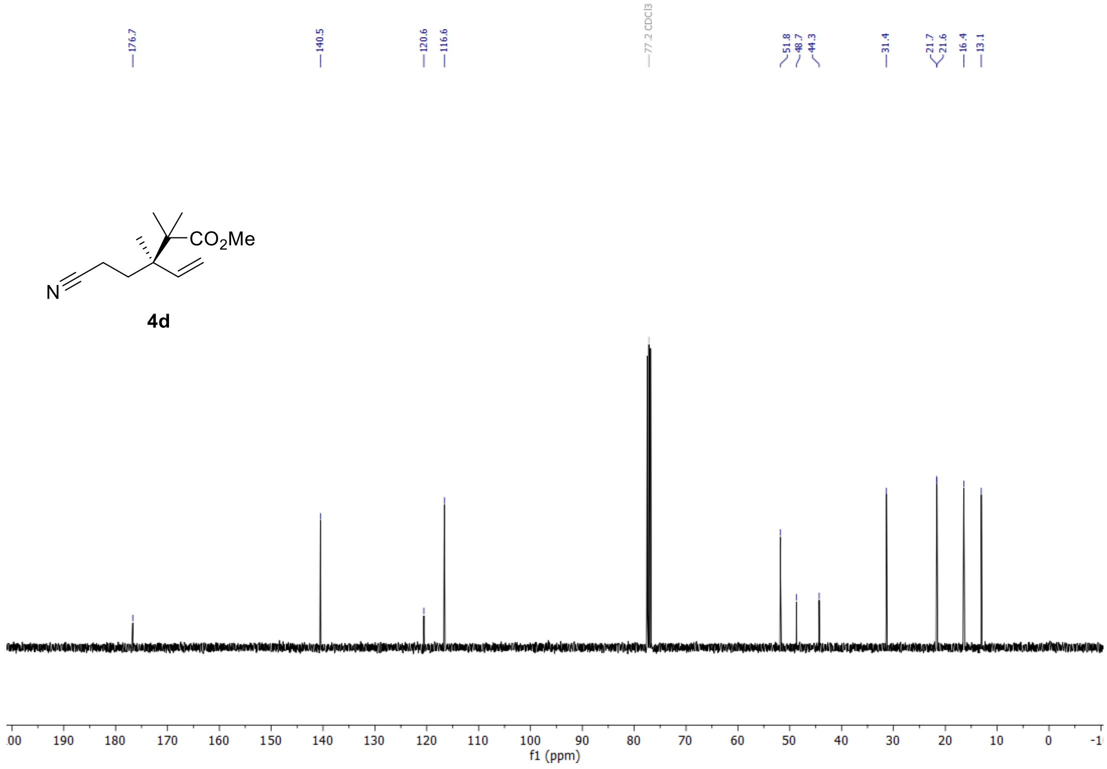

^{1}H NMR (400 MHz, $CDCl_3$) of **4e**

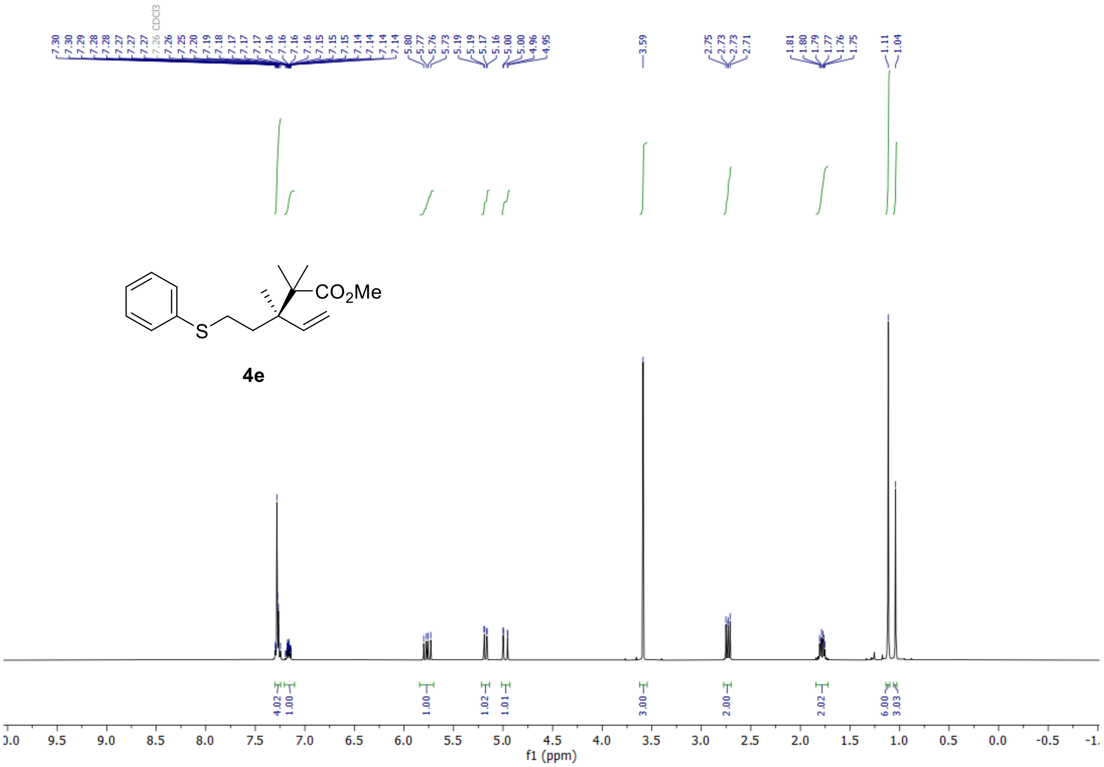

^{13}C NMR (101 MHz, $CDCl_3$) of **4e**

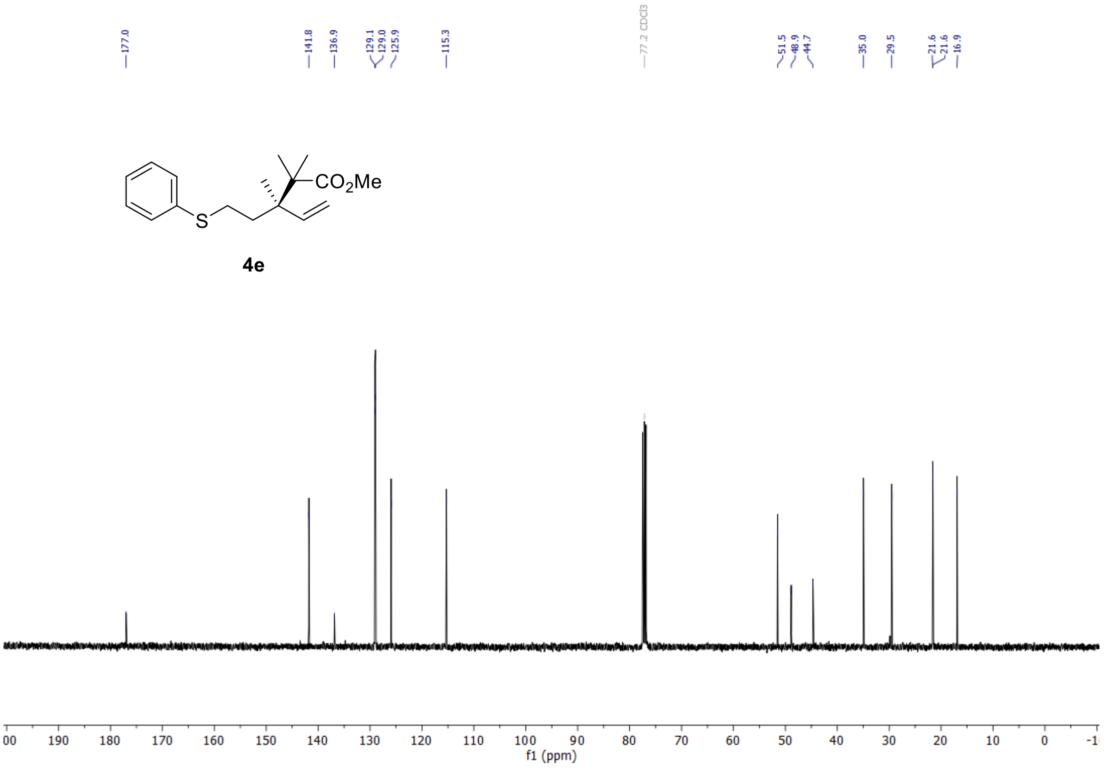

^{1}H NMR (400 MHz, $CDCl_3$) of **4f**

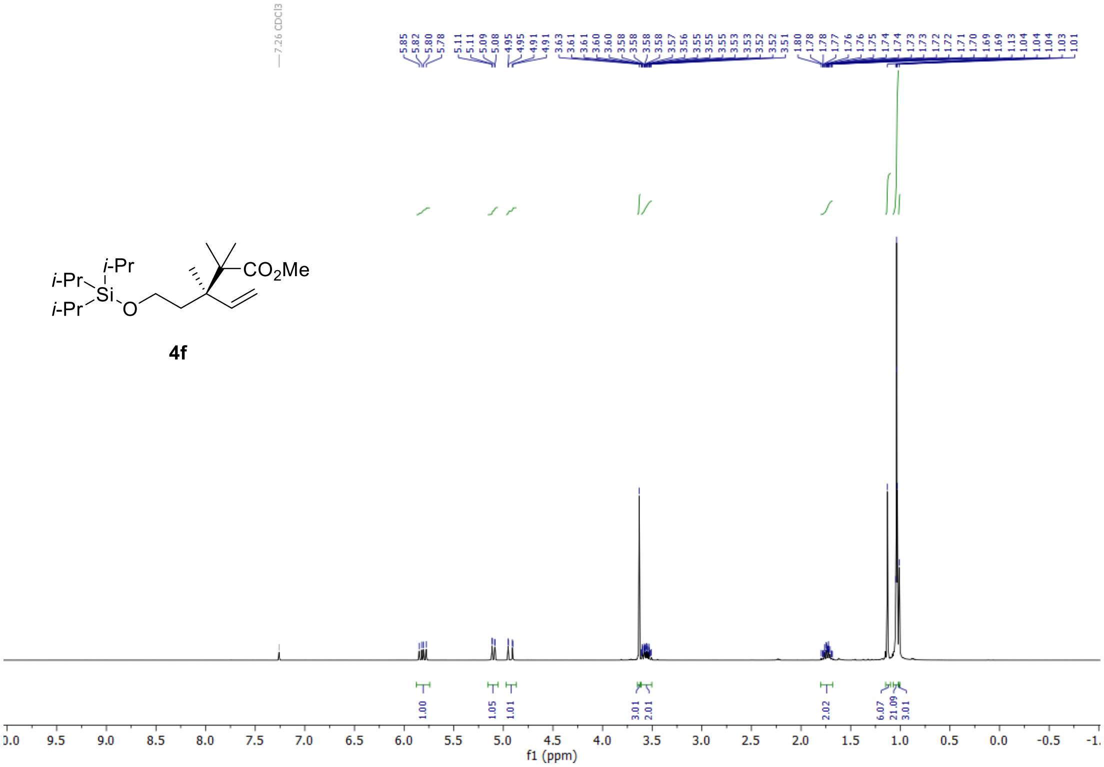

^{13}C NMR (101 MHz, $CDCl_3$) of **4f**

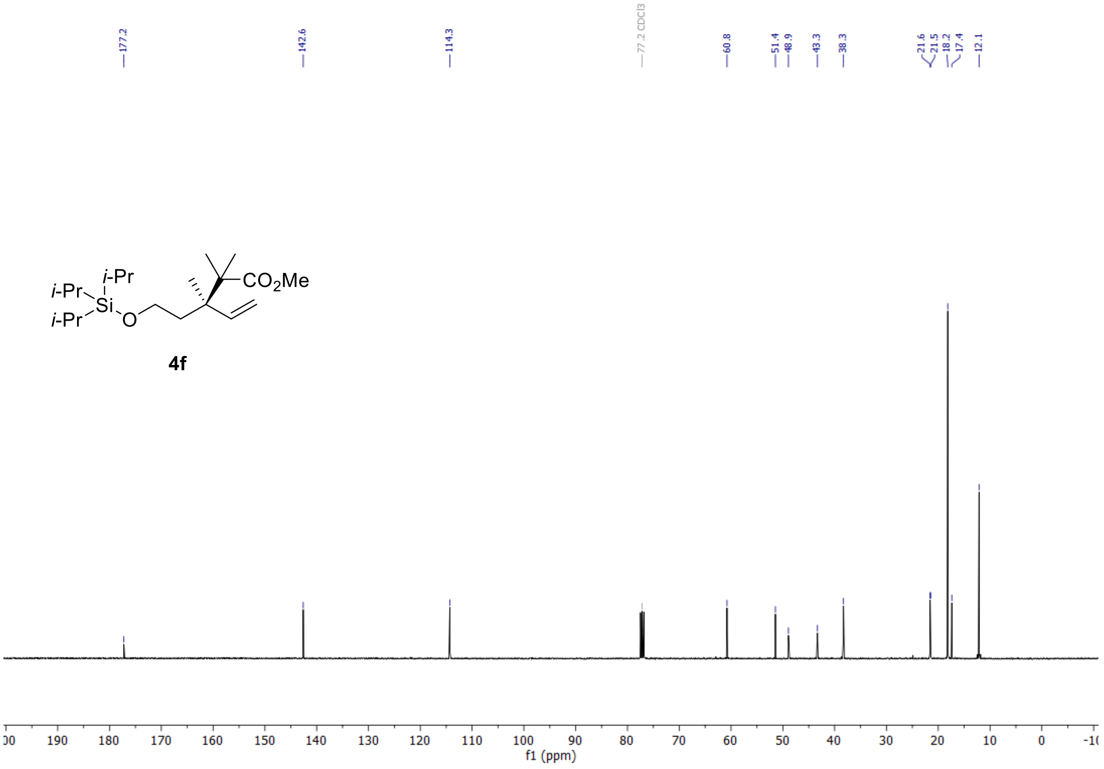

^{1}H NMR (400 MHz, $CDCl_3$) of **4g**

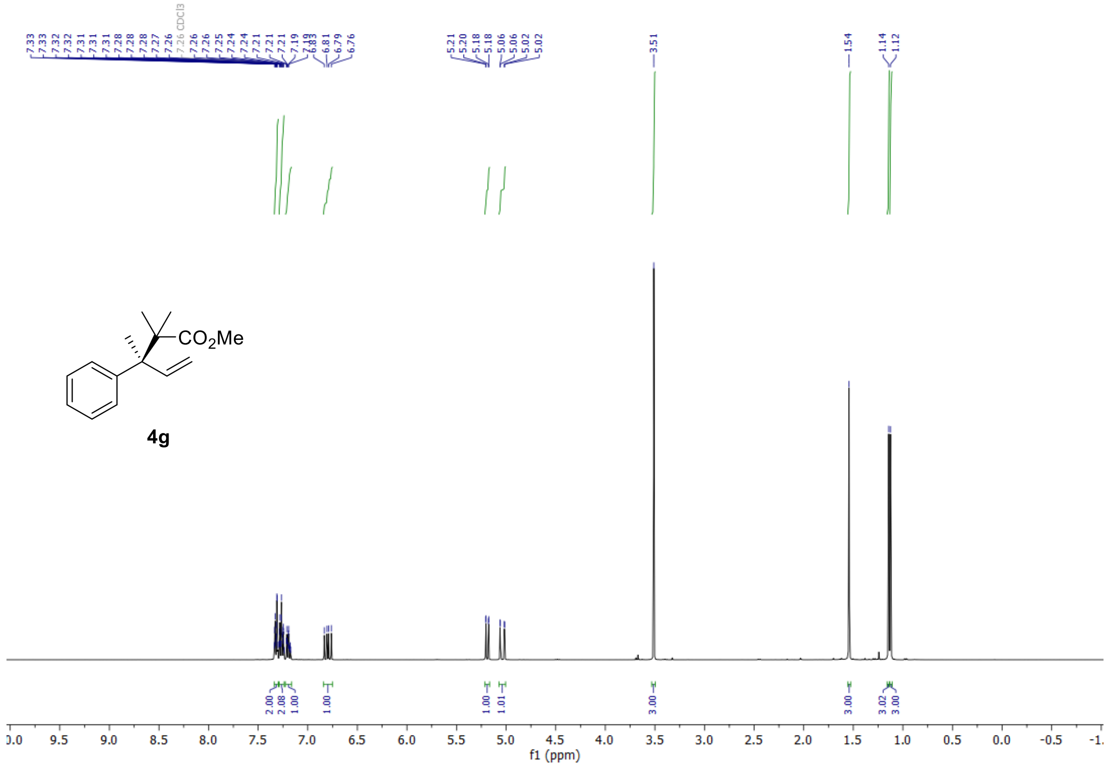

^{13}C NMR (101 MHz, $CDCl_3$) of **4g**

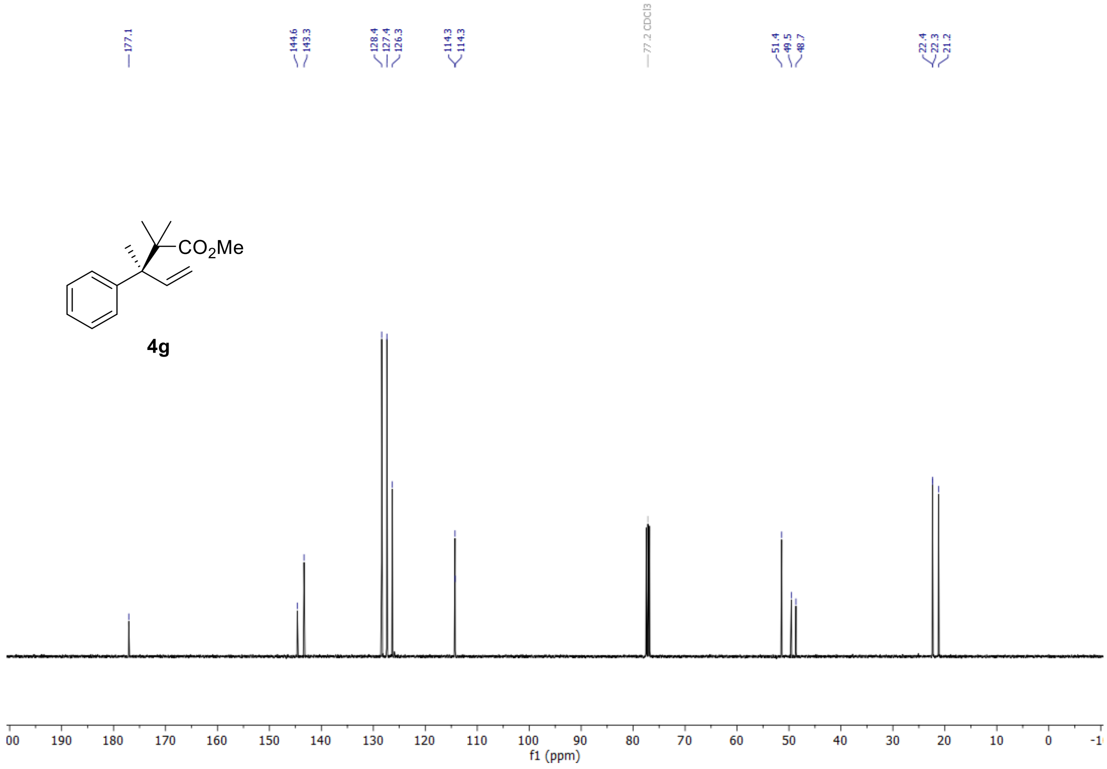

^{1}H NMR (400 MHz, $CDCl_3$) of **4h**

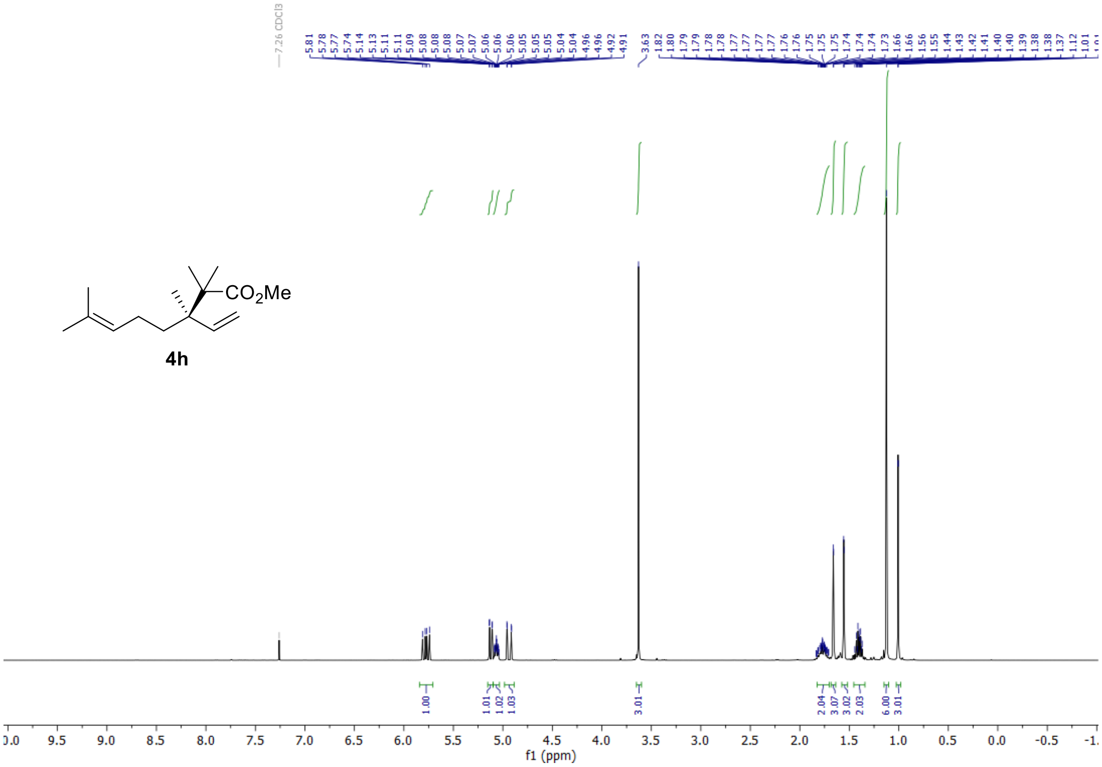

^{13}C NMR (101 MHz, $CDCl_3$) of **4h**

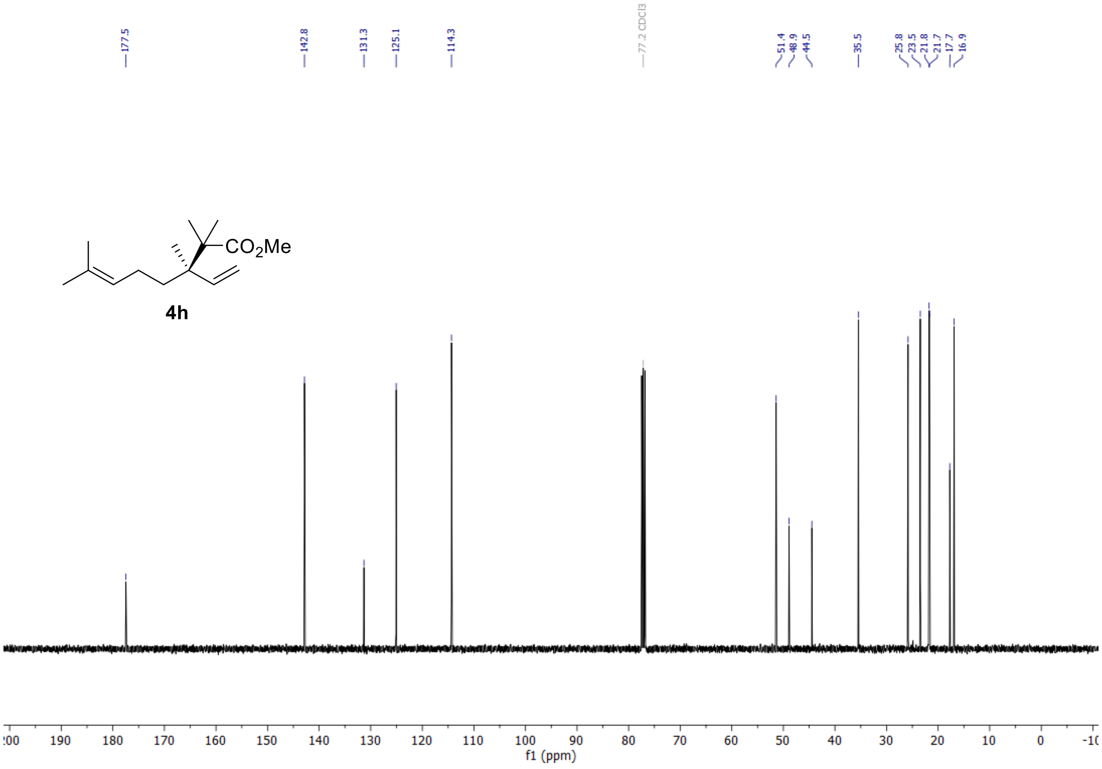

^{1}H NMR (400 MHz, $CDCl_3$) of **SI-3**

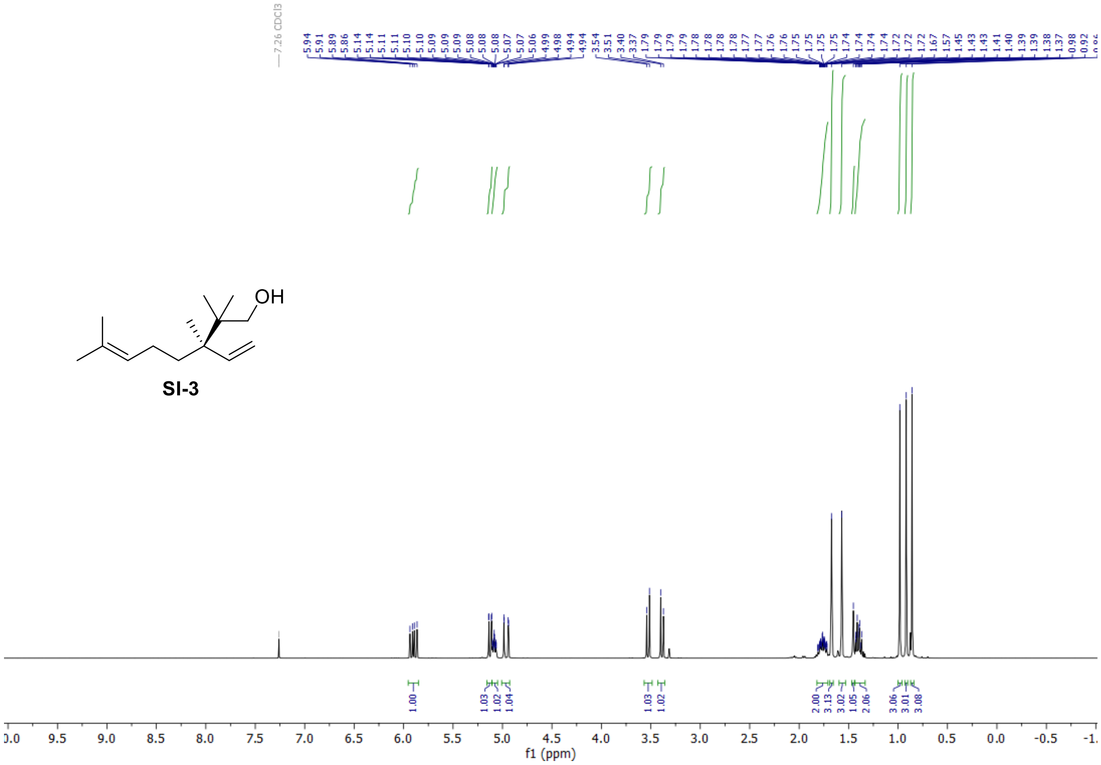

^{13}C NMR (101 MHz, $CDCl_3$) of **SI-3**

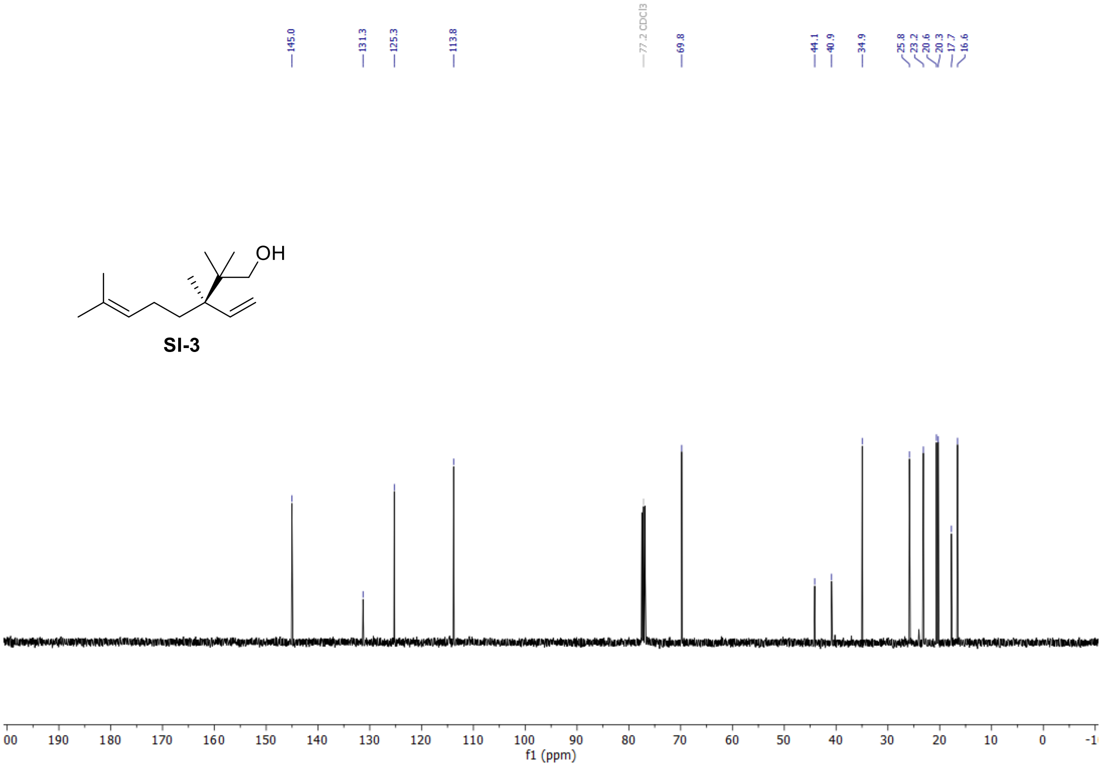

^{1}H NMR (400 MHz, $CDCl_3$) of **4i**

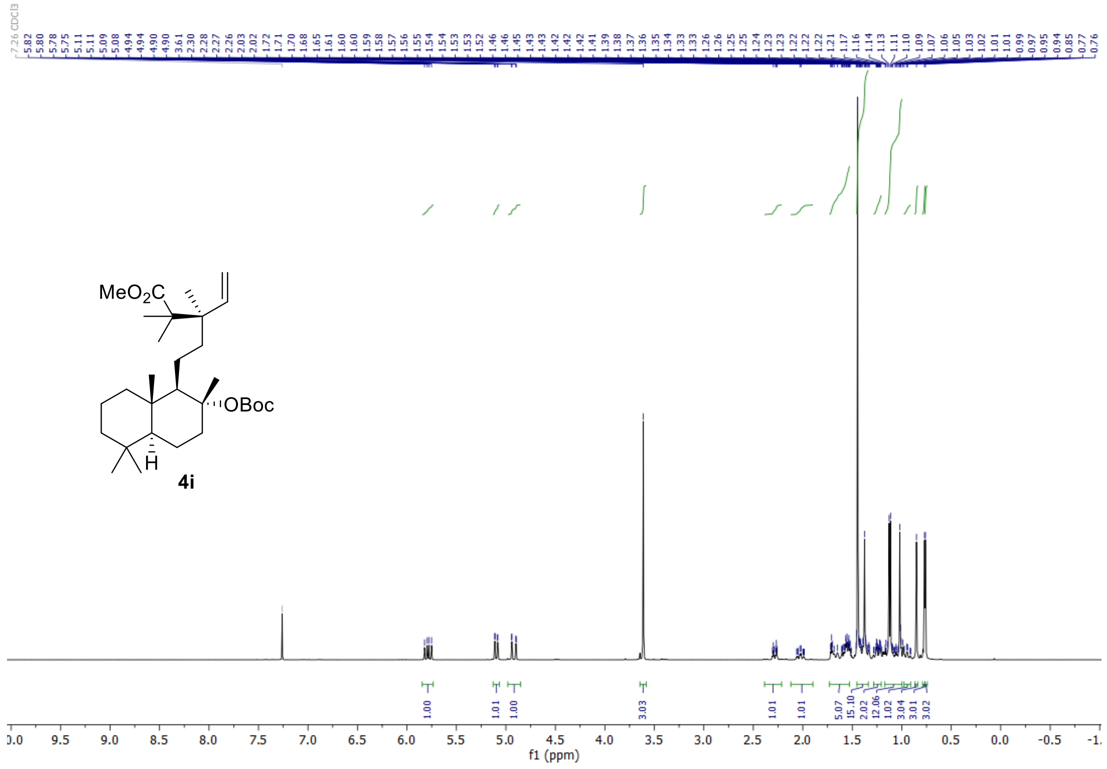

^{13}C NMR (101 MHz, $CDCl_3$) of **4i**

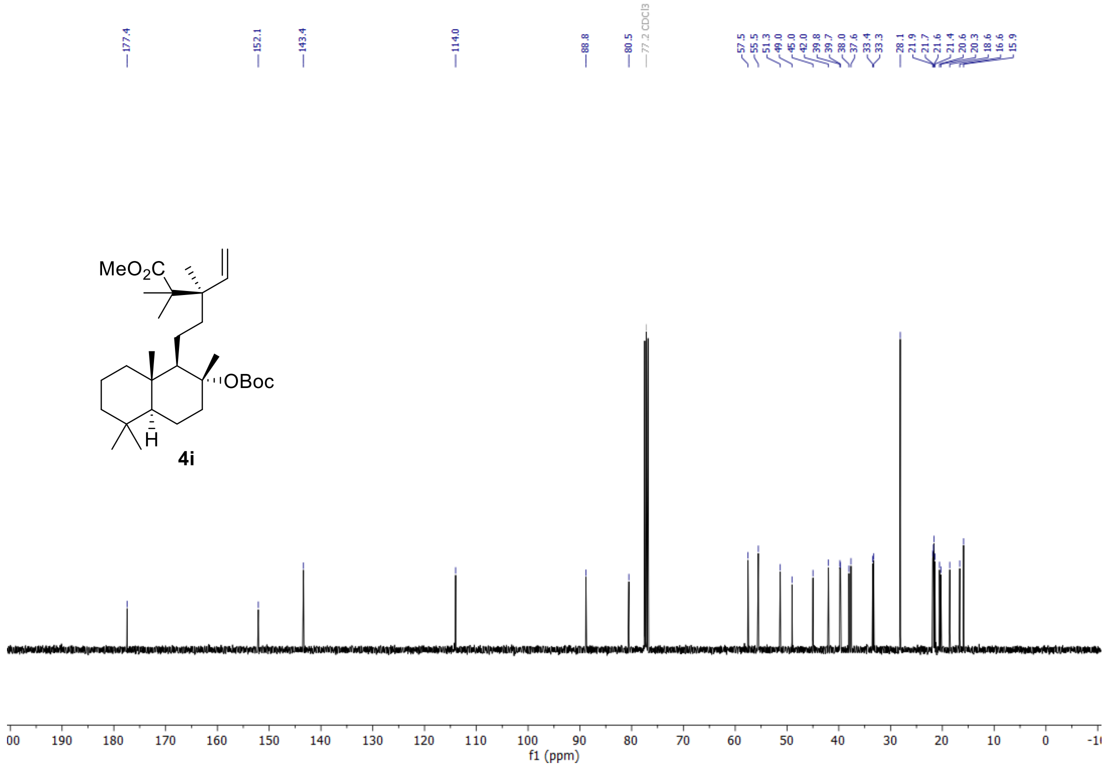

^{1}H NMR (400 MHz, $CDCl_3$) of **4j**

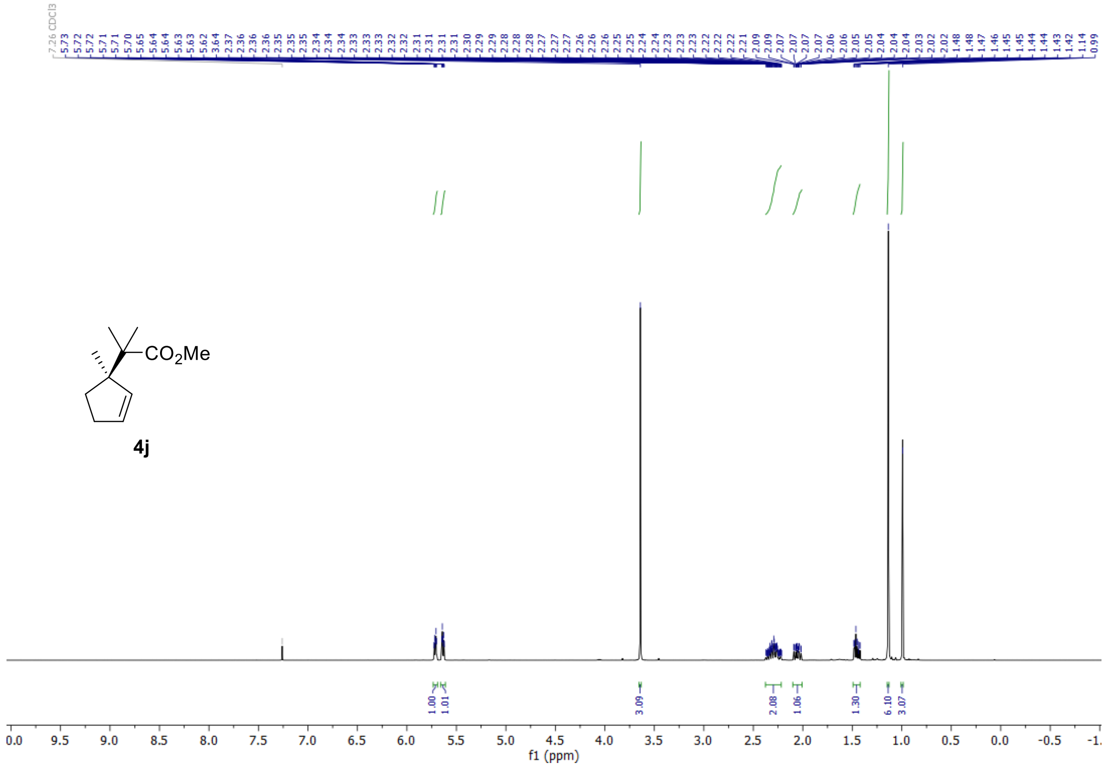

^{13}C NMR (101 MHz, $CDCl_3$) of **4j**

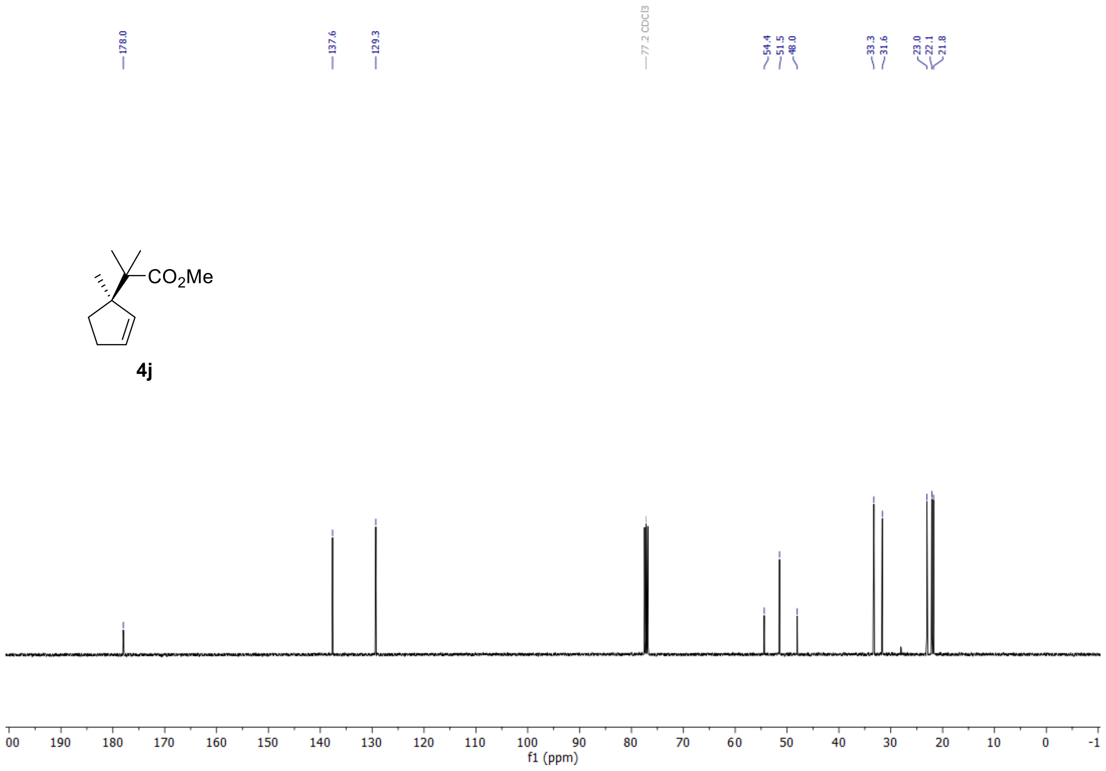

^{1}H NMR (400 MHz, $CDCl_3$) of **SI-4**

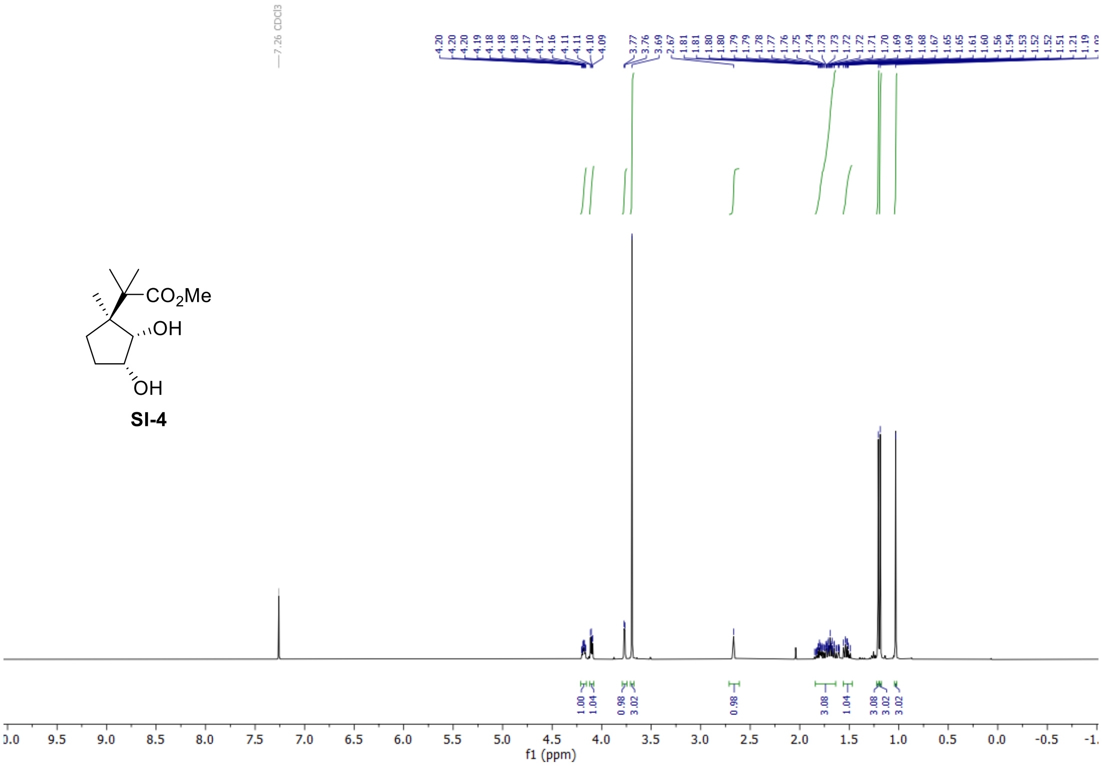

^{13}C NMR (101 MHz, $CDCl_3$) of **SI-4**

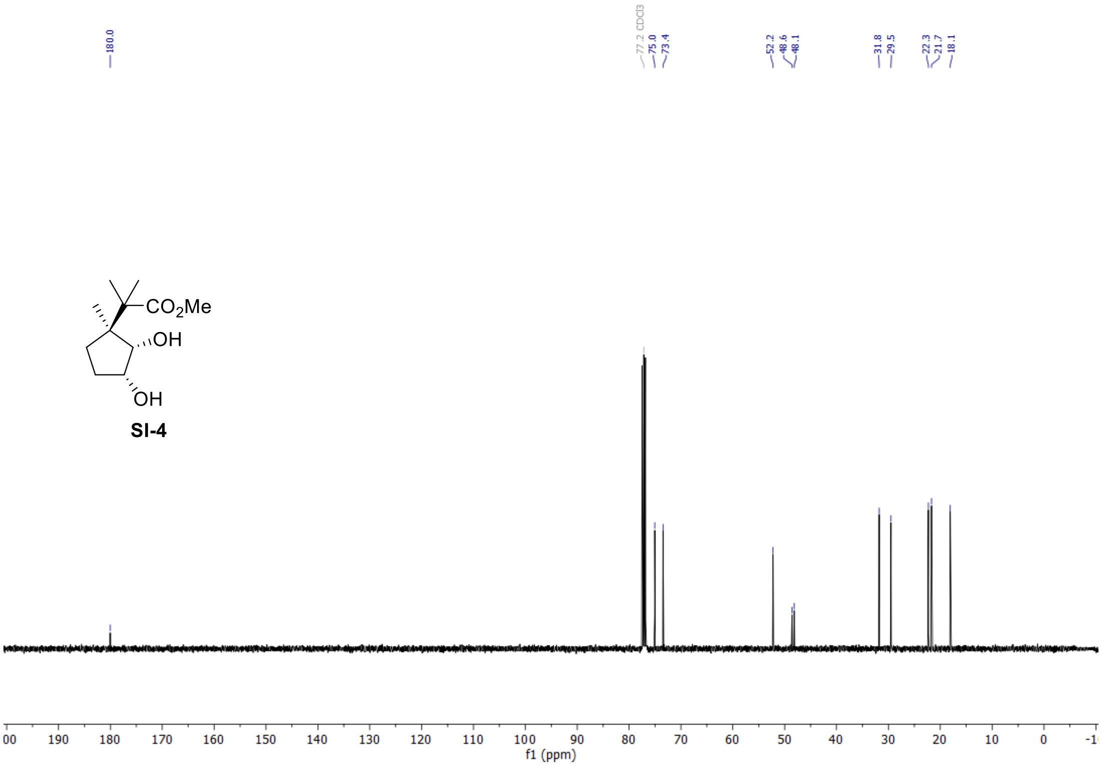

^{1}H NMR (400 MHz, $CDCl_3$) of **4k**

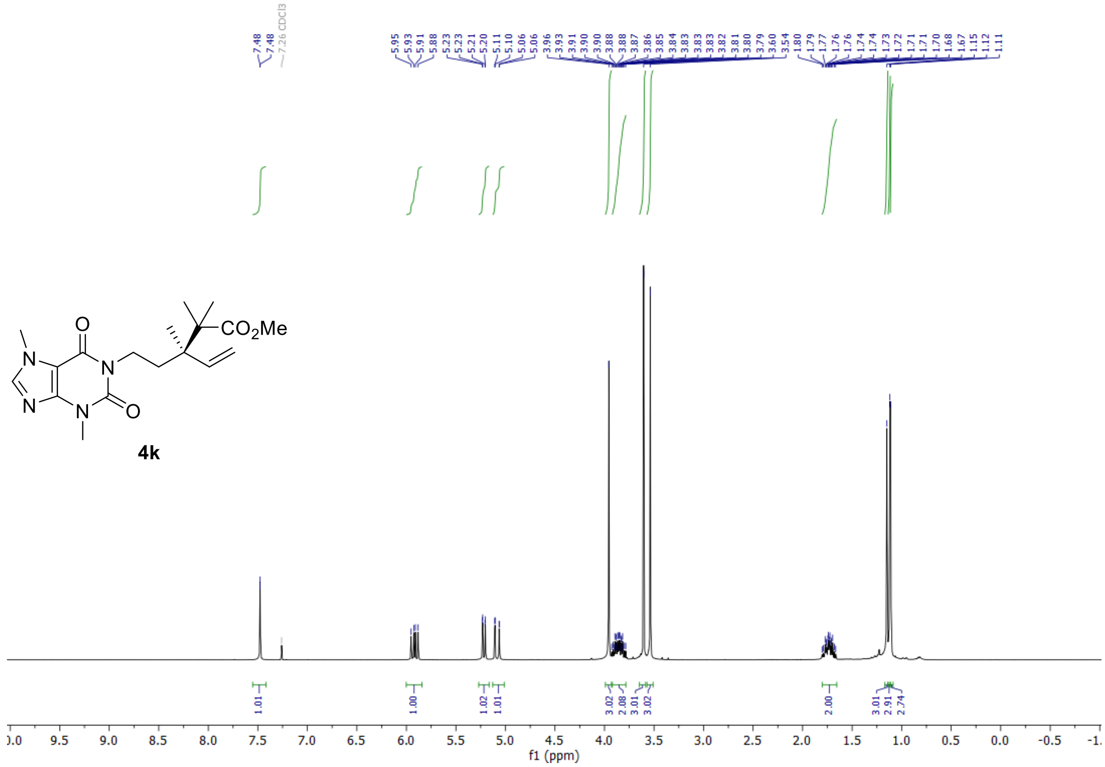

^{13}C NMR (101 MHz, $CDCl_3$) of **4k**

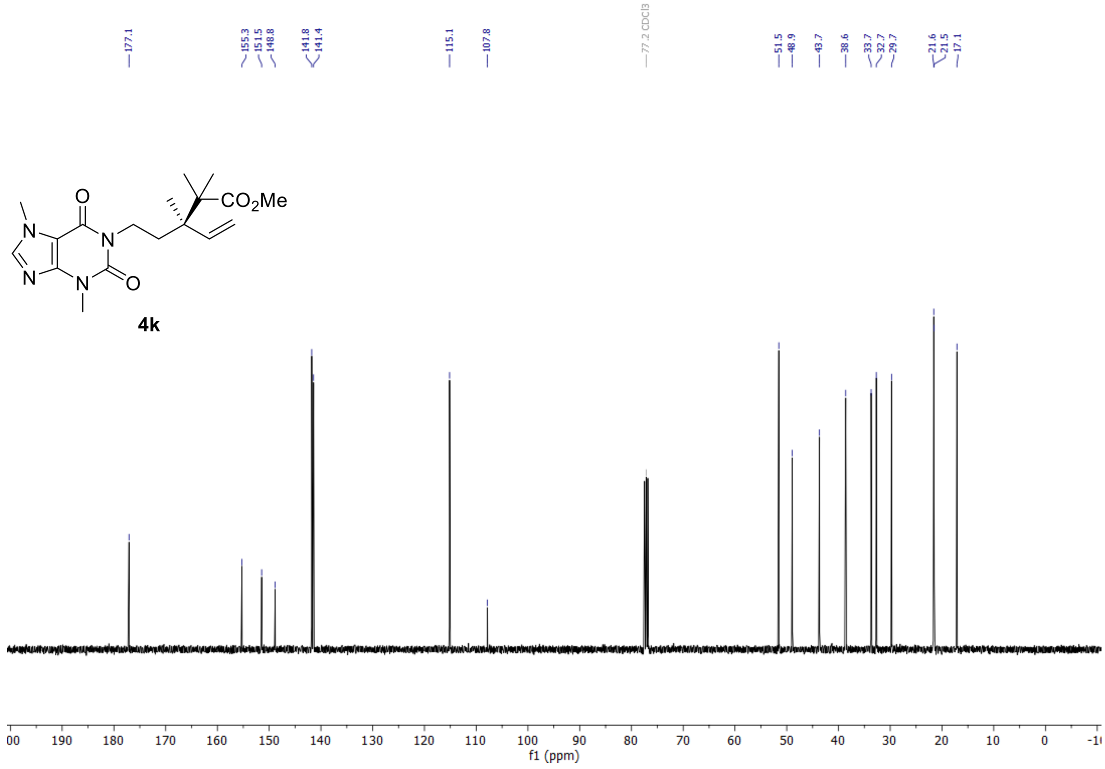

^{1}H NMR (500 MHz, $CDCl_3$) of **4l**

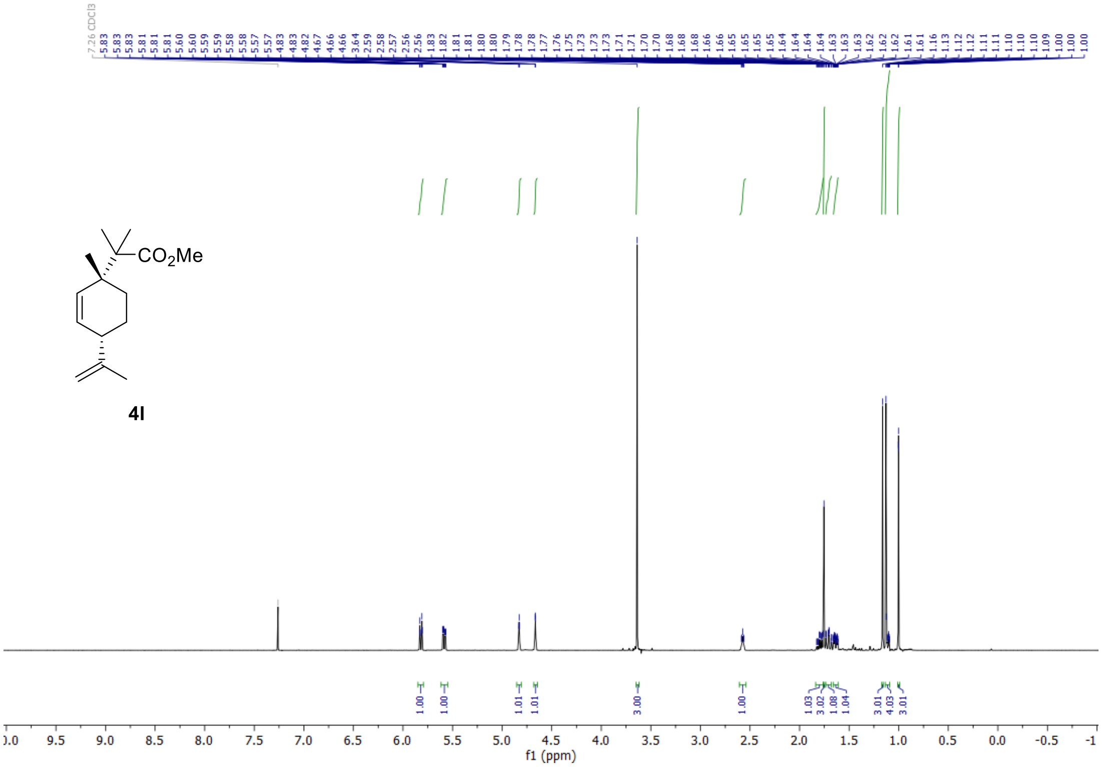

^{13}C NMR (126 MHz, $CDCl_3$) of **4l**

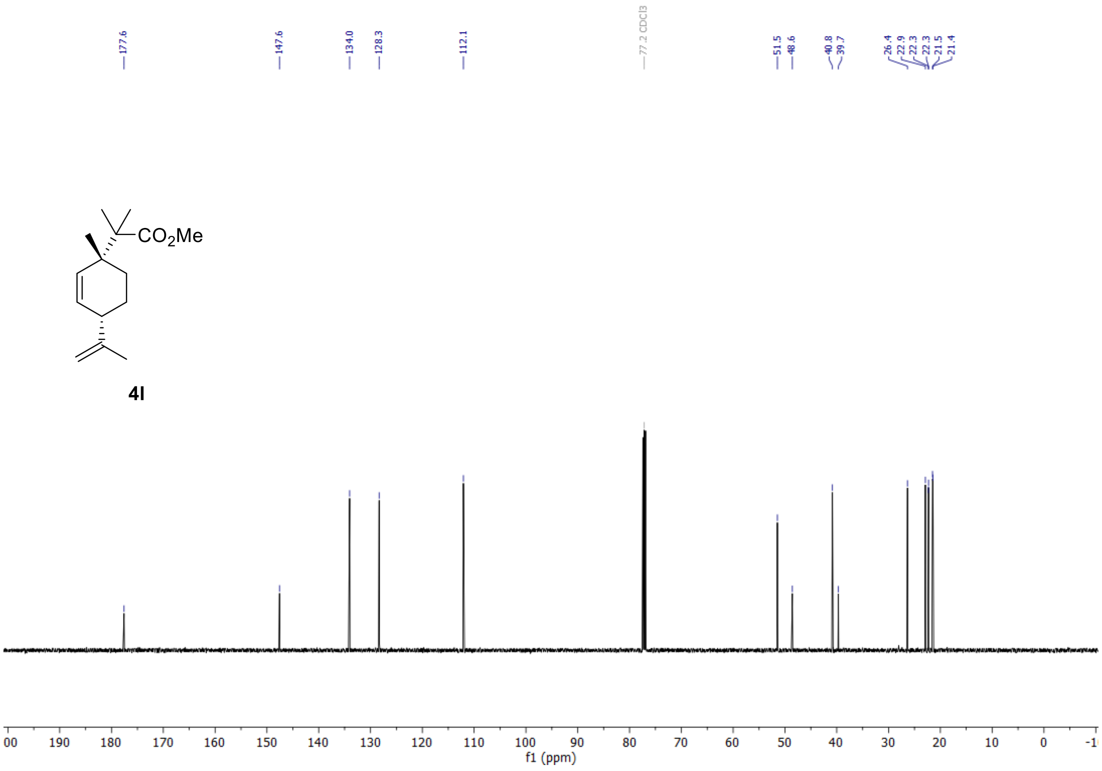

^{1}H NMR (400 MHz, $CDCl_3$) of the 1:2.8:1.3 mixture of **3o/3p/3q**

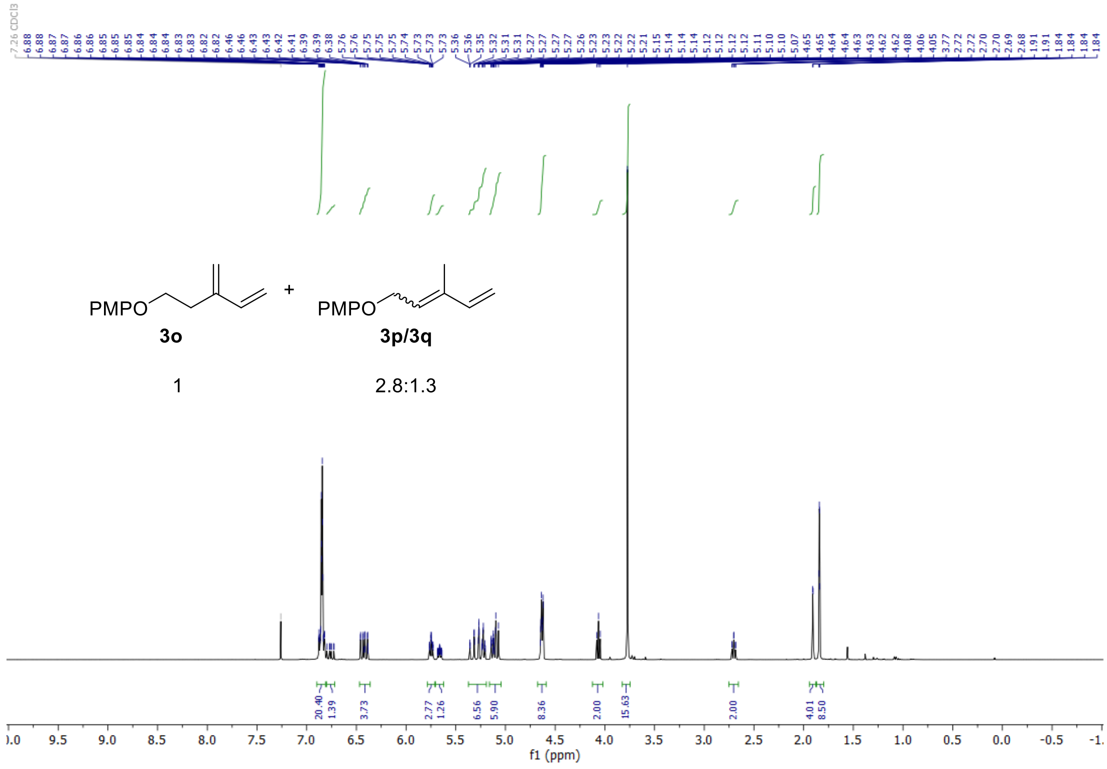

^{13}C NMR (101 MHz, $CDCl_3$) of the 1:2.8:1.3 mixture of **3o/3p/3q**

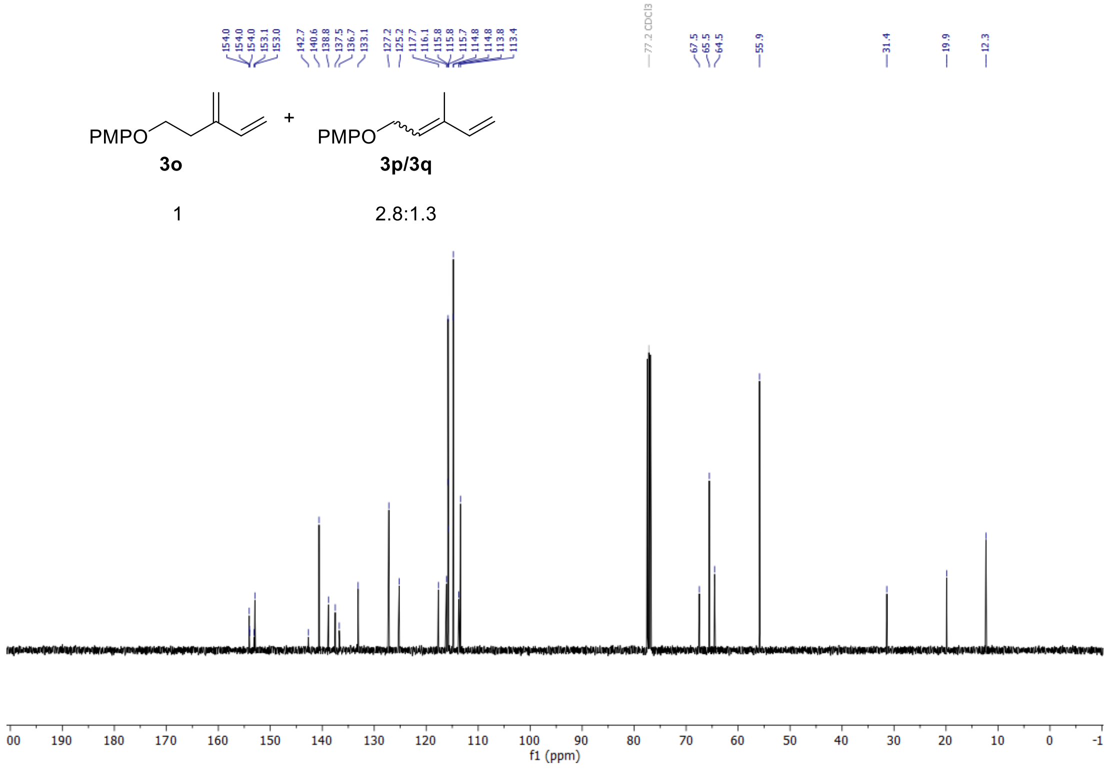

HSQC (400 MHz/101 MHz, $CDCl_3$) of the 1:2.8:1.3 mixture of **3o/3p/3q**

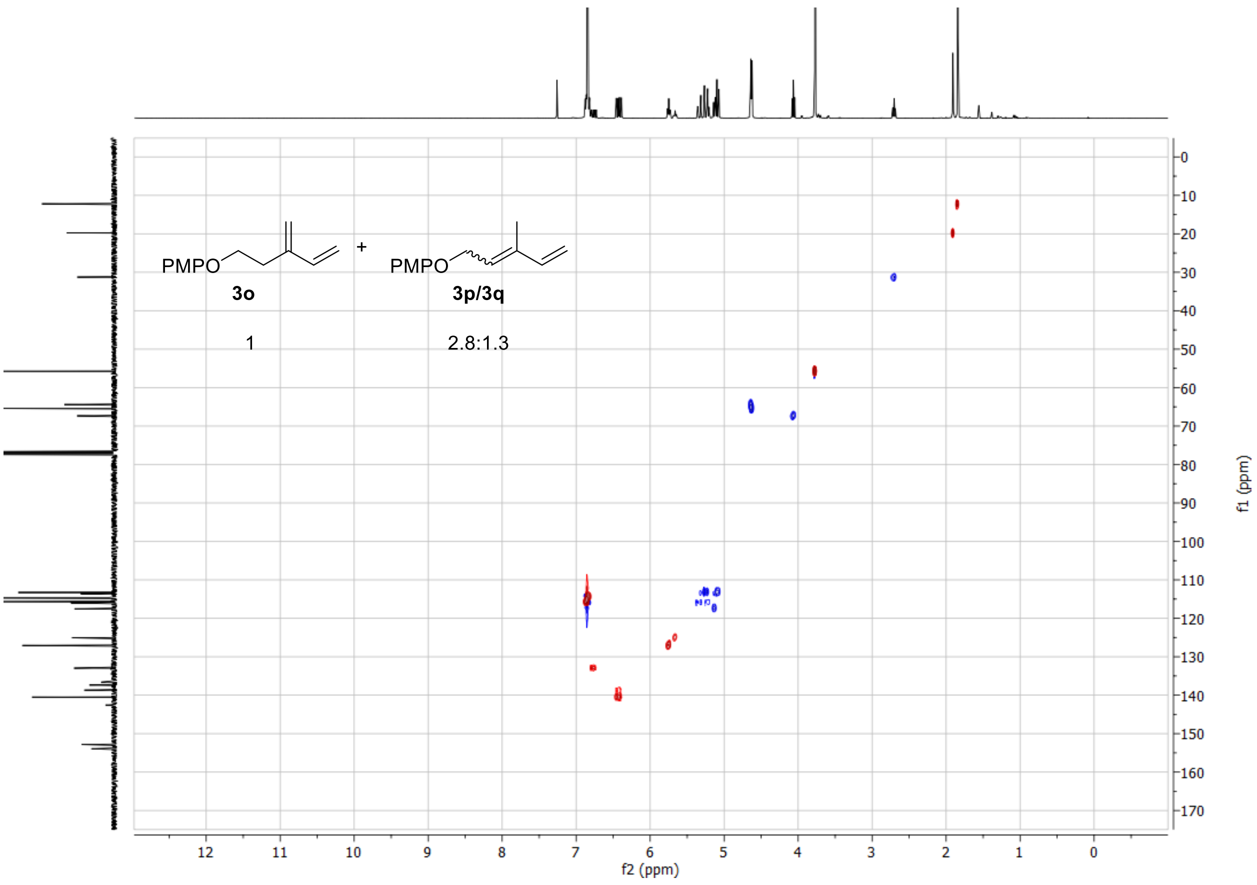

^{1}H NMR (400 MHz, $CDCl_3$) of **SI-5**

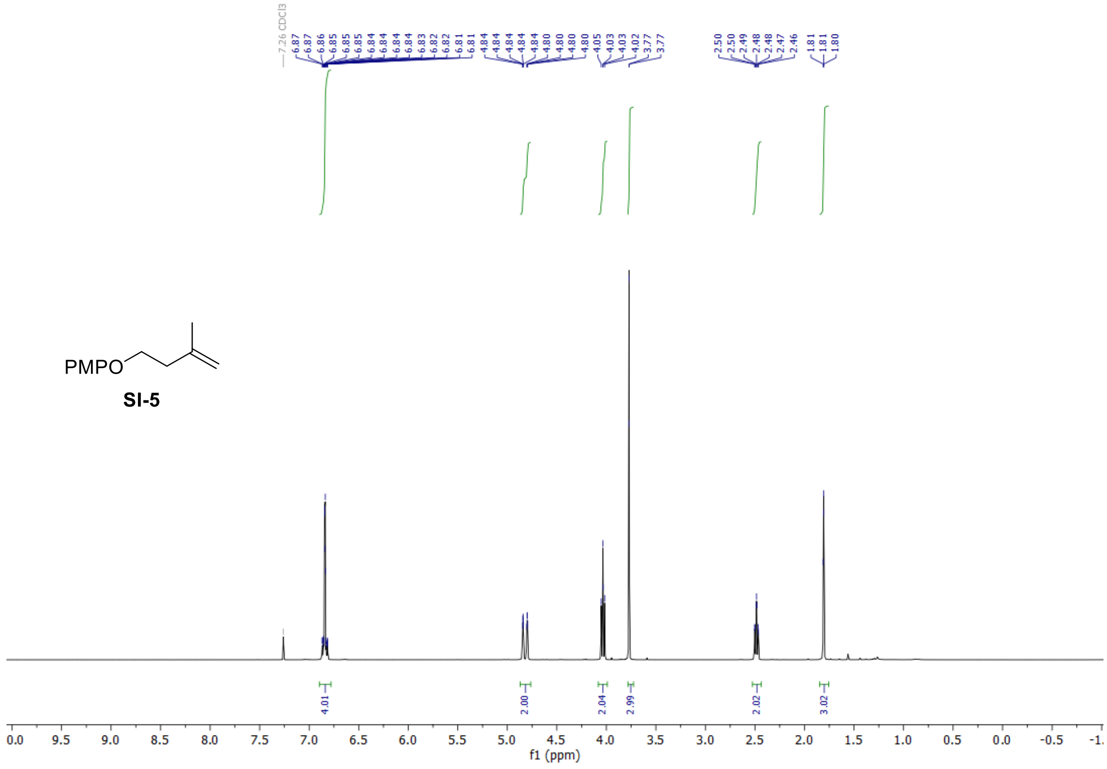

^{13}C NMR (101 MHz, $CDCl_3$) of **SI-5**

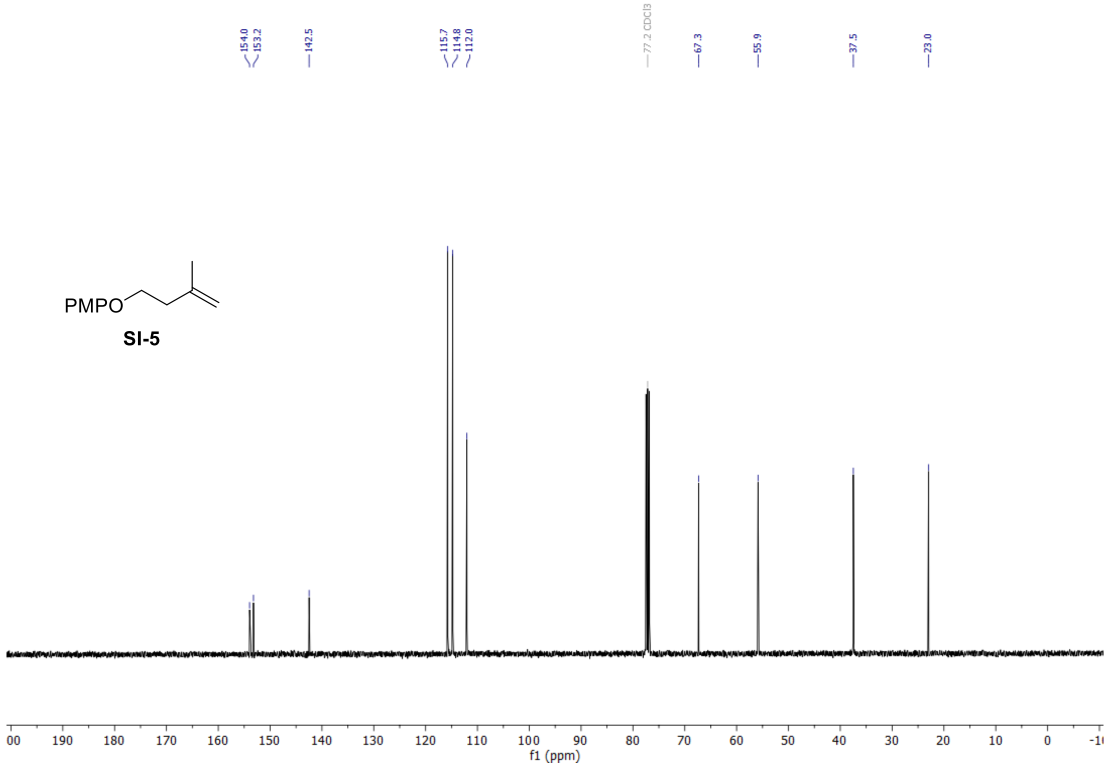

^{1}H NMR (400 MHz, $CDCl_3$) of **(–)-SI-6**

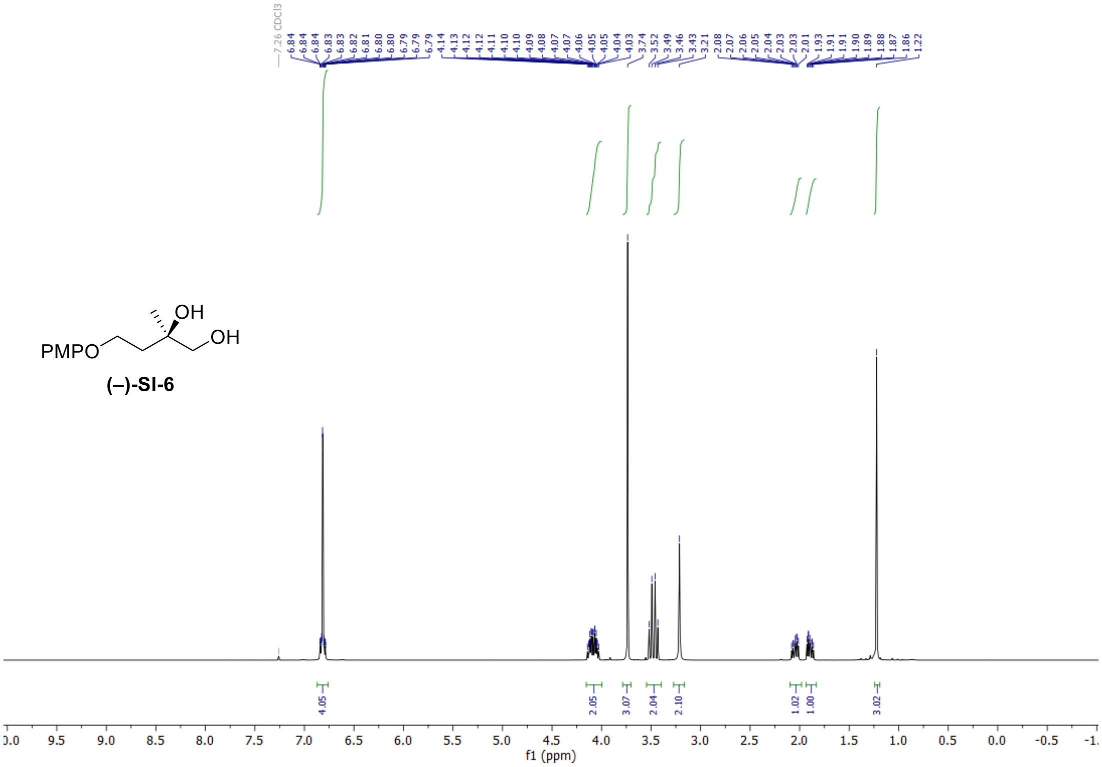

^{13}C NMR (101 MHz, $CDCl_3$) of **(–)-SI-6**

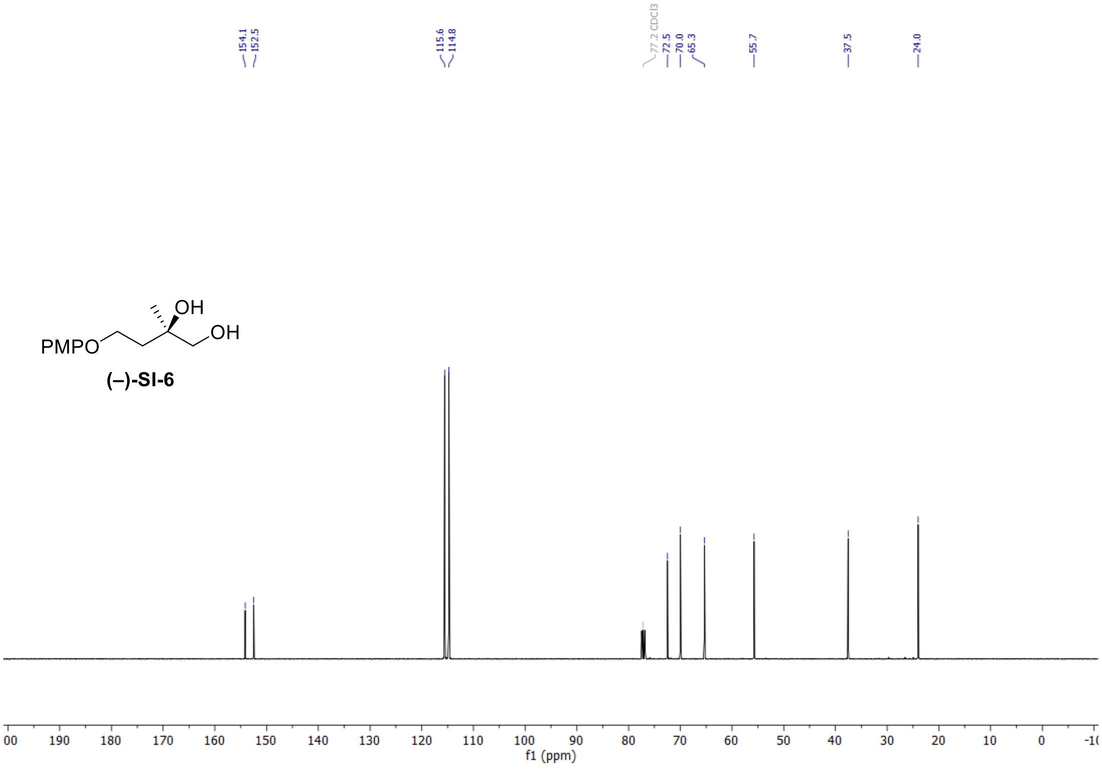

^{1}H NMR (400 MHz, $CDCl_3$) of **SI-7**

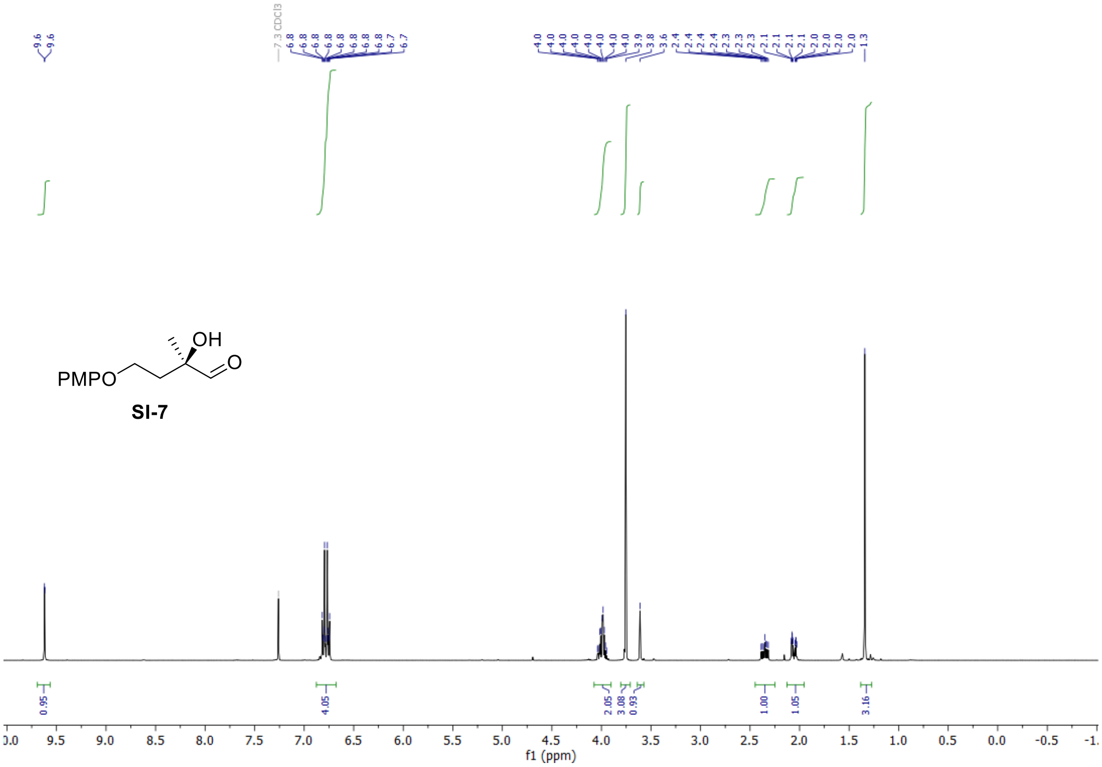

^{13}C NMR (101 MHz, $CDCl_3$) of **SI-7**

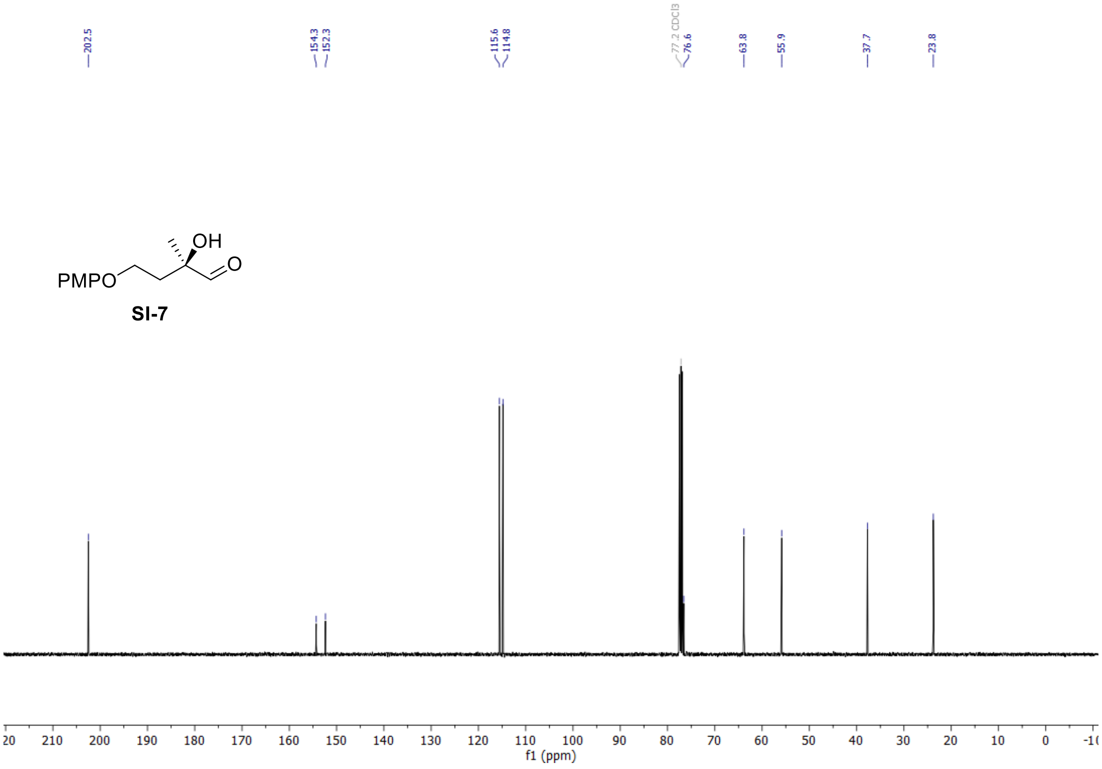

^{1}H NMR (400 MHz, $CDCl_3$) of **SI-8**

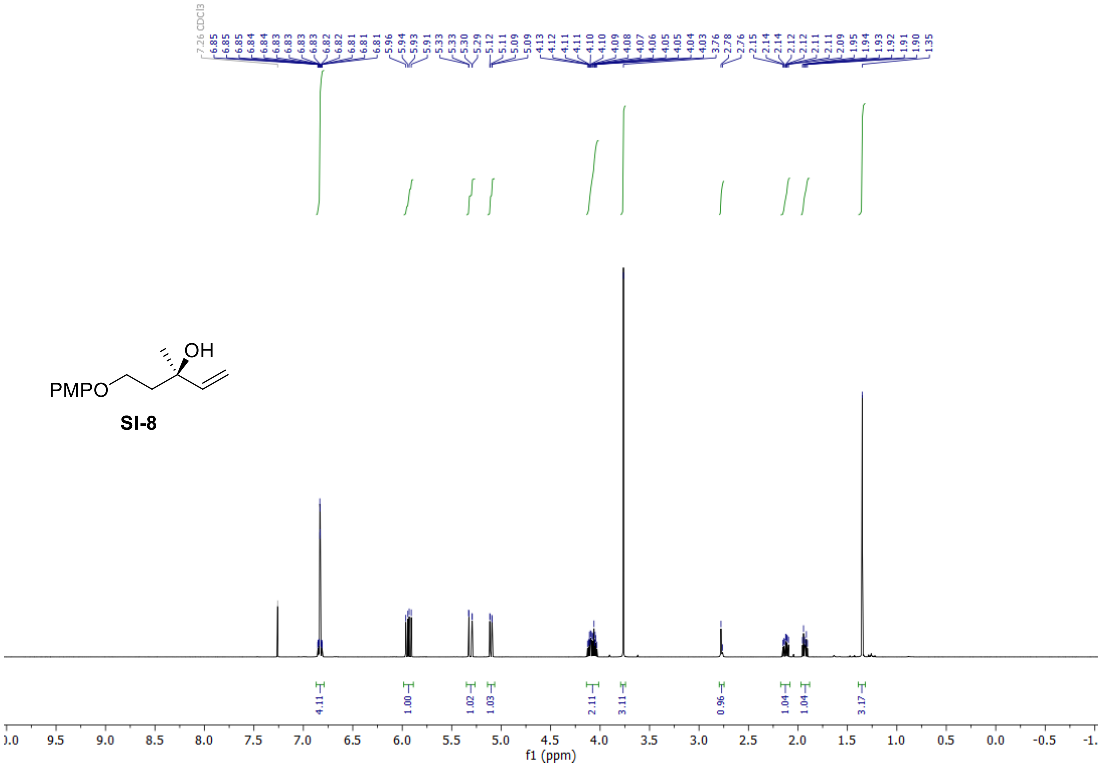

^{13}C NMR (101 MHz, $CDCl_3$) of **SI-8**

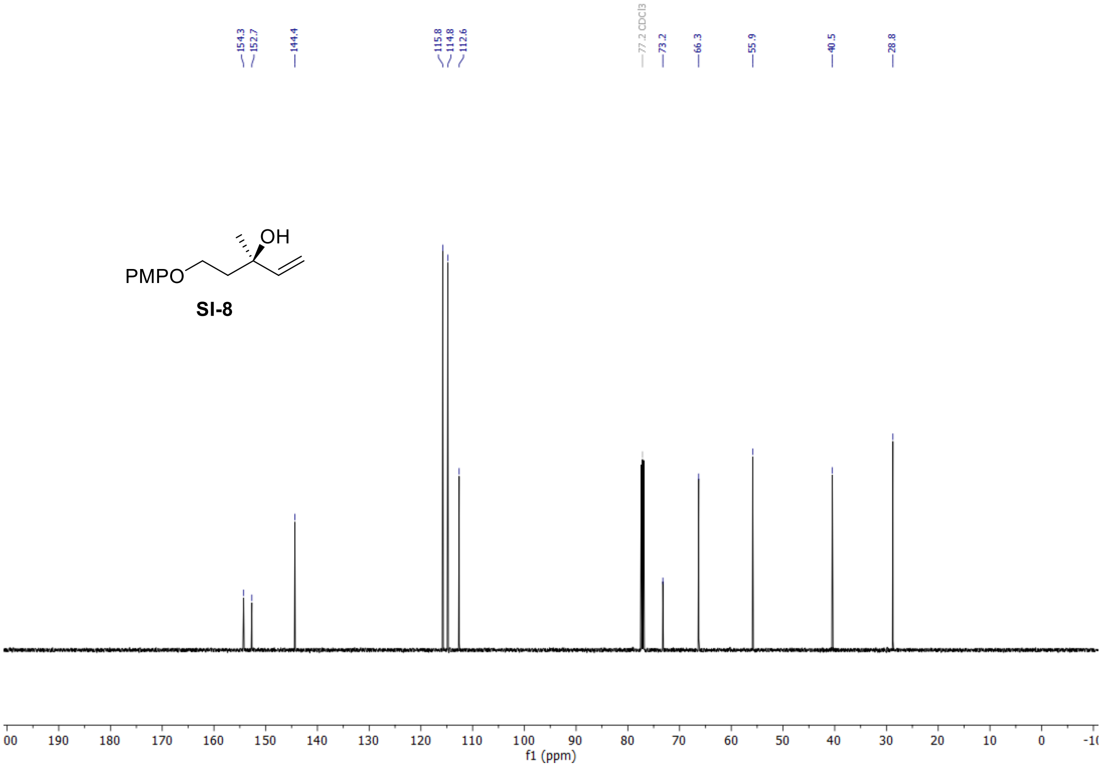

^{1}H NMR (400 MHz, $CDCl_3$) of **SI-9**

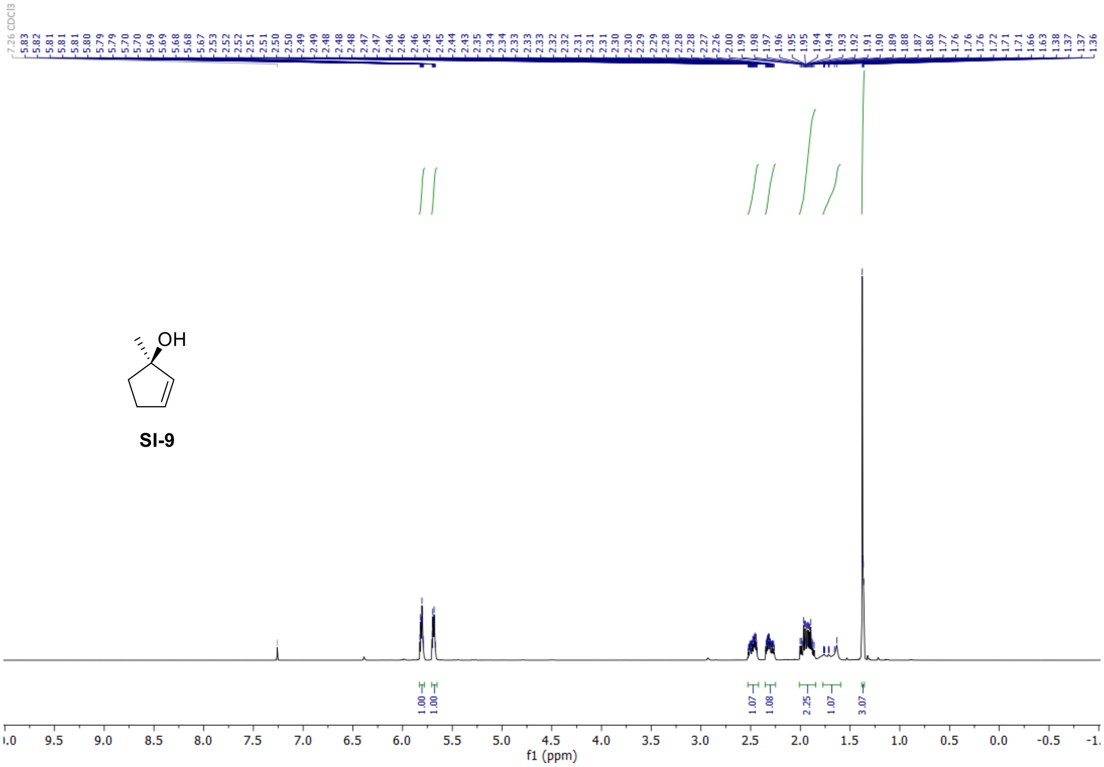

^{13}C NMR (101 MHz, $CDCl_3$) of **SI-9**

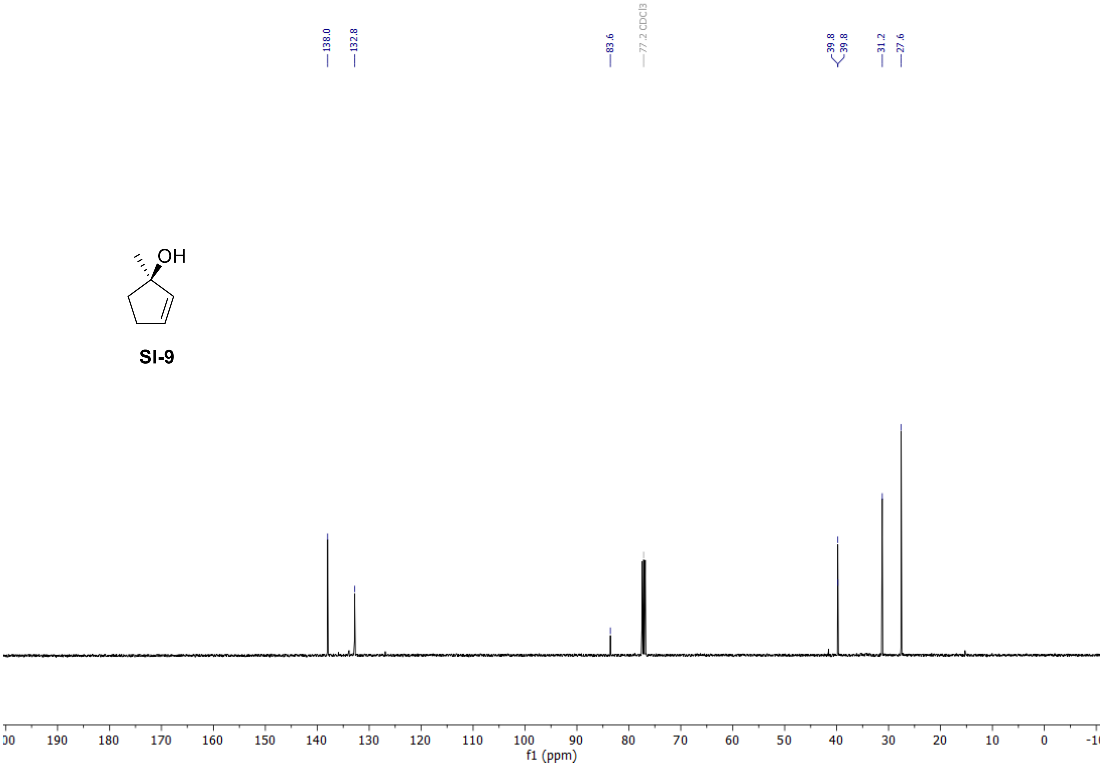

^{1}H NMR (400 MHz, $CDCl_3$) of **1a**

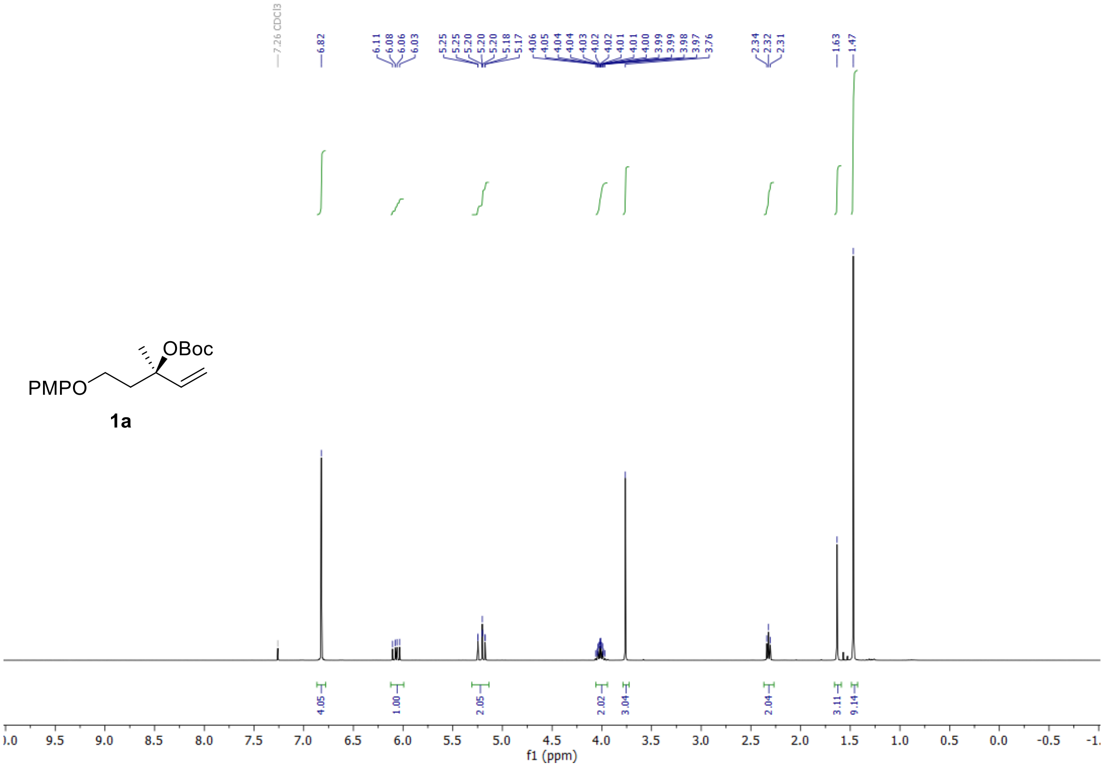

^{13}C NMR (101 MHz, $CDCl_3$) of **1a**

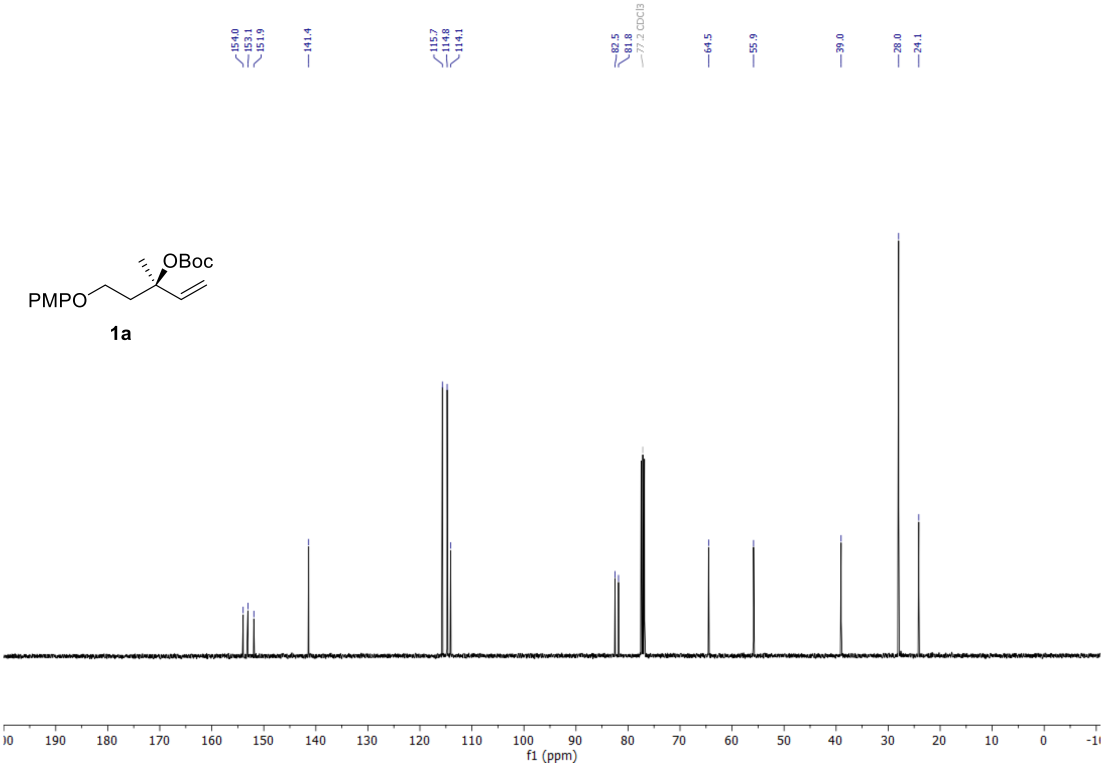

^{1}H NMR (400 MHz, $CDCl_3$) of **1n**

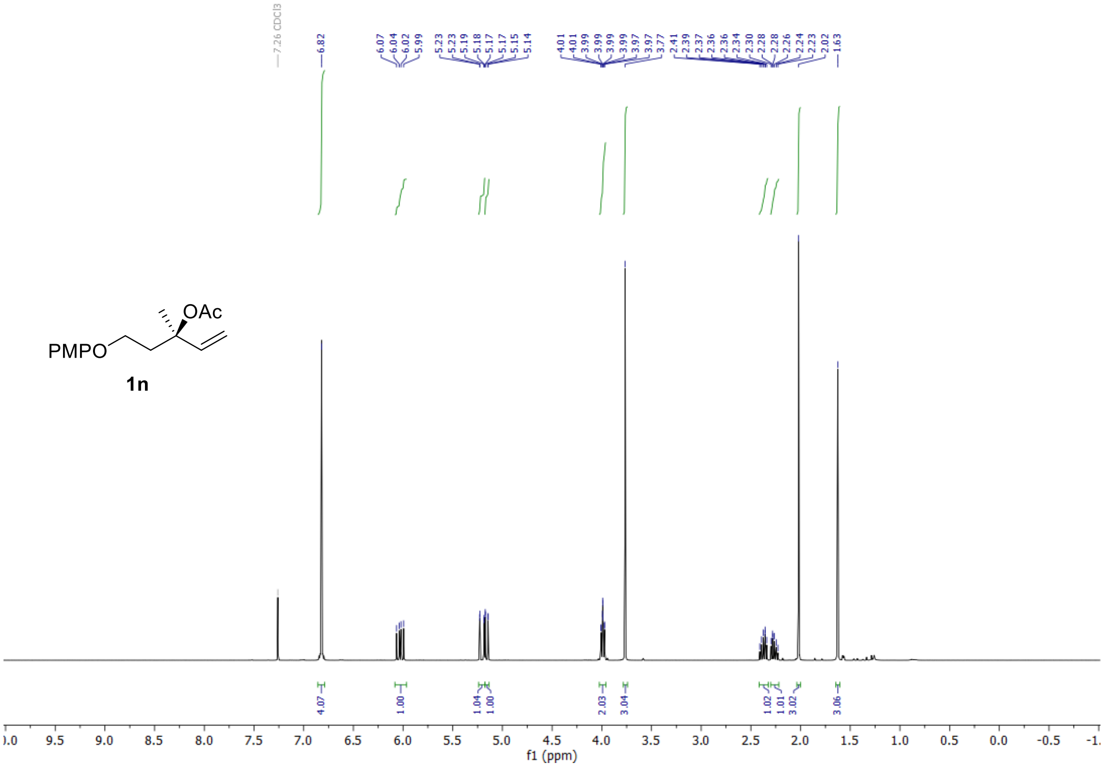

^{13}C NMR (101 MHz, $CDCl_3$) of **1n**

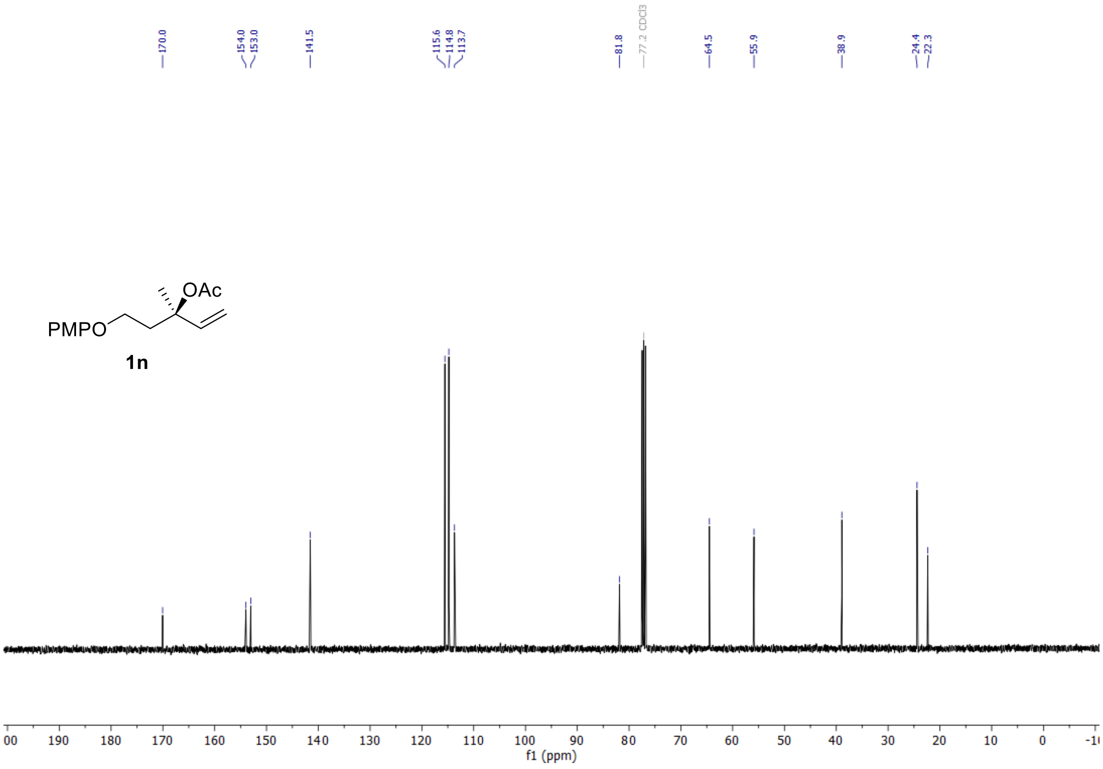

^{1}H NMR (400 MHz, $CDCl_3$) of **1h**

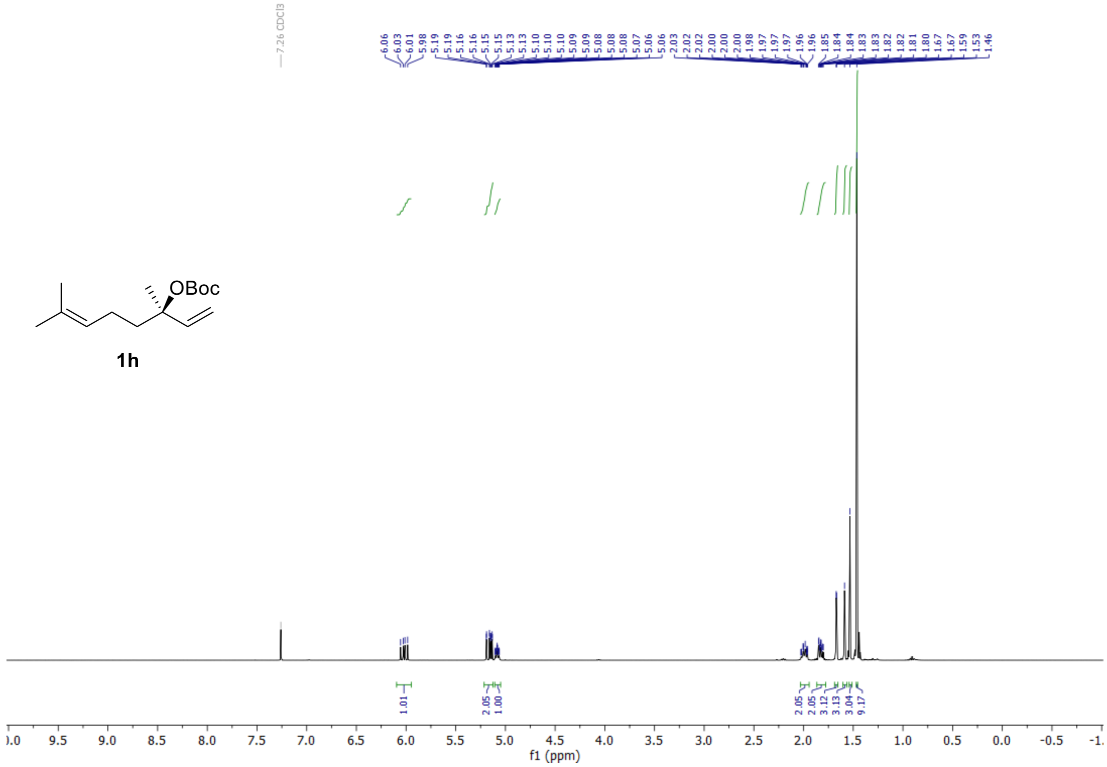

^{13}C NMR (101 MHz, $CDCl_3$) of **1h**

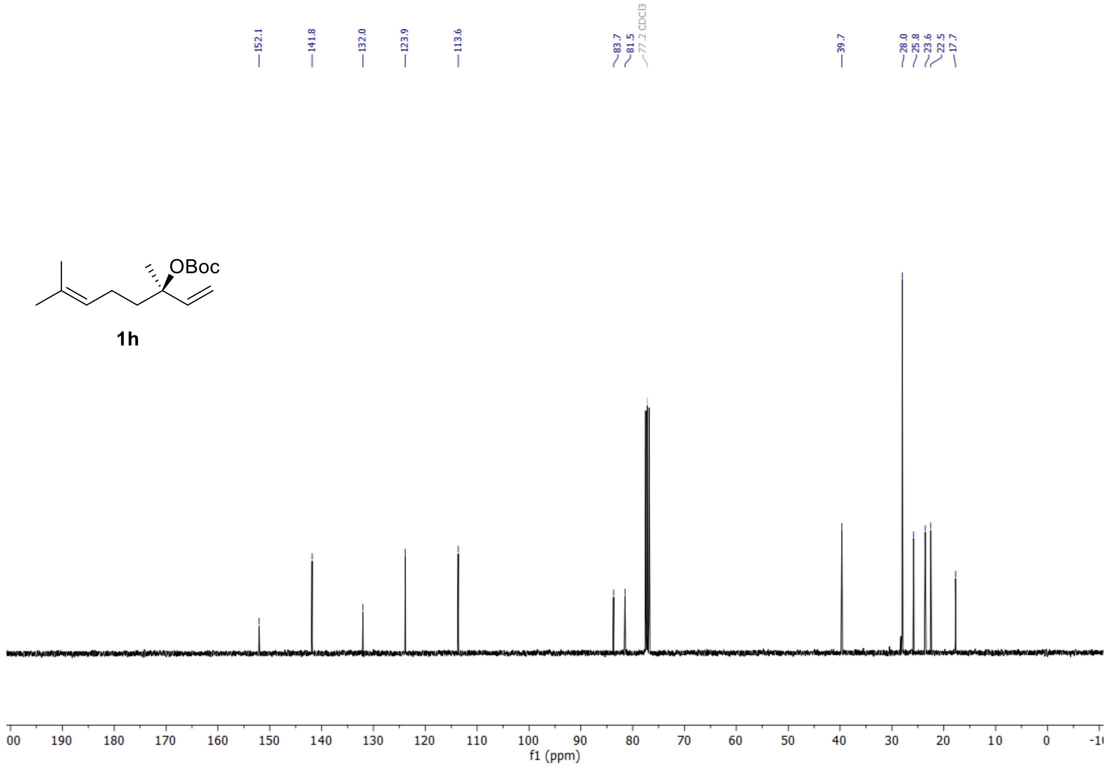

^{1}H NMR (400 MHz, $CDCl_3$) of **1g**

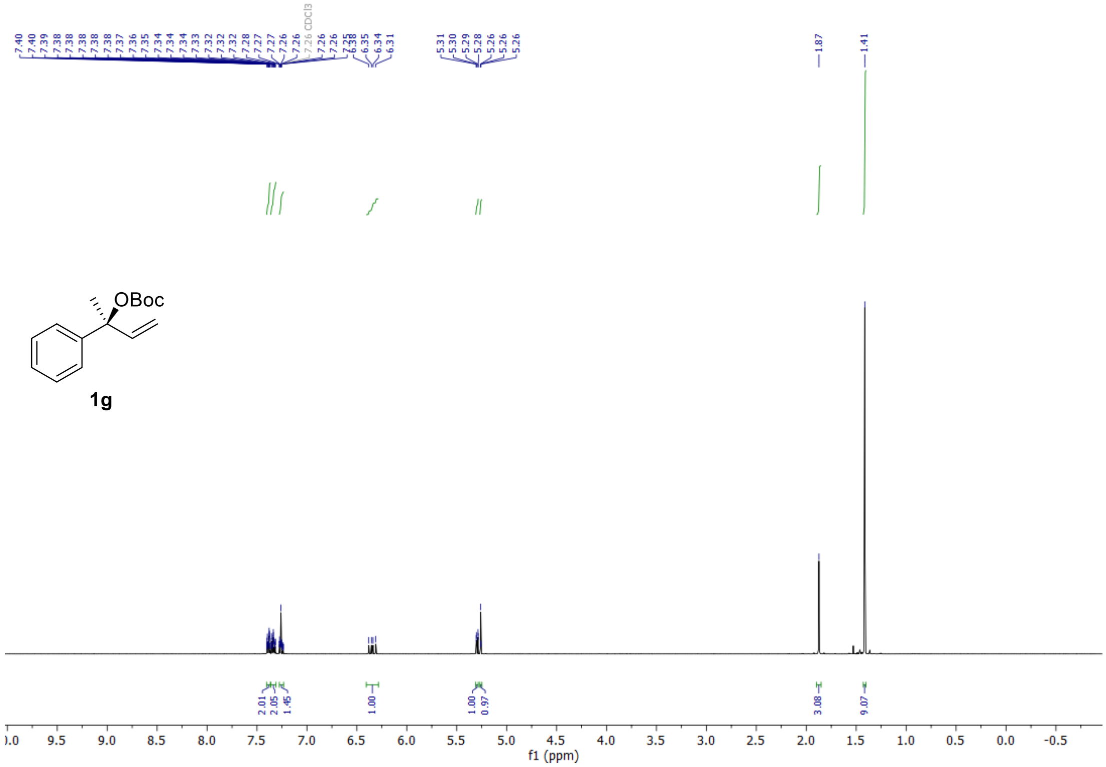

^{1}H NMR (400 MHz, $CDCl_3$) of **SI-14**

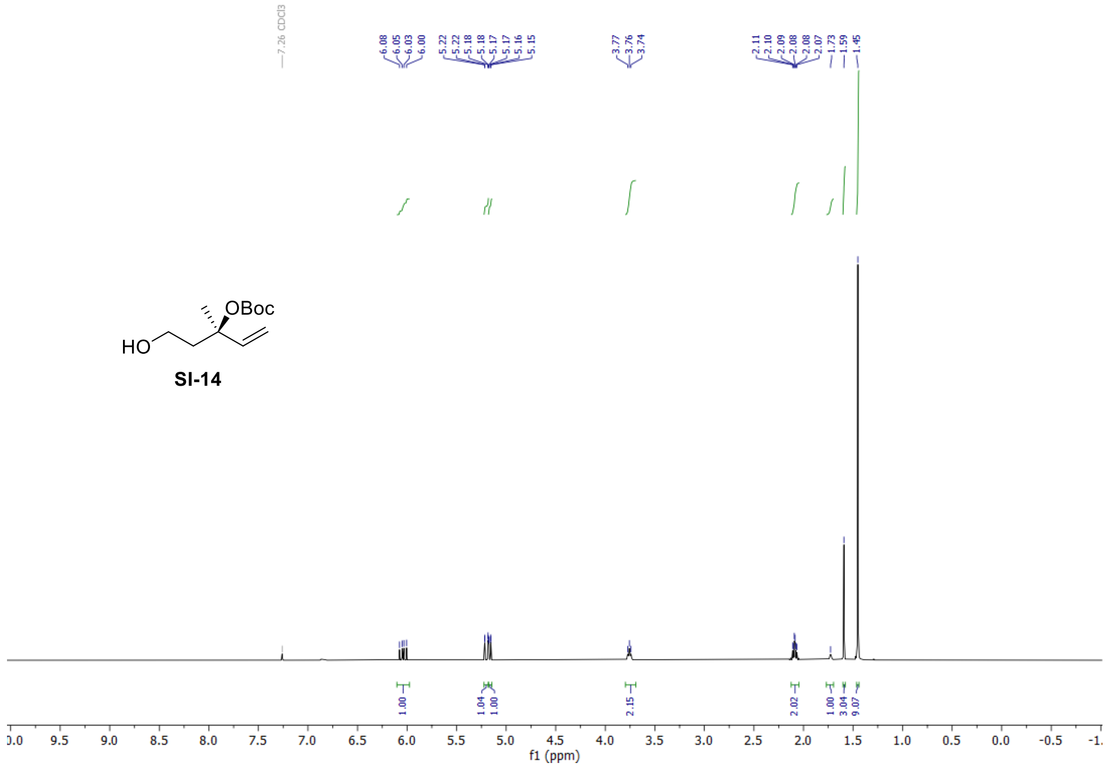

^{13}C NMR (101 MHz, $CDCl_3$) of **SI-14**

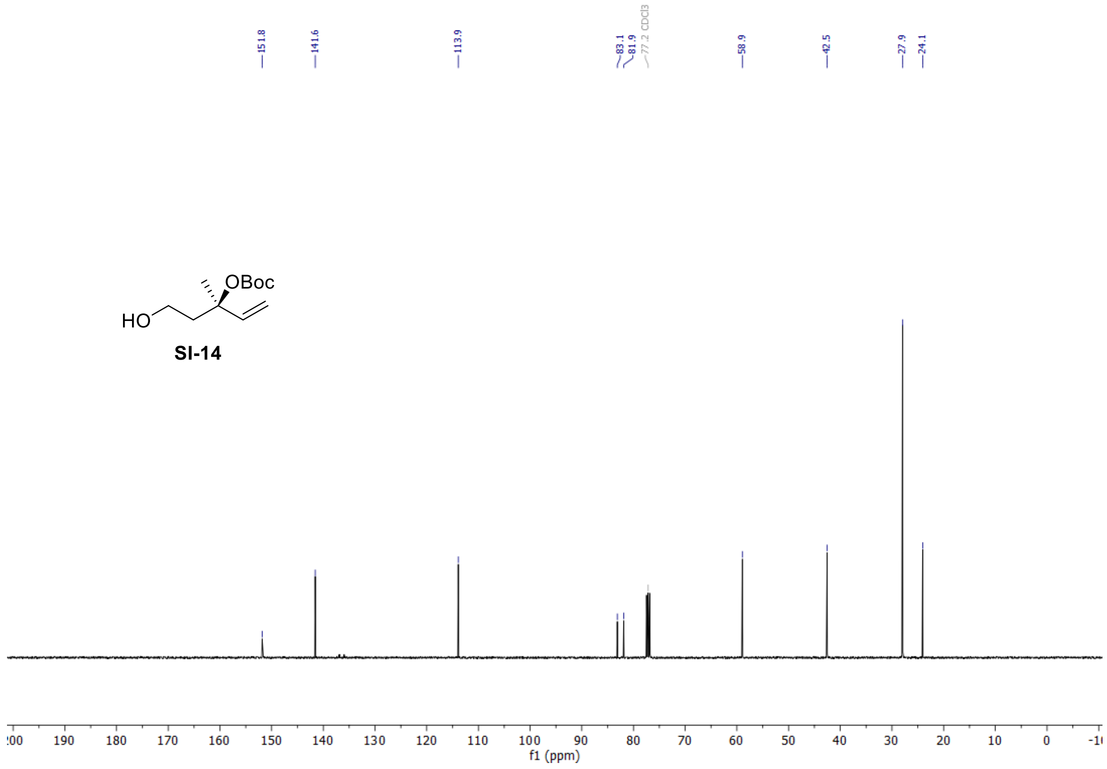

^{1}H NMR (400 MHz, $CDCl_3$) of **1b**

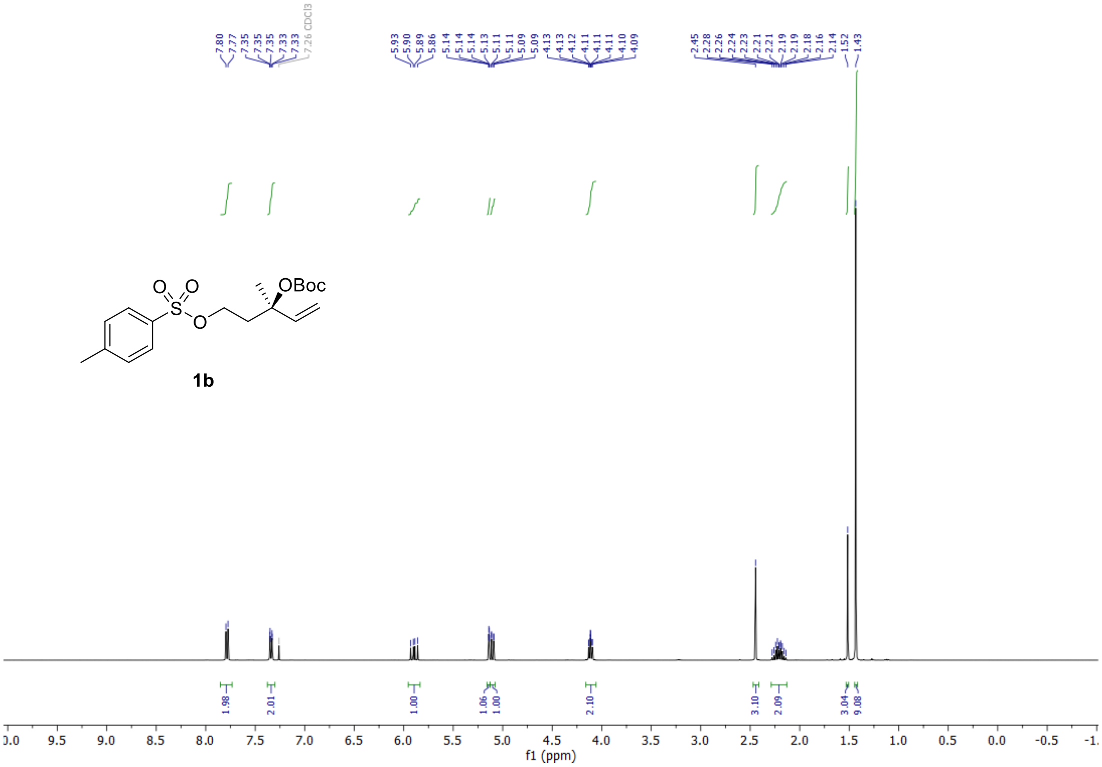

^{13}C NMR (101 MHz, $CDCl_3$) of **1b**

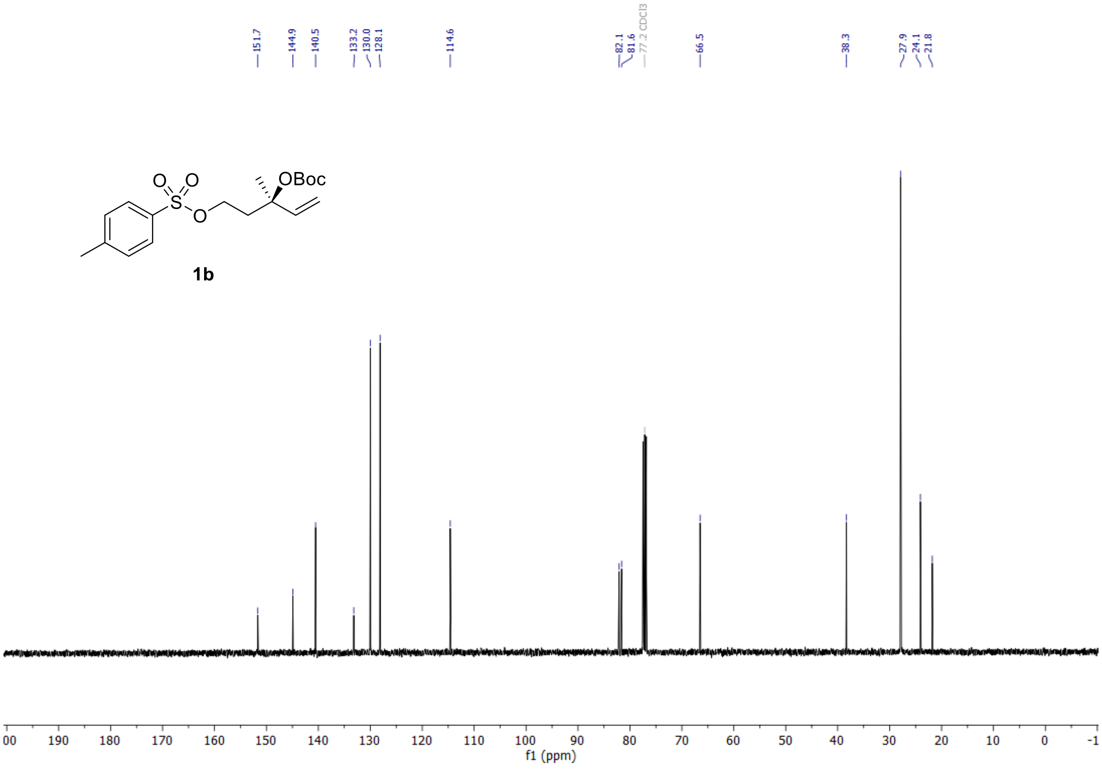

^{1}H NMR (400 MHz, $CDCl_3$) of **1c**

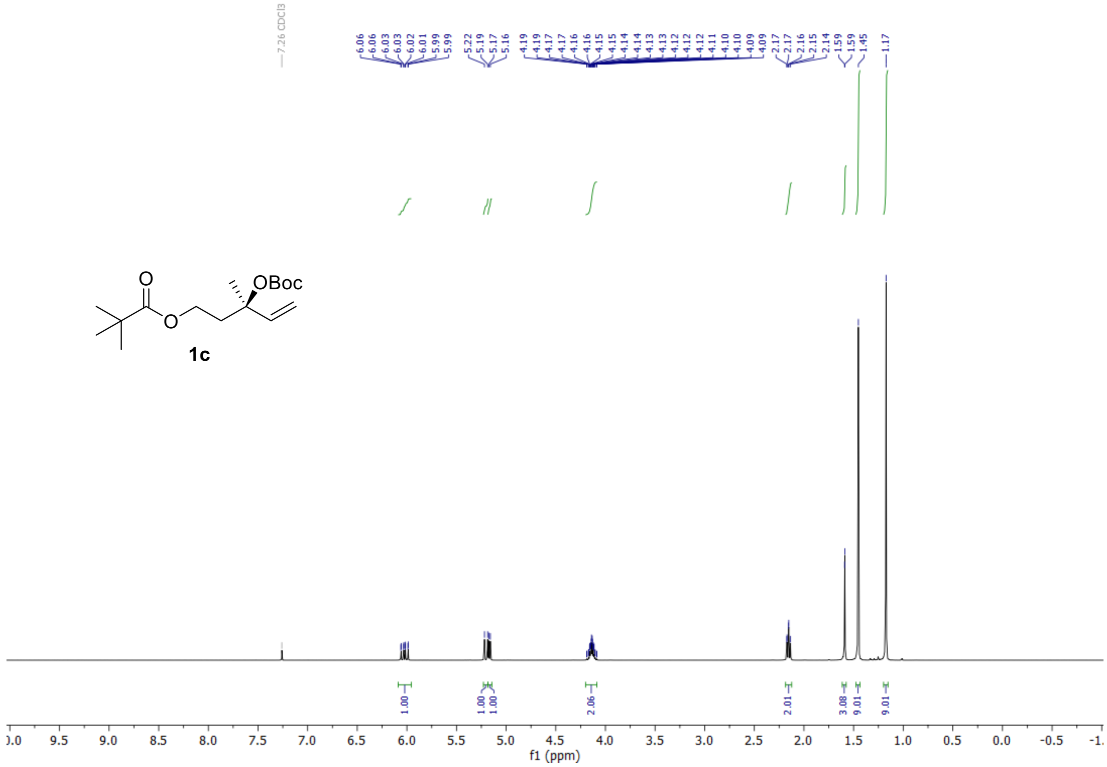

^{13}C NMR (101 MHz, $CDCl_3$) of **1c**

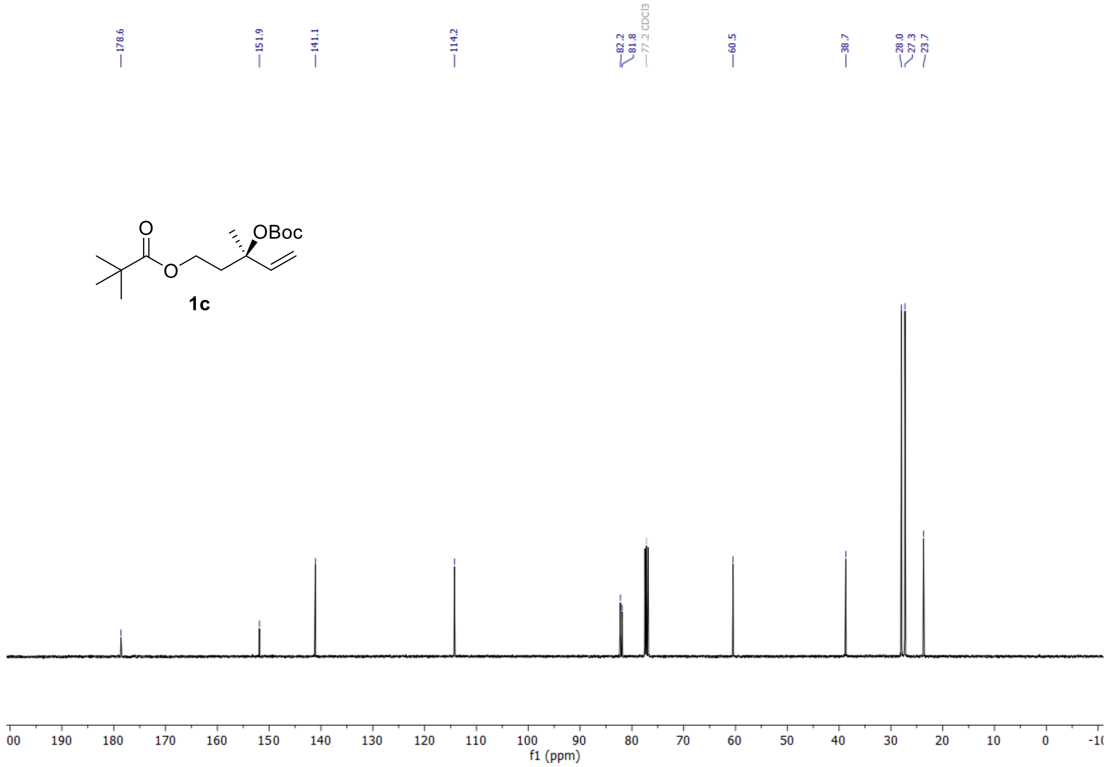

^{1}H NMR (400 MHz, $CDCl_3$) of **1d**

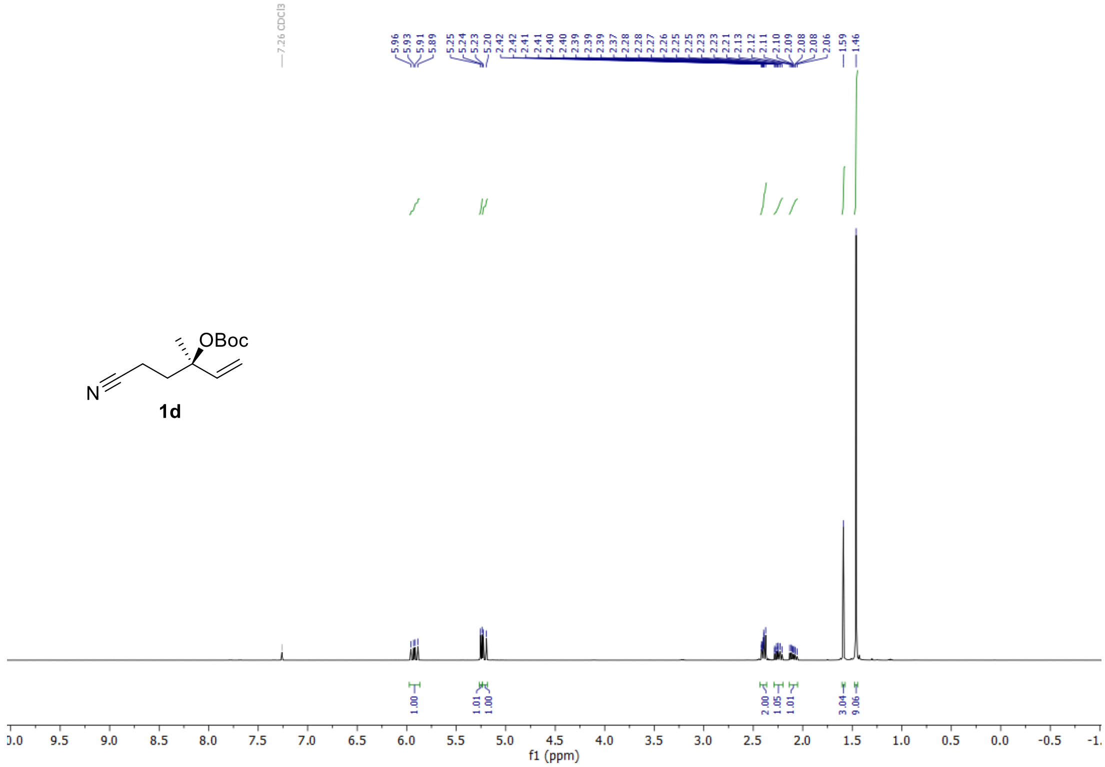

^{13}C NMR (101 MHz, $CDCl_3$) of **1d**

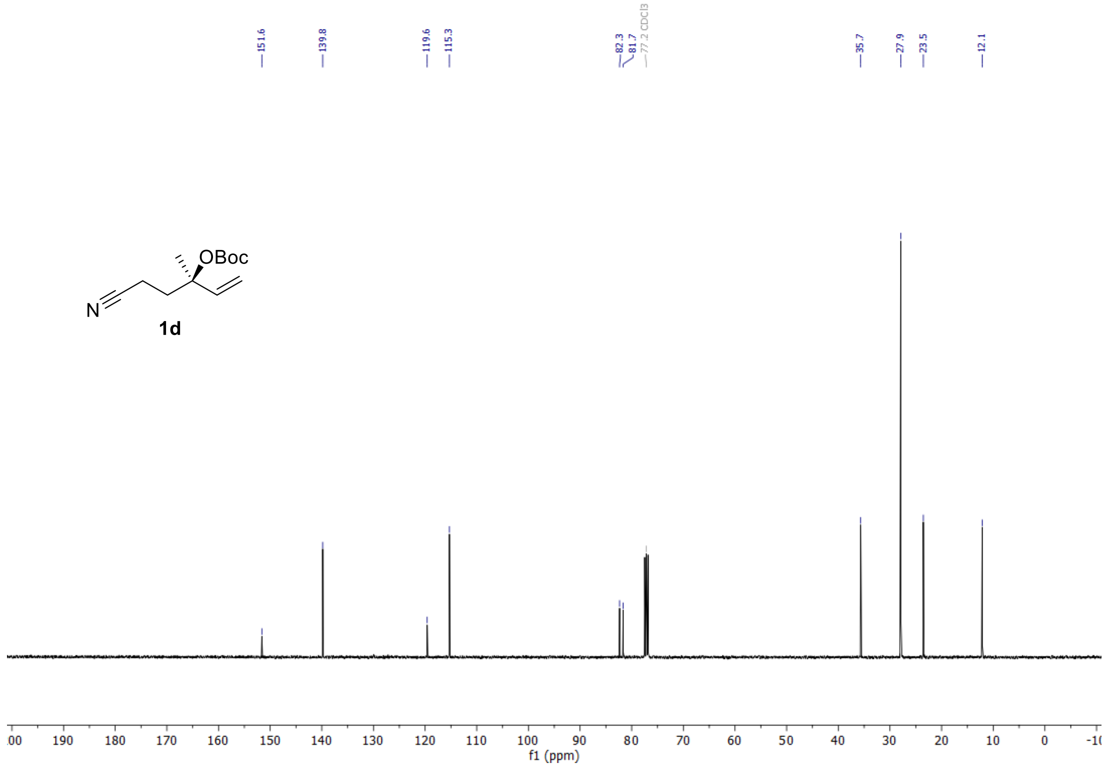

^{1}H NMR (400 MHz, $CDCl_3$) of **1e**

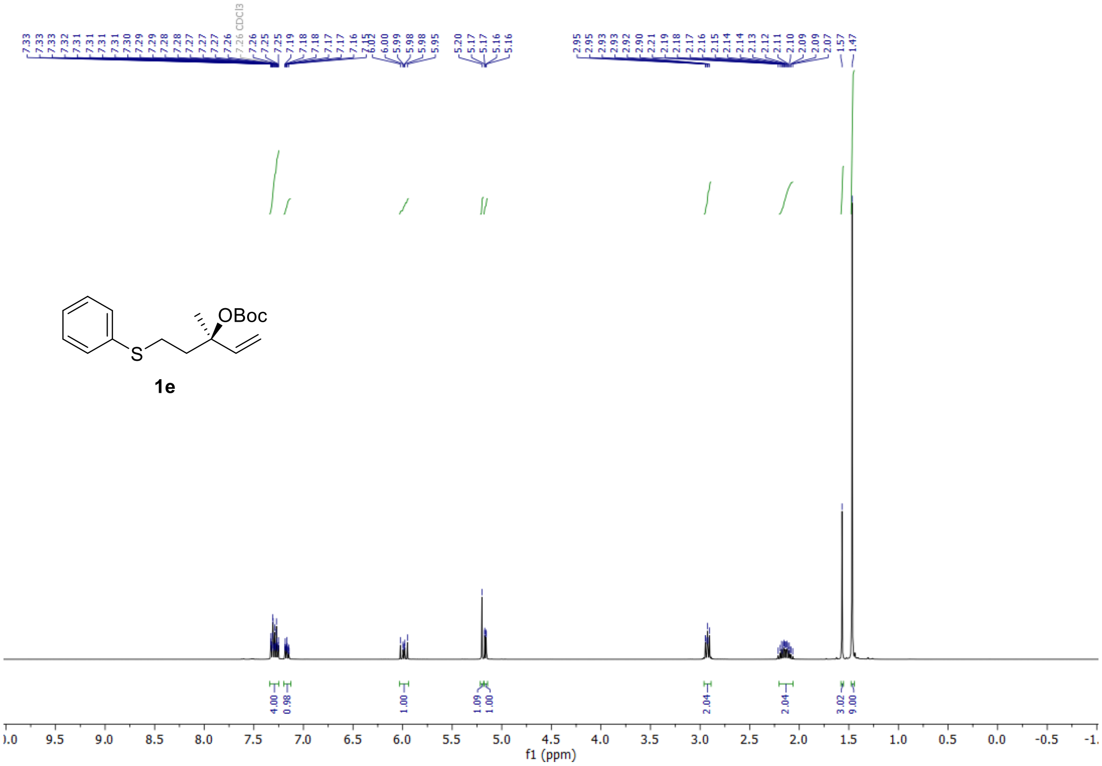

^{13}C NMR (101 MHz, $CDCl_3$) of **1e**

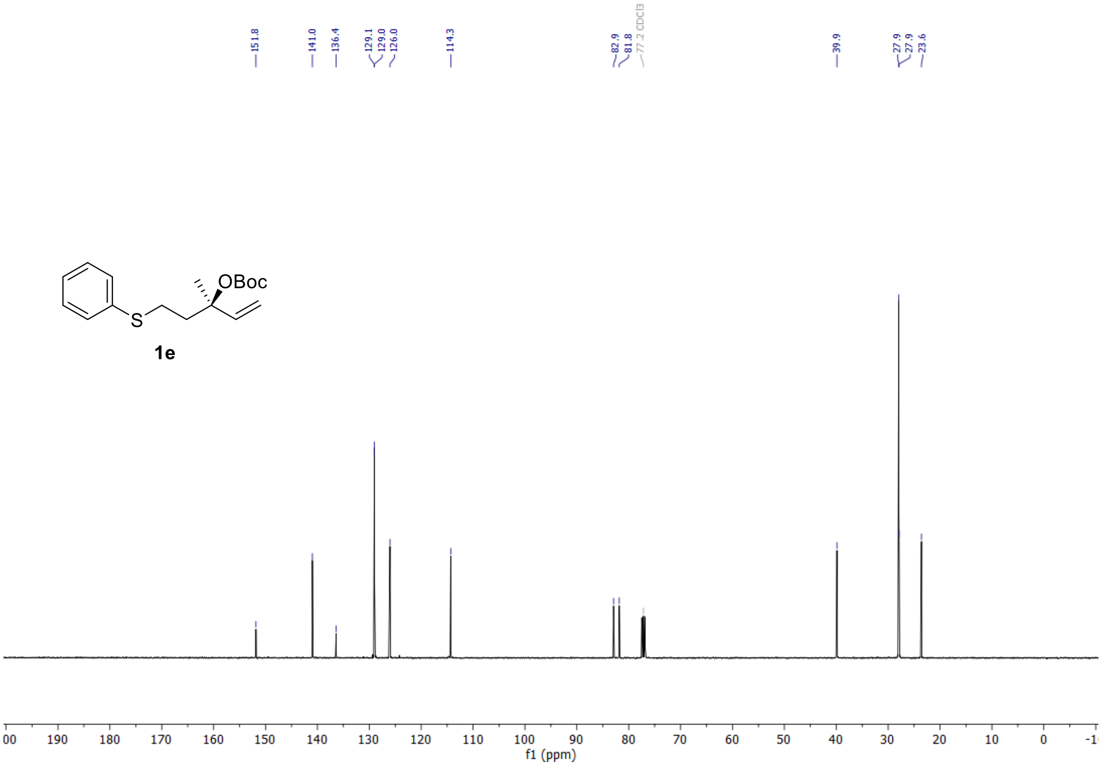

^{1}H NMR (400 MHz, $CDCl_3$) of **1f**

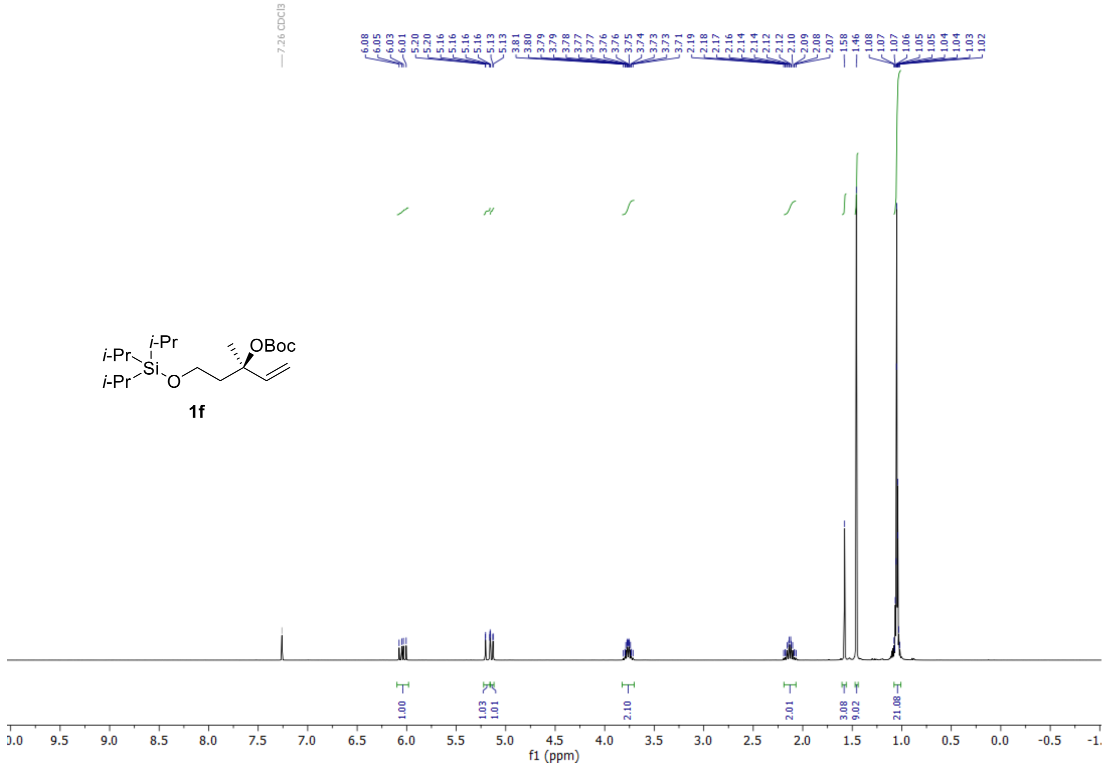

^{13}C NMR (101 MHz, $CDCl_3$) of **1f**

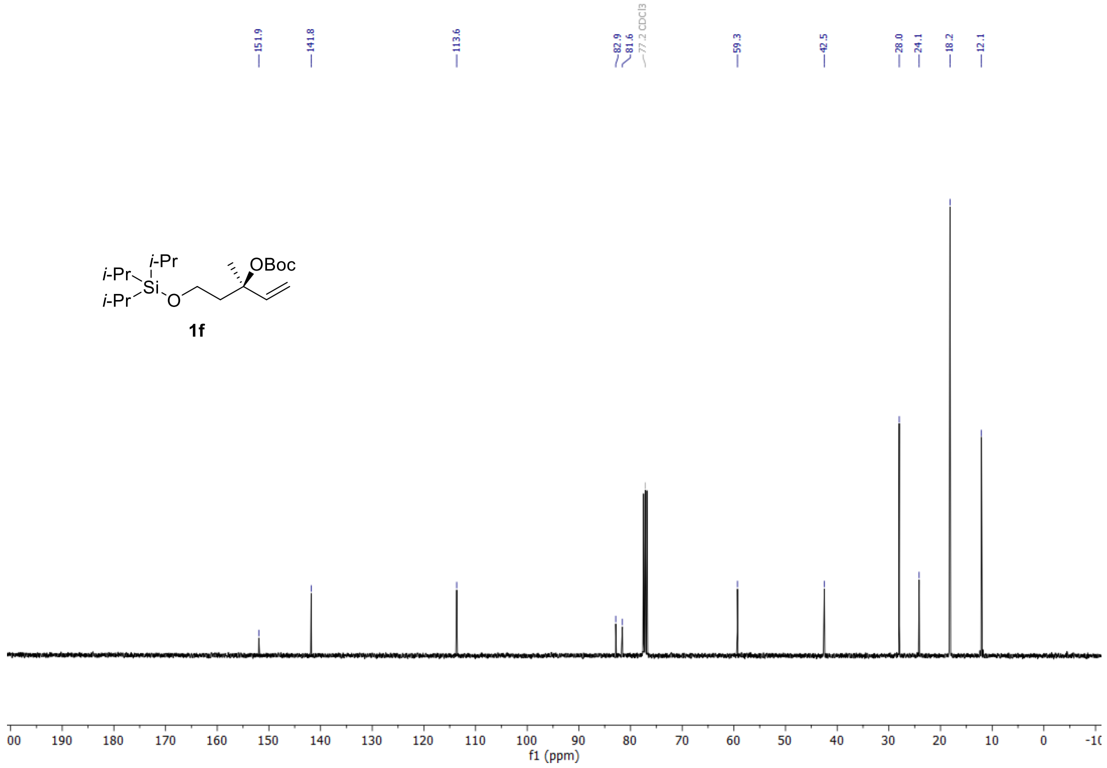

^{1}H NMR (400 MHz, $CDCl_3$) of **1i**

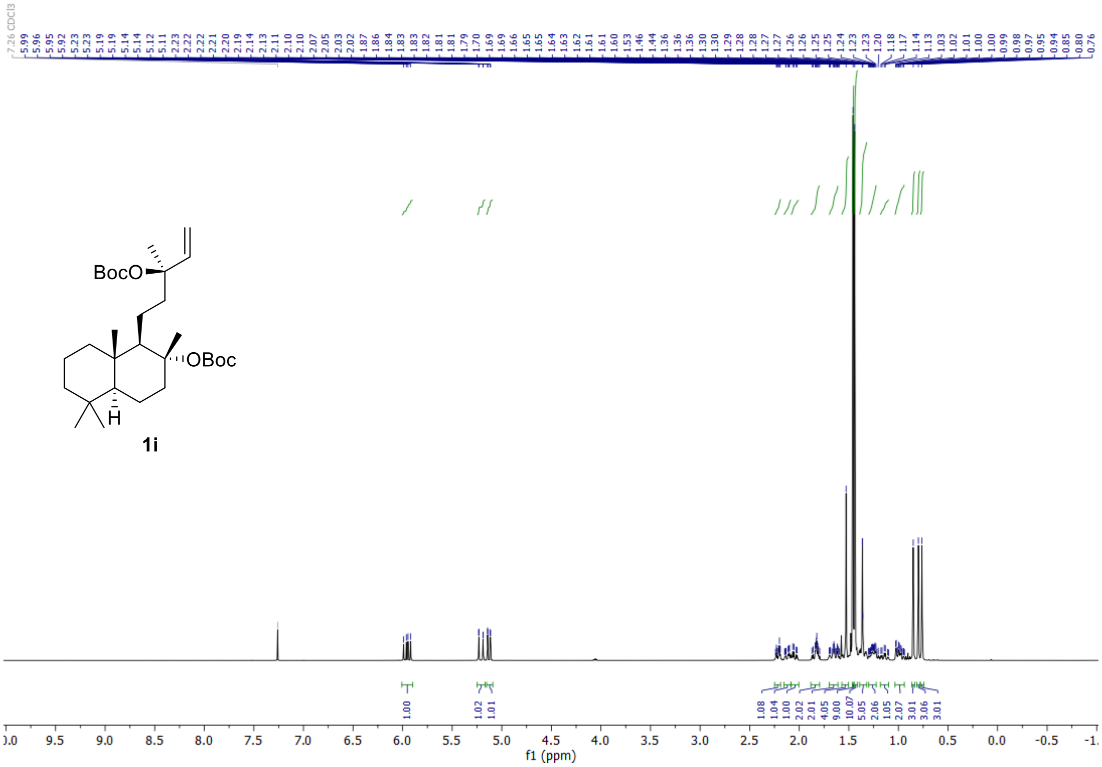

^{13}C NMR (101 MHz, $CDCl_3$) of **1i**

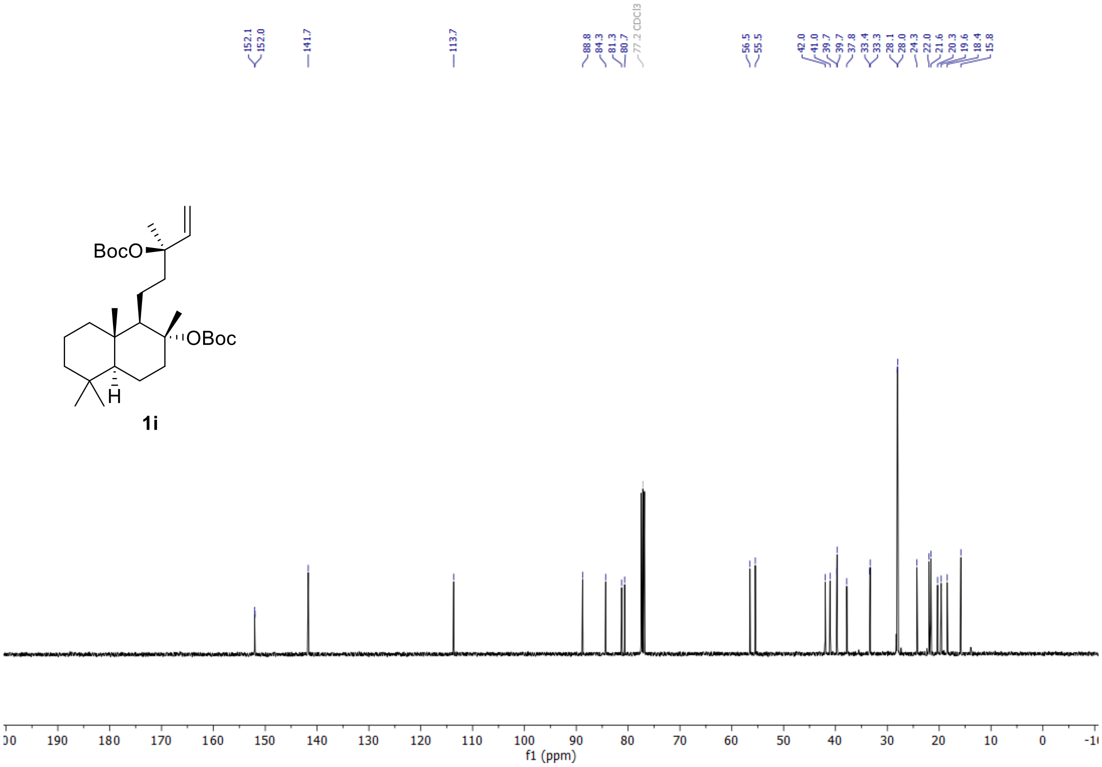

^{1}H NMR (400 MHz, $CDCl_3$) of **1k**

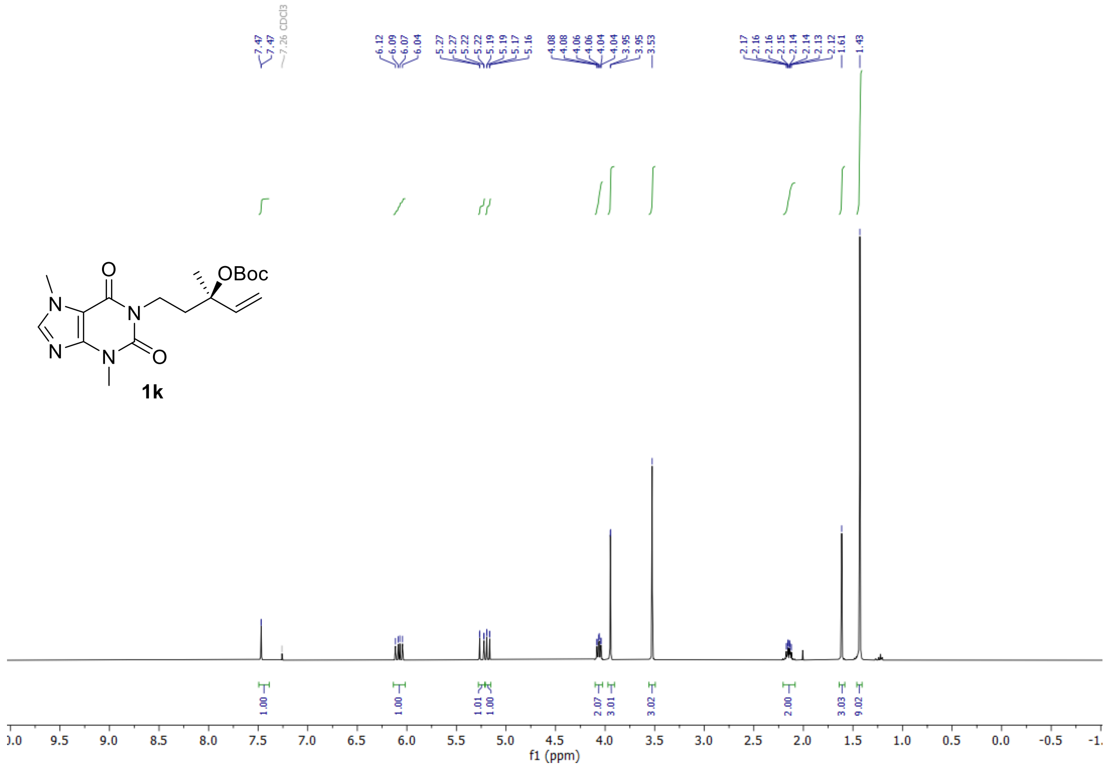

^{13}C NMR (101 MHz, $CDCl_3$) of **1k**

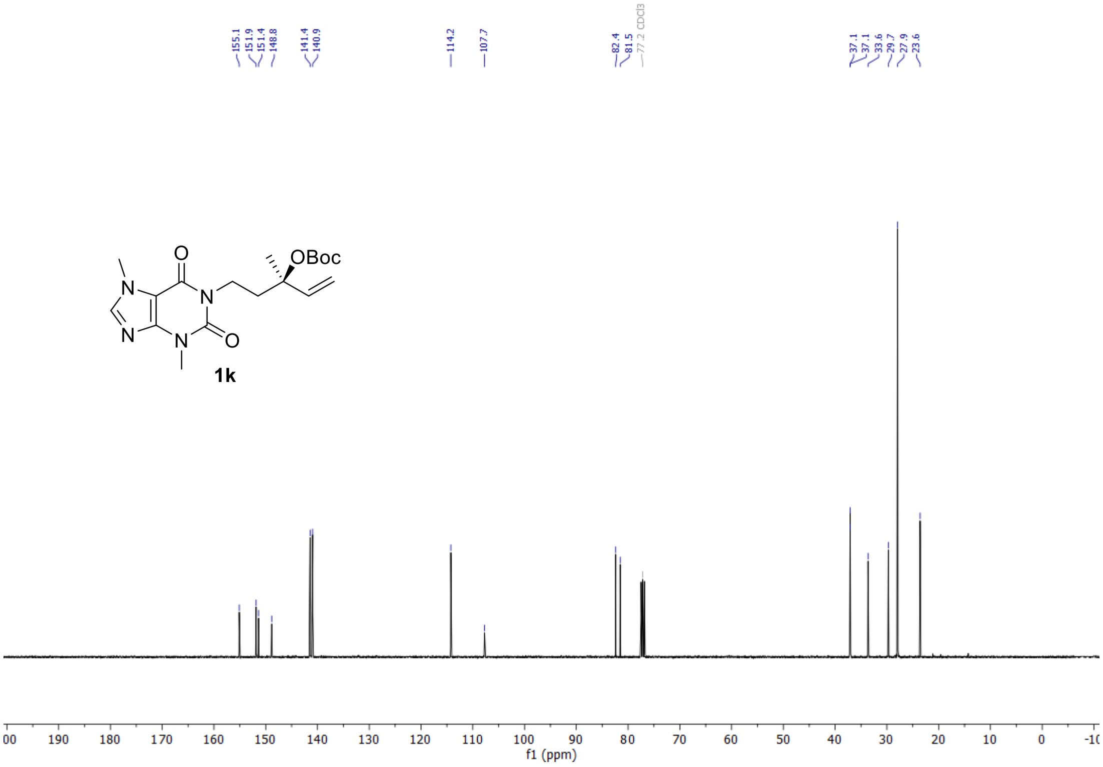

^{1}H NMR (400 MHz, $CDCl_3$) of **1l**

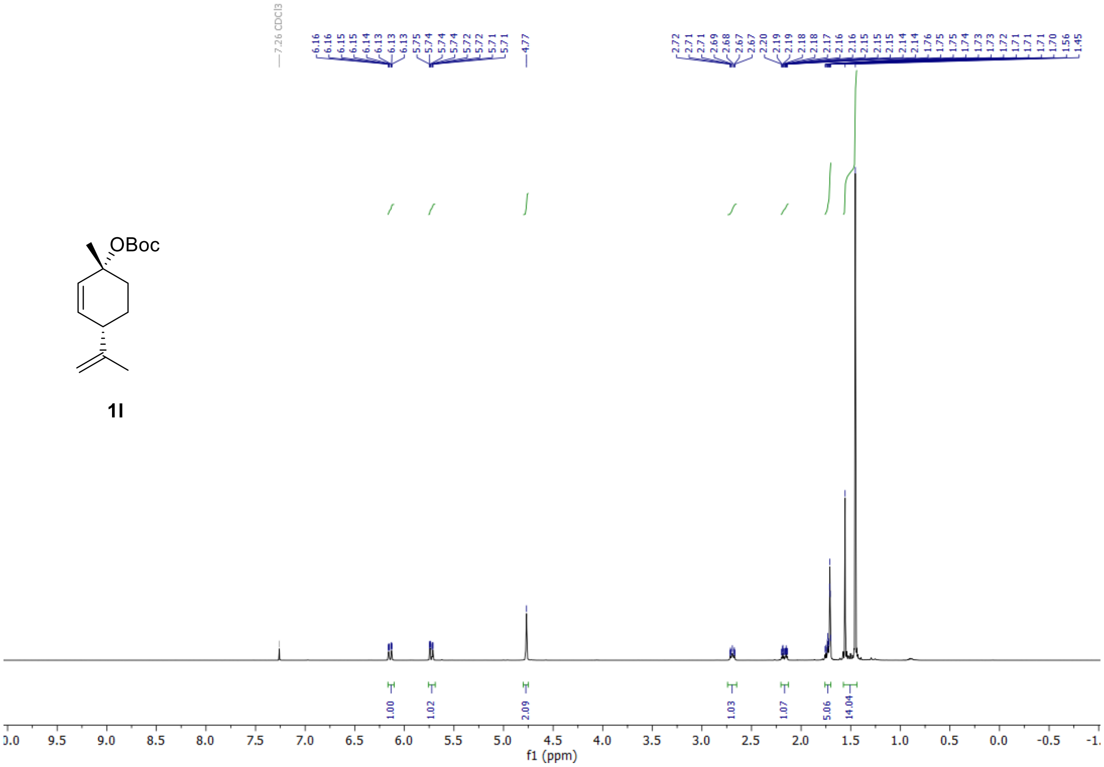

^{13}C NMR (101 MHz, $CDCl_3$) of **1l**

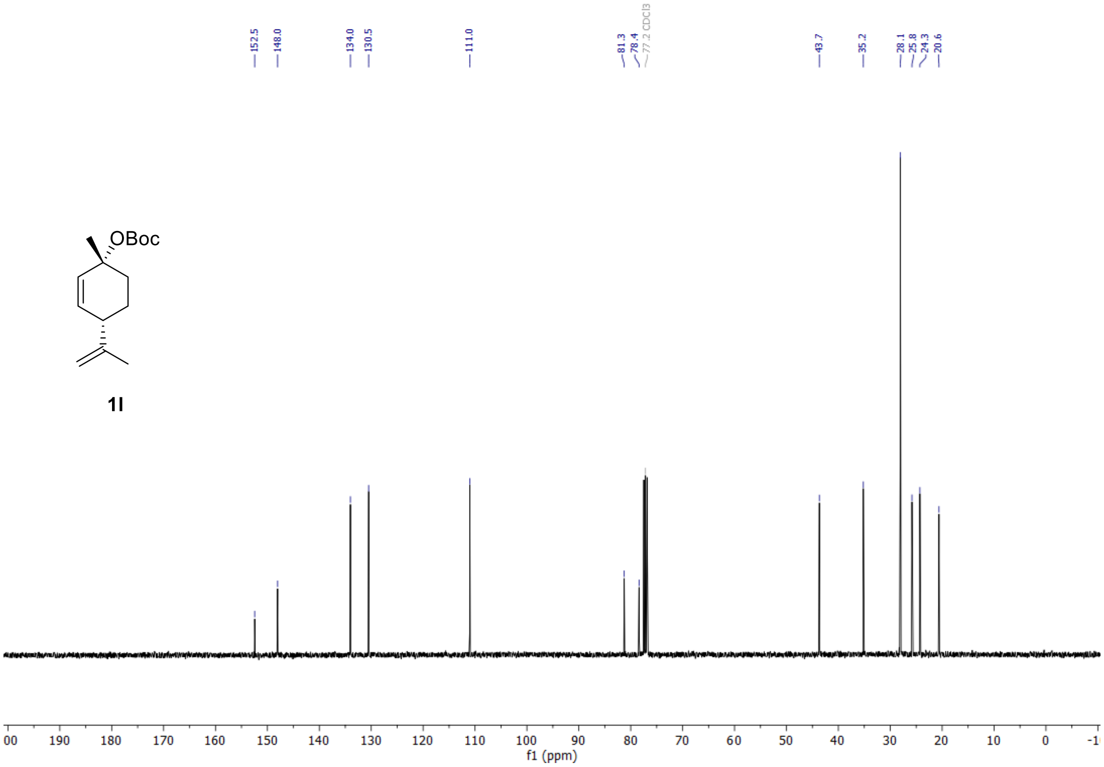

^{1}H NMR (400 MHz, $CDCl_3$) of **1m**

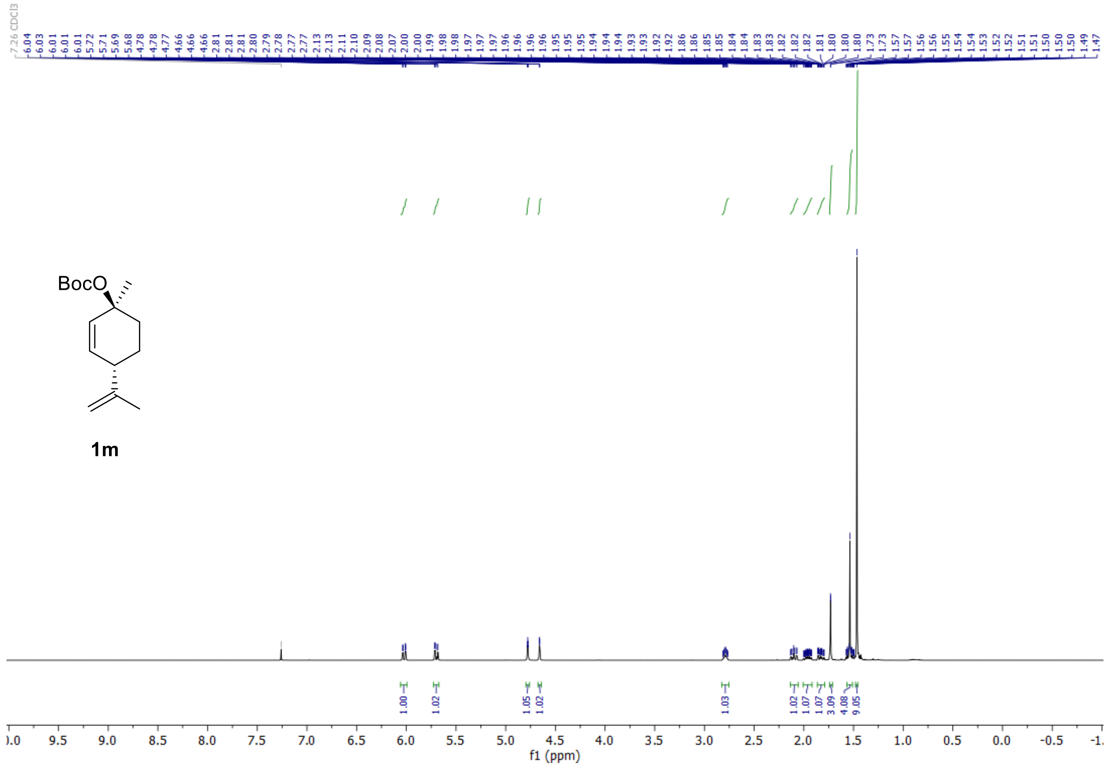

^{13}C NMR (101 MHz, $CDCl_3$) of **1m**

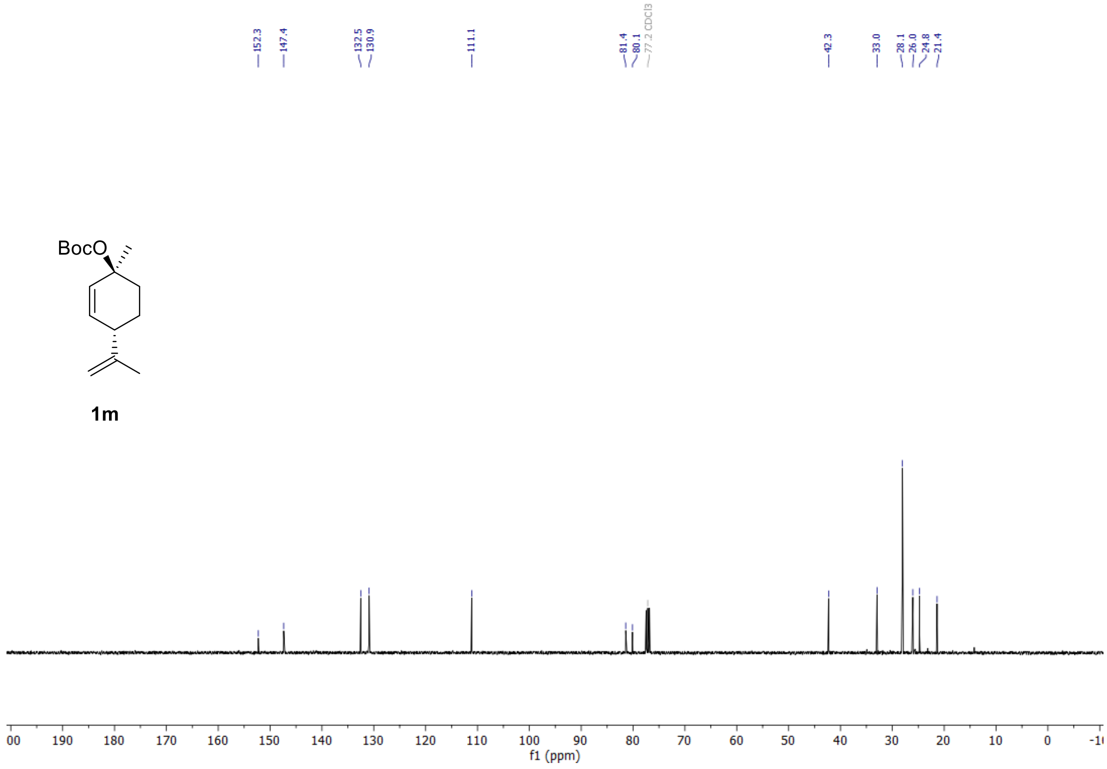

^{1}H NMR (400 MHz, $CDCl_3$) of **L7**

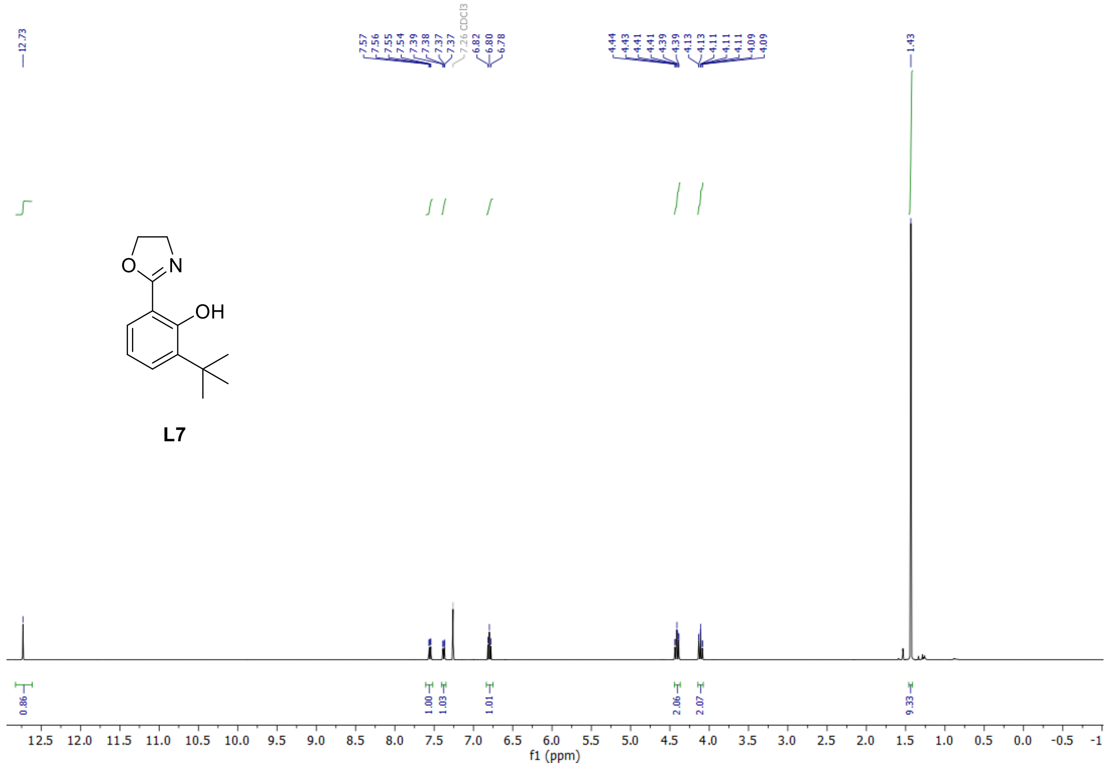

^{13}C NMR (101 MHz, $CDCl_3$) of **L7**

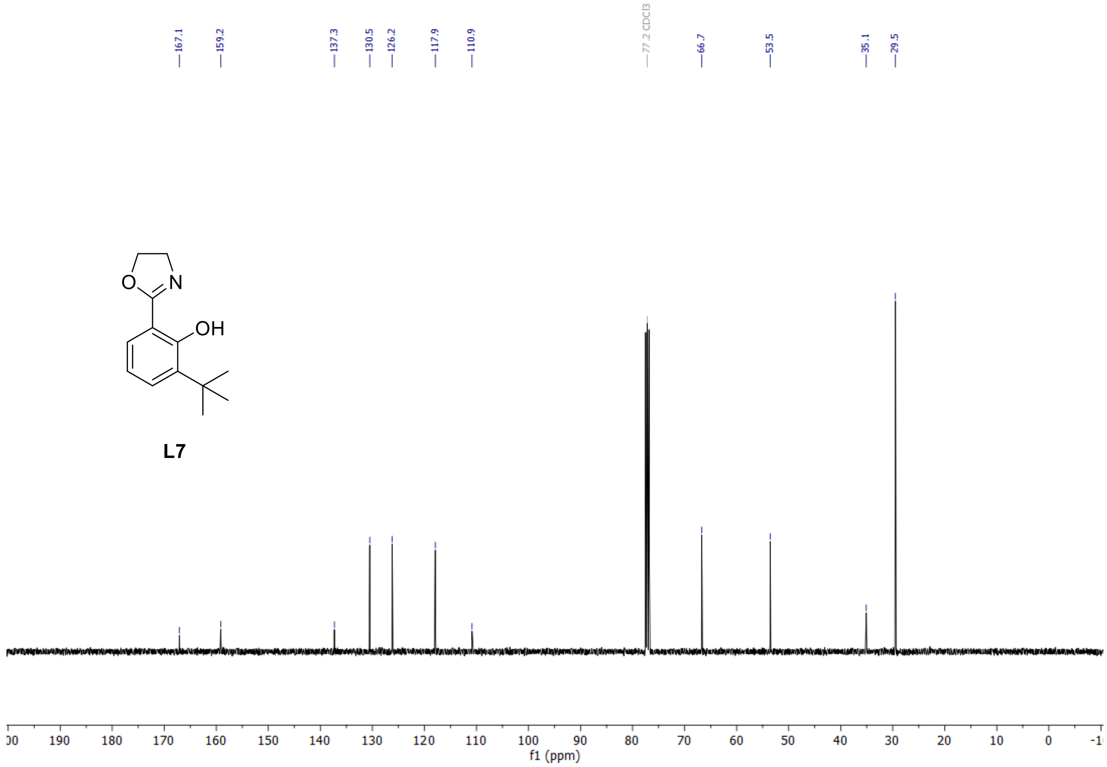

7. SFC Traces

Racemic sample (Daicel Chiralcel OJ-H, 10% MeOH, 2.0 mL/min, 25 °C)

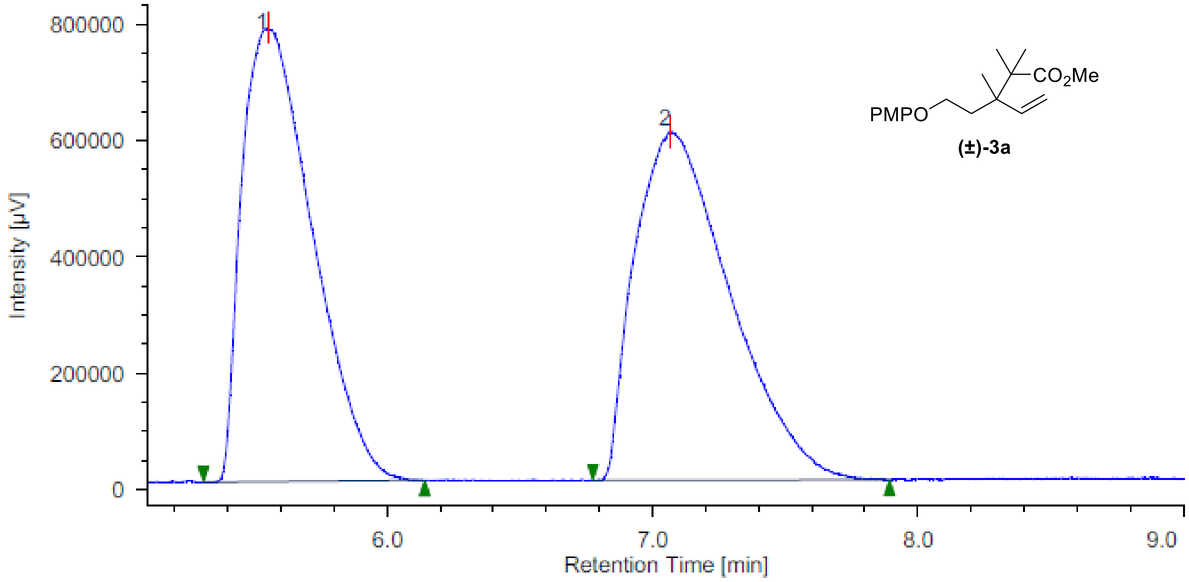

#	Peak Name	CH	tR [min]	Area [µV·sec]	Height [µV]	Area%	Height%	Quantity	NTP	Resolution	Symmetry Factor	Warning
1	Unknown	1	5.550	14370155	779794	49.100	56.559	N/A	1899	2.549	1.728	
2	Unknown	1	7.067	14897197	598929	50.900	43.441	N/A	1708	N/A	1.687	

(+)-3a

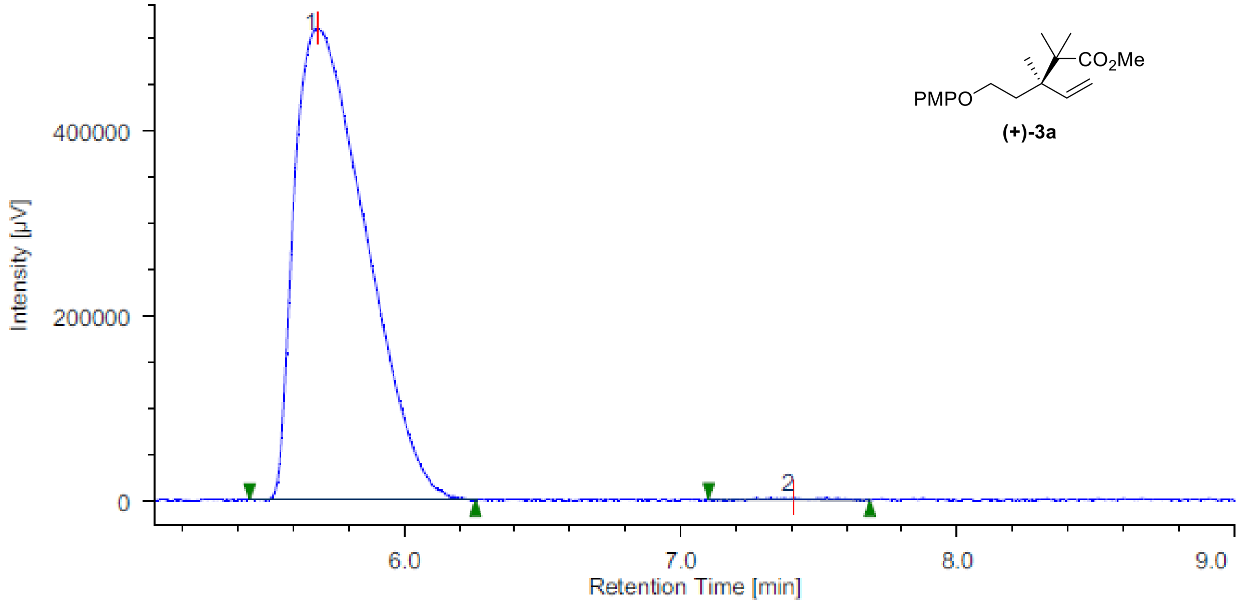

#	Peak Name	CH	tR [min]	Area [µV·sec]	Height [µV]	Area%	Height%	Quantity	NTP	Resolution	Symmetry Factor	Warning
1	Unknown	1	5.683	9095772	508317	99.686	99.485	N/A	2092	3.298	1.937	
2	Unknown	1	7.408	28614	2633	0.314	0.515	N/A	2884	N/A	0.972	

Racemic sample (Daicel Chiralcel OJ-H, 5% MeOH, 2.0 mL/min, 25 °C)

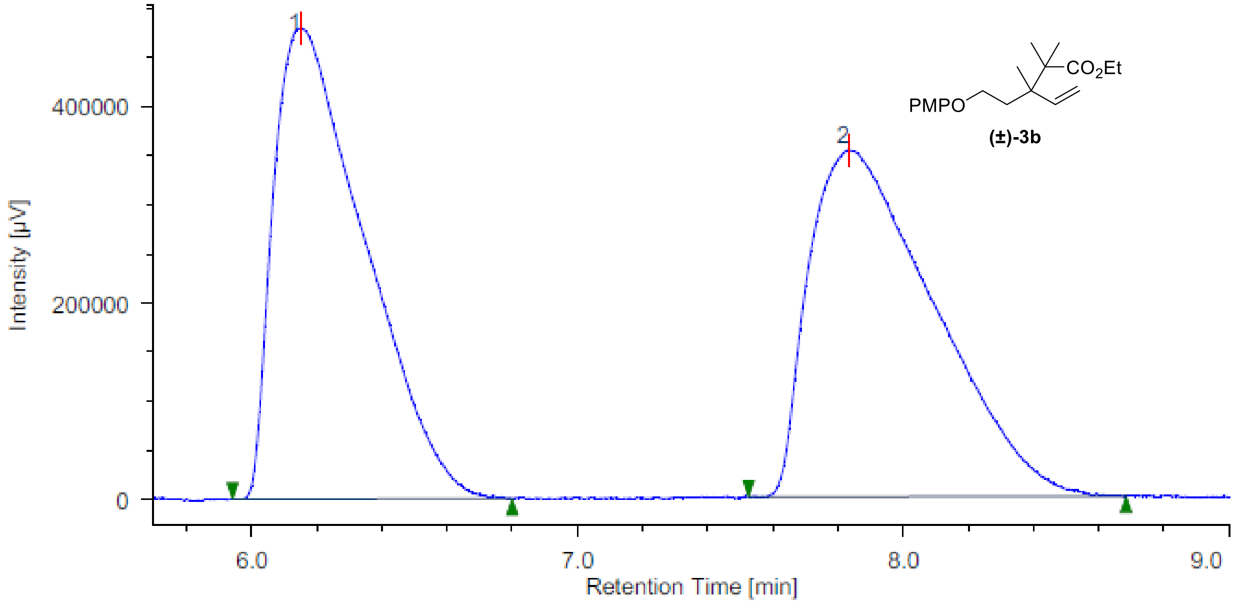

#	Peak Name	CH	tR [min]	Area [µV·sec]	Height [µV]	Area%	Height%	Quantity	NTP	Resolution	Symmetry Factor	Warning
1	Unknown	1	6.150	9289647	478557	49.684	57.598	N/A	2098	2.628	2.086	
2	Unknown	1	7.833	9407841	352303	50.316	42.402	N/A	1758	N/A	1.940	

(+)-3b

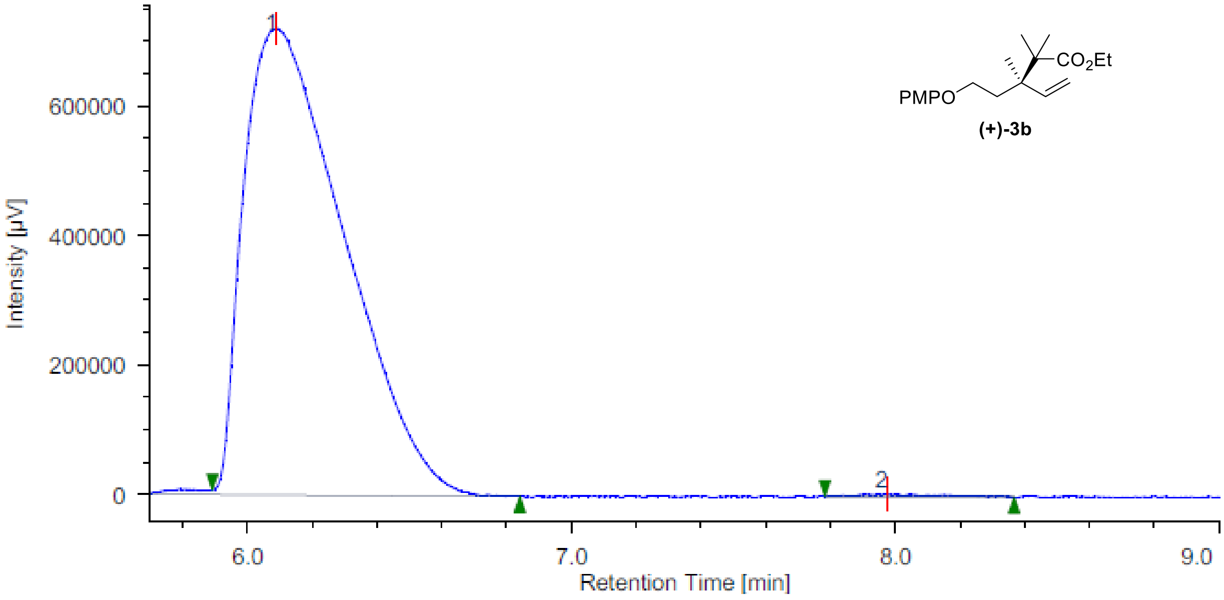

#	Peak Name	CH	tR [min]	Area [µV·sec]	Height [µV]	Area%	Height%	Quantity	NTP	Resolution	Symmetry Factor	Warning
1	Unknown	1	6.092	15503370	719442	99.592	99.483	N/A	1661	3.310	1.910	
2	Unknown	1	7.975	63445	3737	0.408	0.517	N/A	3449	N/A	1.494	

Racemic sample (Daicel Chiralcel OJ-H, 5% MeOH, 2.0 mL/min, 25 °C)

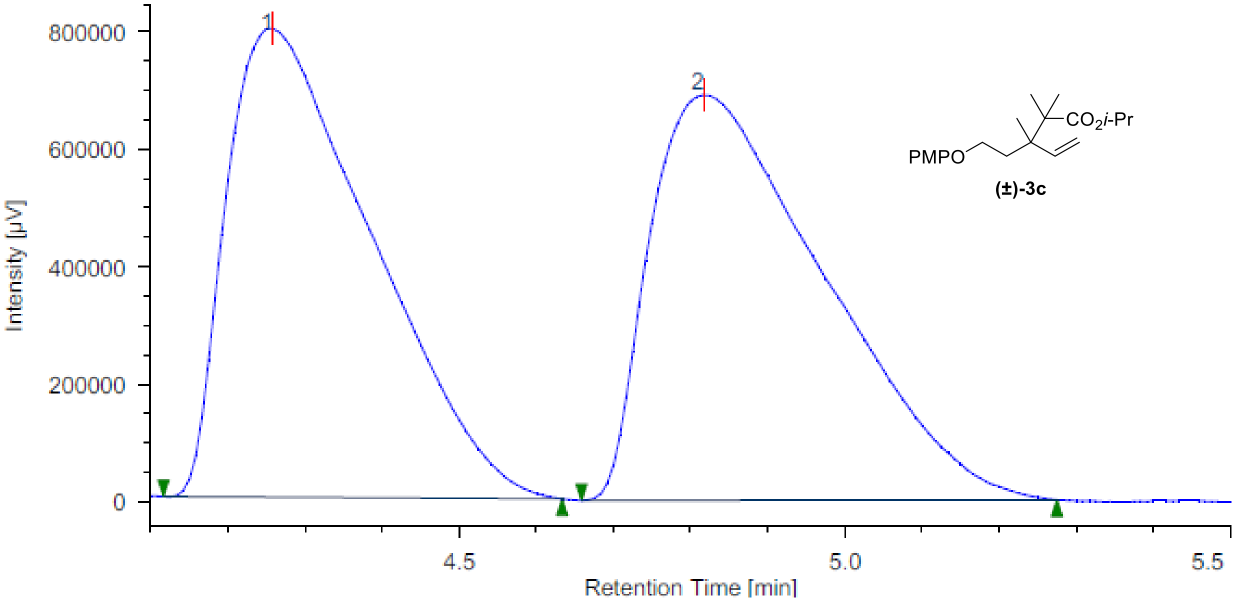

#	Peak Name	CH	tR [min]	Area [µV·sec]	Height [µV]	Area%	Height%	Quantity	NTP	Resolution	Symmetry Factor	Warning
1	Unknown	1	4.258	10471009	796914	49.113	53.644	N/A	2172	1.396	1.906	
2	Unknown	1	4.817	10849112	688641	50.887	46.356	N/A	1949	N/A	1.967	

(+)-3c

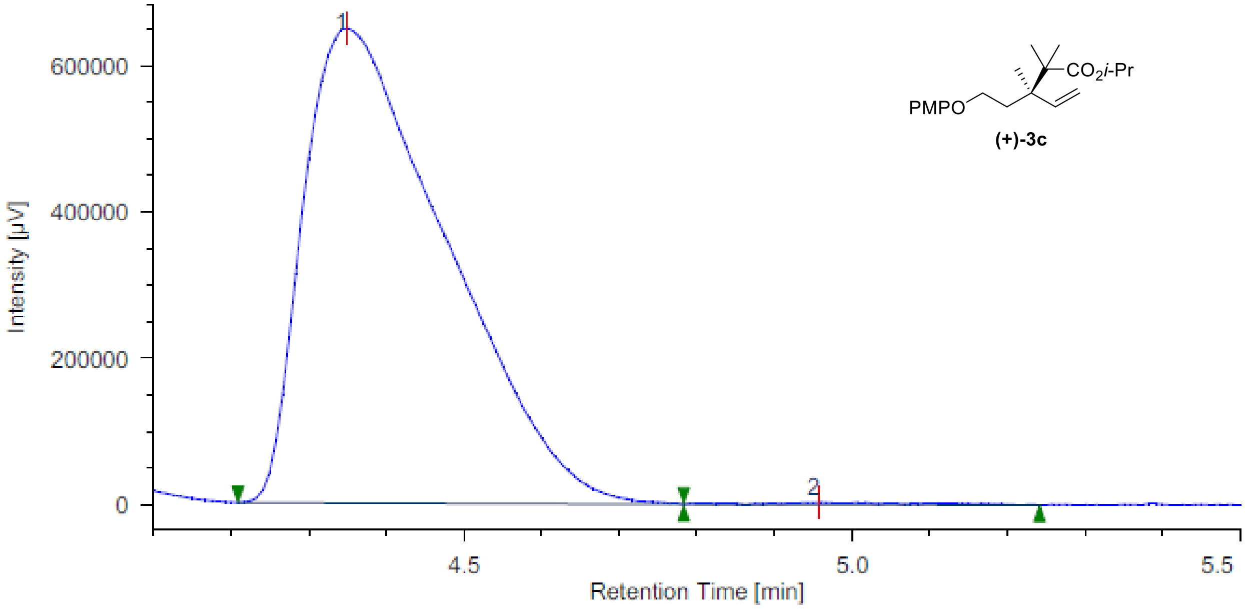

#	Peak Name	CH	tR [min]	Area [µV·sec]	Height [µV]	Area%	Height%	Quantity	NTP	Resolution	Symmetry Factor	Warning
1	Unknown	1	4.350	8310120	648189	99.555	99.513	N/A	2417	1.685	1.945	
2	Unknown	1	4.958	37161	3172	0.445	0.487	N/A	2876	N/A	1.308	

Racemic sample (Daicel Chiralcel OJ-H, 1% MeOH, 2.0 mL/min, 25 °C)

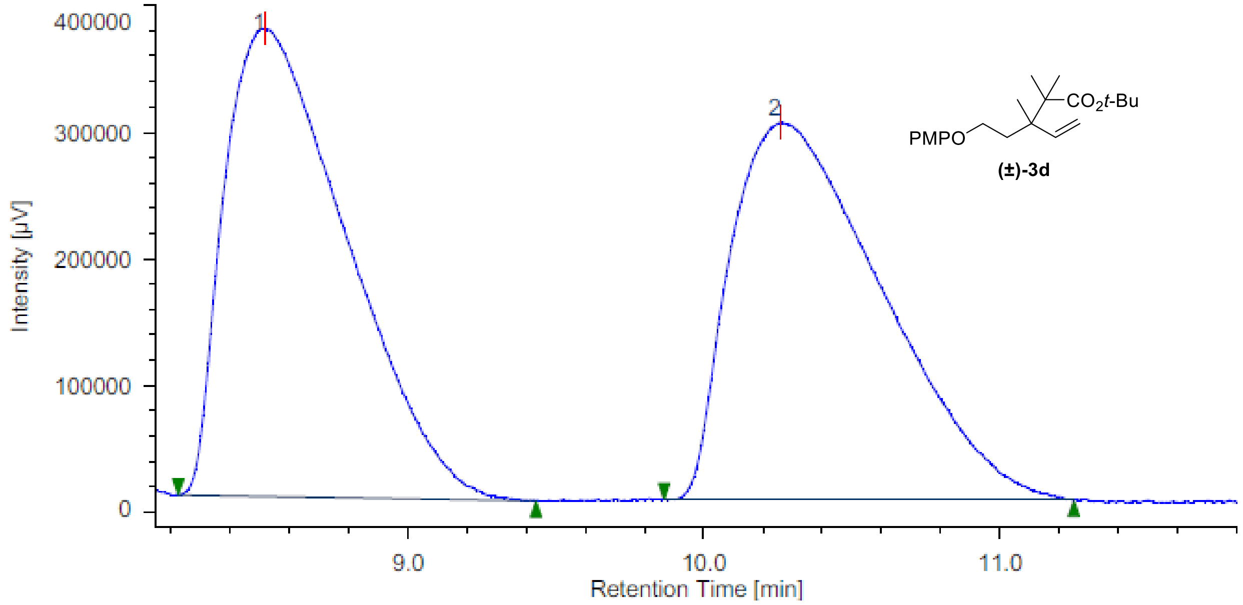

#	Peak Name	CH	tR [min]	Area [µV·sec]	Height [µV]	Area%	Height%	Quantity	NTP	Resolution	Symmetry Factor	Warning
1	Unknown	1	8.517	10639214	369453	50.070	55.350	N/A	1827	1.954	1.861	
2	Unknown	1	10.258	10609259	298027	49.930	44.650	N/A	1716	N/A	1.821	

(+)-3d

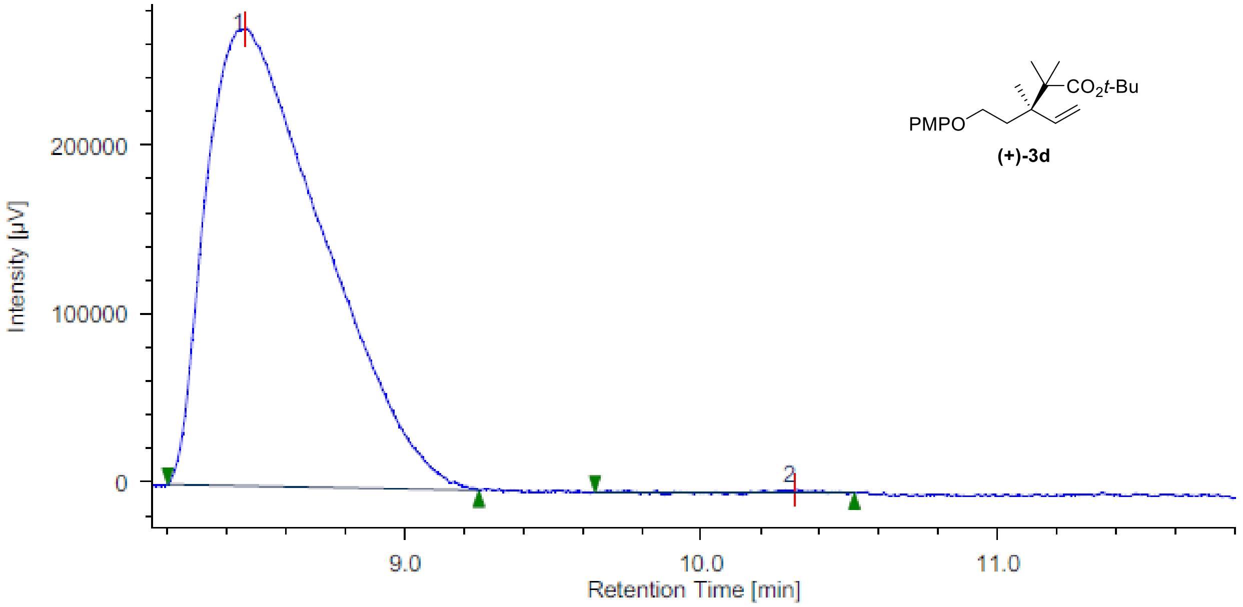

#	Peak Name	CH	tR [min]	Area [µV·sec]	Height [µV]	Area%	Height%	Quantity	NTP	Resolution	Symmetry Factor	Warning
1	Unknown	1	8.458	7472033	271088	99.781	99.372	N/A	1962	1.977	1.885	
2	Unknown	1	10.317	16403	1713	0.219	0.628	N/A	1356	N/A	0.622	

Racemic sample (Daicel Chiralcel OJ-H, 5% MeOH, 2.0 mL/min, 25 °C)

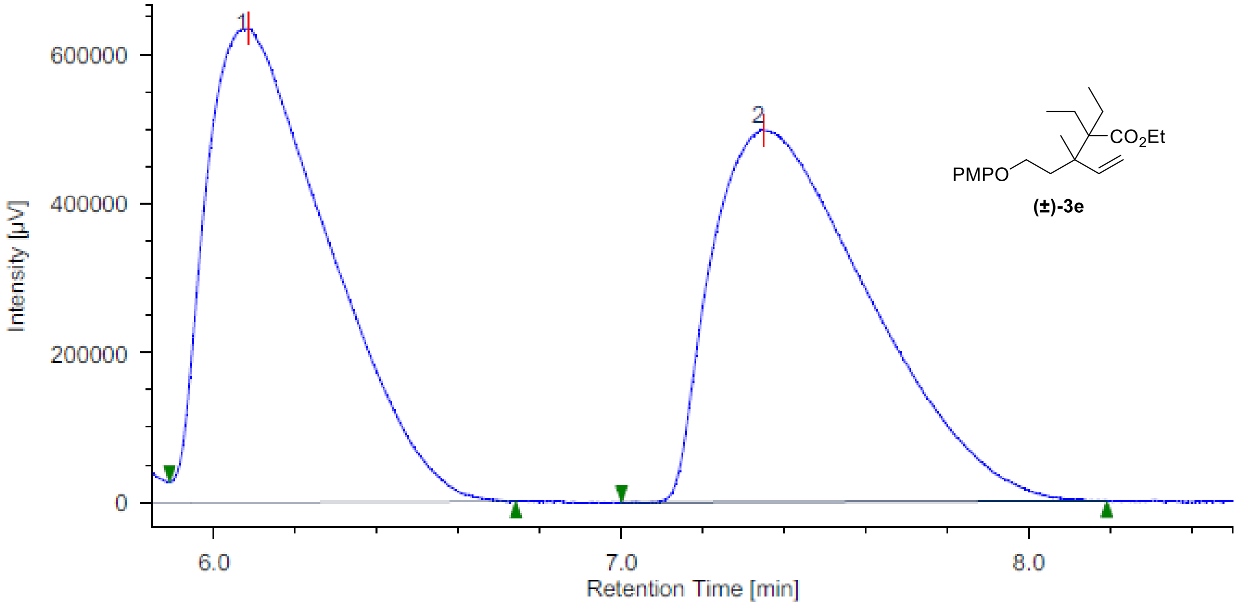

#	Peak Name	CH	tR [min]	Area [µV·sec]	Height [µV]	Area%	Height%	Quantity	NTP	Resolution	Symmetry Factor	Warning
1	Unknown	1	6.083	13248741	634273	49.782	56.047	N/A	1793	1.930	1.807	
2	Unknown	1	7.350	13364796	497403	50.218	43.953	N/A	1572	N/A	1.902	

(+)-3e

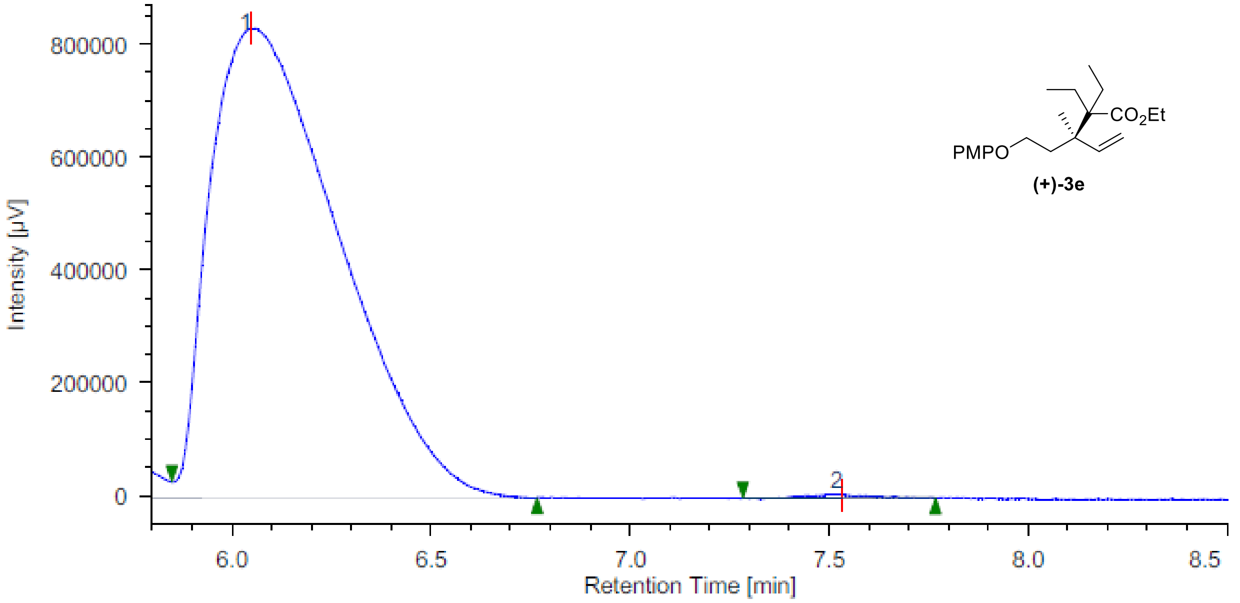

#	Peak Name	CH	tR [min]	Area [µV·sec]	Height [µV]	Area%	Height%	Quantity	NTP	Resolution	Symmetry Factor	Warning
1	Unknown	1	6.050	18669087	830828	99.676	99.416	N/A	1514	3.093	1.889	
2	Unknown	1	7.533	60762	4883	0.324	0.584	N/A	7866	N/A	0.861	

Racemic sample (Daicel Chiralpak IB, 5% MeOH, 2.0 mL/min, 25 °C)

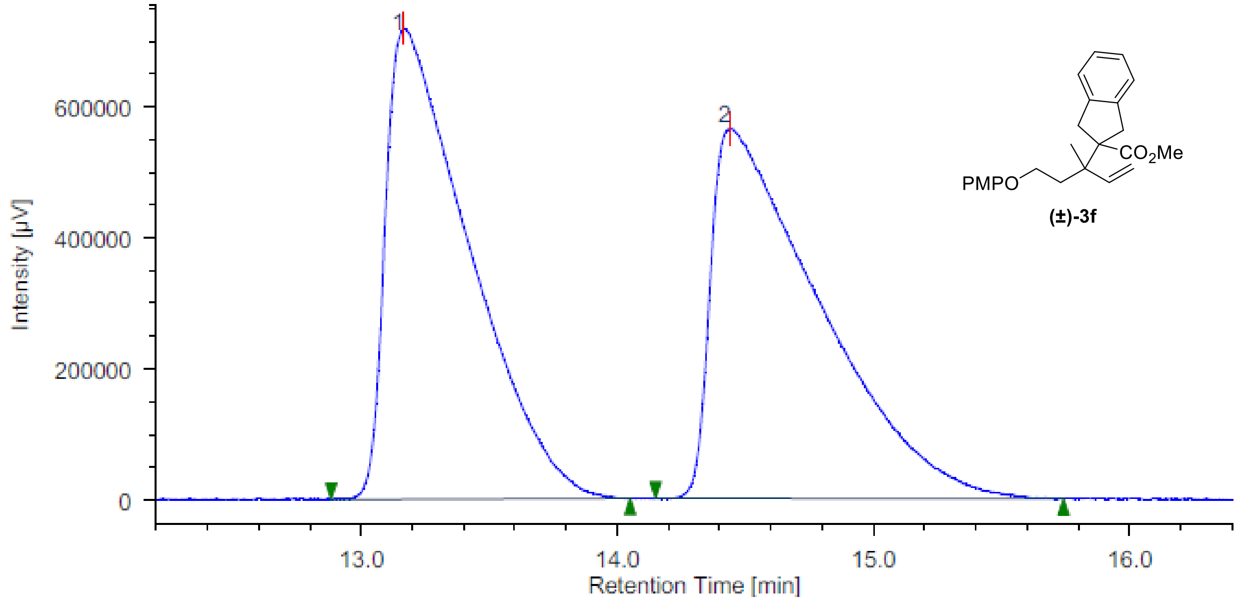

#	Peak Name	CH	tR [min]	Area [µV·sec]	Height [µV]	Area%	Height%	Quantity	NTP	Resolution	Symmetry Factor	Warning
1	Unknown	1	13.167	16293899	717552	49.391	56.010	N/A	7665	1.857	2.711	
2	Unknown	1	14.442	16695639	563557	50.609	43.990	N/A	5552	N/A	3.517	

(–)-3f

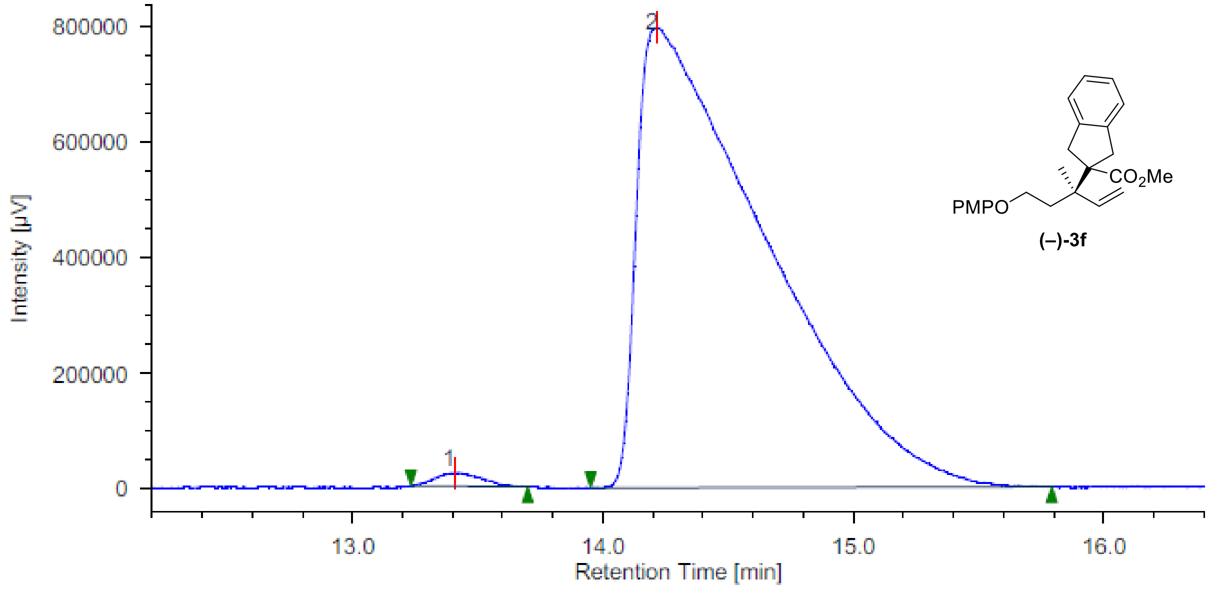

#	Peak Name	CH	tR [min]	Area [µV·sec]	Height [µV]	Area%	Height%	Quantity	NTP	Resolution	Symmetry Factor	Warning
2	Unknown	1	13.408	299781	22987	1.036	2.808	N/A	22254	1.233	1.275	
1	Unknown	1	14.217	28633362	795705	98.964	97.192	N/A	3546	N/A	4.196	

Racemic sample (Daicel Chiralcel OJ-H, 5% MeOH, 2.0 mL/min, 25 °C)

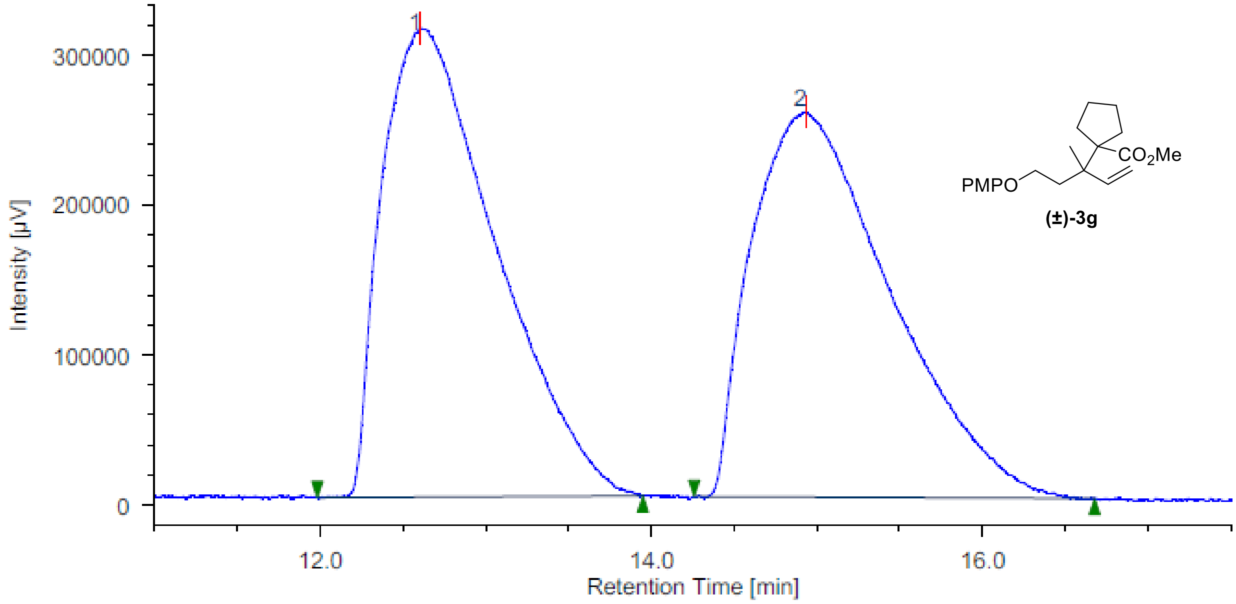

#	Peak Name	CH	tR [min]	Area [µV·sec]	Height [µV]	Area%	Height%	Quantity	NTP	Resolution	Symmetry Factor	Warning
1	Unknown	1	12.608	14886125	312155	49.935	54.864	N/A	1473	1.603	1.890	
2	Unknown	1	14.933	14925132	256807	50.065	45.136	N/A	1404	N/A	1.685	

(+)-3g

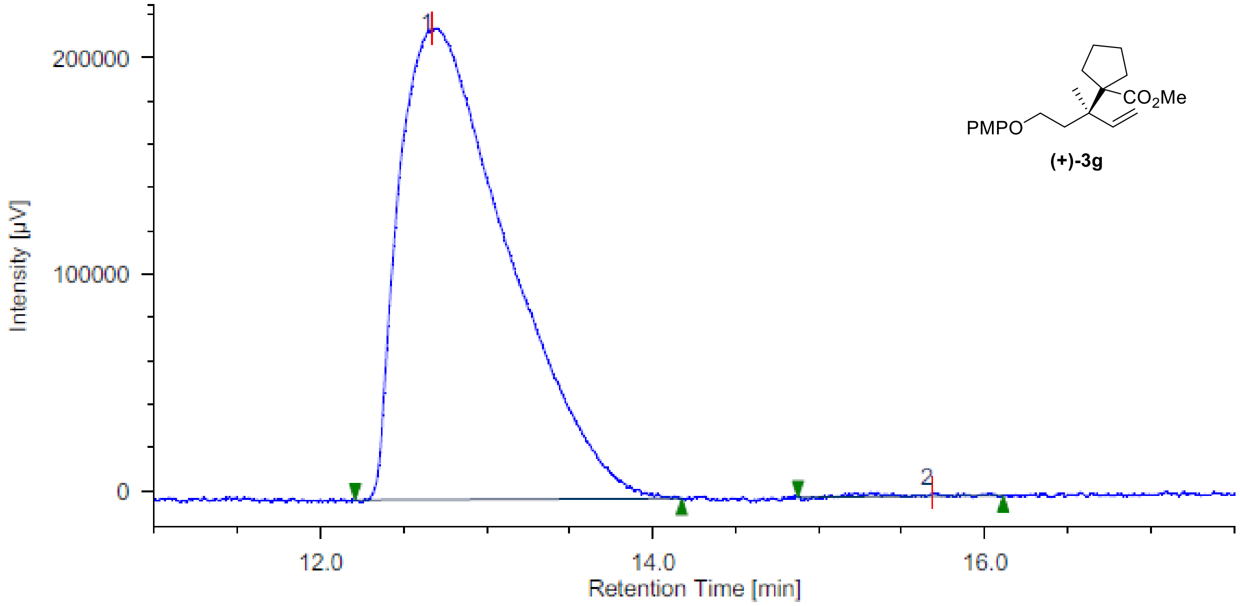

#	Peak Name	CH	tR [min]	Area [µV·sec]	Height [µV]	Area%	Height%	Quantity	NTP	Resolution	Symmetry Factor	Warning
1	Unknown	1	12.675	9887375	217772	99.608	99.208	N/A	1688	2.085	2.125	
2	Unknown	1	15.683	38920	1739	0.392	0.792	N/A	1430	N/A	0.765	

Racemic sample (Daicel Chiralcel OJ-H, 2% MeOH, 2 mL/min, 25 °C)

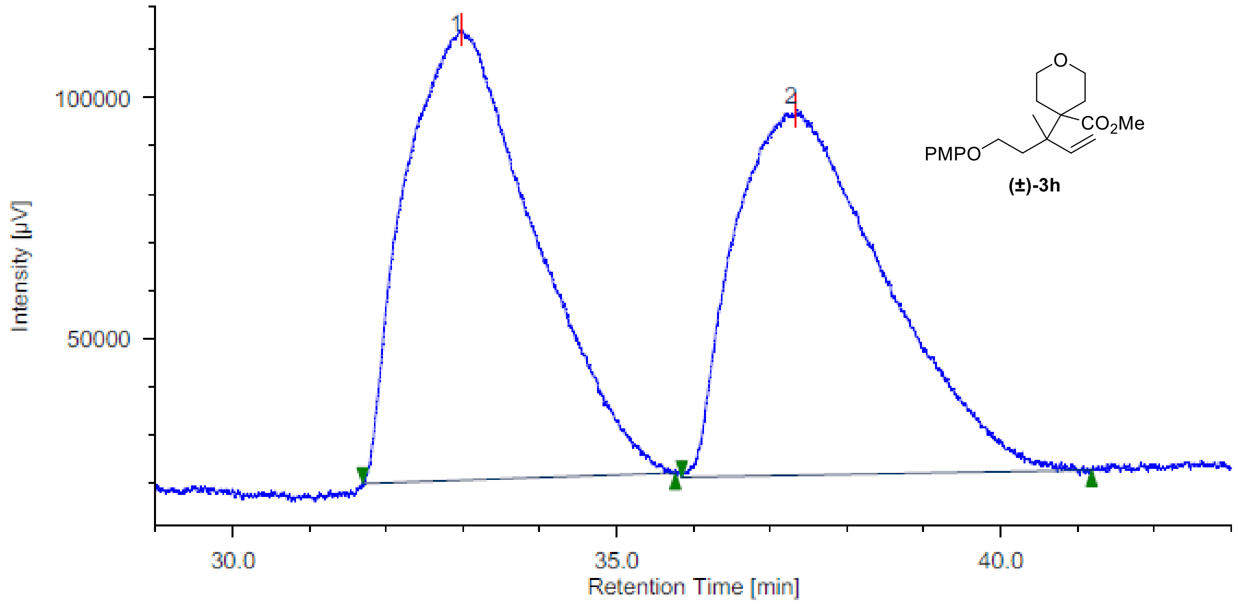

#	Peak Name	CH	tR [min]	Area [µV·sec]	Height [µV]	Area%	Height%	Quantity	NTP	Resolution	Symmetry Factor	Warning
1	Unknown	1	32.992	10931728	93515	51.546	55.225	N/A	1571	1.235	1.469	
2	Unknown	1	37.342	10276014	75819	48.454	44.775	N/A	1601	N/A	1.635	

(+)-3h

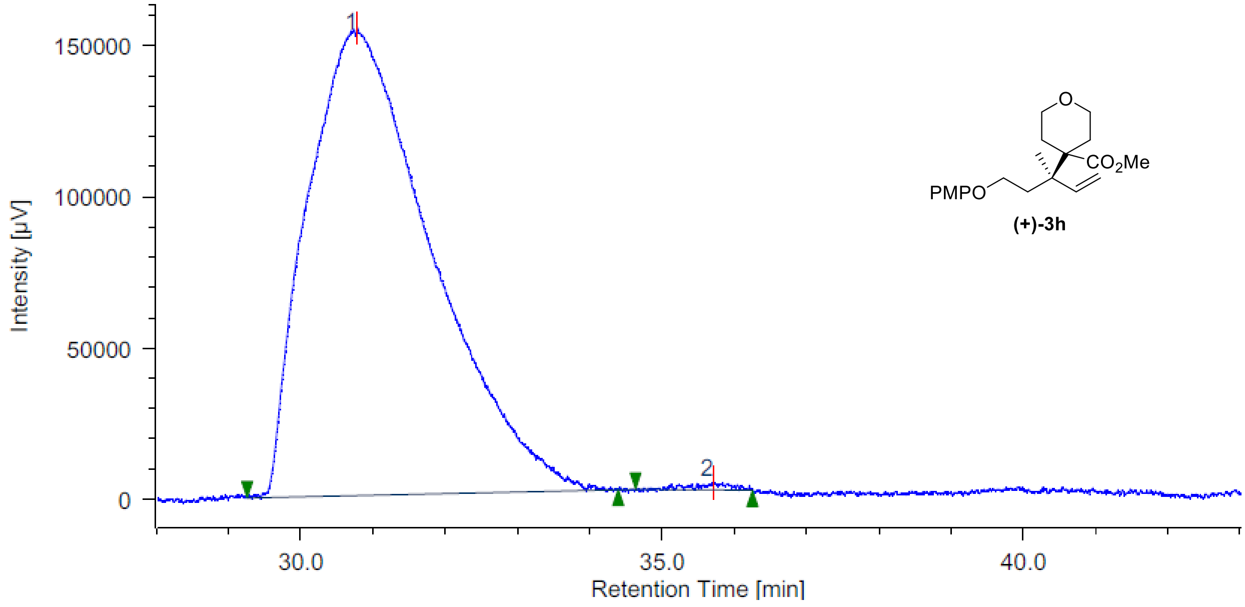

#	Peak Name	CH	tR [min]	Area [µV·sec]	Height [µV]	Area%	Height%	Quantity	NTP	Resolution	Symmetry Factor	Warning
1	Unknown	1	30.783	18384691	154550	99.540	98.497	N/A	1445	1.930	1.652	
2	Unknown	1	35.708	84900	2358	0.460	1.503	N/A	5790	N/A	0.780	

Racemic sample (Daicel Chiralcel OJ-H, 5% MeOH, 2.0 mL/min, 25 °C)

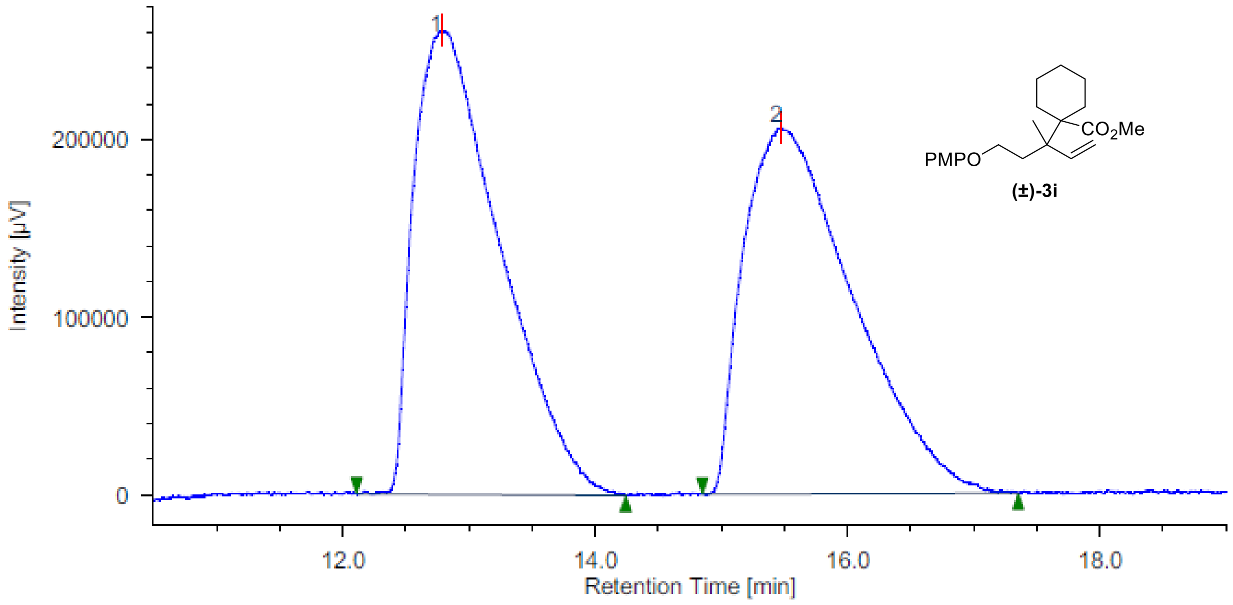

#	Peak Name	CH	tR [min]	Area [µV·sec]	Height [µV]	Area%	Height%	Quantity	NTP	Resolution	Symmetry Factor	Warning
1	Unknown	1	12.783	12362392	260787	49.984	55.908	N/A	1572	1.835	2.011	
2	Unknown	1	15.475	12370064	205672	50.016	44.092	N/A	1403	N/A	1.871	

(+)-3i

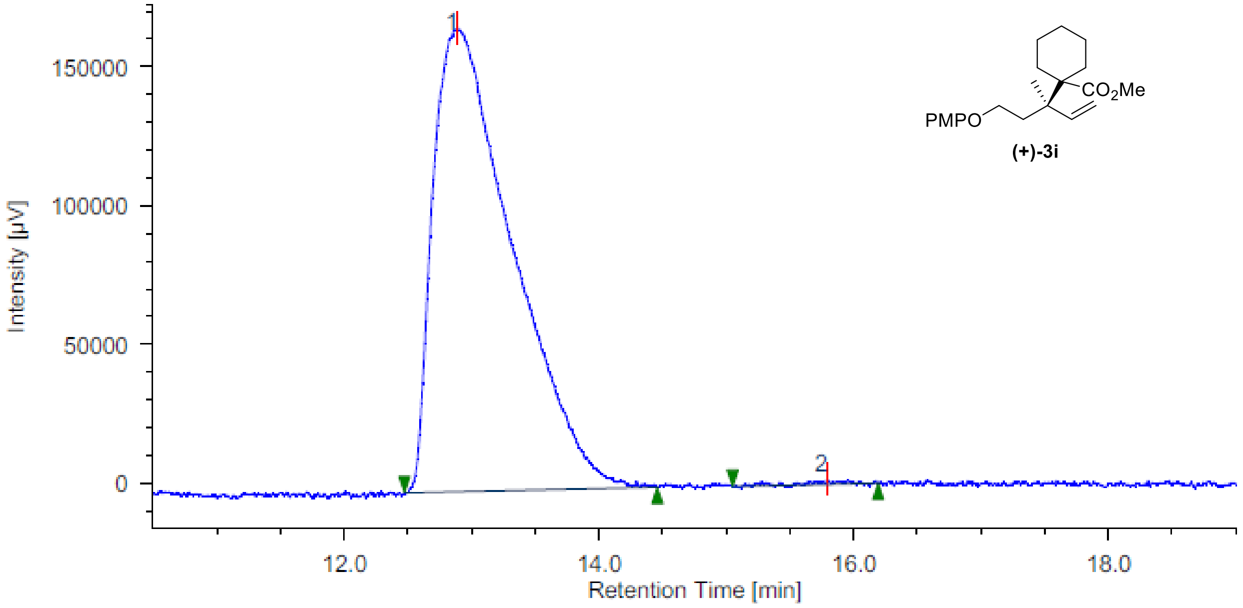

#	Peak Name	CH	tR [min]	Area [µV·sec]	Height [µV]	Area%	Height%	Quantity	NTP	Resolution	Symmetry Factor	Warning
1	Unknown	1	12.892	7212680	166927	99.564	98.755	N/A	1961	2.483	2.121	
2	Unknown	1	15.792	31602	2104	0.436	1.245	N/A	2875	N/A	0.768	

Racemic sample (Daicel Chiralpak AS-H, 1% MeOH, 1.5 mL/min, 25 °C)

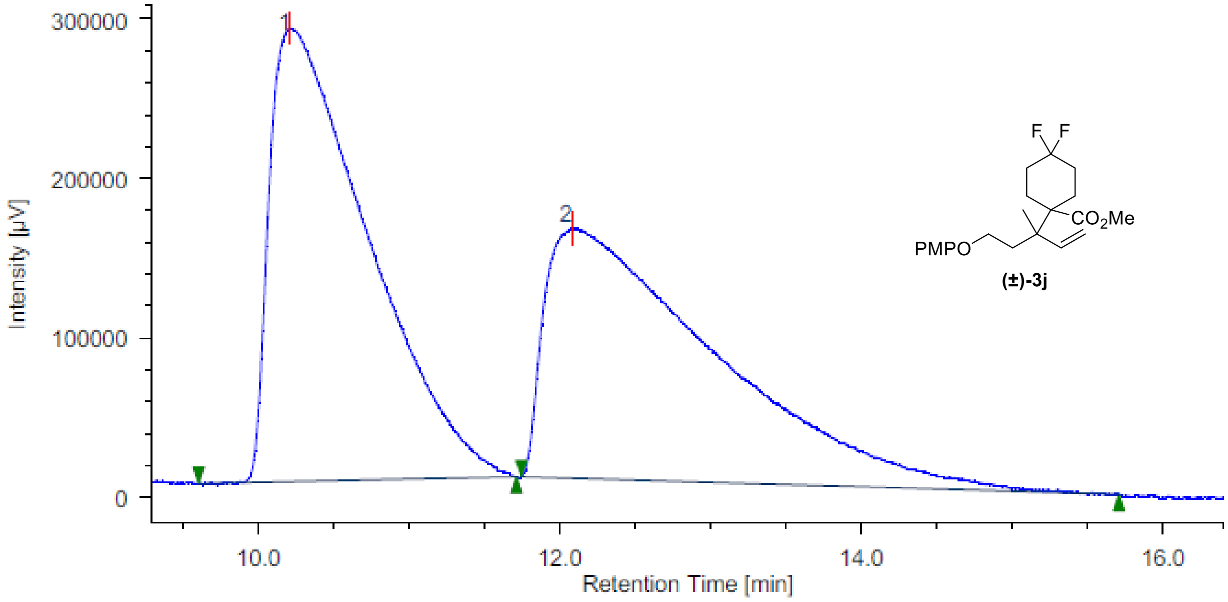

#	Peak Name	CH	tR [min]	Area [µV·sec]	Height [µV]	Area%	Height%	Quantity	NTP	Resolution	Symmetry Factor	Warning
1	Unknown	1	10.217	12850190	284153	51.022	64.455	N/A	1140	1.155	3.066	
2	Unknown	1	12.083	12335510	156704	48.978	35.545	N/A	567	N/A	4.575	

(+)-3j

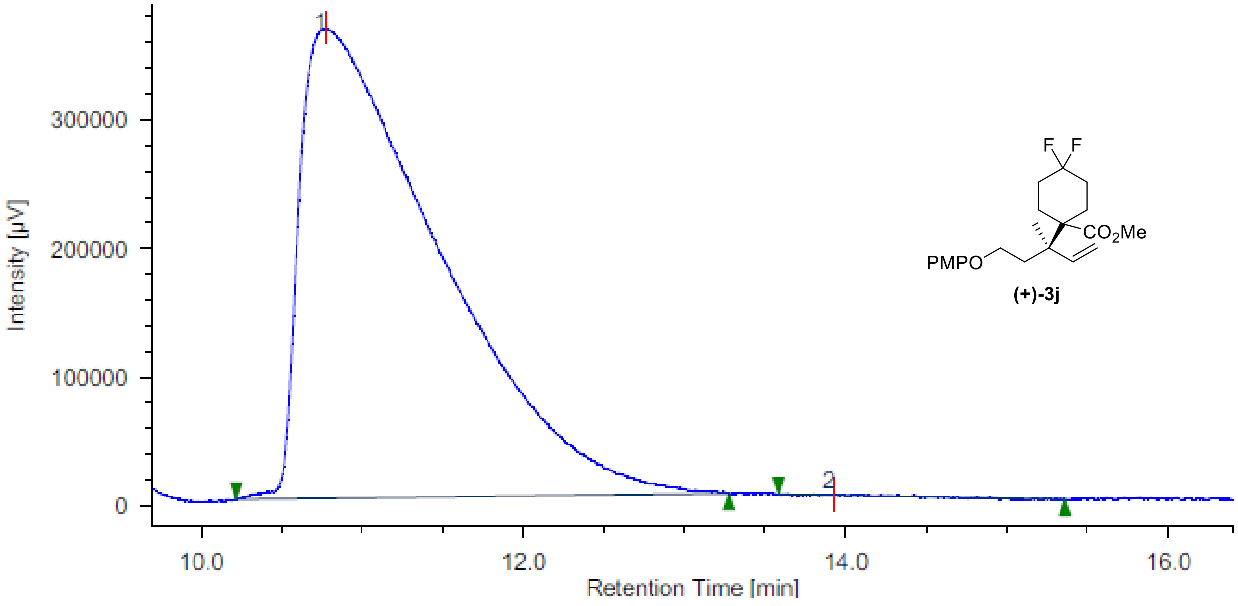

#	Peak Name	CH	tR [min]	Area [µV·sec]	Height [µV]	Area%	Height%	Quantity	NTP	Resolution	Symmetry Factor	Warning
1	Unknown	1	10.775	21963526	365113	99.929	99.762	N/A	752	1.650	3.807	
2	Unknown	1	13.933	15653	869	0.071	0.238	N/A	605	N/A	2.892	

Racemic sample (Daicel Chiralpak IA, 5% MeOH, 2.0 mL/min, 25 °C)

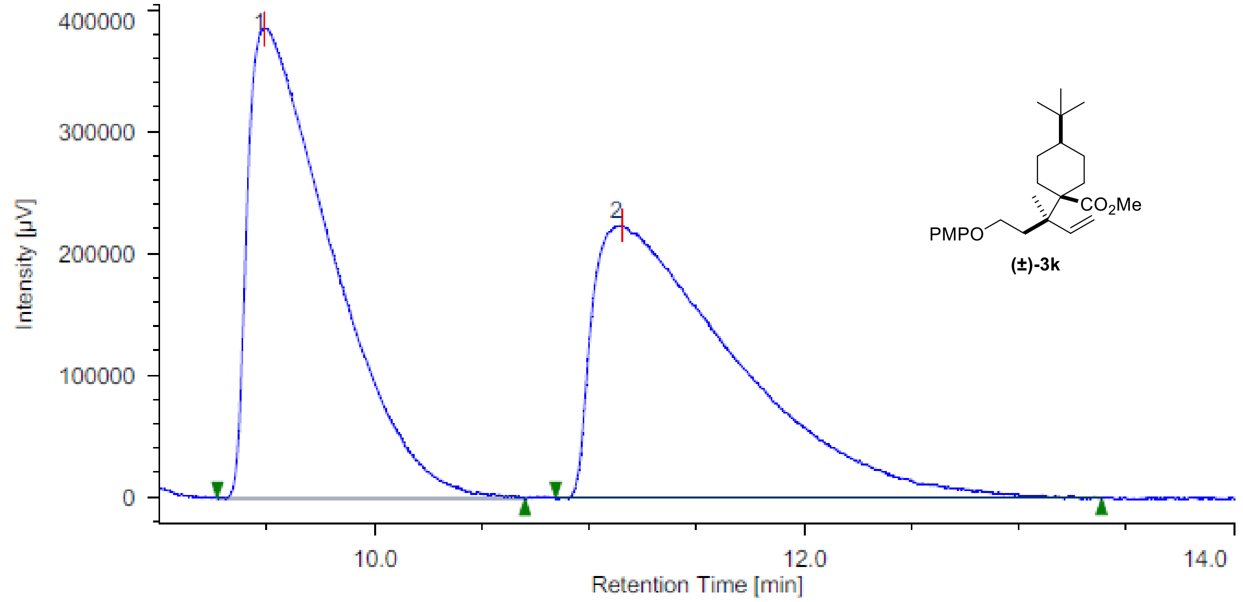

#	Peak Name	CH	tR [min]	Area [µV·sec]	Height [µV]	Area%	Height%	Quantity	NTP	Resolution	Symmetry Factor	Warning
1	Unknown	1	9.492	10533532	385758	50.294	63.266	N/A	2804	1.743	3.255	
2	Unknown	1	11.150	10410549	223982	49.706	36.734	N/A	1402	N/A	3.878	

(+)-3k

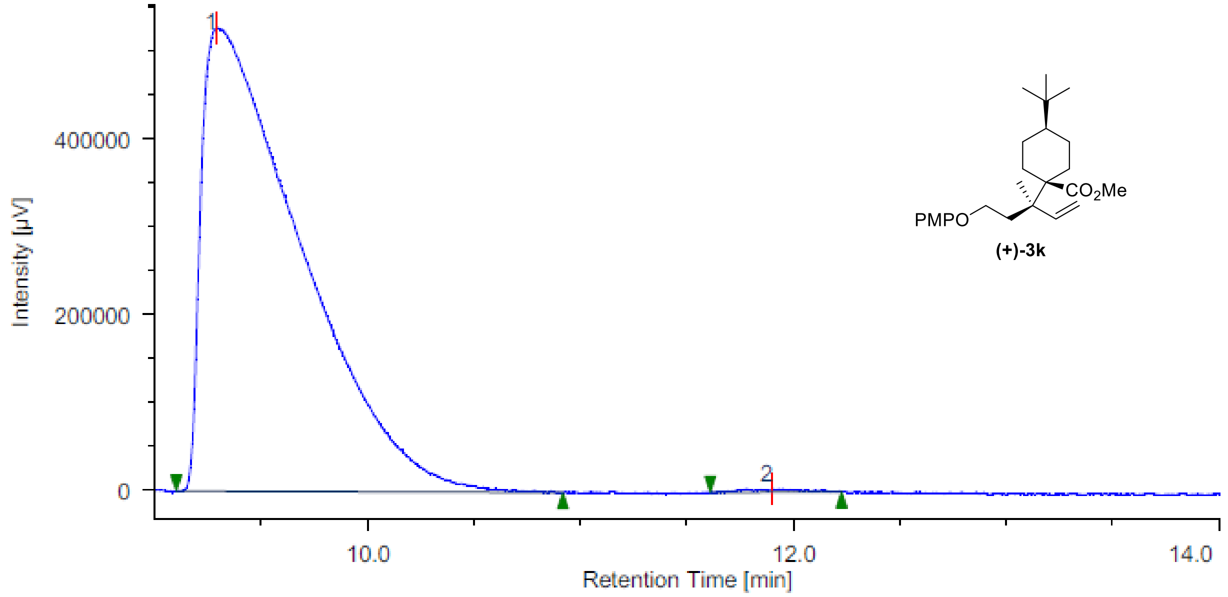

#	Peak Name	CH	tR [min]	Area [µV·sec]	Height [µV]	Area%	Height%	Quantity	NTP	Resolution	Symmetry Factor	Warning
1	Unknown	1	9.292	17137022	526618	99.625	99.241	N/A	1877	3.864	4.351	
2	Unknown	1	11.900	64439	4026	0.375	0.759	N/A	9214	N/A	1.059	

Racemic sample (Daicel Chiralcel OJ-H, 5% MeOH, 2.0 mL/min, 25 °C)

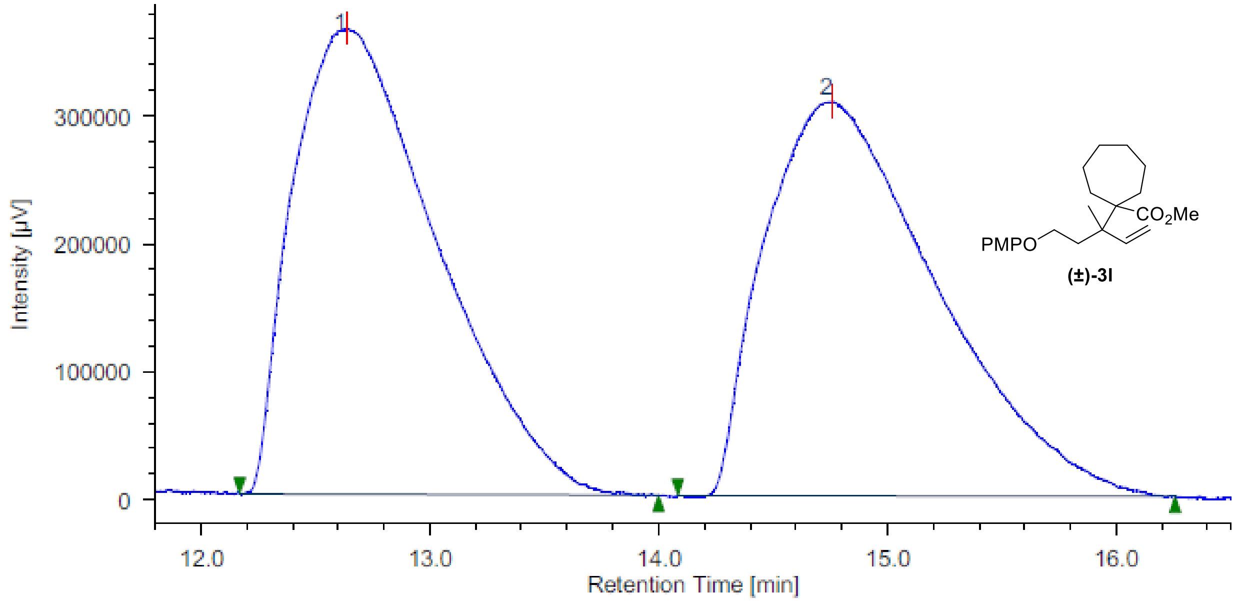

#	Peak Name	CH	tR [min]	Area [µV·sec]	Height [µV]	Area%	Height%	Quantity	NTP	Resolution	Symmetry Factor	Warning
1	Unknown	1	12.633	15890054	363412	49.762	54.132	N/A	1749	1.614	1.724	
2	Unknown	1	14.758	16042087	307938	50.238	45.868	N/A	1700	N/A	1.666	

(+)-3l

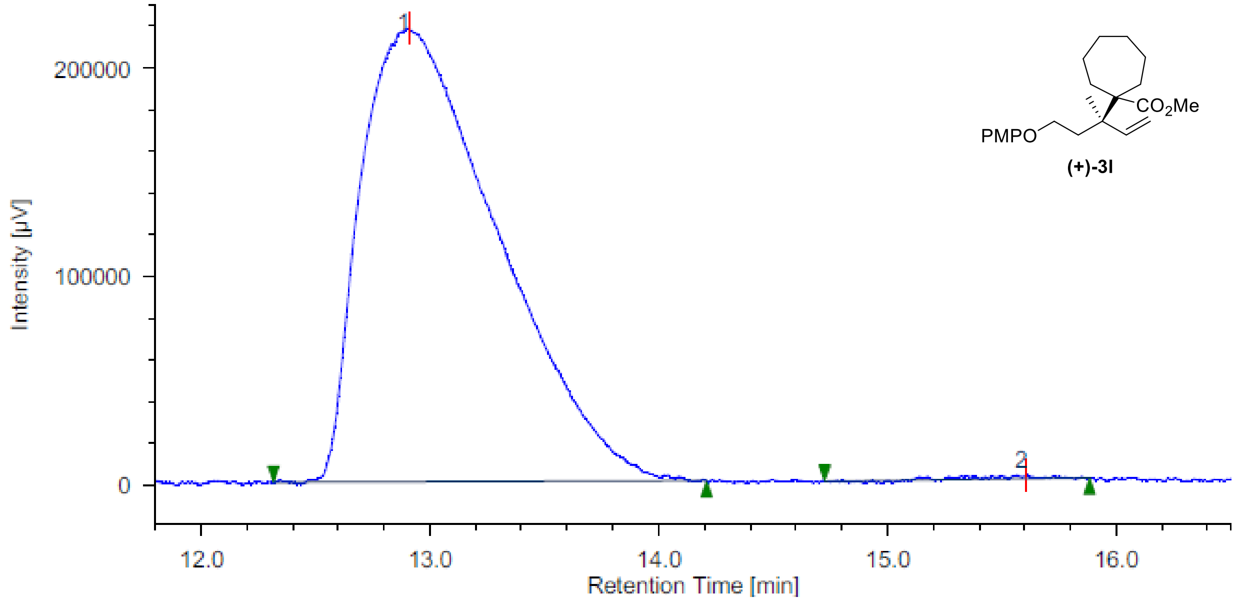

#	Peak Name	CH	tR [min]	Area [µV·sec]	Height [µV]	Area%	Height%	Quantity	NTP	Resolution	Symmetry Factor	Warning
1	Unknown	1	12.908	9040878	217210	99.646	99.218	N/A	2027	2.490	1.824	
2	Unknown	1	15.608	32096	1711	0.354	0.782	N/A	3689	N/A	0.648	

Racemic sample (Daicel Chiralcel AS-H, 3% MeOH, 0.8 mL/min, 25 °C), 1:1 dr

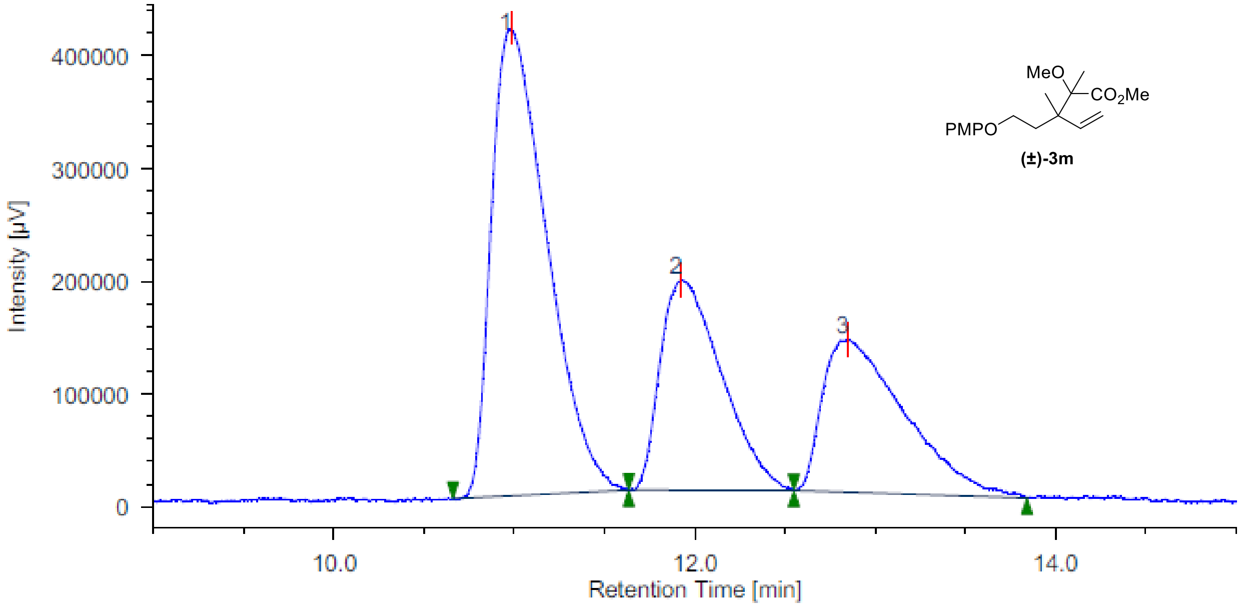

#	Peak Name	CH	tR [min]	Area [µV·sec]	Height [µV]	Area%	Height%	Quantity	NTP	Resolution	Symmetry Factor	Warning
1	Unknown	1	10.983	8887724	415074	50.558	56.283	N/A	5793	1.549	1.668	
2	Unknown	1	11.925	4408774	186905	25.079	25.344	N/A	5521	1.254	1.661	
3	Unknown	1	12.850	4282871	135502	24.363	18.374	N/A	3773	N/A	2.153	

(+)-3m, 1:1 dr

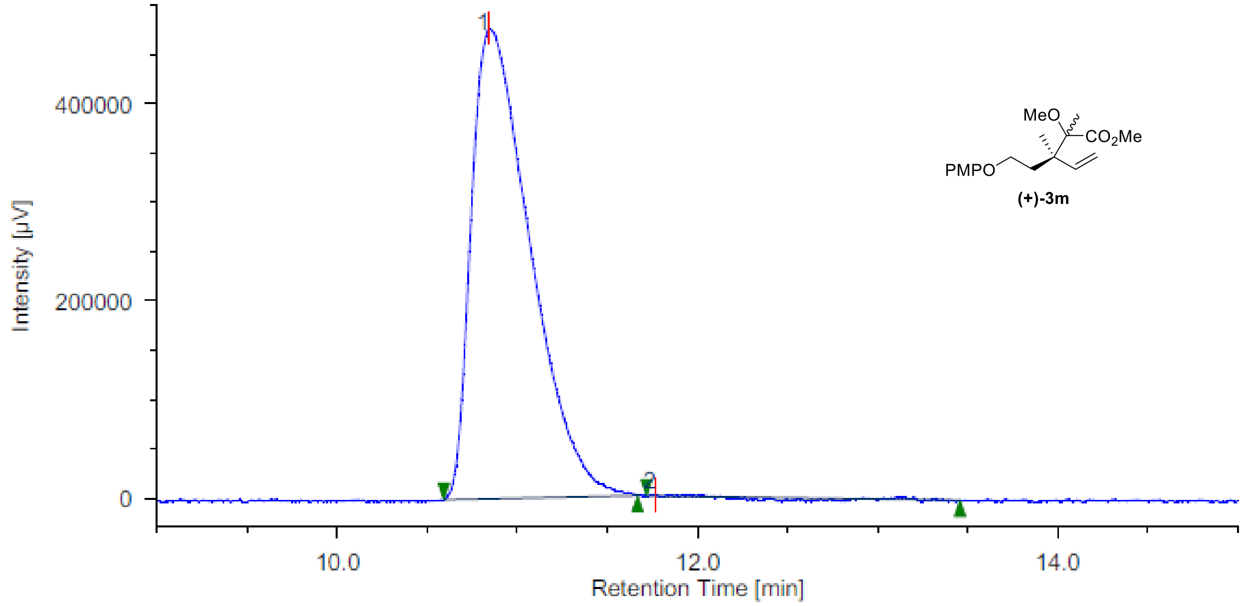

#	Peak Name	CH	tR [min]	Area [µV·sec]	Height [µV]	Area%	Height%	Quantity	NTP	Resolution	Symmetry Factor	Warning
1	Unknown	1	10.842	10860959	477037	99.710	99.718	N/A	5122	0.563	1.940	
2	Unknown	1	11.767	31603	1350	0.290	0.282	N/A	306	N/A	23.126	

Racemic sample (Daicel Chiralcel AS-H, 10% MeOH, 2 mL/min, 25 °C), 1.2:1 dr

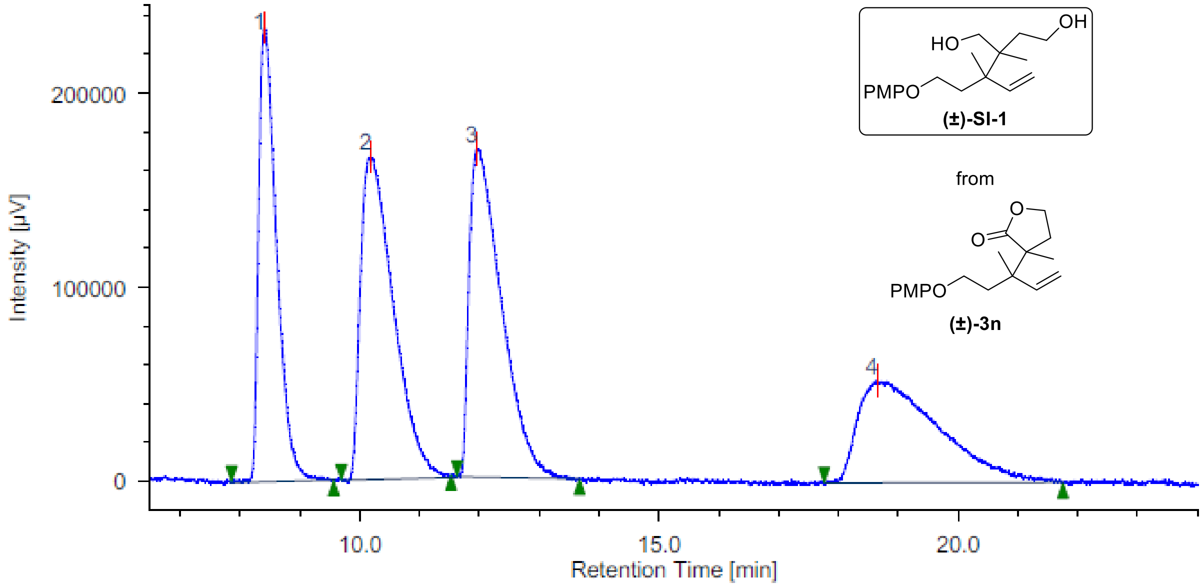

#	Peak Name	CH	tR [min]	Area [µV·sec]	Height [µV]	Area%	Height%	Quantity	NTP	Resolution	Symmetry Factor	Warning
1	Unknown	1	8.417	4855464	233625	21.556	37.611	N/A	3878	2.265	1.837	
2	Unknown	1	10.183	6380644	166111	28.327	26.742	N/A	1585	1.761	2.107	
3	Unknown	1	11.958	6339147	169044	28.143	27.215	N/A	2296	3.855	2.618	
4	Unknown	1	18.650	4949505	52374	21.974	8.432	N/A	903	N/A	2.755	

(+)-SI-1 from (+)-3n, 1.2:1 dr

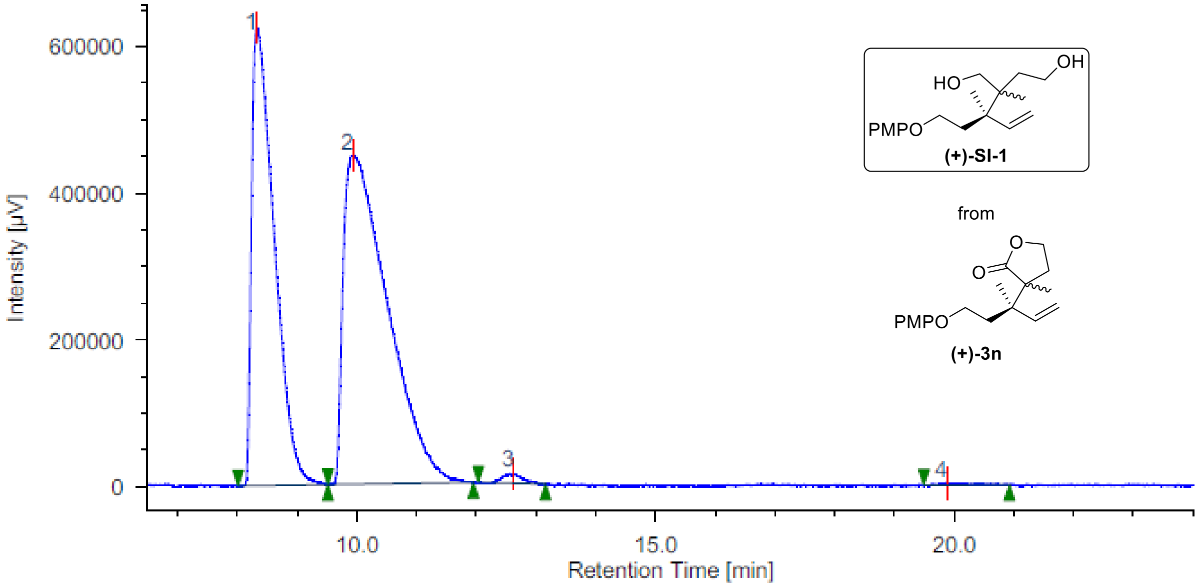

#	Peak Name	CH	tR [min]	Area [µV·sec]	Height [µV]	Area%	Height%	Quantity	NTP	Resolution	Symmetry Factor	Warning
1	Unknown	1	8.325	16344330	623770	40.961	57.340	N/A	2233	1.539	2.773	
2	Unknown	1	9.933	23184999	448417	58.105	41.221	N/A	816	2.644	3.083	
3	Unknown	1	12.625	293670	12762	0.736	1.173	N/A	6023	6.280	1.327	
4	Unknown	1	19.875	79098	2897	0.198	0.266	N/A	2281	N/A	1.935	

Racemic sample (Daicel Chiralcel OJ-H, 1% MeOH, 2.0 mL/min, 25 °C)

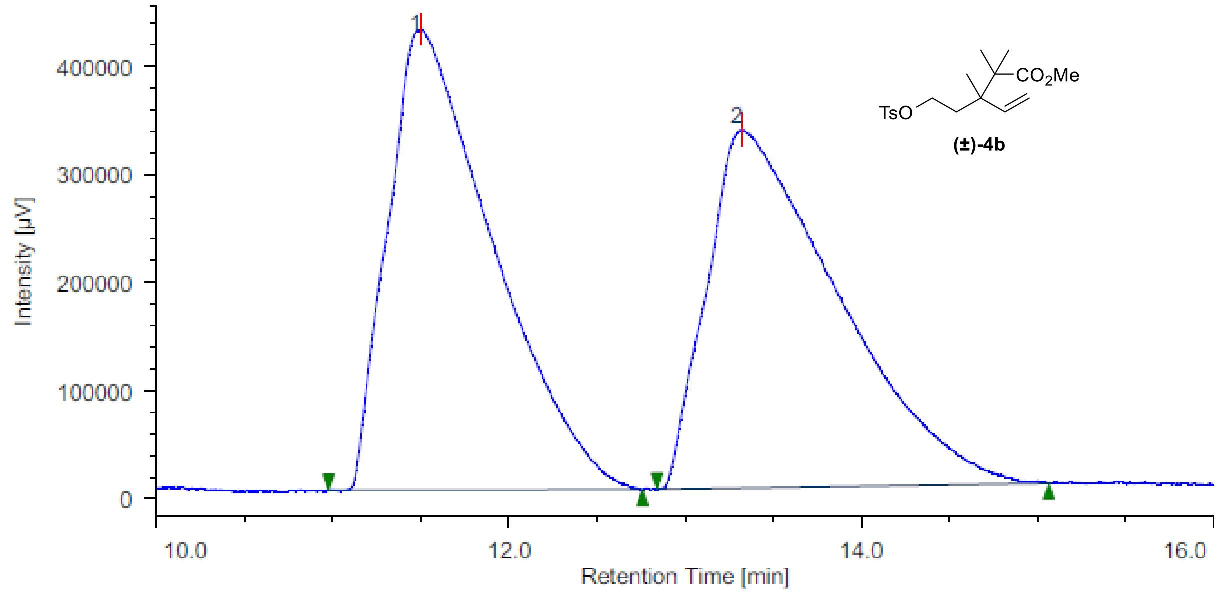

#	Peak Name	CH	tR [min]	Area [µV·sec]	Height [µV]	Area%	Height%	Quantity	NTP	Resolution	Symmetry Factor	Warning
1	Unknown	1	11.500	17679467	426154	50.765	56.266	N/A	1710	1.474	1.880	
2	Unknown	1	13.325	17146956	331238	49.235	43.734	N/A	1512	N/A	2.151	

(+)-4b

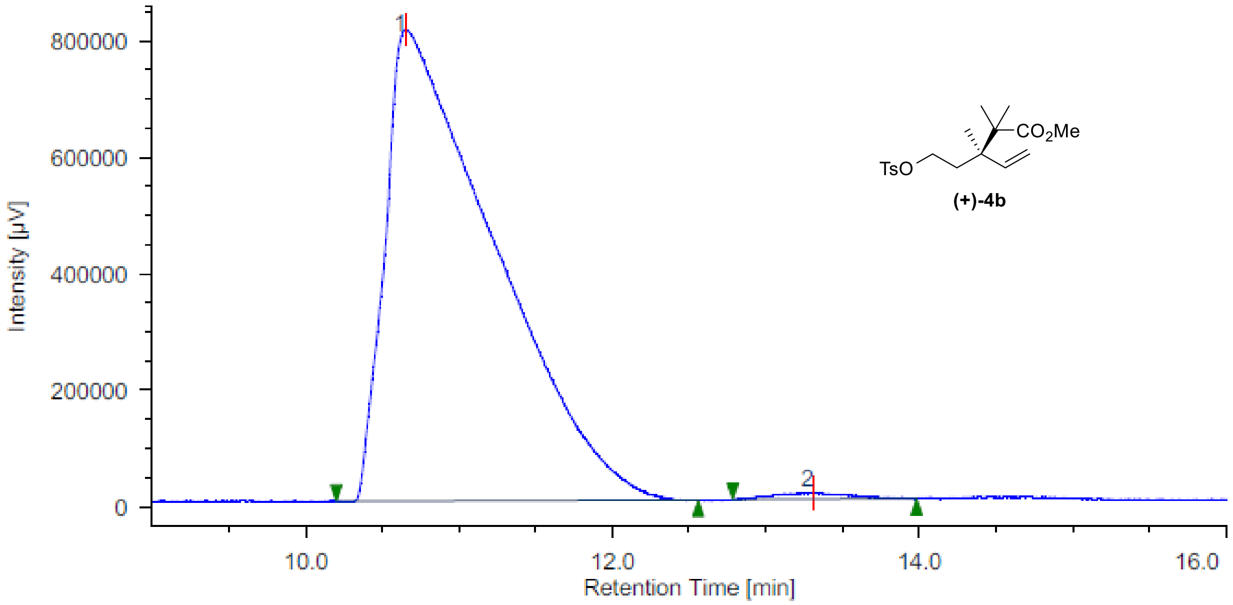

#	Peak Name	CH	tR [min]	Area [µV·sec]	Height [µV]	Area%	Height%	Quantity	NTP	Resolution	Symmetry Factor	Warning
1	Unknown	1	10.650	40172929	809513	99.090	98.648	N/A	1033	2.328	2.939	
2	Unknown	1	13.308	368746	11093	0.910	1.352	N/A	3047	N/A	1.108	

Racemic sample (Daicel Chiralcel AS-H, 3% MeOH, 2.0 mL/min, 25 °C)

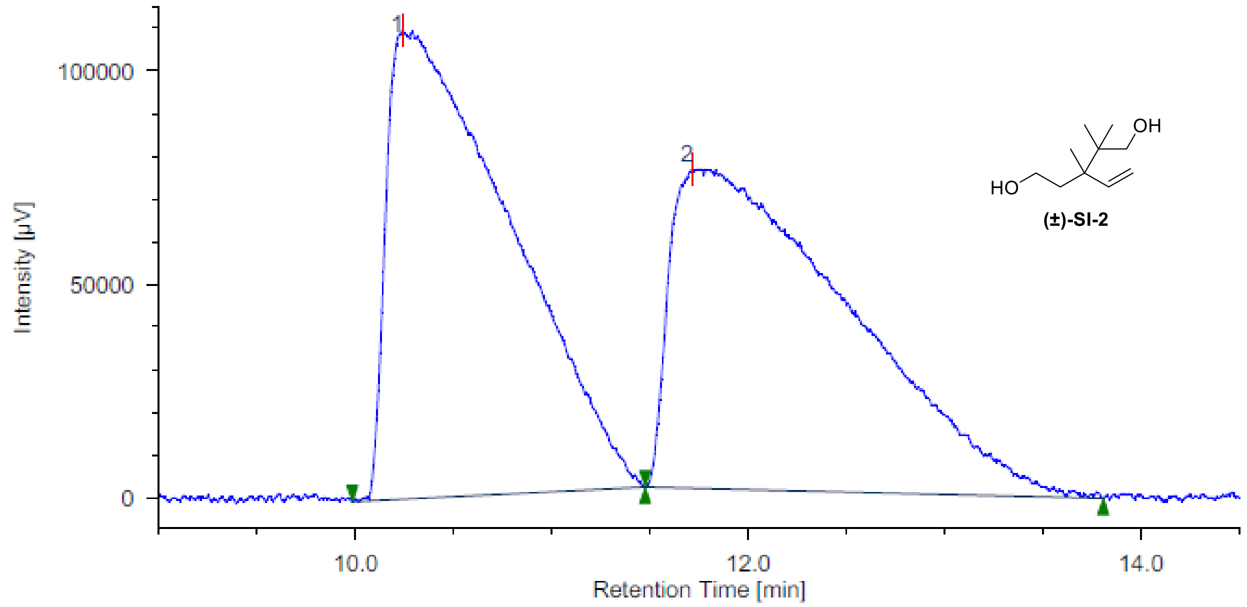

#	Peak Name	CH	tR [min]	Area [µV·sec]	Height [µV]	Area%	Height%	Quantity	NTP	Resolution	Symmetry Factor	Warning
1	Unknown	1	10.242	4778561	109830	50.004	59.504	N/A	1099	0.978	4.135	
2	Unknown	1	11.717	4777832	74747	49.996	40.496	N/A	687	N/A	4.670	

(+)-SI-2 from (+)-4c

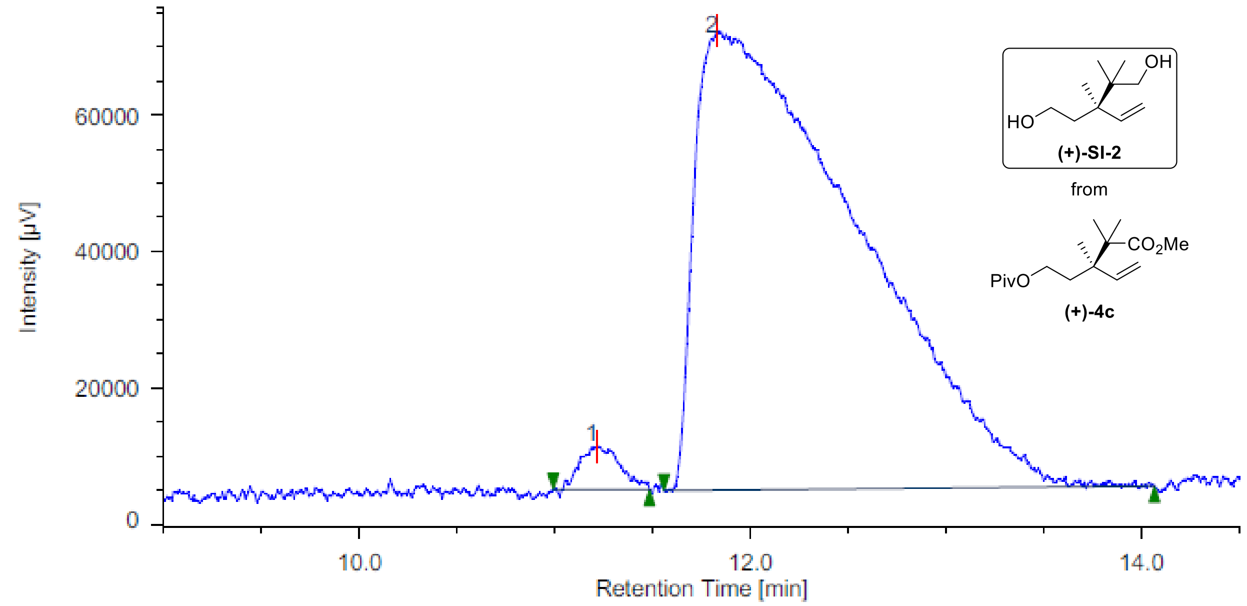

#	Peak Name	CH	tR [min]	Area [µV·sec]	Height [µV]	Area%	Height%	Quantity	NTP	Resolution	Symmetry Factor	Warning
2	Unknown	1	11.217	90128	6277	2.250	8.533	N/A	12553	0.607	1.263	
1	Unknown	1	11.833	3916005	67282	97.750	91.467	N/A	838	N/A	4.472	

(+)-SI-2 from (+)-4f

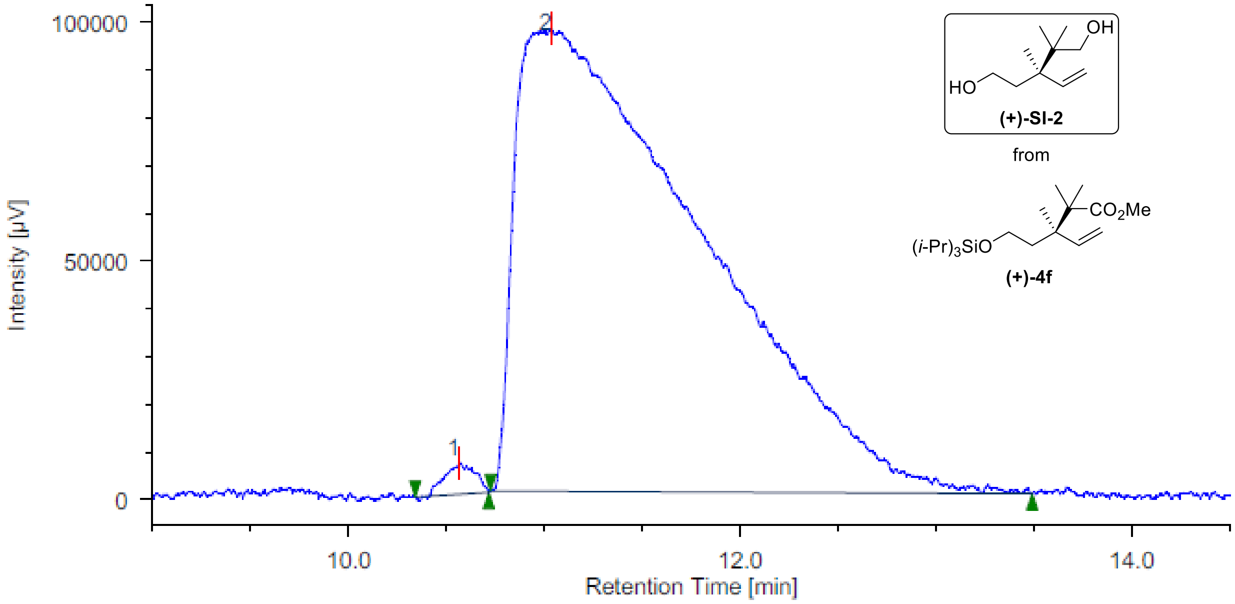

#	Peak Name	CH	tR [min]	Area [µV·sec]	Height [µV]	Area%	Height%	Quantity	NTP	Resolution	Symmetry Factor	Warning
2	Unknown	1	10.567	69674	6513	1.076	6.307	N/A	15863	0.433	0.984	
1	Unknown	1	11.033	6407205	96749	98.924	93.693	N/A	584	N/A	3.802	

Racemic sample (Daicel Chiralcel OJ-H, 2% MeOH, 2.0 mL/min, 25 °C)

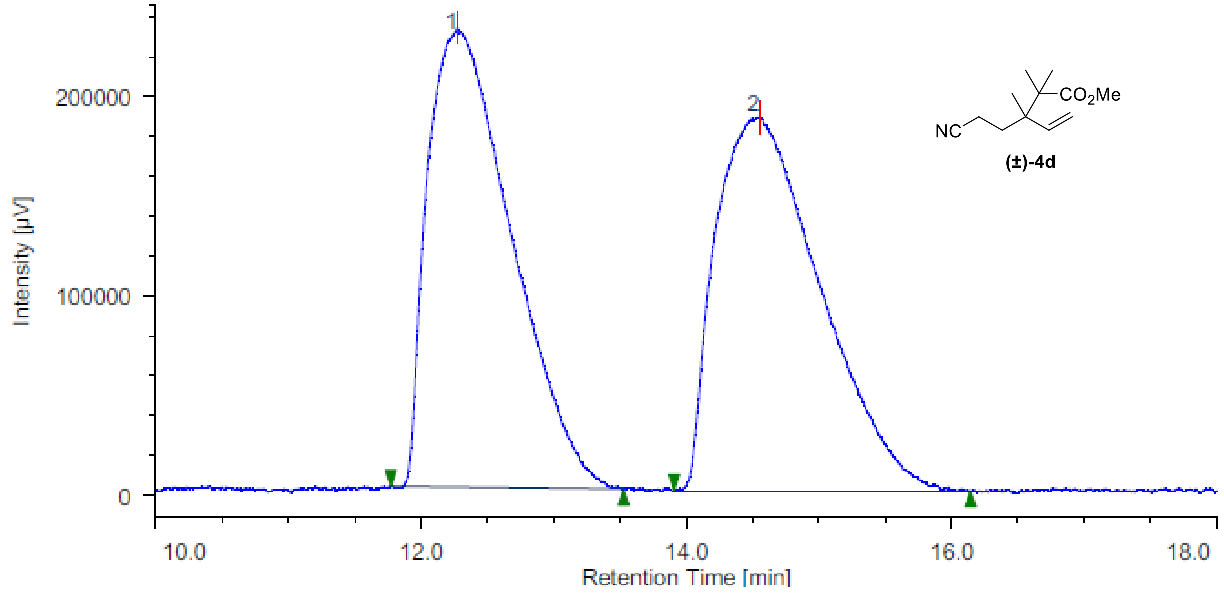

#	Peak Name	CH	tR [min]	Area [µV·sec]	Height [µV]	Area%	Height%	Quantity	NTP	Resolution	Symmetry Factor	Warning
1	Unknown	1	12.275	10089086	230382	49.560	55.177	N/A	1633	1.662	1.816	
2	Unknown	1	14.550	10268407	187148	50.440	44.823	N/A	1448	N/A	1.586	

(+)-4d

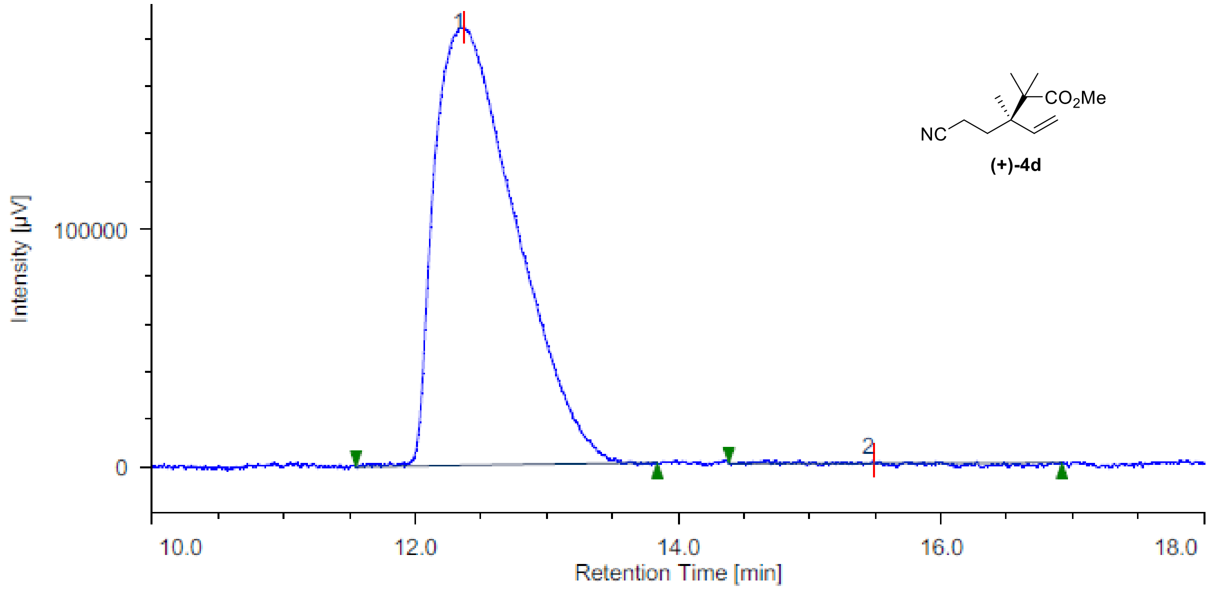

#	Peak Name	CH	tR [min]	Area [µV·sec]	Height [µV]	Area%	Height%	Quantity	NTP	Resolution	Symmetry Factor	Warning
1	Unknown	1	12.375	7944719	183992	99.758	99.383	N/A	1745	2.170	1.824	
2	Unknown	1	15.483	19245	1142	0.242	0.617	N/A	1346	N/A	1.167	

Racemic sample (Daicel Chiralcel OJ-H, 100% CO_2, 1.0 mL/min, 25 °C)

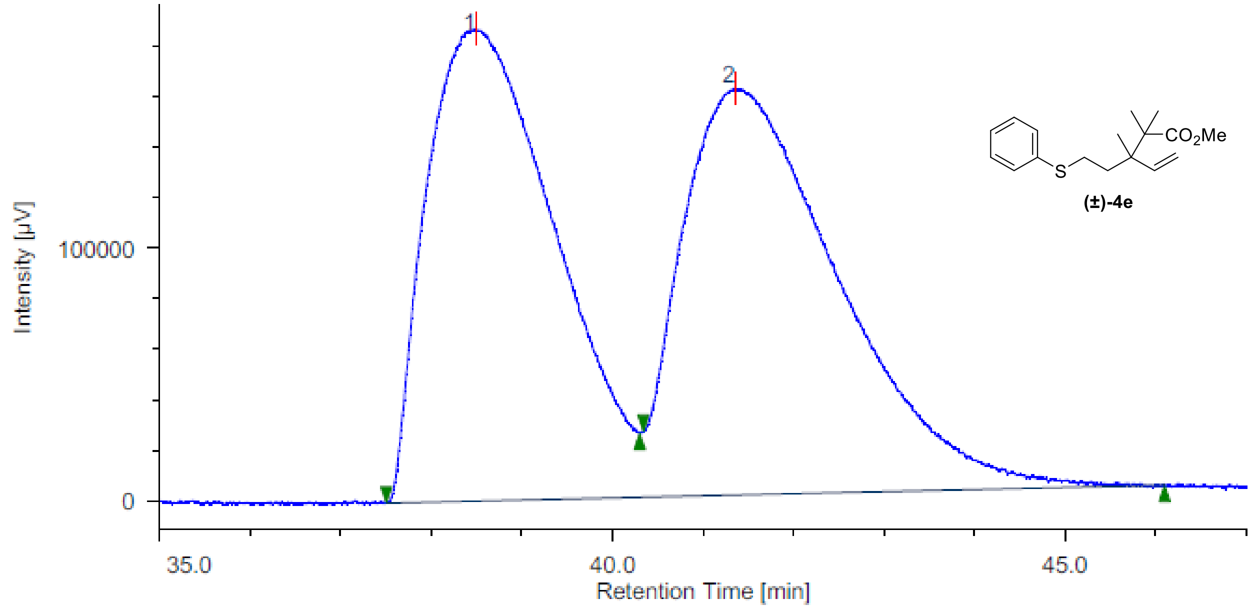

#	Peak Name	CH	tR [min]	Area [µV·sec]	Height [µV]	Area%	Height%	Quantity	NTP	Resolution	Symmetry Factor	Warning
1	Unknown	1	38.483	18283405	186847	48.517	53.717	N/A	3063	0.957	N/A	
2	Unknown	1	41.350	19401204	160990	51.483	46.283	N/A	2634	N/A	N/A	

(+)-4e

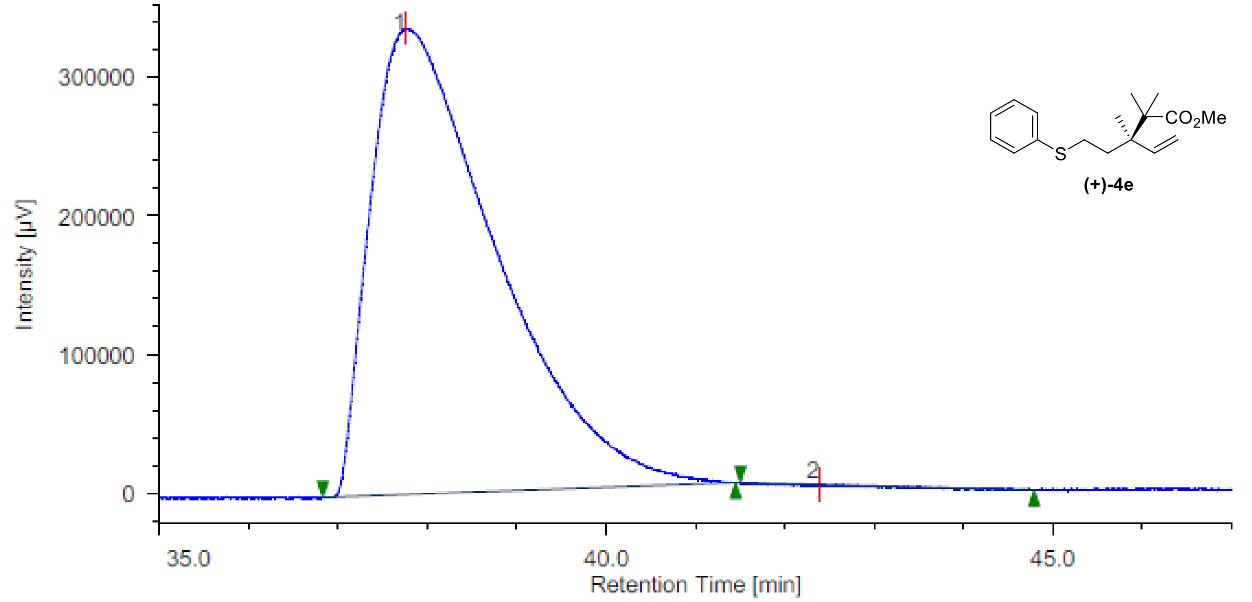

#	Peak Name	CH	tR [min]	Area [µV·sec]	Height [µV]	Area%	Height%	Quantity	NTP	Resolution	Symmetry Factor	Warning
1	Unknown	1	37.750	32809025	335301	99.983	99.919	N/A	3471	1.403	2.362	
2	Unknown	1	42.383	5466	272	0.017	0.081	N/A	1745	N/A	148.620	

Racemic sample (Daicel Chiralcel OJ-H, 2% MeOH, 2.0 mL/min, 25 °C)

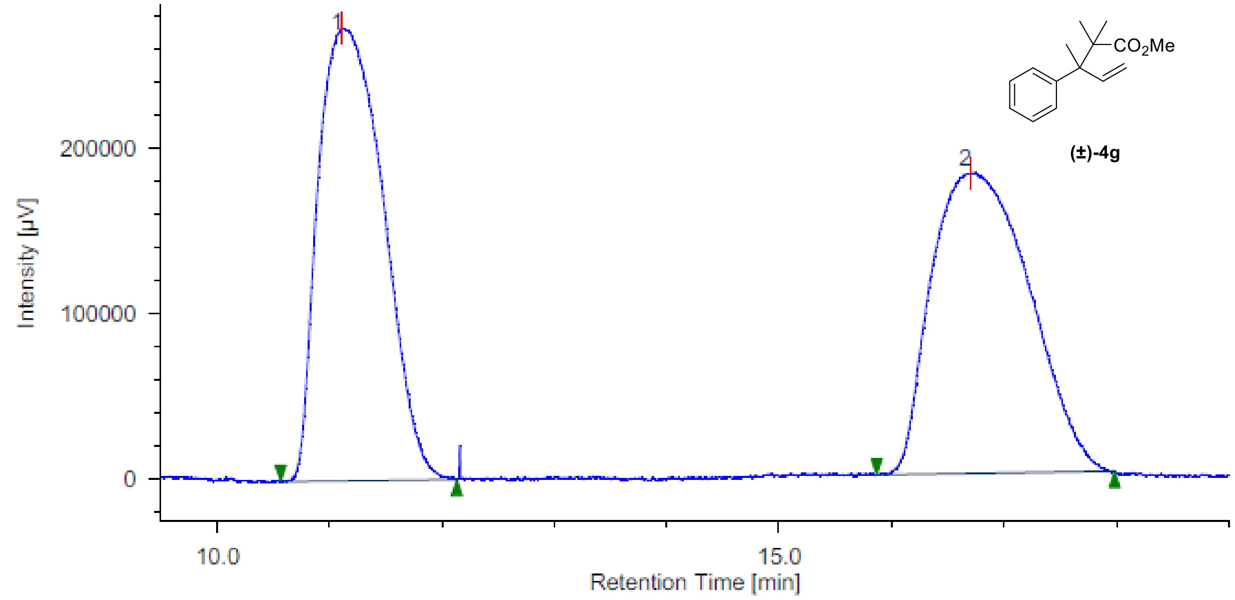

#	Peak Name	CH	tR [min]	Area [µV·sec]	Height [µV]	Area%	Height%	Quantity	NTP	Resolution	Symmetry Factor	Warning
1	Unknown	1	11.117	11044662	273807	49.959	60.084	N/A	1496	3.878	1.441	
2	Unknown	1	16.708	11062719	181899	50.041	39.916	N/A	1472	N/A	1.345	

(+)-4g

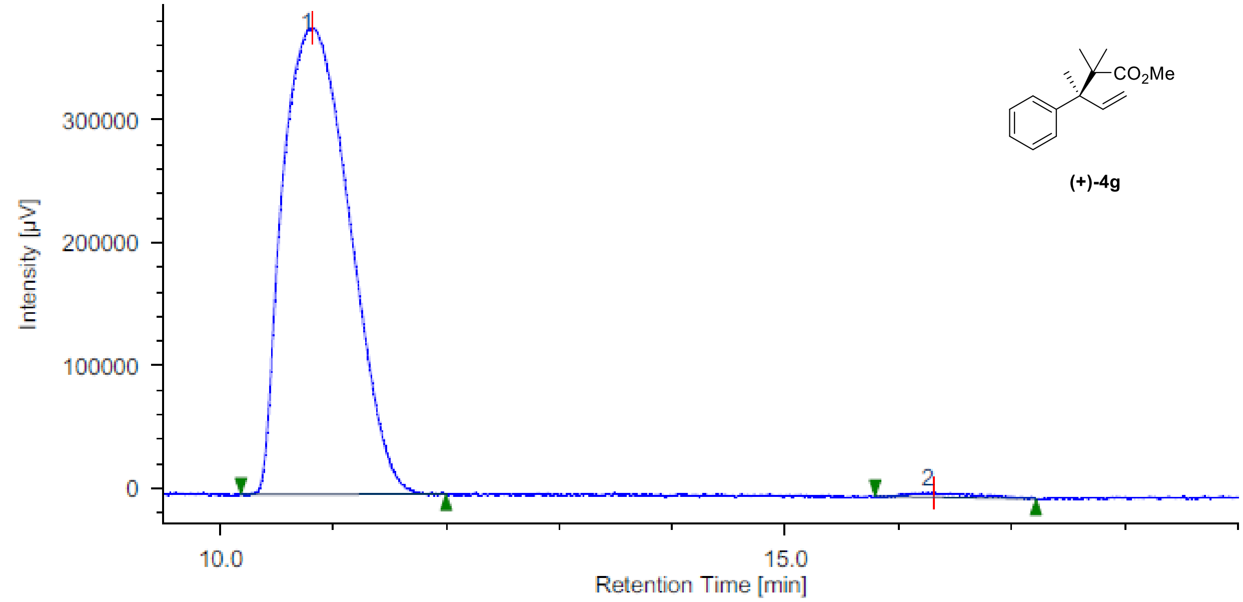

#	Peak Name	CH	tR [min]	Area [µV·sec]	Height [µV]	Area%	Height%	Quantity	NTP	Resolution	Symmetry Factor	Warning
1	Unknown	1	10.817	15589420	379528	99.024	99.062	N/A	1360	4.065	1.324	
2	Unknown	1	16.308	153678	3592	0.976	0.938	N/A	1803	N/A	1.380	

Racemic sample (Daicel Chiralpak IA, 2% MeOH, 2.0 mL/min, 25 °C)

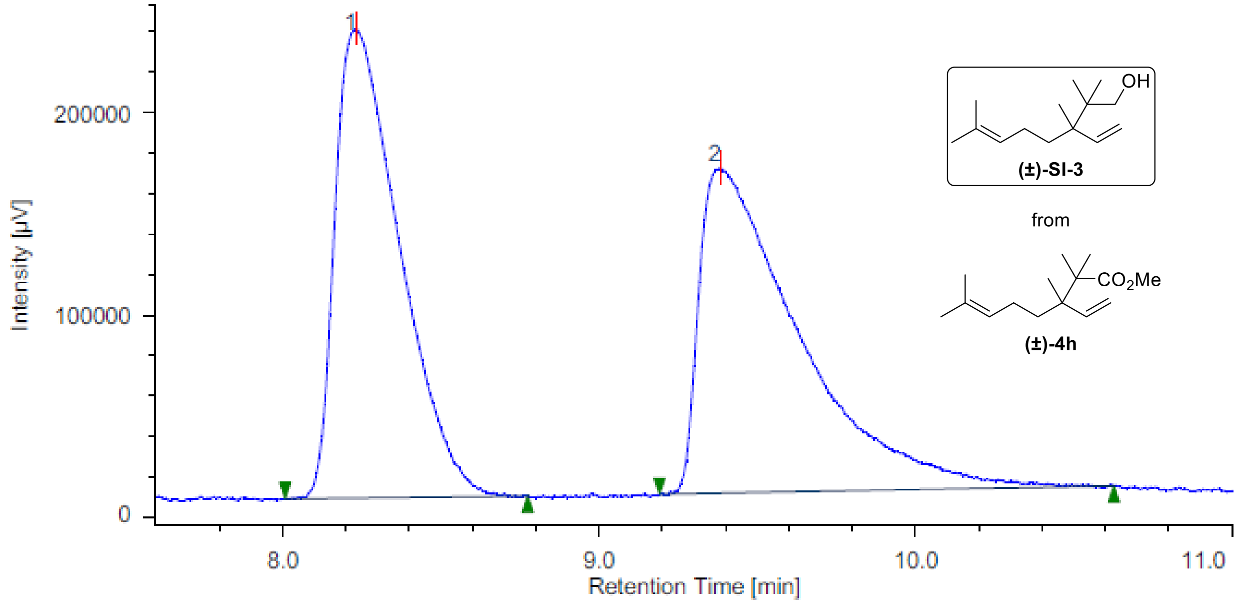

#	Peak Name	CH	tR [min]	Area [µV·sec]	Height [µV]	Area%	Height%	Quantity	NTP	Resolution	Symmetry Factor	Warning
1	Unknown	1	8.233	3281536	231803	48.192	59.072	N/A	7614	2.511	1.819	
2	Unknown	1	9.383	3527775	160605	51.808	40.928	N/A	4815	N/A	3.569	

(+)-SI-3 from (+)-4h

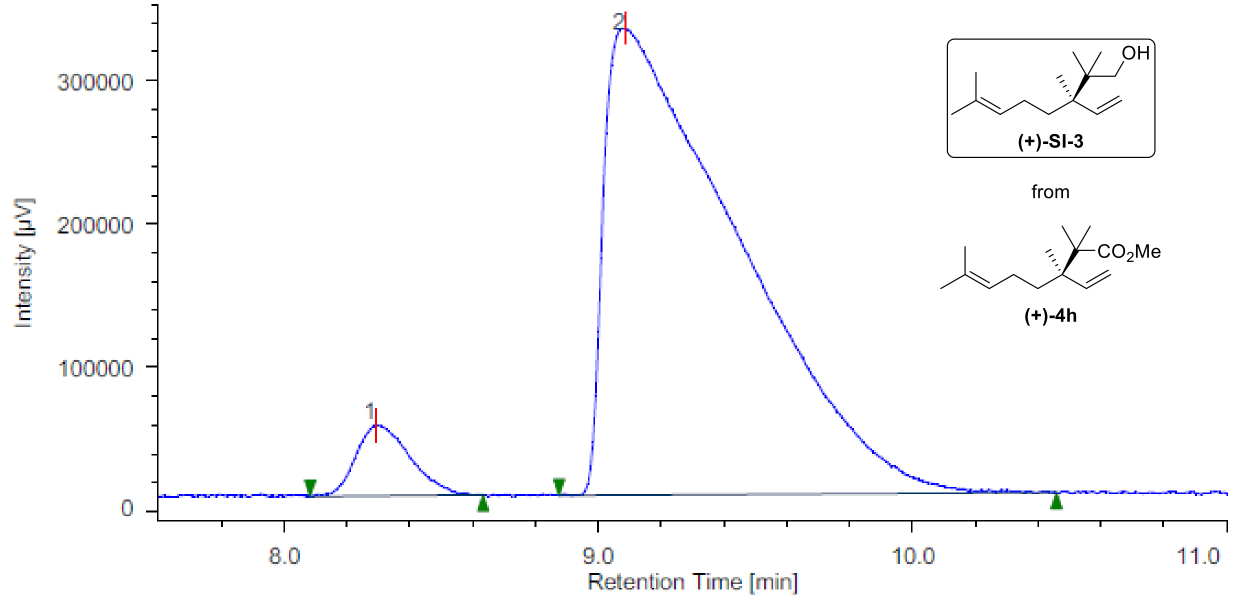

#	Peak Name	CH	tR [min]	Area [µV·sec]	Height [µV]	Area%	Height%	Quantity	NTP	Resolution	Symmetry Factor	Warning
1	Unknown	1	8.292	588165	49407	5.726	13.195	N/A	11017	1.411	1.382	
2	Unknown	1	9.083	9683903	325045	94.274	86.805	N/A	2018	N/A	4.479	

Racemic sample (Daicel Chiralcel OJ-H, 5% MeOH, 2.0 mL/min, 25 °C)

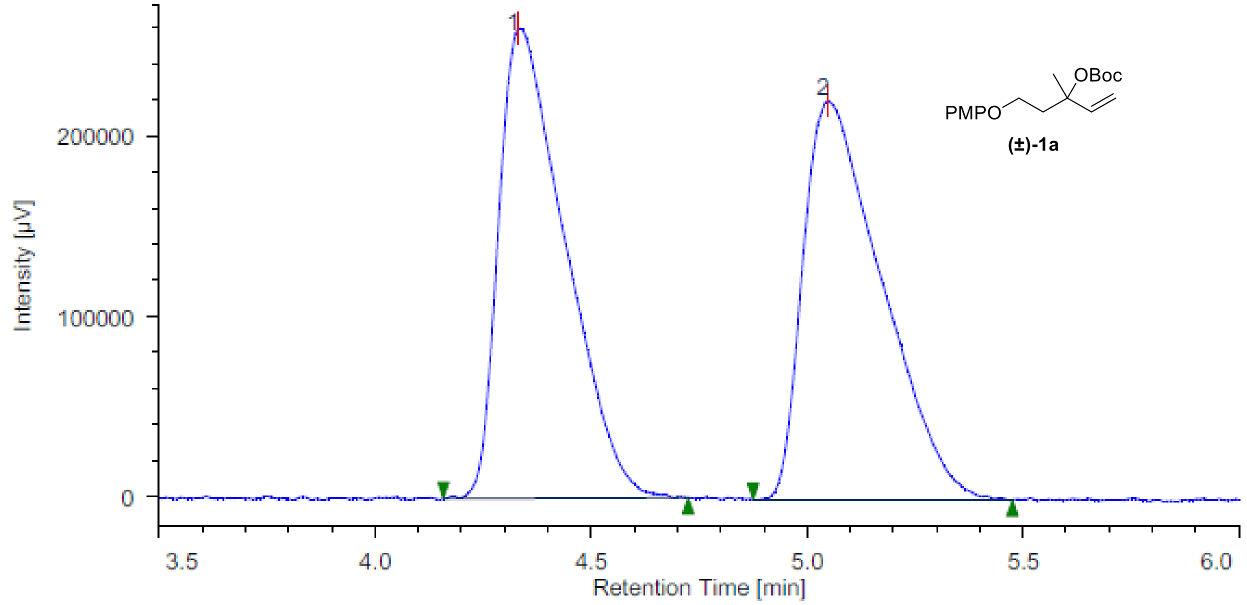

#	Peak Name	CH	tR [min]	Area [μV·sec]	Height [μV]	Area%	Height%	Quantity	NTP	Resolution	Symmetry Factor	Warning
1	Unknown	1	4.333	2790754	260323	49.923	54.084	N/A	3562	2.265	1.783	
2	Unknown	1	5.050	2799324	221008	50.077	45.916	N/A	3444	N/A	1.773	

(+)-1a

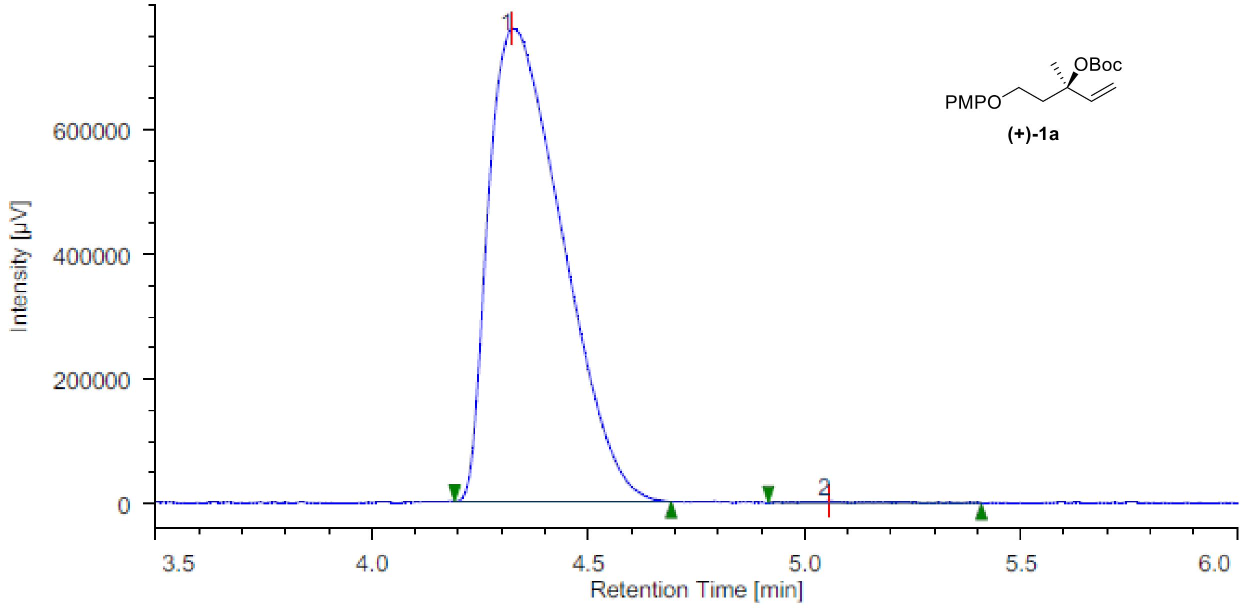

#	Peak Name	CH	tR [min]	Area [μV·sec]	Height [μV]	Area%	Height%	Quantity	NTP	Resolution	Symmetry Factor	Warning
1	Unknown	1	4.325	9001308	758486	99.607	99.618	N/A	2776	2.014	1.784	
2	Unknown	1	5.058	35524	2908	0.393	0.382	N/A	2535	N/A	1.745	

Racemic sample (Daicel Chiralpak IA, 2% MeOH, 2.0 mL/min, 25 °C)

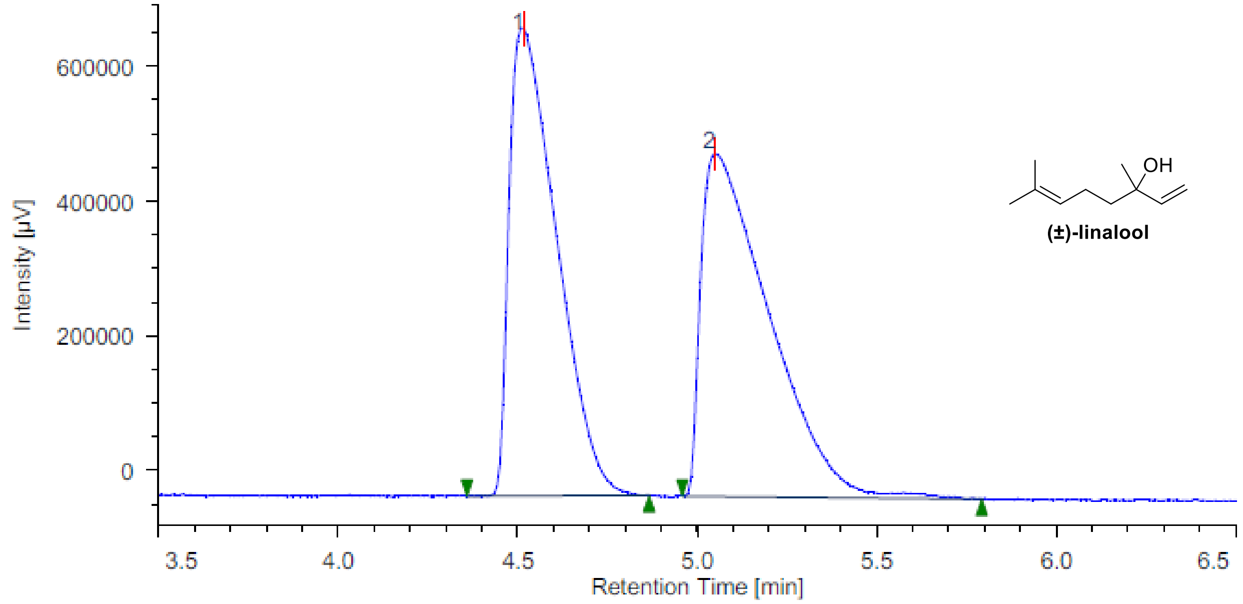

#	Peak Name	CH	tR [min]	Area [μV·sec]	Height [μV]	Area%	Height%	Quantity	NTP	Resolution	Symmetry Factor	Warning
1	Unknown	1	4.517	6382109	693381	48.738	57.699	N/A	5201	1.778	2.057	
2	Unknown	1	5.050	6712665	508348	51.262	42.301	N/A	3311	N/A	3.131	

(–)-linalool

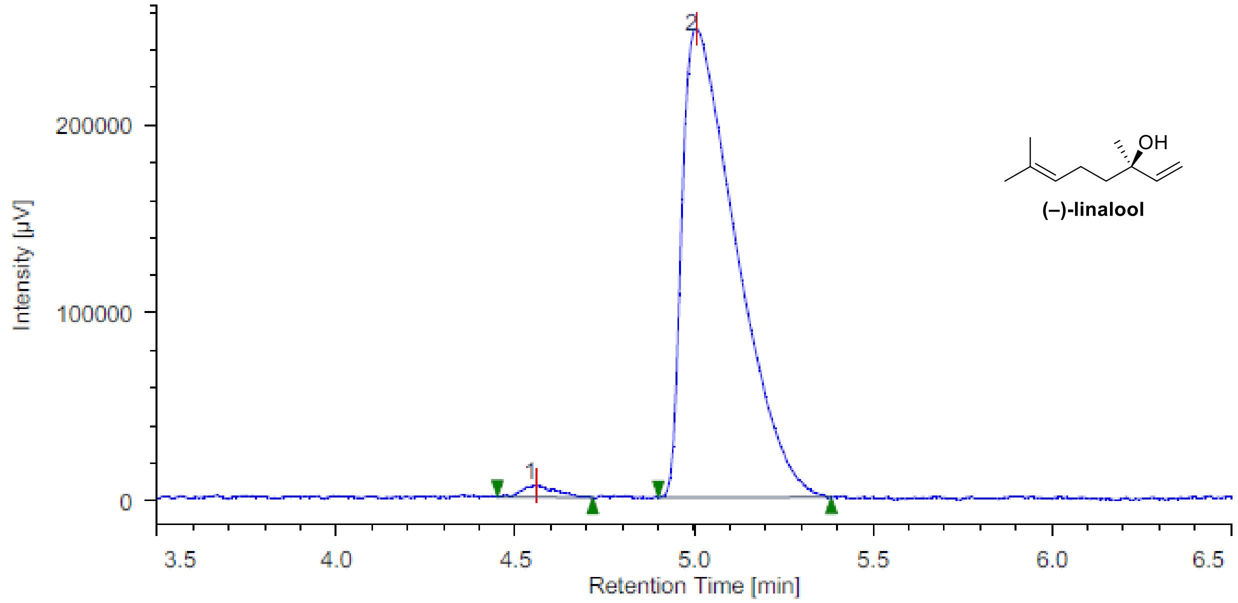

#	Peak Name	CH	tR [min]	Area [μV·sec]	Height [μV]	Area%	Height%	Quantity	NTP	Resolution	Symmetry Factor	Warning
1	Unknown	1	4.558	41273	6393	1.549	2.500	N/A	10892	1.988	1.264	
2	Unknown	1	5.008	2624094	249352	98.451	97.500	N/A	5144	N/A	2.340	

Racemic sample (Daicel Chiralcel OJ-H, 1% MeOH, 2.0 mL/min, 25 °C)

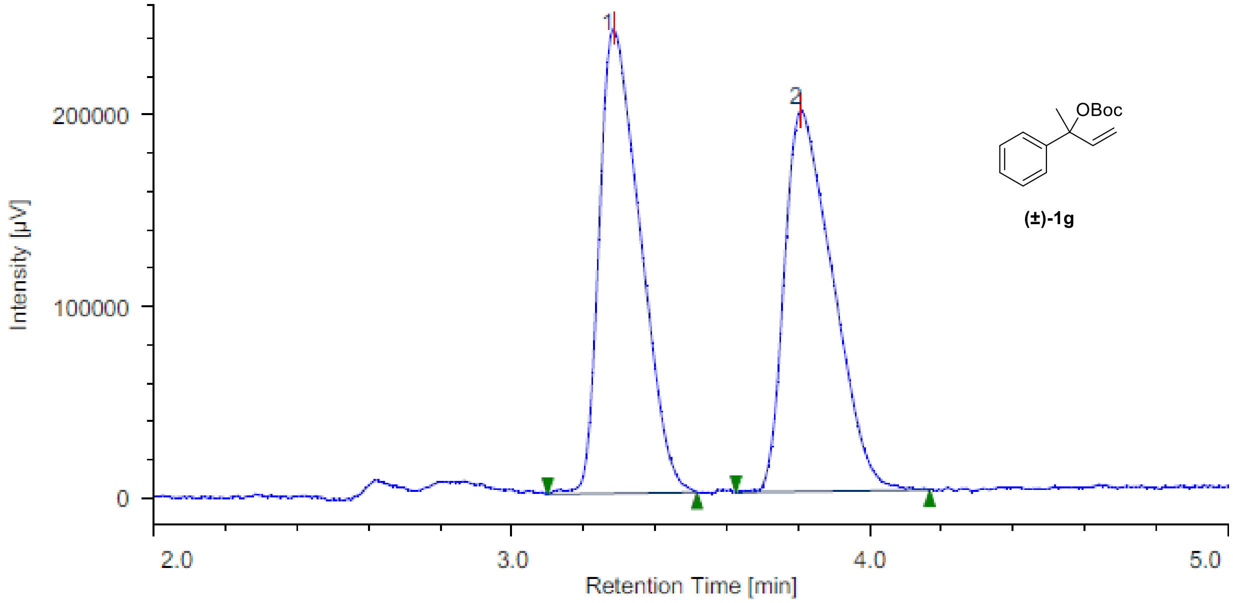

#	Peak Name	CH	tR [min]	Area [µV·sec]	Height [µV]	Area%	Height%	Quantity	NTP	Resolution	Symmetry Factor	Warning
1	Unknown	1	3.283	1929621	242591	50.832	54.987	N/A	3624	2.216	1.547	
2	Unknown	1	3.808	1866459	198589	49.168	45.013	N/A	3514	N/A	1.545	

(–)-1g

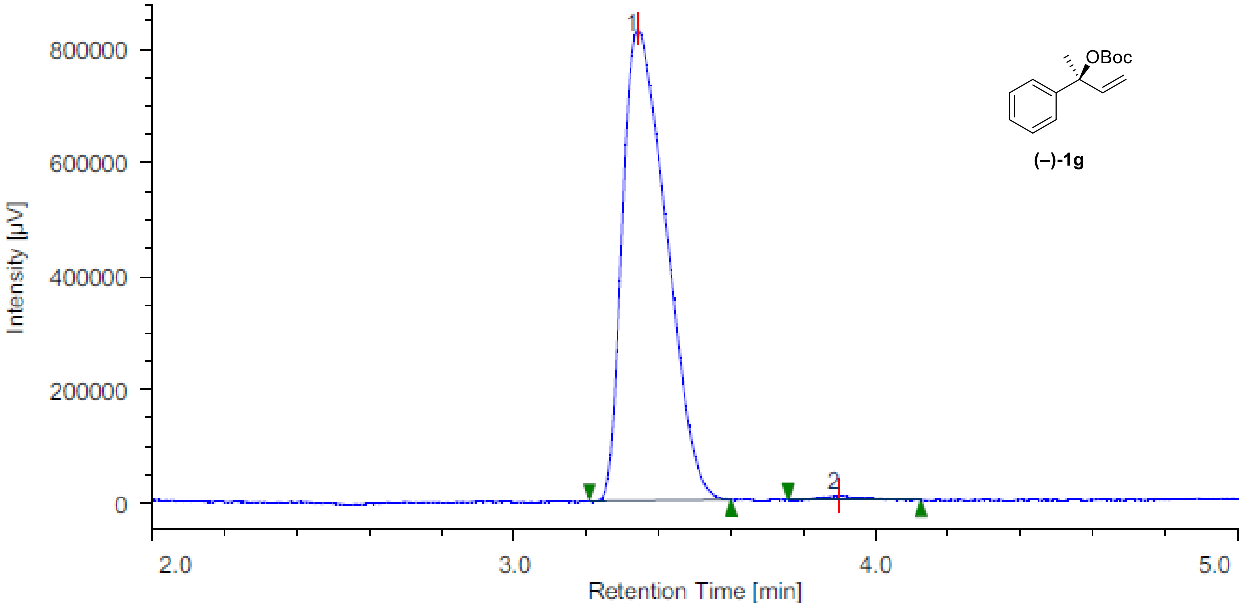

#	Peak Name	CH	tR [min]	Area [µV·sec]	Height [µV]	Area%	Height%	Quantity	NTP	Resolution	Symmetry Factor	Warning
1	Unknown	1	3.342	7000709	830657	99.090	99.113	N/A	3232	2.375	1.552	
2	Unknown	1	3.900	64311	7436	0.910	0.887	N/A	4358	N/A	1.578	

8. HPLC Traces

Racemic sample (Daicel Chiralpak AD-H, 30% *i*-PrOH, 1.0 mL/min, 25 °C, 250 nm)

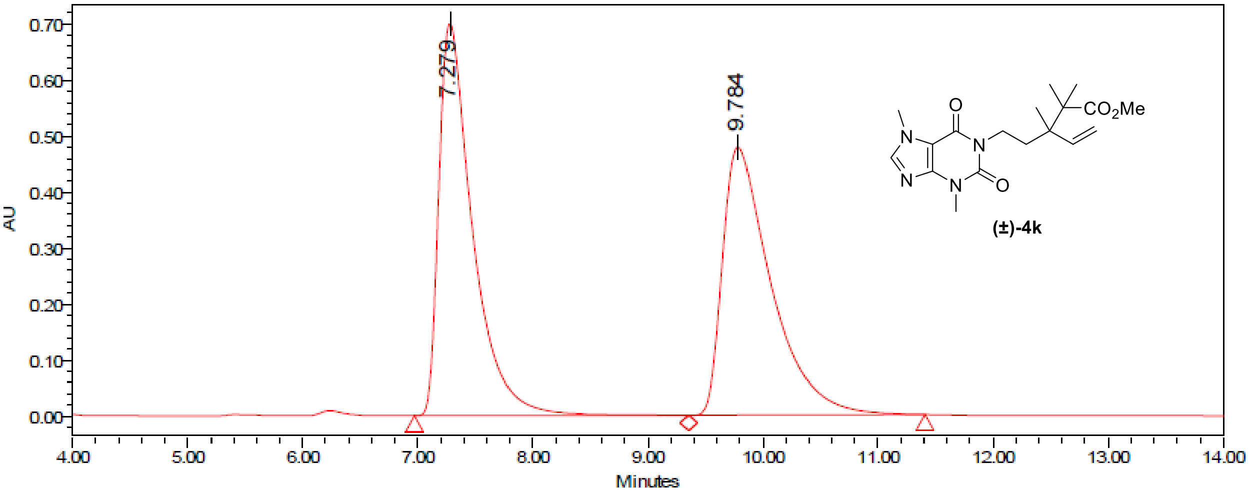

Peak Results

	Name	RT	Area	% Area	Height	Units
1		7.279	15362688	50.34	699522	
2		9.784	15153462	49.66	478252	

(+)-4k

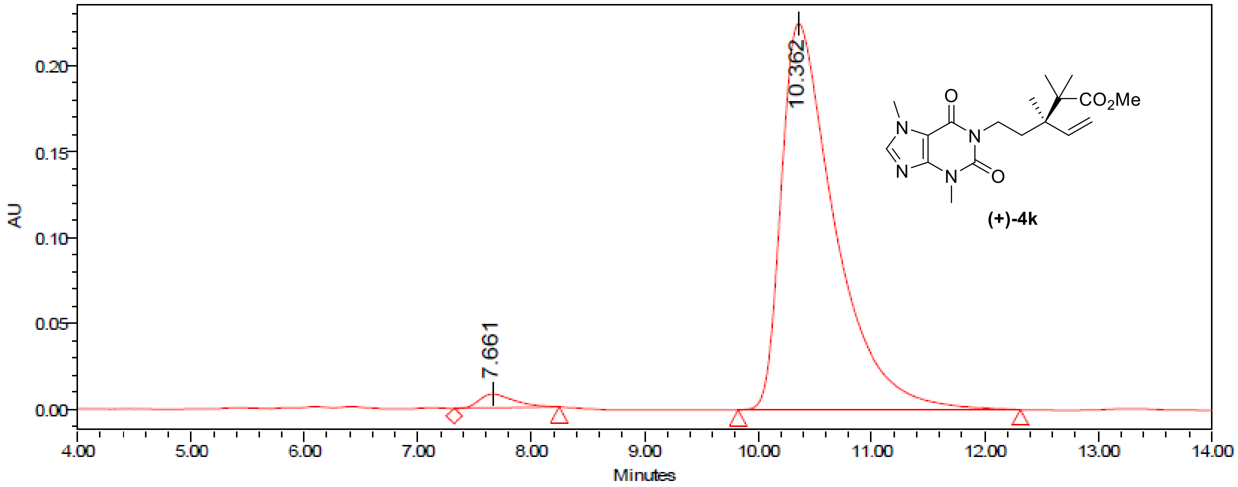

Peak Results

	Name	RT	Area	% Area	Height	Units
1		7.661	173389	2.34	7885	
2		10.362	7231435	97.66	224985	

Racemic sample (Daicel Chiralpak AD-H, 5% *i*-PrOH, 1.0 mL/min, 25 °C, 215 nm)

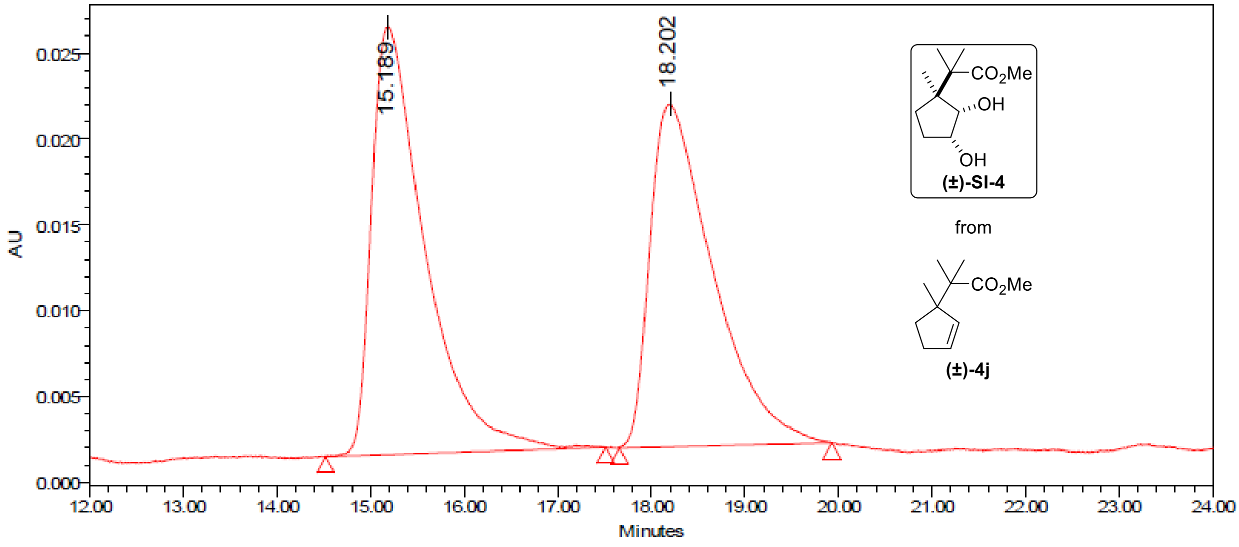

Peak Results

	Name	RT	% Area	Height	Amount	Units
1		15.189	49.07	24915		
2		18.202	50.93	19964		

(–)-SI-4

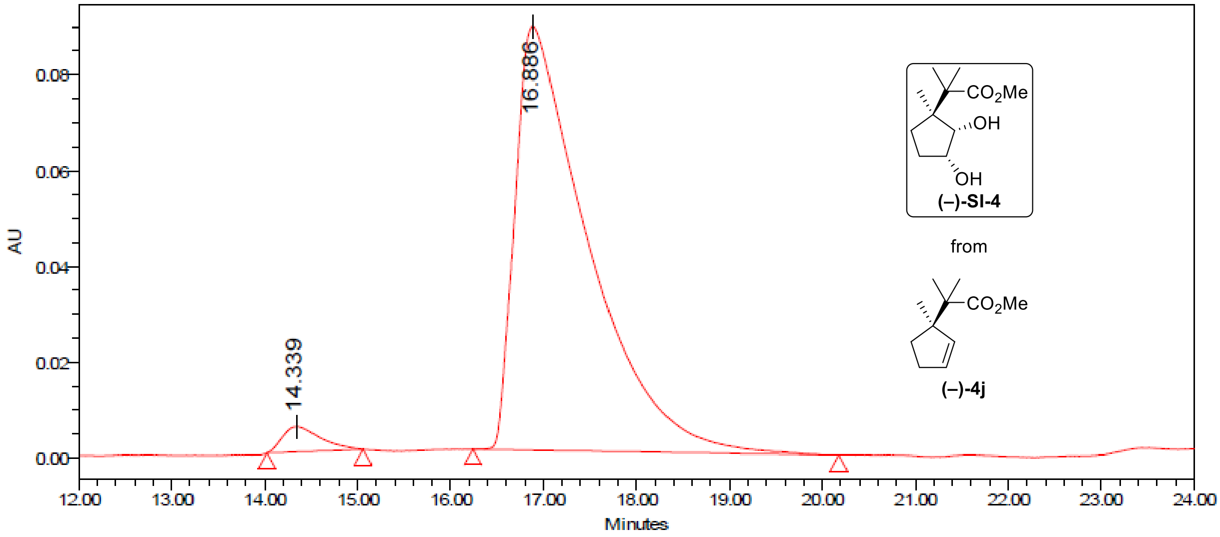

Peak Results

	Name	RT	% Area	Height	Amount	Units
1		14.339	3.04	5263		
2		16.886	96.96	88486		

9. X-Ray Crystallographic Data

X-Ray Crystallographic Data for 3i

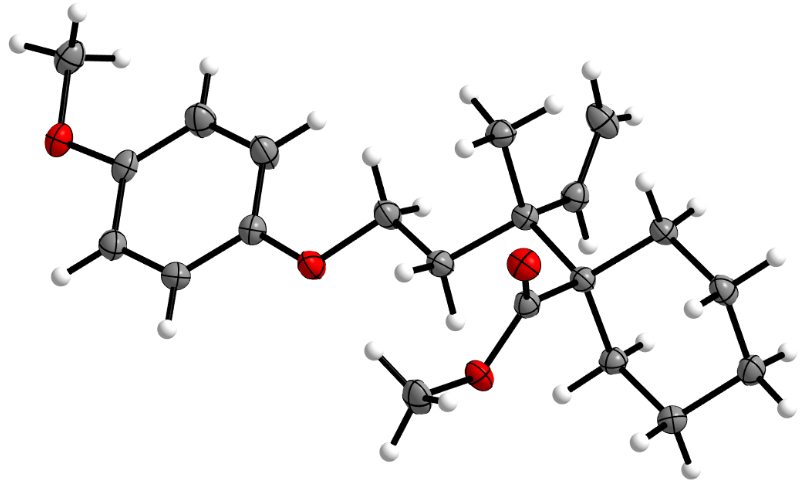

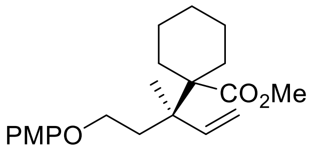

3i

CCDC deposition number	2334068
Empirical formula	$C_{21}H_{30}O_4$
Formula weight	346.45
Temperature/K	100.0(1)
Crystal system	orthorhombic
Space group	$P2_12_12_1$
a/Å	6.19436(4)
b/Å	8.51383(7)
c/Å	35.9236(4)
α/°	90
β/°	90
γ/°	90
Volume/Å^3	1894.53(3)
Z	4
ρ_{calc}g/cm^3	1.215
μ/mm^{-1}	0.659
F(000)	752.0
Crystal size/mm^3	0.159 × 0.137 × 0.059
Radiation	Cu Kα (λ = 1.54184)
2Θ range for data collection/°	4.92 to 149.452
Index ranges	$-7 \le h \le 7, -10 \le k \le 10, -37 \le l \le 43$

Reflections collected	66815
Independent reflections	3802 [R_{int} = 0.0264, R_{sigma} = 0.0093]
Data/restraints/parameters	3802/0/229
Goodness-of-fit on F^2	1.049
Final R indexes [I>=2σ (I)]	R_1 = 0.0317, wR_2 = 0.0845
Final R indexes [all data]	R_1 = 0.0322, wR_2 = 0.0848
Largest diff. peak/hole / e $Å^{-3}$	0.18/-0.26
Flack parameter	0.02(4)

X-Ray Crystallographic Data for 3j

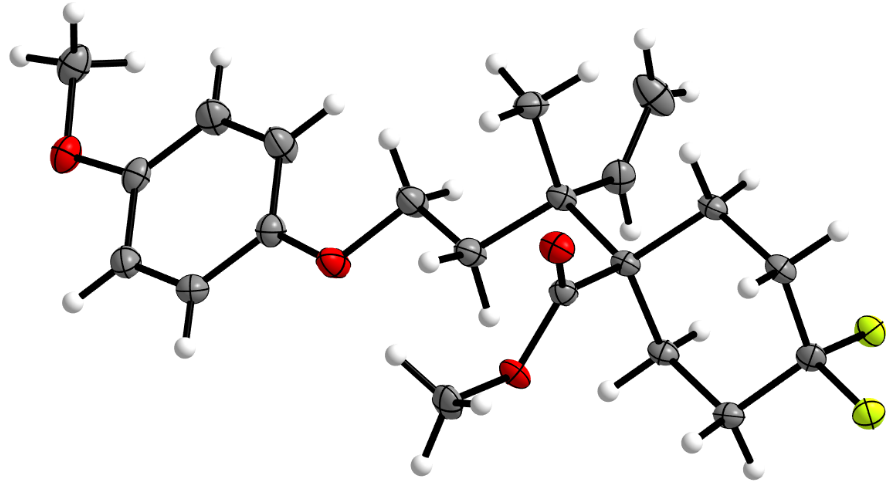

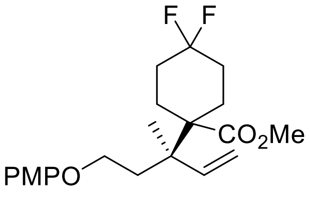

3j

CCDC deposition number	2334069
Empirical formula	$C_{21}H_{28}O_4F_2$
Formula weight	382.43
Temperature/K	100.0(1)
Crystal system	orthorhombic
Space group	$P2_12_12_1$
a/Å	6.19082(3)
b/Å	8.67538(5)
c/Å	36.5474(2)
α/°	90
β/°	90
γ/°	90
Volume/Å^3	1962.880(19)
Z	4
ρ_{calc}g/cm^3	1.294
μ/mm^{-1}	0.839
F(000)	816.0
Crystal size/mm^3	0.15 × 0.108 × 0.083
Radiation	Cu Kα (λ = 1.54184)
2Θ range for data collection/°	4.836 to 149.648
Index ranges	$-7 \leq h \leq 7$, $-10 \leq k \leq 10$, $-45 \leq l \leq 45$
Reflections collected	53435
Independent reflections	3935 [R_{int} = 0.0201, R_{sigma} = 0.0081]

Data/restraints/parameters	3935/0/247
Goodness-of-fit on F^2	1.043
Final R indexes [I>=2σ (I)]	R_1 = 0.0260, wR_2 = 0.0655
Final R indexes [all data]	R_1 = 0.0265, wR_2 = 0.0659
Largest diff. peak/hole / e $Å^{-3}$	0.26/-0.17
Flack parameter	-0.02(2)

10. References

(1) Zhao, Y.; He, Z.; Li, S.; Tang, J.; Gao, G.; Lan, J.; You, J. An air-stable half-sandwich Ru^{II} complex as an efficient catalyst for [3+2] annulation of 2-arylcyclo-2-enones with alkynes. *Chem. Commun.* **2016**, *52*, 4613–4616.

(2) Closs, G. L.; Closs, L. E. Carbenes from Alkyl Halides and Organolithium Compounds. I. Synthesis of Chlorocyclopropanes. *J. Am. Chem. Soc.* **1960**, *82*, 5723–5728.

(3) Klein, J. Electronic Effects on the Stereochemistry of Attack on Trigonal Atoms in Six Membered Rings. *Tetrahedron* **1974**, *30*, 3349–3350.

(4) Corey, E. J.; Guzman-Perez, A.; Noe, M. C. The Application of a Mechanistic Model Leads to the Extension of the Sharpless Asymmetric Dihydroxylation of Allylic 4-Methoxybenzoates and Conformationally Related Amine and Homoallylic Alcohol Derivatives. *J. Am. Chem. Soc.* **1995**, *117*, 10805–10816.

(5) Hugelshofer, C. L.; Magauer, T. A General Entry to Antifeedant Sesterterpenoids: Total Synthesis of (+)-Norleucosceptroid A, (–)-Norleucosceptroid B, and (–)-Leucosceptroid K. *Angew. Chem. Int. Ed.* **2014**, *53*, 11351–11355.

(6) Meylemans, H. A.; Quintana, R. L.; Goldsmith, B. R.; Harvey, B. G. Solvent-Free Conversion of Linalool to Methylcyclopentadiene Dimers: A Route To Renewable High-Density Fuels. *ChemSusChem* **2011**, *4*, 465–469.

(7) Roosen, P. C.; Vanderwal, C. D. A Formal Synthesis of 7,20-Diisocyanoadociane. *Angew. Chem. Int. Ed.* **2016**, *55*, 7180–7183.

(8) Kiuchi, F.; Itano, Y.; Uchiyama, N.; Honda, G.; Tsubouchi, A.; Nakajima-Shimada, J.; Aoki, T. Monoterpene Hydroperoxides with Trypanocidal Activity from *Chenopodium ambrosioides*. *J. Nat. Prod*. **2002**, *65*, 509–512.

(9) Stymiest, J. L.; Bagutski, V.; French, R. M.; Aggarwal, V. K. Enantiodivergent conversion of chiral secondary alcohols into tertiary alcohols. *Nature* **2010**, *456*, 778–783.

(10) Evans, P. A.; Oliver, S. Regio- and Enantiospecific Rhodium-Catalyzed Allylic Substitution with an Acyl Anion Equivalent. *Org. Lett.* **2013**, *15*, 5626–5929.

(11) Zhang, P.; Kyne, R. E.; Morken, J. P. Enantioselective Construction of All-Carbon Quaternary Centers by Branch-Selective Pd-Catalyzed Allyl–Allyl Cross-Coupling. *J. Am. Chem. Soc.* **2011**, *133*, 9716–9719.

(12) Mills, L. R.; Di Mare, F.; Gygi, D.; Lee, H.; Simmons, E. M.; Kim, J.; Wisniewski, S. R.; Chirik; P. J. Phenoxythiazoline (FTz)-Cobalt(II) Precatalysts Enable C(sp^2)–C(sp^3) Bond-Formation for Key Intermediates in the Synthesis of Toll-like Receptor 7/8 Antagonists. *Angew. Chem. Int. Ed.* **2023**, *62*, e202313848.

(13) Wailes, J.; Black, J.; Morris, J.; Briggs, E.; Tate, J.; Aspinall, M.; Ng, S.; Herbicidal Compounds. WO2020/094524 A1, 2020.

(14) Wu, Y.; Zhou, B. Rhodium(III)-Catalyzed Selective C-H Acetoxylation and Hydroxylation Reactions. *Org. Lett.* **2017**, *19*, 3532–3535.

(15) Liu, J.; Su, X.; Han, M.; Wu, D.; Gray, D. L.; Shapley, J. R.; Wert, C. J.; Strathmann, T. J. Ligand Design for Isomer-Selective Oxorhenium(V) Complex Synthesis. *Inorg. Chem.* **2017**, *56*, 1757–1769.
